# Impact of the severity of negative energy balance on gene expression in the subcutaneous adipose tissue of periparturient primiparous Holstein dairy cows: Identification of potential novel metabolic signals for the reproductive system

**DOI:** 10.1371/journal.pone.0222954

**Published:** 2019-09-26

**Authors:** Namya Mellouk, Christelle Rame, Delphine Naquin, Yan Jaszczyszyn, Jean-Luc Touzé, Eric Briant, Daniel Guillaume, Theodoros Ntallaris, Patrice Humblot, Joëlle Dupont

**Affiliations:** 1 INRA UMR85 Physiologie de la Reproduction et des Comportements, Nouzilly, France; 2 CNRS UMR7247 Physiologie de la Reproduction et des Comportements, Nouzilly, France; 3 Université François Rabelais de Tours F-37041 Tours, Nouzilly, France; 4 Institute for Integrative Biology of the Cell (I2BC), CEA, CNRS, Université Paris-Sud, Gif-sur-Yvette, France; 5 INRA, UEPAO 1297, Nouzilly, France; 6 Division of reproduction, Department of Clinical Sciences, SLU, Uppsala, Sweden; Hull York Medical School, UNITED KINGDOM

## Abstract

The severity of negative energy balance (NEB) in high-producing dairy cows has a high incidence among health diseases. The cow’s energy status during early lactation critically affects metabolic and reproductive parameters. The first objective of this study was to investigate by RNA-seq analysis and RT-qPCR the gene expression profile in white adipose tissue and by gene ontology and upstream regulation tools the relationships with energy metabolism and reproduction in two groups of primiparous dairy cows with extreme NEB statuses (NEB < -9 Mcal/day vs. NEB > -9 Mcal/day) around parturition. The second objective was to determine the potential involvement of a new adipokine identified as a candidate for the regulation of ovarian function in our RNA-seq analysis by using bovine primary granulosa culture, thymidine incorporation to determine cell proliferation and ELISA assays to measure progesterone secretion. The RNA-seq analysis revealed that 514 genes were over-expressed and 695 were under-expressed in the adipose tissue of cows with severe NEB (SNEB) and cows with moderate NEB (MNEB) during the -4 and 16 wkpp period. In addition, 491 genes were over-expressed and 705 genes were under-expressed in the adipose tissue of SNEB cows compared to MNEB cows. Among these differently expressed genes (DEGs), 298 were related to metabolic functions and 264 to reproductive traits. A set of 19 DEGs were validated by RT-qPCR, including *CCL21* (C-C motif chemokine ligand 21). Moreover, *CCL21*, a gene known to be secreted by adipose tissue, was chosen for further analysis in plasma and ovaries. The use of next-generation sequencing technologies allowed us to characterise the transcriptome of white adipose tissue from primiparous cows with different levels of NEB during lactation. This study highlighted the alteration of the expression of genes related to lipid metabolism, including *CCL21*, which is released in the bloodstream and associated with the *in vitro* regulation of ovarian functions.

## Introduction

The periparturient period is crucial for the health status and reproductive performance of dairy cows [1]. At the beginning of lactation, the energy needs for milk production are higher than the available energy consumed from feed intake resulting in a negative energy balance (NEB). This leads to fat mobilisation and consequently elevated plasma concentrations of non-esterified fatty acids (NEFAs), which are used as a fuel source by peripheral tissues and the mammary gland for milk fat synthesis, [2, 3]. However, fat mobilisation may induce various disorders such as inflammatory diseases by reducing immune function [4], displacement of the abomasum [5] and several metabolic disorders, such as ketosis and fatty liver [6–8]. Excessive lipolysis during the early lactation period has also been associated with several reproductive dysfunctions including placental retention, metritis and endometritis [9] and low energy related to changes in immune gene expression in the endometrium [10]. The relationships between lipid metabolism and fertility are well documented in the dairy cow [11], but mechanisms that mediate the effects of energy/lipid metabolism on reproductive parameters need to be unraveled. During the transition period and the onset of lactation, white adipose tissue (WAT) is the main metabolic organ involved in fat mobilization and consequently for plasma NEFA changes induced by the NEB in dairy cows. Consequently, better understanding of the WAT metabolic regulatory function during the periparturient period could contribute to have more robust animals by improving lactation and reproductive performances and by reducing disease incidence.

WAT is a plastic organ and an important regulator of whole-body metabolism in response to the energy status of the animal. Indeed it contributes to the control of energy storage by regulating fatty acids anabolism or catabolism, and the release of molecules called adipokines [12]. In dairy cattle, the link between adipose tissue lipogenesis and lipolysis with milk production has been well documented [13, 14]. Early lactation is associated with intensive lipid mobilisation, an increase of circulating NEFAs levels and the loss of body weight and back fat thickness. Conversely, late lactation and the dry period are frequently accompanied by lipid synthesis accumulation in WAT [15, 16]. Transcriptomic analysis of bovine adipose tissue has indicated the differential expression of a large number of genes that control the use of nutrients and metabolites, inflammation and the immune processes, cellular synthesis, tissue remodeling and angiogenesis during lactation [17]. One way to limit the dramatic changes in WAT during early lactation is to find new nutritional strategies that may lower the amplitude of the NEB. Several transcriptomic data on adipose tissue from cattle fed differently have been reported in the recent years [18, 19]. Most of the them were obtained from microarrays or quantitative PCR, which are less sensitive and less exhaustive than next-generation RNA sequencing [20]. Only one recent study using this latter technique showed the importance of adipose transcriptional regulation of metabolism in growing cattle [21]. Over the last decade, there has been new interest in the role of adipokines on the reproductive tract. For instance, an *in vitro* treatment with recombinant RESISTIN, ADIPONECTIN or CHEMERIN decreases the progesterone and estradiol secretions by primary bovine granulosa cells [22–24]. Conversely, recombinant VISFATIN increases basal and IGF1-induced steroidogenesis in cultured bovine granulosa cells [25]. Recent data showed that the plasma profiles of novel adipokines, such as RESISTIN, ADIPONECTIN and CHEMERIN varied during the periparturient period in dairy cows [26–29]. Furthermore, the *in vitro* effects of these adipokines were described in reproductive tissues in cattle [22–24]. Thus, transcriptomic analysis of adipose tissue could identify new adipokines secreted by WAT that are susceptible to act simultaneously on energy metabolism and reproductive functions.

In a previous study, we showed that the energy content of the diet could alter the plasma profiles of various novel adipokines, including resistin, adiponectin and chemerin in primiparous dairy cows [24, 26, 30]. From this study, we selected animals with an extreme energy balance phenotype during the postpartum period to analyse gene expression changes related to NEB in subcutaneous white adipose tissue on a whole genome basis. Differentially expressed genes (DEGs) and pathways mainly related to metabolic functions were identified according to the different energy states and times around calving. The *in silico* analysis of DEGs allowed us to discover new secreted candidates related to lipid metabolism; for one of them, CCL21, we characterised the plasma profile and its expression and role in the bovine ovary *in vitro*.

## Materials and methods

### Ethical issues

An ethics committee (Comité d’Ethique en Expérimentation Animale Val de Loire, CEEA VdL), approved all experimental protocols (protocol reference number 2012-10-4), which were consistent with the guidelines provided by the French Council for Animal Care.

### Animals

The animals originated from the experimental unit UEPAO (Institut National de la Recherche Agronomique, Nouzilly, France), where the study took place from September 2012 to January 2015. Thirty-nine animals were managed in a straw-bedded yard and fed either a high energy (HE) diet calculated to yield 35 kg of milk/cow per day (n = 17) or a low-energy (LE) diet calculated to yield 25 kg of milk/cow per day (n = 22). The diets started 4 weeks before the presumed first calving date (-4 wkpp) and were provided until the dry period (**S1 Fig**). The compositions of HE and LE diets are indicated in **S1 Table**.

### Measurement of live body weight, variation of empty body weight, milk yield, dry matter intake, back fat thickness and energy balance (EB)

During the dry period, the animal housing was not equipped with defined feeders, so it was not possible to determine energy balance (EB. Thus, animals were kept in groups in loose housing where the total DMI from feed was estimated from the given amount. After calving, cows were milked twice daily and weighed automatically after each milking (software RIC version RW1.7). Only live body weight (LBW) data measured following morning milking were used in subsequent analyses because the afternoon LBW was submitted to high variations. As LBW is affected by digestive contents, the estimation of empty body weight (EBW) was corrected for the digestive tract content. A change of 4.5 kg of digestive contents per kg of DMI was assumed [31]. Variation of EBW (VEBW) was calculated d after d: EBW of the previous d was taken as reference weight. Live body weight and VEBW as compared to one month before calving were measured from—4 wk peripartum (wkpp) to 16 wkpp in females during their first lactation. All cows were milked twice daily. At the entrance of the milking parlour, cows were identified by an electronic collar and milk yield (MY) of each cow was automatically recorded (software R-Manufeed 500 pro, vc5 version 2.011.14, 1996, France). Dry matter intake was determined from the intake of fresh matter and the DM content of each feed of the ration. The diet was distributed twice daily, at 9 am and at 3 pm, and each cow had access to several defined feeders (Insentec B.V., Marknesse, The Netherlands). On average, there was one feeder for two cows. When a cow arrived in front of the feeder, it was recognized by a unique passive transponder attached to her ear tag. If the cows were allowed, the feeder opened and the quantity of food eaten was automatically recorded (software RIC version RW1.7). Animals had access to their diet all the time. Dry matter intake was calculated daily for the HE and LE diets, from calving to 16 wkpp. The chemical composition of each feed is shown **in S1 Table.**

The nutritional values of the different ingredients composing the diets were available from chemical analysis. Energy balance (expressed in Mcal/d) was calculated during the lactating period only (from calving to 16 wkpp), as it was not possible to measure DMI during the dry period. It was calculated at 1 and 16 wkpp according to the INRA feeding systems as described in [30] as the difference between the energy intake and the energy requirements for maintenance, MY and pregnancy. According to the INRA system, the daily requirement for maintenance is 1.1 * 0.041 * kg^0.75^, and the requirement for MY is 0.44 * MY. Energy intake and EB are expressed in Mcal/d, where kg^0.75^ indicates metabolic body weight as described in [30]. From these 39 animals, we selected two sub-groups of animals either with severe NEB (SNEB, n = 22) or with moderate NEB (MNEB, n = 17) according to their EB at 1 wkpp (EB < -9 Mcal/day for SNEB and EB > -9 Mcal/day for MNEB). All observations, sampling and data registration took place during the animals’ first lactations at different energy mobilisation time points (-4, 1 and 16 wkpp). The animals were kept alive after the study.

### Subcutaneous adipose tissue thickness

Adipose tissue mobilization was assessed through subcutaneous fat thickness measurements in the sacral region using ultrasonographic examination with a linear probe (LA 332 3.5/10.0- MHz transducer; Mylab30vet; R-Esaote, Hospimedi, Saint-Crépin-Ibouvillers, France), as previously described [30]. Back fat thickness (BFT) was determined at -4, 1 and 16 wkpp.

### Subcutaneous adipose tissue sampling

Cows were fasted for 12 h before surgery and anaesthesia was induced by intravenous (IV) injections of 12 to 14 mg of xylazine (Rompun, Bayer, Leverkusen, Germany). Subcutaneous (SC) fat was collected from the dewlap under the neck and immediately frozen in liquid nitrogen. Briefly, the area of each biopsy sampling point was shaved, washed and disinfected with 70% ethanol and iodine. Infiltration anaesthesia with 20 mg lidocaine (Lurocaïne, Vetoquinol, Lure, France) was applied. A 1.5 cm skin incision was made at the dewlap under the neck about 20 minutes after the infiltration. After the sampling (about 5 minutes), the wounds were closed by a suture, treated with aluminum spray and monitored every day until healing. Subcutaneous adipose tissue biopsies were conducted from each animal at -4, 1 and 16 wkpp (**S2 Fig**).

### Plasma metabolite assays

Blood samples were taken from the coccygeal vein with heparinised Vacutainers (Dutcher, Brumath, France) before diet distribution at -4, 1 and 14 wkpp. Blood was immediately centrifuged (2,000 *g* for 15 min at 4°C) and the separated plasma was stored at −20°C until assay. Plasma non esterified fatty acids (NEFAs) and glucose were determined by enzymatic colorimetry assays (Wako Chemicals GmbH, Neuss, Germany; Sigma Aldrich, Saint Quentin-Fallavier, France). The intra- and inter-assay coefficients of variation (CV) of both plasma fatty acids and glucose measures were 6 and 7.8%, respectively. The plasma concentration of insulin was measured by RIA from 100 μL of undiluted plasma, as previously described [32]. The plasma concentration of CCL21 was determined using a bovine Elisa Kit (MBS2610928, My BioSource, San Diego, USA). The intra- and inter-assay CV were 8 and 12%, respectively.

### Reproductive parameters

In the above sub-groups of cows (n = 17 and n = 22 for the MNEB and SNEB groups, respectively), all procedures for the measurement of reproductive parameters were conducted as previously described [30]. Briefly, during the cycle before artificial insemination (AI), the ovarian follicular dynamics of primiparous cows were monitored three times per week by transrectal ultrasonography using a linear probe (LV 513 6.0/8.0-MHz transducer; Mylab30; Esaote) allowing for the detection, counting and measurement of small (SF, 3–5 mm), medium (MF, > 5 and ≤ 7 mm) and large follicles (LF, > 7 mm). Based on these observations, the growth of each follicle was followed and the number of follicular waves (0,1,2,3 or > 4) was determined as described by [33]. The mean number of follicles in each class per cow was calculated from the total number of follicles of a given class from both ovaries (SF, MF, or LF) divided by the number of ultrasonographic examinations. The length of the cycle was calculated by combining the results from ovarian scans and data from oestrus detection. After AI, ovulation was confirmed by measuring progesterone three times per week until 21 days post-AI. The postpartum commencement of luteal activity (CLA) was defined as the first day that the plasma progesterone concentration was > 0.70 ng/mL; this threshold was defined from our full data set as the value at which 95% of the minimum concentrations flanking the supposed day of oestrus were higher than this concentration. Pregnancy was determined on day 35 by ultrasonography and on day 90 by transrectal palpation. The pregnancy rate was calculated as the number of pregnant females divided by the total number of inseminated females.

### RNA extraction, library preparation and RNA sequencing

Total RNA from the fat tissue of three cows per group (with the more extreme NEB at 1 wkpp; SNEB: -17.30, -13.15, -17.84 Mcal/day and MNEB: -0.59, -2.20, -2.97 Mcal/day, respectively) was extracted using a RNeasy Midi kit (Qiagen®, Courtaboeuf, France) and purified using a DNAfree kit (Invitrogen by Life Technologies), according to the manufacturer’s recommendations. Total RNA quality was assessed with an Agilent Bioanalyzer 2100, using a RNA 6000 pico kit (Agilent Technologies). Directional RNA-Seq libraries were constructed using the TruSeq Stranded Total RNA library prep kit (Illumina), following the manufacturer’s instructions, using 750 ng of total RNA. After the Ribo-Zero step, samples were checked with an Agilent Bioanalyzer for proper rRNA depletion. The quality of the final libraries was assessed with an Agilent Bioanalyzer, using an Agilent High Sensitivity DNA Kit. Libraries were pooled in equimolar proportions and sequenced using two single read 75 pb runs, on an Illumina NextSeq500 instrument, using NextSeq 500 High Output 75 cycles kits.

### Sequence data filtering

Demultiplexing was done (bcl2fastq2 V2.15.0) and adapters were removed (Cutadapt1.3); only reads longer than 10 pb were kept for analysis.

### Read mapping and gene identification

Reads were mapped onto the cow’s reference genome bosTau8.fa (downloaded from the UCSC database, http://hgdownload.soe.ucsc.edu/goldenPath/bosTau8/bigZips/) with TopHat2 (using default parameters). The corresponding filtered annotation file from UCSC contained 13,896 genes. Since samples were treated with a ribosomal depletion protocol rather than a polyA selection of mRNAs, a high proportion of reads was found to map on introns. However, it has been shown that there is a strong correlation of the read coverage levels between exon and intron [34]. Thus differential expression analysis was performed twice, either only on exonic parts of each gene or on the entire gene sequences. Mapped reads were assigned to features with htseq-count (intersection-nonempty mode) (**S2 Table**).

### Differential expression analysis

Normalization of read contents and differential expression analysis were performed with DESeq2, both for the mRNA and for the whole gene differential expression analysis. Genes with a FDR-adjusted P-value < 0.01 (Benjamini-Hochberg method) were considered statistically significant. The differentially expressed genes common to the two analyses were selected.

### Bioinformatics analysis of DEGs

Differentially expressed genes (DEGs) according to week (-4 vs. 1 wkpp or 1 vs. 16 wkpp) were determined from the RNAseq data collected at three time points (-4, 1 and 16 wkpp) and analyses were performed separately for the two NEB groups. DEGs related to the NEB status (SNEB vs MNEB) were determined from RNAseq data generated at 1 and 16 wkpp. The variation in gene expression was determined according to DESeq analysis. The over-expressed genes were genes that were highly expressed at -4 compared to at 1 wkpp, or at 1 compared to at 16 wkpp, or in SNEB cows compared to MNEB cows. The under-expressed genes were genes that were less expressed at -4 compared to at 1 wkpp, or at 1 compared to 16 wkpp, or in SNEB cows compared to MNEB cows (**S1 Fig**).

### Gene network analysis and biomarker identification

A downstream effects analysis including a gene network analysis was performed in 2016 using the Ingenuity Pathway Analysis propriety tool (IPA; http://www.ingenuity.com, Redwood City, CA). The purpose of downstream effects analysis is to identify biological processes and functions that are likely to be causally affected by up- and down-regulated genes. In addition, it is also predicted whether those processes are increased or decreased as described by [35]. The build version and the content version used were 366632M and 26127183 (release date 2015-11-30), respectively. The IPA database included those following data sources: BIND, BIOGRID, Cognia, DIP, Gene Ontology (GO), Ingenuity Expert Findings, Ingenuity ExpertAssist Findings, INTACT, Interactome studies, MINT, MIPS, Online Mendelian Inheritance in Man (OMIM). The calculation of the overlap *P*-value and the activation Z-score is essentially the same as in standard enrichment functional analysis, based upon Fischer’s exact test, and is well detailed by [35].Consequently, only direct interactions (known interactions from published studies with humans and rodents referenced in the IPA database) and metabolic downstream biological processes that are increased or decreased based on gene expression results (filtered under the “diseases & functions” section) among the genes analysed in the present study were included in the analyses for the identification of main biological functions and gene networks. Relevant biomarker candidates were also identified in 2018 using IPA under preset parameters, and references that indicate expression or function related to reproductive parameters were extracted from the NCBI database.

### Quantitative real time RT-PCR

The total RNA of subcutaneous adipose tissue (n = 7 per NEB group, picked randomly) and ovarian tissue (n = 6) was extracted by homogenisation in the TRIzol® reagent using an Ultraturax (Invitrogen ™ by Life Technologies ™, Villebon sur Yvette, France) and extracted using the RNeasy Midi kit (Quiagen®, Courtaboeuf, France), according to the manufacturer's recommendations. The concentration and purity of isolated RNA were determined with a NanoDrop spectrophotometer (Peqlab Biotechnologie GmbH, Erlangen, Germany). The integrity of RNA was checked on 1.25% agarose-formaldehyde gels. The cDNA was generated by reverse transcription (RT) of total RNA (1 μg) in a mixture comprising: 0.5 mM each of deoxyribonucleotide triphosphate (dATP, dGTP, dCTP, DTTP) 2M of RT buffer, 15 μg/μL of oligodT, 0.125 U of ribonuclease inhibitor and 0.05 U MMLV (Moloney murine leukemia virus reverse transcriptase) for 1 hour at 37°C.

Real-time PCR was performed using the MyiQ Cycle device (Bio-Rad, Marnes-la-Coquette, France), in a mixture of SYBR Green Supermix 1X reagent (Bio-Rad, Marnes la Coquette, France), 250 nM specific primers (Invitrogen ™ by Life Technologies ™, Villebon sur Yvette, France) (**S3 Table**) and 5 μL of cDNA diluted by a fifth, for a total volume of 20 μL.

For each biological sample, analyses were performed from duplicates on the same plate. PCR amplification with water, instead of cDNA, was performed systematically as a negative control. After incubation for 2 min at 50°C and a denaturation step of 10 min at 95°C, samples were subjected to 40 cycles (30 s at 95°C, 30 s at 60°C, 30 s at 72°C), followed by the acquisition of the melting curve. For each gene, expression was calculated according to primer efficiency (E) and quantification cycle (Cq), where expression = E^-Cq^. Then, the expression of the target gene relative to the reference gene was analysed. The levels of mRNA expression were standardised to the geometric mean of three reference genes (*UXT*, *SADH* and *PPIA*), which has been reported as an accurate normalisation factor [36]. Comparisons for differential expression between the two energy balance statuses were performed as described above.

### Protein extraction and Western blot

Subcutaneous adipose tissue from six animals per group, picked randomly at different time points (-4, 1 and 16 wkpp), were lysed using an Ultraturax (Invitrogen TM by Life Technologies TM, Villebon sur Yvette, France) in lysis buffer (Tris 1 M (pH 7.4), NaCl 0.15 M, EDTA 1.3 mM, EGTA 1 mM, VO43− 23 mM, NaF 0.1 M, NH2PO4 1%, Triton 0.5%). The lysates were centrifuged for 20 min at 16,000 *g* at 4°C and the supernatant containing proteins was collected and kept on ice. The protein concentration was measured using a bicinchoninic acid (BCA) protein assay (Interchim, Montluçon, France). Lysate protein (60 μg) was mixed with Laemmli buffer 5 X and proteins were denatured for 5 min by heat shock at 95°C. Proteins were loaded in an electrophoresis sodium dodecyl sulphate-polyacrylamide gel (12%). Then, the proteins were transferred to a nitrocellulose membrane. Membranes were blocked with Tris-Buffered Saline Tween buffer containing 0.05% of Tween 20 and 5% of milk for 30 min at room temperature. Membranes were incubated overnight at 4°C with anti-CCL21 (Thermofischer, Villebon sur Yvette, France) or anti-vinculin antibodies (Sigma, Aldrich, Saint Quentin Fallavier, France). Then, membranes were incubated for 90 min at room temperature with horseradish peroxidase (HRP)-conjugated anti-rabbit or anti-mouse IgG (Sigma, Aldrich, Saint Quentin Fallavier, France). Proteins of interest were detected by enhanced chemiluminescence (Western Lightning Plus-ECL, Perkin Elmer, Villebon-sur-Yvette, France) with G-box 10 SynGene (Ozyme, St. Quentin en Yvelines, France) and GeneSnap software (Ozyme, St. Quentin en Yvelines, France). Then, proteins were quantified with GeneTools software. The results were expressed as the intensity signal in arbitrary units after normalisation.

### Immunohistochemistry

Bovine ovary sections (n = 4 from 8 cows) from a slaughterhouse were fixed, then deparaffinised, hydrated, and microwaved for 5 min in an antigen unmasking solution (Vector Laboratories Inc., AbCys), then allowed to reach room temperature. Sections (7 μm) were washed for 5 min in PBS then incubated in a peroxidase-blocking reagent for 10 min at room temperature in order to inhibit endogenous peroxidase activity (DakoCytomation). After two 5 min washes in PBS, the non-specific background was eliminated by blocking with 5% lamb serum in PBS for 20 min, followed by an overnight incubation at 4°C with PBS containing a rabbit primary antibody raised against CCL21 (1:100; Sigma, Aldrich, Saint Quentin Fallavier, France). Sections were washed in PBS twice for 5 min each, then incubated for 30 min at room temperature with a ready-to-use labeled polymer-HRP anti-rabbit antibody (EnVision Plus HRP system; DakoCytomation). The sections were washed in PBS twice, and staining was revealed after incubation at room temperature with 3,3-diaminobenzidine tetrahydrochloride (DAB) (Liquid DAB+ Substrate Chromogen System; DakoCytomation). The negative controls were prepared by replacing the primary antibodies with rabbit IgG.

### Primary bovine granulosa cells culture

Bovine ovaries (about 40 ovaries for one culture) were obtained from adult cows collected at a local slaughterhouse and transported aseptically before dissection. Granulosa cells were recovered from small antral follicles (3–5 mm, SF) in modified McCoy’s 5A medium, followed by 5 min centrifugation. Cells were washed, counted in a haemocytometer and cultured in McCoy’s 5A supplemented with 20 mmol/L HEPES, penicillin (100 U/mL), streptomycin (100 mg/L), l-glutamine (3 mmol/L), 0.1% BSA, 5 mg/L transferrin, 20 mg/L selenium, 0.1 μmol/L androstenedione and 10% fetal bovine serum (FBS, PAA Laboratories, les Mureaux, France). Approximately 4×10^5^ viable cells were seeded in each plastic multi-well containing 1 mL of medium. After 24 h of culture, cells were starved with McCoy’s 5A medium containing 1% FBS overnight, and then incubated in fresh culture medium with 10, 50, 100, 200 ng/mL, or without the recombinant human CCL21 (Bio-techne, Lille, France) for 24 or 48 hours. The human protein sequence of CCL21 shares 73% identify with the bovine sequence. All cultures were performed in a water-saturated atmosphere of 95% air/5% CO_2_ at 37°C.

### Proliferation assay

Primary bovine granulosa cells were cultured for 24 h in McCoy’s 5A medium and 10% fetal bovine serum (FBS). Cells were plated in 24-well plates (2×10^5^ viable cells/well) and four replicates from four independent cultures were tested for each experimental condition (with or without recombinant human CCL21: 10, 50, 100, 200 ng/mL) for each culture. After several washes and overnight serum starvation, the cells were cultured for 24 h with 1 μCi/μL [3H] thymidine (Amersham Life Science) in the presence or absence of CCL21. Thymidine was then removed with PBS and the cells were fixed with cold 50% trichloroacetic acid for 15 min on ice. Finally, the cells were lysed using 0.5N NaOH and radioactivity was counted in a β-photomultiplier by adding scintillation fluid (Packard Bioscience).

### Progesterone ELISA assay

The progesterone concentration in serum-free medium from primary bovine granulosa cells was measured after 48 h of treatment using ELISA assays [37]. The limit of detection for progesterone was 12 pg/tube. The intra- and inter-assay coefficients of variation for progesterone were less than 10% and 11%, respectively. The results were expressed as the concentration of progesterone (ng/mL) per mg of protein per well. Data were obtained from three independent cultures, and each treatment (recombinant human CCL21: 10, 50, 100, 200 ng/mL) had four replicates.

### Statistical analyses

Results are represented as least squares means (LSM) ± SEM; differences with *P* ≤ 0.05 were considered significant. If not specified, phenotypic data were analysed using the MIXED procedure for linear mixed models; we included “cow” as a variable using SAS software (version 9.3; SAS Institute Inc., Cary, NC). A repeated effect of time (wkpp) within animals was tested. The residuals from the observations generated from the mixed models were tested for normal distribution. The statistical model included the fixed effect of week for both experiment and diet, week and diet × week interaction, as previously described [30]. A Chi-square test was used to analyse the success rate after AI after 35 and 90 days and the percentage of follicular waves. A Pearson test was used to analyse correlations between reproductive parameters and plasma concentrations of CCL21. The area under the curve (AUC) of plasma CCL21 concentrations was performed as the sum of linear AUC, calculated according to the following formula: AUC = x (t1) + y (t2) × 2/(t2 –t1), with x and y as a value of plasma CCL21 concentration at two time points (t1 or t2). The correlation was noted (‘r’) and the p-value was considered significant if *P* < 0.05.

## Results

### Effect of different levels of negative energy balance on metabolic indicators

All evaluated parameters (DMI, EB, LBW, VEBW, MY, BFT) and the plasma concentration of NEFA and insulin were affected by week (*P* < 0.0001), except for the plasma concentration of glucose (**Table 1**). A NEB group effect on DMI, EB, LBW and the plasma concentration of insulin (*P* < 0.05) was also found. During this study, animals were selected according to their energy balance. As expected, SNEB animals had more severe NEB, compared to MNEB animals at 1 week postpartum (wkpp), and there was an interaction between group and week. Moreover, we observed that SNEB animals had lower DMI and LBW than MNEB animals at 1 and 16 wkpp (**Table 1**). Differences in the plasma concentrations of insulin were more pronounced at -4 wkpp, with a lower level in SNEB animals than in MNEB animals (0.64 ± 0.09 vs. 0.79 ± 0.10 ng/mL, respectively; **Table 1**). There was no significant effect of the levels of NEB on VEBW, BFT, MY, the plasma concentrations of NEFA and glucose over the full period of observation (**Table 1**).

**Table 1:** Zootechnical and nutritional parameters of cows either with severe (SNEB) or moderate (MNEB) NEB at -4, 1 and 16 weeks peripartum and plasma metabolites concentration of cows either with SNEB or moderate MNEB NEB at -4, 1 and 14 weeks peripartum.

Group	SNEB			MNEB			P-value		
Week peripartum	-4	1	16	-4	1	16	Group	Week	Group*Week
Dry matter intake (DMI, kg/day)		9.36 ± 0.64	16.52 ± 0.69		13.08 ± 0.66	18.51 ± 0.62	**<0.0001**	**<0.0001**	0.15
Energy balance (EB, Mcal/d)		-12.10 ± 0.55	-1.51 ± 0.83		-5.23 ± 0.76	-0.95 ± 1.04	**<0.0001**	**<0.0001**	**0.0002**
Live body weight (LBW, kg/day)	629.05 ± 13.67	539.28 ± 13.52	532.57 ± 12.41	647.59 ± 9.52	558.26 ± 13.14	567.14 ± 12.37	**0.02**	**<0.0001**	0.78
Variation of empty body weight (VEBW, kg/day)		-14.23 ± 1.22	-15.04 ± 1.80		-13.85 ± 1.33	-12.46 ± 1.21	0.33	**<0.0001**	0.53
Milk yield (MY, kg/day)		23.39 ± 0.95	24.22 ± 0.74		22.47 ± 1.37	26.79 ± 1.16	0.41	**0.02**	0.11
Back fat thickness (BFT, cm)	0.77 ± 0.09	0.60 ± 0.07	0.15 ± 0.01	0.62 ± 0.05	0.53 ± 0.04	0.18 ± 0.02	0.08	**<0.0001**	0.42
Week peripartum	-4	1	14	-4	1	14	Group	Week	Group*Week
Glucose (mmol/L)	3.97 ± 0.44	3.51 ± 0.23	4.42 ± 0.59	4.40 ± 0.59	4.08 ± 0.36	4.30 ± 0.52	0.43	0.33	0.85
NEFA (mmol/L)	0.47 ± 0.04	1.99 ± 0.21	0.67 ± 0.08	0.36 ± 0.04	1.46 ± 0.19	0.57 ± 0.06	0.57	**<0.0001**	0.89
Insulin (ng/mL)	0.64 ± 0.09	0.43 ± 0.09	0.55 ± 0.08	0.79 ± 0.10	0.40 ± 0.08	0.53 ± 0.08	**0.02**	**0.01**	0.61

### Effect of different levels of negative energy balance on reproductive parameters

We observed that the commencement of luteal activity occurred later in SNEB than in MNEB animals (37 ± 5 vs. 25 ± 2 days; *P* = 0.04; **Table 2**). The length of the oestrus cycle was longer in SNEB than in MNEB cows (39 ± 5 vs. 23 ± 2 days; *P* = 0.007, respectively; **Table 2**). The numbers of follicular waves were not different between SNEB and MNEB cows, as well as the number of SF (63 ± 16 vs. 35 ± 8; *P* = 0.15), MF (11 ± 2 vs. 7 ± 1; *P* = 0.10), and LF (7 ± 1 vs. 5 ± 1; *P* = 0.11; **Table 2**). We also found that calving-first AI (79 ± 5 vs. 65 ± 3 days; *P* = 0.09), calving-calving intervals (409 ± 13 vs. 402 ± 10 days; *P* = 0.72) and conception rates at 35 (38 vs. 41%; *P* = 0.90) and 90 days (38 vs. 35%; *P* = 0.90) were not affected by the level of the NEB (**Table 2**).

**Table 2:** Reproductive parameters of cows either with severe (SNEB) or with moderate (MNEB) negative energy balance.

	SNEB	MNEB	P-value
Commencement of luteal activity (days)	37.38 ± 5.02	24.65 ± 2.44	**0.04**
Cycle length (days)	39.14 ± 4.67	23.41 ± 1.87	**0.007**
Follicular waves (0)^1^	14.28% (3/21)	5.88% (1/17)	0.44
Follicular waves (1)^1^	14.28% (3/21)	17.65% (3/17)	0.80
Follicular waves (2)^1^	4.76% (1/21)	29.41% (5/17)	0.08
Follicular waves (3)^1^	19.05% (4/21)	29.41% (5/17)	0.55
Follicular waves (≥4)^1^	47.62% (10/21)	17.65% (3/17)	0.16
Number of small follicles^2^	62.95 ± 15.85	35.18 ± 7.55	0.15
Number of medium follicles^2^	11.43 ± 2.21	6.77 ± 1.31	0.10
Number of large follicles^2^	7.24 ± 1.11	4.94 ± 0.74	0.11
Calving-first AI interval (days)	78.78 ± 4.97	65.20 ± 3.20	0.09
Calving-calving interval (days)	409.35 ± 13.09	402.03 ± 10.34	0.72
Success rate 35 days after AI	38.10% (8/21)	41.18% (7/17)	0.90
Success rate 90 days after AI	38.10% (8/21)	35.29% (6/17)	0.90

^1^”Follicular waves (0)” means no follicular wave, the number in parenthesis indicates the follicular wave number.

^2^During the cycle before artificial insemination (AI), the ovarian follicular dynamics of primiparous cows were monitored three times per week by transrectal ultrasonography allowing for the detection, counting and measurement of small (3–5 mm), medium (5 and ≤ 7 mm) and large follicles (> 7 mm) as described in materiels and methods. During this period, the mean number of follicles in each class per cow was calculated from the total number of follicles of a given class from both ovaries (SF, MF, or LF) divided by the number of ultrasonographic examinations.

### Differential mRNA expression of genes in the subcutaneous adipose tissue of SNEB and MNEB dairy cows identified by RNA sequencing

**Fig 1:**
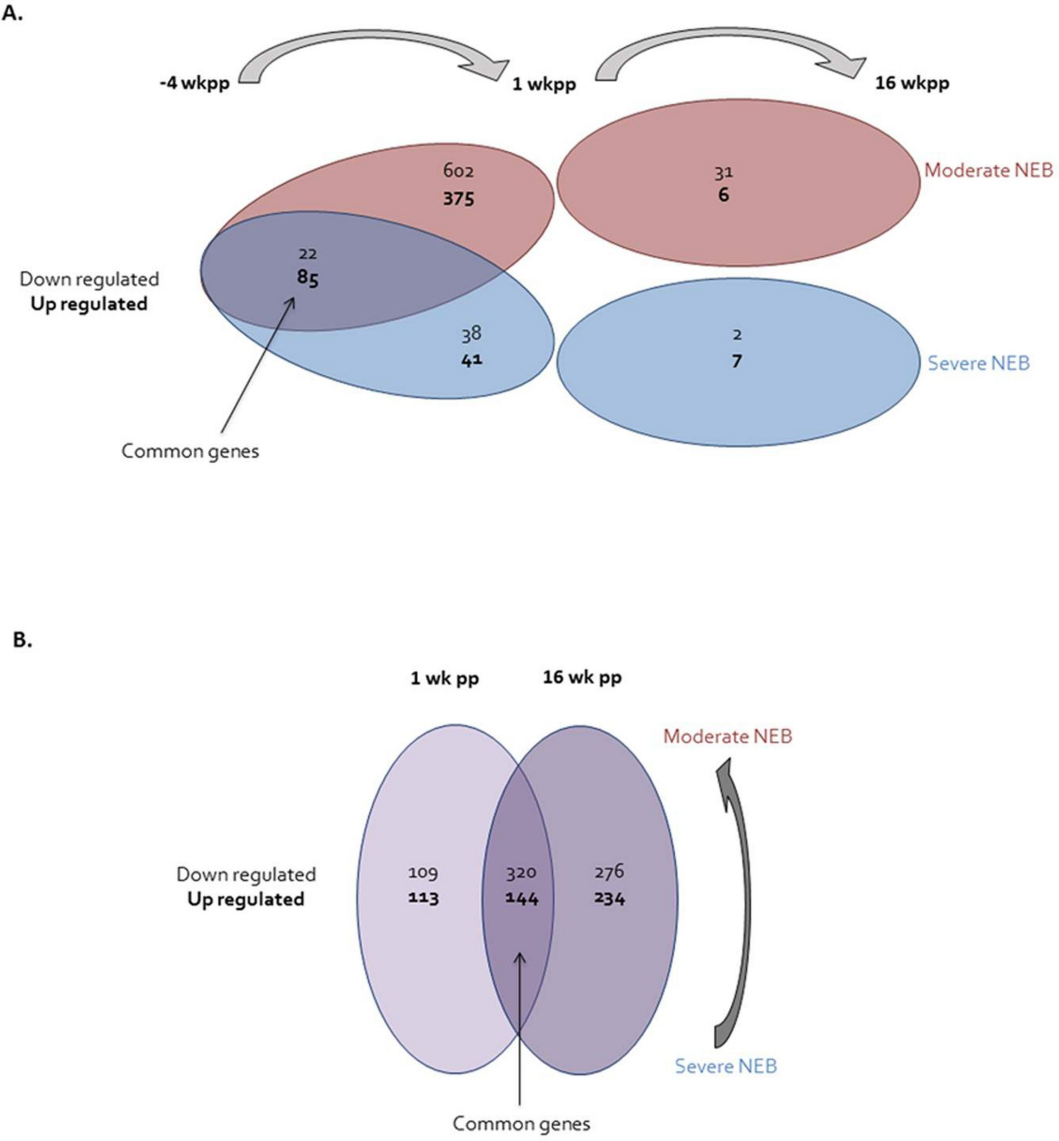
Venn diagram representation of gene differentially expressed according to the time of lactation (-4, 1 and 16 weeks peripartum) (A) or the status of the negative energy balance (moderate and severe negative energy balance) (B). wkpp: weeks peripartum; NEB: negative energy balance.

The expression of genes in subcutaneous adipose tissue at -4 wkpp relative to their expression at 1 wkpp, and at 1 wkpp relative to their expression at 16 wkpp, was evaluated separately in SNEB (n = 3) and MNEB (n = 3) animals (**Fig 1A**). In MNEB cows, 977 DEGs were found between -4 and 1 wkpp (**S4 Table**) and 37 DEGs between 1 wkpp and 16 wkpp (**S5 Table**). In SNEB cows, 79 DEGs were found between -4 and 1 wkpp (**S6 Table**) and 9 DEGs between 1 and 16 wkpp (**S7 Table**). In addition, 107 DEGs were common to MNEB and SNEB between -4 and 1 wkpp. When comparing the expression of genes in the adipose tissue of MNEB and SNEB cows at 1 or 16 wkpp (**Fig 1B**), 214 DEGs at 1 wkpp (**S8 Table**) and 510 DEGs at 16 wkpp (**S9 Table**) were found between the two groups of cows. Moreover, 464 DEGs were common in SNEB and MNEB cows at 1 and 16 wkpp.

### Identification of the main molecular and cellular functions by gene ontology and upstream regulation tools

**Fig 2:**
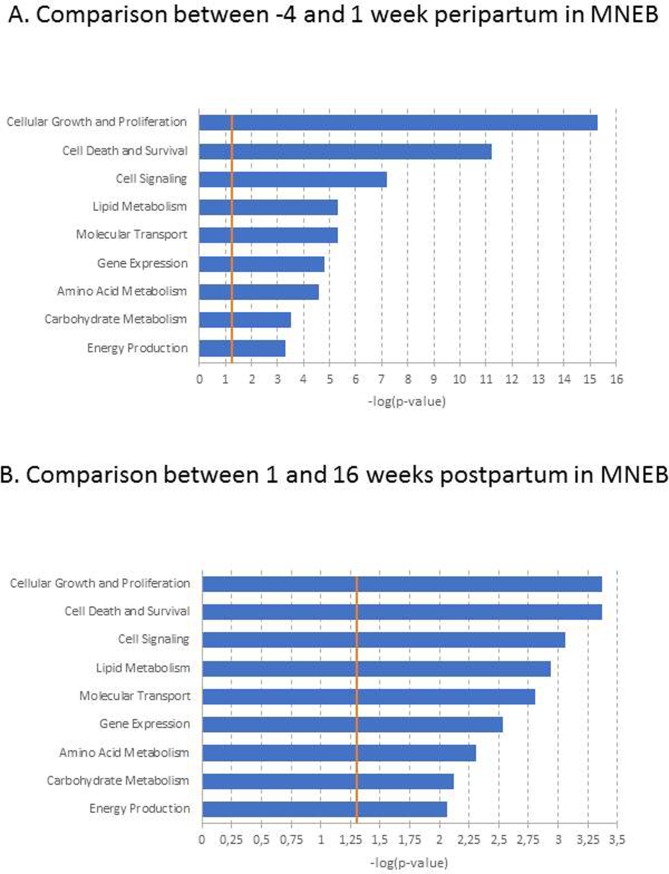
Molecular and cellular functions of differentially expressed genes between -4 and 1week peripartum (A) or 1 and 16 weeks postpartum (B) in adipose tissue of cows with moderate negative energy balance (MNEB). The bars indicate the likelihood [-log (p-value)] that the specific molecular or cellular function was affected by time compared with other functions represented by the list of differentially expressed genes. Threshold–log p-value (orange line) is set to 1.3, which equals a p-value of 0.05.

When analysing the results of the different comparisons (by time and group), all DEGs in adipose tissue were submitted to the Ingenuity Knowledge Base. The subcategories under the IPA category “Molecular and Cellular functions”, which reveal potentially the most prevalent biological changes found in adipose tissue, are reported. In MNEB cows, 143 DEGs between -4 and 1 wkpp and 13 DEGs between 1 and 16 wkpp were involved in metabolic functions (**Fig 2A and 2B**).

**Fig 3:**
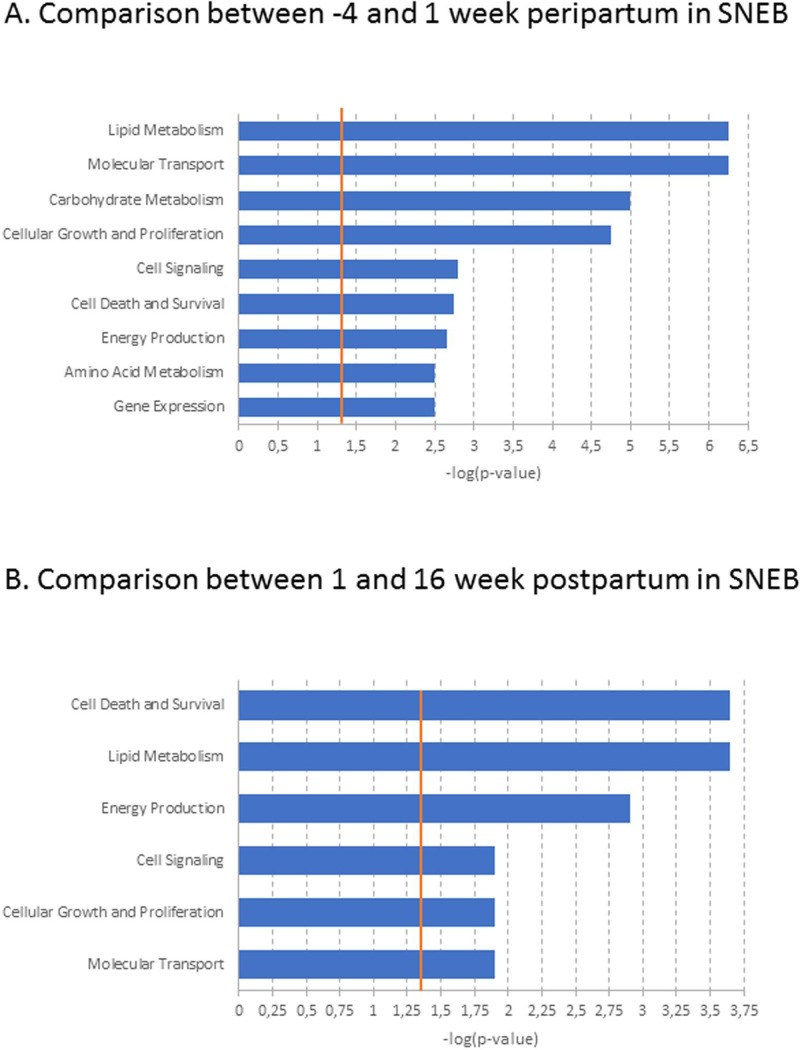
Molecular and cellular functions of differentially expressed genes between -4 and 1week peripartum (A) or 1 and 16 weeks postpartum (B) in adipose tissue of cows with severe negative energy balance (SNEB).The bars indicate the likelihood [-log (p-value)] that the specific molecular or cellular function was affected by time compared with other functions represented by the list of differentially expressed genes. Threshold–log p-value (orange line) is set to 1.3, which equals a p-value of 0.05.

Among the DEGs involved in metabolic functions, 90 genes were linked in a lipid metabolism network, including *SCD* (stearoyl-CoA desaturase (delta-9-desaturase)) and *BHLHE40* (basic helix-loop-helix family, member E40). Interestingly, *SCD* and *BHLHE40* were expressed more at -4 wkpp than at 1 wkpp (**S3 Fig**) and expressed less at 1 wkpp than at 16 wkpp (**S3 Fig**). In SNEB cows, 22 DEGs between -4 and 1 wkpp and 2 DEGs between 1 and 16 wkpp were involved in metabolic functions (**Fig 3A and 3B**).

**Fig 4:**
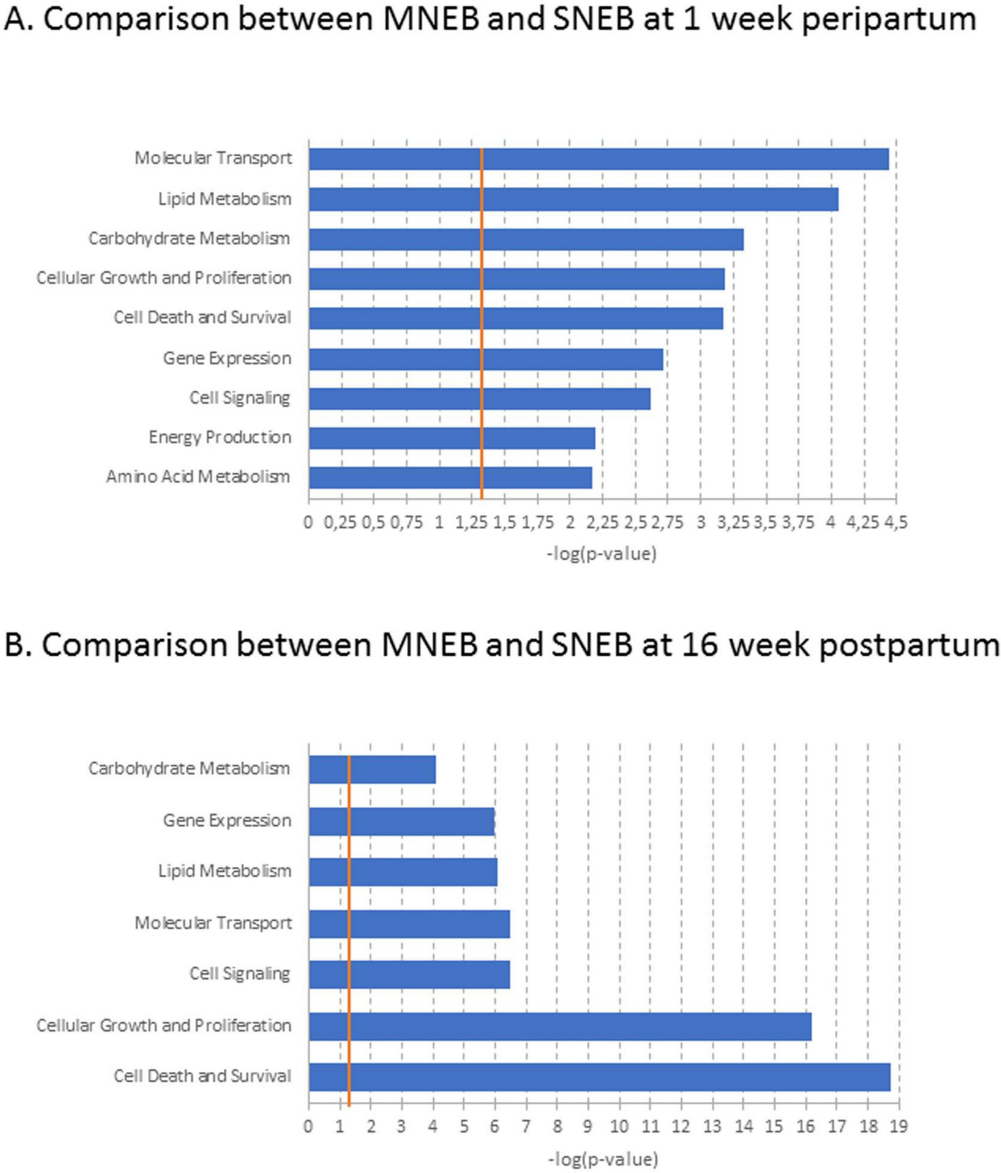
Molecular and cellular functions of differentially expressed genes between SNEB (severe negative energy balance) and MNEB (moderate negative energy balance) cows at 1 (A) and 16 (B) week postpartum.The bars indicate the likelihood [-log (p-value)] that the specific molecular or cellular function was affected by time compared with other functions represented by the list of differentially expressed genes. Threshold–log p-value (orange line) is set to 1.3, which equals a p-value of 0.05.

A network related to lipid metabolism function, which linked 8 genes that were expressed less and 3 genes that were expressed more at -4 wkpp than at 1 wkpp, was also observed (**S4 Fig**). However, in SNEB cows, between 1 and 16 wkpp, no network was reported by the IPA analysis of DEGs. Finally, 37 DEGs between SNEB and MNEB cows at 1 wkpp and 81 DEGs between SNEB and MNEB cows at 16 wkpp were involved in metabolic functions (**Fig 4**).

Some of the DEGs between SNEB and MNEB cows that belong to the regulation of lipid metabolism are represented in S6 Table. At 1 wkpp, the network included 13 highly expressed genes and 6 less expressed genes in SNEB cows relative to MNEB cows (**S5 Fig**). At 16 wkpp, the network included 10 over-expressed genes and 9 under-expressed genes in SNEB cows compared to MNEB cows (**S5 Fig).**

### Identification of gene clusters by the RT-qPCR validation of DEGs

**Fig 5:**
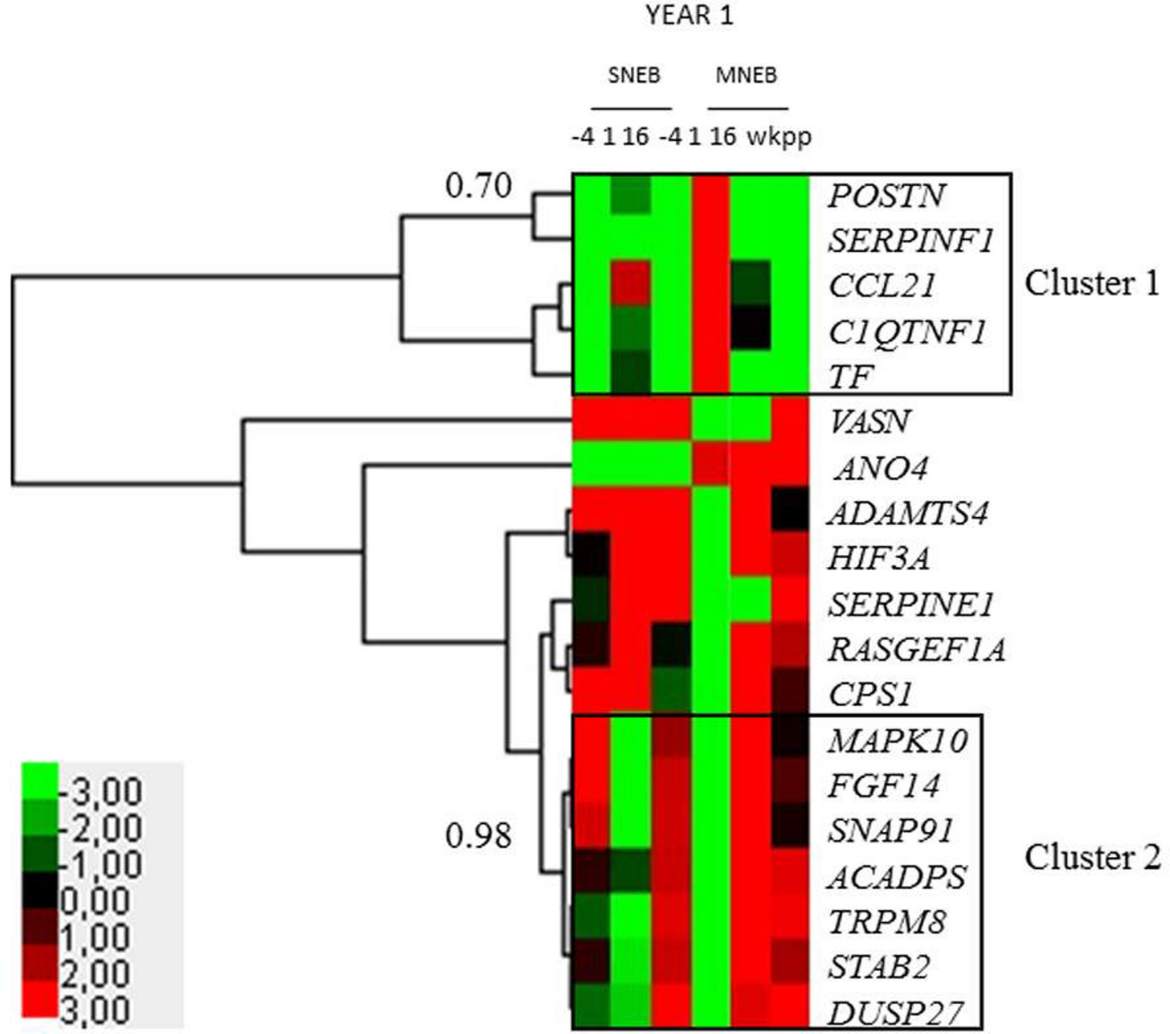
Clustering of candidate genes in adipose tissue of cows either with severe (SNEB) or moderate (MNEB) negative energy balance. Quantitative RT-PCR analysis was performed on subcutaneous adipose tissue samples collected at -4, 1 and 16 wkpp (n = 7 SNEB, n = 7 MNEB). Clustering of genes was performed using Cluster 3.0 software. Two clusters stand out, and node correlation thresholds are indicated for each cluster.

To further validate the difference in gene expression in adipose tissue during lactation in SNEB and MNEB cows, we measured the expression of 19 genes from the RNA sequencing analysis that were selected based on their fold change (range between -4 and -1.63 for under-expressed genes and between 1.82 and 5.94 for over-expressed genes), in adipose tissue at -4, 1 and 16 wkpp during the first lactation. A Euclidian distance/average linkage analysis, performed with Cluster 3.0 software, enabled us to find two different clusters exhibiting a high threshold (≥ 0.7; **Fig 5**). Cluster 1 was composed of five genes: *POSTN* (periostin), *SERPINF1* (serpin family F member 1), *CCL21* (C-C motif chemokine ligand 21), *C1QTNF1* (C1q and TNF related 1) and *TF* (transferrin). Cluster 2 was composed of seven genes: *MAPK10* (mitogen-activated protein kinase 10), *FGF14* (fibroblast growth factor 14), *SNAP91* (synaptosome associated protein 91), *ACADPS* (calcium dependent secretion activator), *TRPM8* (transient receptor potential cation channel subfamily M member 8), *STAB2* (stabilin 2) and *DUSP27* (dual specificity phosphatase 27, atypical). In cluster 1, all genes were over-expressed at 1 and 16 wkpp compared to at -4 wkpp in SNEB cows, whereas the opposite was observed in MNEB cows. In cluster 2, genes were over-expressed at 1 wkpp compared to at -4 and 16 wkpp in SNEB cows, whereas these genes were over-expressed at -4 wkpp compared to at 1 and 16 wkpp in MNEB cows (**Fig 5**).

### Identification of relevant biomarker candidates related to reproductive traits by using gene ontology and upstream regulations tools

All DEGs were also analysed using the biomarker filters tool of IPA. From this analysis, 264 biomarker candidates were reported from the different lists of DEGs, including 116 biomarker candidates between -4 and 1 wkpp and 10 additional biomarker candidates between 1 and 16 wkpp in MNEB cows (**S10 and S11 Tables**). In SNEB cows, 22 biomarker candidates were found between -4 and 1 wkpp, with 2 additional candidates between 1 and 16 wkpp (**S12 and S13 Tables**). From the list of DEGs issued from the comparison of MNEB and SNEB samples, 34 and 80 biomarker candidates were highlighted at 1 wkpp and 16 wkpp, respectively (**S14 and S15 Tables**).

Of these biomarker candidates, 177 were found to be expressed in the mammalian reproductive tract or related to reproductive functions, including *AMH* (anti-Mullerian hormone), *BMP7* (bone morphogenetic protein 7), *ESR1* (oestrogen receptor 1) and *SOX10* (SRY-box 10) (**S15 Table**). Moreover, some of these biomarker candidates, such as *IHIT5* (inter-alpha-trypsin inhibitor heavy chain family member 5), *SRFP2* (secreted frizzled related protein 2) and *CCL21* (C-C motif chemokine ligand 21), are known to be released in blood and are considered potentially novel adipokines (**S10 Table**).

### mRNA and protein expression of *CCL21* in the subcutaneous adipose tissue of SNEB and MNEB dairy cows

**Fig 6:**
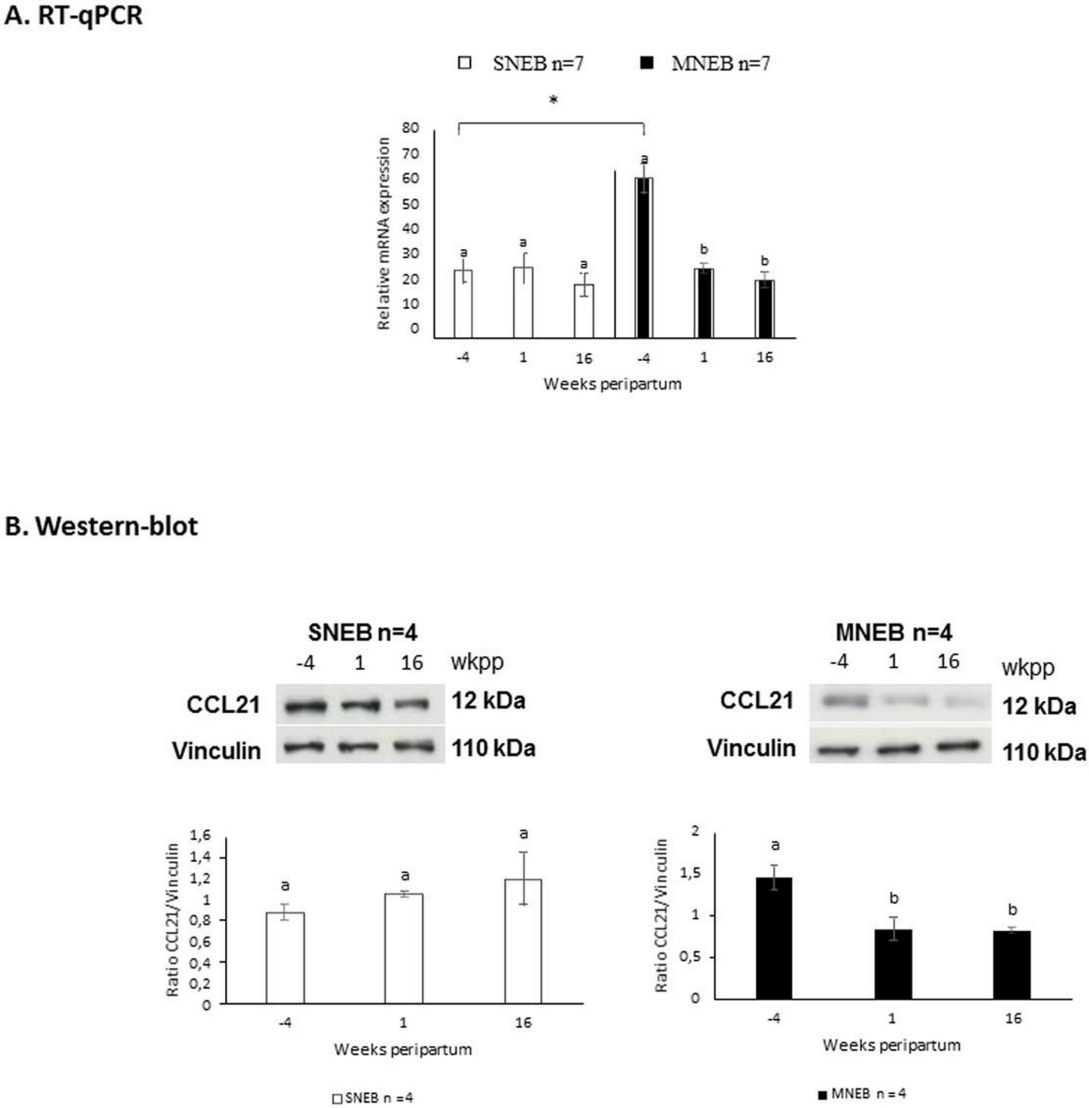
mRNA (A) and protein expression (B) level of *CCL21* in subcutaneous adipose tissue of cows either with severe (SNEB) or moderate (MNEB) negative energy balance. A. Quantitative RT-PCR analysis for *CCL21* was performed on subcutaneous adipose tissue samples collected at -4, 1 and 16 wkpp (n = 7 SNEB, n = 7 MNEB) as described in material and methods. B. Protein expression was determined by western blot using bovine anti-CCL21 antibody (12 kDa). Expression of vinculin (110 kDa) was used to normalize the expression of CCL21. Results are represented as LSM ± SEM. Low case letters represent significant effect of time separately in the group SNEB and MNEB (*P* < 0,05). * *P* < 0,05 (effect of group).

The mRNA and protein amount of *CCL21* was determined in the adipose tissue of SNEB and MNEB cows at -4, 1 and 16 wkpp. The MNEB cows had higher *CCL21* mRNA levels than the SNEB cows at -4 wkpp (**Fig 6A**). Immunoblotting protein extracts revealed the presence of CCL21 (12 kDa) in subcutaneous adipose tissues from all samples. As observed for the regulation of the *CCL21* mRNA expression pattern (**Fig 6A**), the CCL21 protein level decreased at 1 and 16 wkpp compared to at -4 wkpp in MNEB cows and a similar profile was seen in SNEB cows (**Fig 6B**). In our experimental conditions, we could not compare the CCL21 protein level between the two groups (MNEB and SNEB) since the samples were loaded on different membranes.

### Plasma CCL21 levels in SNEB and MNEB dairy cows

**Fig 7:**
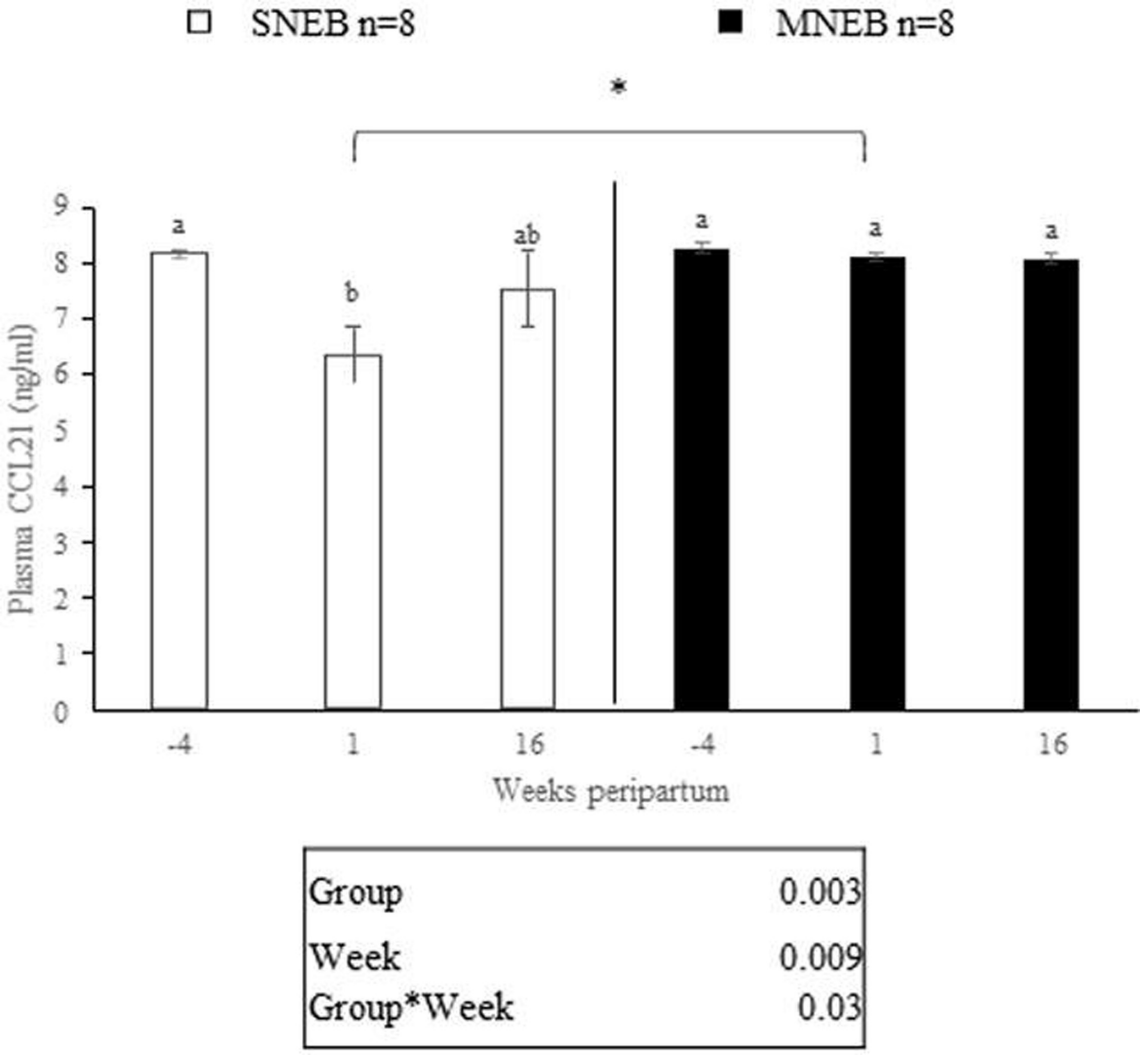
Concentration of CCL21 in plasma of cows either with severe (SNEB) or moderate (MNEB) negative energy balance. Plasma level of CCL21 was determined by ELISA assay after collecting blood at -4, 1, and 16 weeks peripartum (wkpp) of cows with SNEB (n = 8) or low NEB (n = 8). Results are represented as LSM ± SEM. Low case letters represent a significant (*P* < 0.05) time effect separately in the group SNEB and MNEB and * represents a significant (*P* < 0. 05) group effect.

Plasma CCL21 levels were measured at -4, 1 and 16 wkpp using ELISA assays. Consistent with the above expression and protein levels in fat tissue, we observed a decrease in the plasma concentrations of CCL21 at 1 wkpp in SNEB cows compared with at -4 wkpp (**Fig 7**). There was no variation in plasma CCL21 levels in MNEB cows. Moreover, we found that MNEB cows had higher plasma CCL21 levels than SNEB cows at 1 wkpp (**Fig 7**). Furthermore, we observed a negative correlation between the calving-first AI interval and the AUC (area under the curve) of plasma CCL21 concentrations (r = -0.98, *P* = 0.02, n = 8 for each group).

### mRNA expression of *CCL21* and its receptor *CCR7*, and protein localisation of CCL21 in bovine ovarian tissue

**Fig 8:**
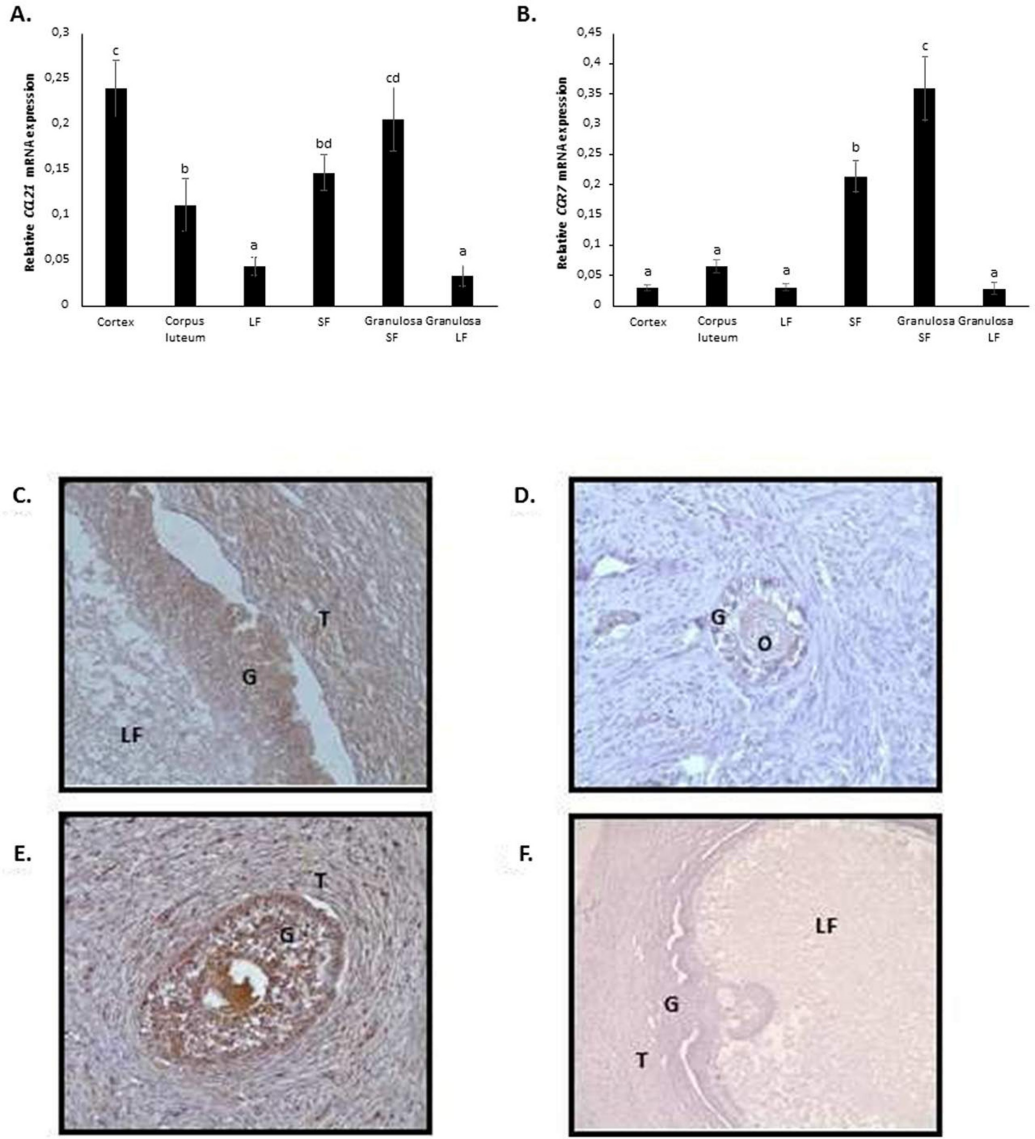
mRNA expression of *CCL21* and its receptor *CCR7* and protein localization of CCL21 in bovine ovary. The mRNA expression in ovarian cortex, corpus luteum, large follicles (LF), small follicles (SF), granulosa of large follicles and granulosa of small follicles was determined by RT-qPCR using specific bovine primers. The mRNA expression of *CCL21* (A) and *CCR7* (B) was normalized to geometric mean of three references genes (*UXT*, *SADH* and *PPIA*). Protein expression was determined by immunohistochemistry using anti-CCL21 antibody (A, B and C) and rabbit IgG (D). Immuno-specific staining is brown. The sections were counterstained with haematoxylin. Granulosa and theca cells from pre-ovulatory follicle (A), primary follicle (B), second follicle (C) and pre-ovulatory follicle (D). T: Theca cells; G; Granulosa cells; LF: Follicular fluid; O: oocyte.

The expression of *CCL21* and its receptor *CCR7*, and the localization of CCL21 were determined in different types of samples taken from bovine ovaries obtained from a slaughterhouse. The mRNA expression of *CCL21* was greater in cortex and granulosa cells from small follicles than in the corpus luteum, large follicles and granulosa cells from large follicles (**Fig 8A**). *CCR7* mRNA was more highly expressed in small follicles and granulosa cells from small follicles (**Fig 8B**) compared to other types of samples. Immunohistochemistry performed on bovine ovarian follicle sections showed that CCL21 was present in primary (**Fig 8C**) and secondary pre-ovulatory follicles (**Fig 8A–8E**). More precisely, CCL21 was detected in granulosa cells, theca cells (**Fig 8C–8E**) and oocytes (primary follicle) (**Fig 8D**).

### Effect of CCL21 on progesterone secretion and the proliferation of bovine granulosa cells

**Fig 9:**
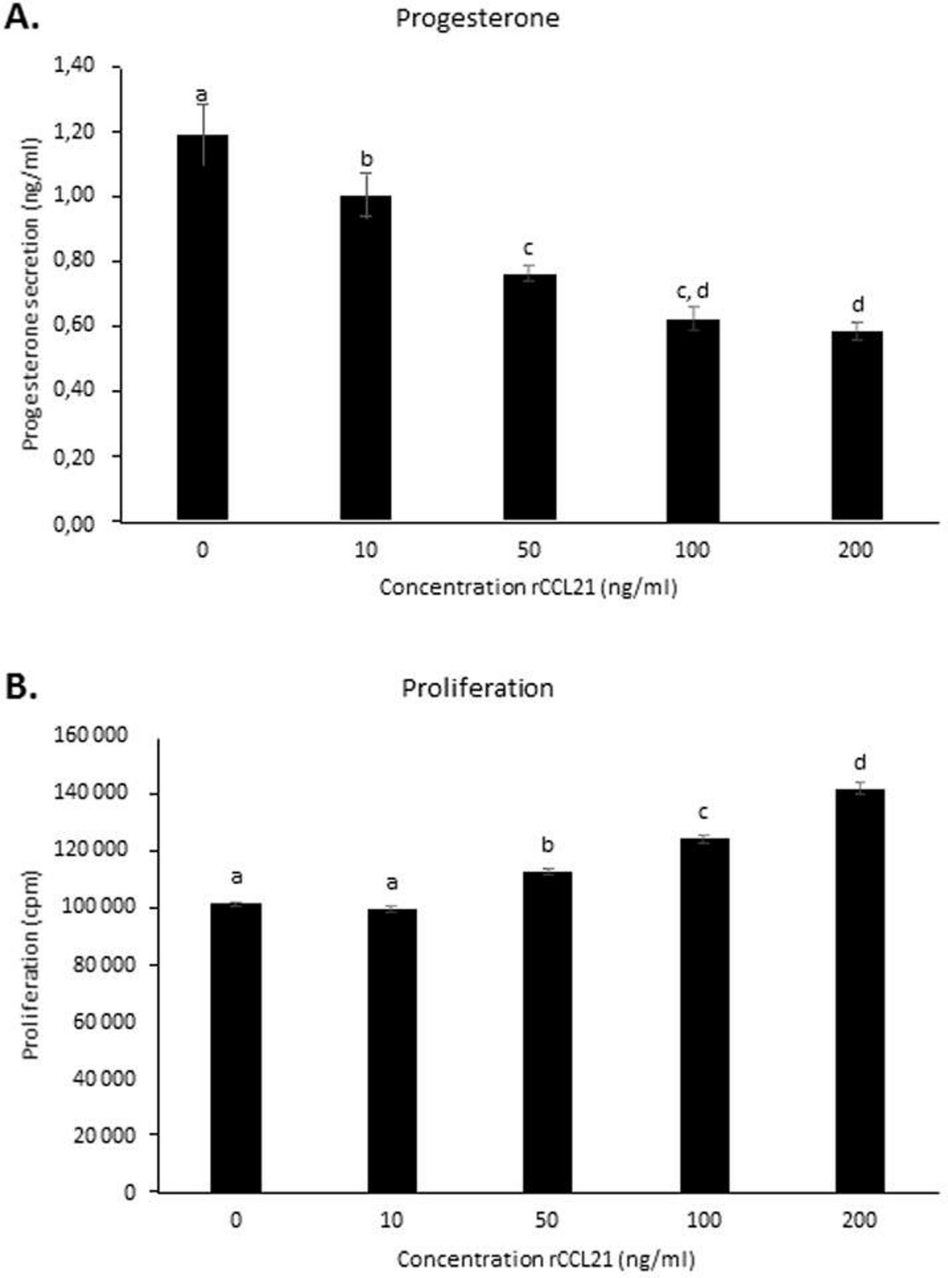
Effect of recombinant CCL21 on basal progesterone secretion (A) and proliferation by bovine primary granulosa cells (bGC) (B). Bovine granulosa cells (bGC) were cultured in medium with serum and then in serum-free medium in the absence or in the presence of recombinant CCL21 (10, 50, 100, 200 ng/ml) for 24 h or 48 h. The concentration of progesterone in culture medium was measured by ELISA assay. The proliferation of bGC cells was performed using ^3^H thymidine incorporation method. Results are represented as LSM ± SEM of three independent experiments. Different letters indicated significant effect with *P* < 0.05.

Cells were incubated in serum-free media containing various concentrations of recombinant CCL21 for 48 h; we observed that the secretion of progesterone decreased in a dose-dependent manner (*P* < 0.001) (**Fig 9A**). We also examined the effect of CCL21 on bovine granulosa cell proliferation in culture. Thymidine methyl- [3H] incorporation by primary bovine granulosa cells treated with different concentrations of CCL21 was assessed after 24 h of culture. Recombinant human CCL21 increased thymidine methyl-[3H] incorporation, leading to an increase of cell proliferation in a dose-dependent manner (*P* < 0.001) (**Fig 9B**).

## Discussion

Our results reveal that the level of NEB regulates metabolic and reproductive parameters associated with modifications of the transcriptome profile in the subcutaneous WAT of primiparous Holstein dairy cows at different time points of lactation (-4, 1 and 16 wkpp). The different techniques used to analyse gene expression showed a very strong correspondence between RNA-seq and RT-qPCR analysis for DEGs. By using *in silico* tools such as IPA, we documented that, in WAT, many of the DEGs between SNEB and MNEB cows are related to lipid metabolism networks; several of them are also known to be secreted and potentially involved in mammalian reproductive functions. A special focus on *CCL21* revealed that plasma CCL21 concentrations decreased significantly one week after calving in SNEB cows, whereas its profile was unaltered in MNEB cows. On the contrary, CCL21 (mRNA and protein) levels were down regulated between -4 and 1 wkpp in the subcutaneous adipose tissue of MNEB cows, whereas levels were unaffected in SNEB cows, suggesting that subcutaneous adipose tissue is not the only source of CCL21 in blood. Moreover, we showed that *CCL21* and its receptor *CCR7* were expressed in ovarian cells and that recombinant human CCL21 decreased *in vitro* progesterone secretion by granulosa cells, whereas it increased the proliferation of these cells, suggesting that CCL21 could regulate *in vivo* folliculogenesis.

### Negative energy levels, metabolic data and adipose tissue transcriptome

In high-producing dairy cows, the energy balance tends to be negative during the transition from late pregnancy to early lactation. NEB status mostly induces changes in body condition, adipose tissue deposits and the profile of circulating hormones. As described in our previous study [30], the severity of NEB after calving leads to a lower DMI, LBW and plasma insulin concentration. Currently, the effect of NEB on plasma insulin level appears after parturition [38, 39]. However, we found that the plasma concentration of insulin decreased from -4 to 16 wkpp and was lower in cows with SNEB at -4 wkpp. The transition period is also associated with the high mobilisation of adipose tissue, resulting in an increase of the circulating NEFA levels [30]. Paradoxically, from this group of cows, we did not find any significant effect of the level of NEB on plasma NEFA concentrations or BFT between SNEB and MNEB dairy cows due to the variability of the animals’ responses. However, we confirmed that energy status is related to adipose tissue adaptation by assessing its transcriptome profile at different times of lactation [14].

Previous studies demonstrated that changes in the activity level of enzymes controlling lipid metabolism occur in adipose tissue during lactation [40]. More recently, many studies indicate metabolic adaptations to lactation in adipose tissue [14, 41]. According to these studies, we showed that the most important affected function is lipid metabolism [13, 42, 43]. Interestingly, the highest number of DEGs was found during the transition period, when energy needs for milk production are critical; this makes sense given the huge physiological changes induced by the initiation of milk production. These results are in agreement with recent transcriptomic analyses of bovine adipose tissue performed during the transition from pregnancy to lactation. Sumner-Thomson et al. [14] demonstrated the importance of several specific genes, but did not provide the corresponding functional analysis. In addition, Janovick et al. [44] found around 3,000 genes that were both affected by stage of lactation and energy status; consistent with more recent studies and the results obtained here, they associated DEGs with several metabolic functions [44–46]. Herein, we found that energy status impacts the bovine white subcutaneous adipose tissue transcriptome regulating lipid metabolism. These results suggest that the modulation of transcriptome by the levels of NEB (either by milk production or by nutrition) in adipose tissue regulates lipid metabolism, but is not sufficient to affect fat accumulation. However, the fact that some lipid metabolism-related genes have different expression profiles depending on the localisation and the type of adipose tissue (e.g. visceral adipose tissue [omental, mesenteric, and retroperitoneal] vs. subcutaneous adipose tissue [tail head, withers, and sternum]) should be taken into account [47, 48].

### Negative energy levels, reproductive data and adipose tissue transcriptome

As already described by numerous groups, the severity of NEB is responsible for reproductive disorders in dairy cows [49–51]. Considering the results obtained in a previous study and the choice of extreme groups of NEB, a greater effect on reproductive performance was expected [30]. Hence, we confirm here from a more pronounced difference between the groups that severe NEB delays the commencement of luteal activity by 12.73 days [30]. These results are in agreement with those obtained by von Leesen et al. [52], who showed that cows with a higher genetic merit for energy balance early in lactation express an earlier commencement of luteal activity. In addition, we found that the cycle length was longer in cows with SNEB compared to cows with MNEB. However, other reproductive traits already described to be affected by NEB, such as follicular waves and fertility rate, were not affected by the level of NEB [53]. This could be due to the small sample size. Former studies reporting the effect of NEB on the transcriptomic analysis of reproductive tissues did not provide a functional relationship with energy metabolism [54, 55]. For the first time, here, we link the effects of the level of NEB on adipose tissue metabolism to its effects on reproductive parameters related to ovarian activity. In order to achieve our aim, we highlighted several potential biomarkers by combining bioinformatics analysis and published bibliography references. Of the DEGs, 264 were found to be expressed or have an effect on reproduction. From this list, we selected *CCL21* to validate its potential expression and effects on the bovine ovary.

### CCL21 as a novel bioactive adipokine

Cytokines are a family of secreted proteins originally known to be mainly involved in immunoregulatory and inflammatory processes [56]. In female reproduction, ovulation, menstruation, embryo implantation and delivery are considered inflammatory events [57] Hence, there is growing evidence showing the role of cytokines on reproductive function. Cytokines may affect the neuroendocrine events of reproduction, ovarian/testis function, the endometrium, the developing embryo, the placenta and parturition [58]. Moreover, chemokines participate in limiting bacterial infection and are involved in inflammatory processes in the reproductive tract [59]. Tríbulo et al. [60] recently showed that *CCL21* is highly expressed in the bovine oviduct and endometrium during the first day of oestrus, proposing that it was an embryokine. Due to the recent notion that adipose tissue plays a more important role than simply energy storage, and that cytokines may function in an endocrine manner, we hypothesised that *CCL21* may also be considered an adipokine. Indeed, *CCL21* and its receptor (*CCR7*) are expressed in adipose tissue at -4, 1 and 16 wkpp in SNEB and MNEB cows. The transcript expression of *CCL21* is up-regulated in the adipose tissue of MNEB cows during a period of high-energy mobilisation (1 wkpp). However, there was no significant difference in *CCR7* levels. Despite the fact that the accumulation of mRNA is not always indicative of protein synthesis, we observed a good concordance between the regulation of mRNA and the protein expression of *CCL21* in adipose tissue.

This study also shows that CCL21 is an adipokine, based on the measurements from blood with a specific bovine ELISA kit. For the first time, we characterised the circulating profile of CCL21 from late pregnancy to mid lactation under different levels of NEB. Conversely, for CCL21 expression in adipose tissue, we found that the plasma CCL21 concentrations were significantly lower in cows with SNEB compared to cows with MNEB at 1 wkpp. As the origin of circulating CCL21 was not determined in our study, we can not affirm that the released CCL21 was derived from adipose tissue. However, there was a significant negative correlation between the AUC of plasma CCL21 concentration and the calving-first AI interval; this result suggests that CCL21 may be involved in the regulation of ovarian function.

Previous studies indicated that cytokines mediate steroid synthesis in bovine granulosa cells [61]. Interestingly, we showed that *CCL21* and its receptor *CCR7* were highly expressed in the granulosa cells of SF; our lab obtained similar results for adiponectin and chemerin [22, 24]. Consistent with the effect of these adipokines, CCL21 decreased progesterone production by cultured granulosa cells at a lower dose (10 ng/mL); it also increased granulosa cell proliferation. It could be speculated that the regulation of steroidogenesis in the granulosa cells of small follicles by CCL21 may slow down follicle development and consequently prolong the calving-AI interval. However, in the context of our study, it was not possible to test the effect of the level of NEB on *CCL21* ovarian expression.

## Conclusion

In conclusion, changes in the subcutaneous adipose transcriptome caused by severe NEB in dairy cows induce changes in the expression profile of genes involved in lipid metabolism. This suggests that these genes are likely to take part in the control mechanism for adipose tissue adaptation during early lactation. Moreover, this study revealed potential links between the genes involved in adipose tissue metabolism and reproductive function. These first results may be important to identify potential biomarkers of sub or infertility associated with metabolic disorders. With this perspective, the identification of a potential novel secreted adipokine, CCL21, which could be involved in the regulation of ovarian function, appears to be of critical interest. This work paves the way for further studies, which are required to unravel the physiology, pathophysiology and endocrine mechanisms underlying the potential effects of newly-identified bioactive molecules for reproduction-related problems in dairy cows.

## Supporting information

S1 Fig Description of the timing for blood samples, ovarian and fat ultrasound, adipose tissue biopsy and fertility measurements for the peripartum period during first lactation of primiparous Hostein dairy cows fed with either a high-energy (HE) or a low energy (LE) diet. W = week peripartum, Gd = gestational day, A: first AI, US: ultrasound.
(TIF)

S2 Fig Experimental design of adipose tissue sampling and time of comparison by RNA sequencing and RT-PCR. For RT-PCR: n = 7 (SNEB) and n = 7 (MNEB). For RNA sequencing: n = 3 (SNEB) and n = 3 (MNEB). wkpp: weeks peripartum
(TIF)

S3 Fig Genes differentially expressed involved in lipid metabolism between -4 and 1 weeks peripartum (A) or 1 and 16 weeks postpartum (B) of cows with moderate negative energy balance (MNEB). Functional gene interaction networks were identified by Ingenuity Pathway Analysis (IPA). (A) Genes are colored based on fold-change values determined by RNA-Seq analysis, where the red-color symbols signify higher expression at -4 wkpp and green-color gene symbols indicate higher expression at 1 wkpp. (B) Genes are colored based on fold-change values determined by RNA-Seq analysis, where the red-color symbols signify higher expression at 1 wkpp and green-color gene symbols indicate higher expression at 16 wkpp. Each gene was assigned a shape and function by IPA as shown in the “Network Shapes” legend inset.
(TIF)

S4 Fig Genes differentially expressed involved in lipid metabolism between -4 and 1 weeks peripartum of cows with severe negative energy balance (SNEB). Functional gene interaction networks were identified by Ingenuity Pathway Analysis (IPA). Genes are colored based on fold-change values determined by RNA-Seq analysis, where the red-color symbols signify higher expression at -4 wkpp and green-color gene symbols indicate higher expression at 1 wkpp. Each gene was assigned a shape and function by IPA as shown in the “Network Shapes” legend inset.
(TIF)

S5 Fig Genes differentially expressed involved in lipid metabolism between severe (SNEB) and moderate (MNEB) negative energy balance at 1 (A) and 16 (B) week postpartum. Functional gene interaction networks were identified by Ingenuity Pathway Analysis (IPA). Genes are colored based on fold-change values determined by RNA-Seq analysis, where the red-color symbols signify higher expression in SNEB cows and green-color gene symbols indicate higher expression in MNEB cows. Each gene was assigned a shape and function by IPA as shown in the “Network Shapes” legend insert.
(TIF)

S1 Table A. Composition of the high energy (HE) and low-energy (LE) diets (% of DM) and B. Chemical composition and nutritional value of feeds (g/kg of DM unless otherwise noted).
(DOC)

S2 Table Summary of the average statistics of the sequence quality and alignment information for the high and low NEB cows. (DOCX)

S3 Table List of primers (Bos Taurus) used for RT-qPCR. (DOCX)

S4 Table Genes differentially expressed at 1 WKPP as compared to -4 WKPP in MNEB animals. (DOCX)

S5 Table Genes differentially expressed in MNEB animals at 16 WKPP as compared to 1WKPP. (DOCX)

S6 Table Genes differentially expressed in SNEB animals at 1 WKPP as compared to -4WKPP. (DOCX)

S7 Table Genes differentially expressed at 16 WKPP as compared to 1 WKPP in SNEB animals. (DOCX)

S8 Table Genes differentially expressed in SNEB and MNEB animals at 1 WKPP. (DOCX)

S9 Table Genes differentially expressed between MNEB and SNEB animals at 16 WKPP. (DOCX)

S10 Table List of differential expressed genes between -4 and 1week peripartum in adipose tissue of cows with MNEB (moderate negative energy balance) highlighted as biomarkers with IPA and their links with reproductive parameters. (DOCX)

S11 Table List of differential expressed genes between 1 and 16 weeks peripartum in adipose tissue of cows with MNEB (moderate negative energy balance) highlighted as biomarkers with IPA and their links with reproductive parameters. (DOCX)

S12 Table List of differential expressed genes between -4 and 1 week peripartum in adipose tissue of cows with SNEB (severe negative energy balance) highlighted as biomarkers with IPA and their links with reproductive parameters. (DOCX)

S13 Table List of differential expressed genes between 1 and 16 weeks peripartum in adipose tissue of cows with SNEB (severe negative energy balance) highlighted as biomarkers with IPA and their links with reproductive parameters. (DOCX)

S14 Table List of differential expressed genes in adipose tissue between cows with SNEB (severe negative energy balance) and cows with MNEB (moderate negative energy balance) at 1 week peripartum, highlighted as biomarkers with IPA and their links with reproductive parameters. (DOCX)

S15 Table List of differential expressed genes in adipose tissue between cows with SNEB (severe negative energy balance) and cows with MNEB (moderate negative energy balance) at 16 weeks peripartum, highlighted as biomarkers with IPA and their links with reproductive parameters. (DOCX)
