# Supplementary figures and images for: Impact of the severity of negative energy balance on gene expression in the subcutaneous adipose tissue of periparturient primiparous Holstein dairy cows: Identification of potential novel metabolic signals for the reproductive system
Source: PLoS One. 2019 Sep 26;14(9):e0222954. doi: 10.1371/journal.pone.0222954 (PMC6763198; doi:10.1371/journal.pone.0222954)

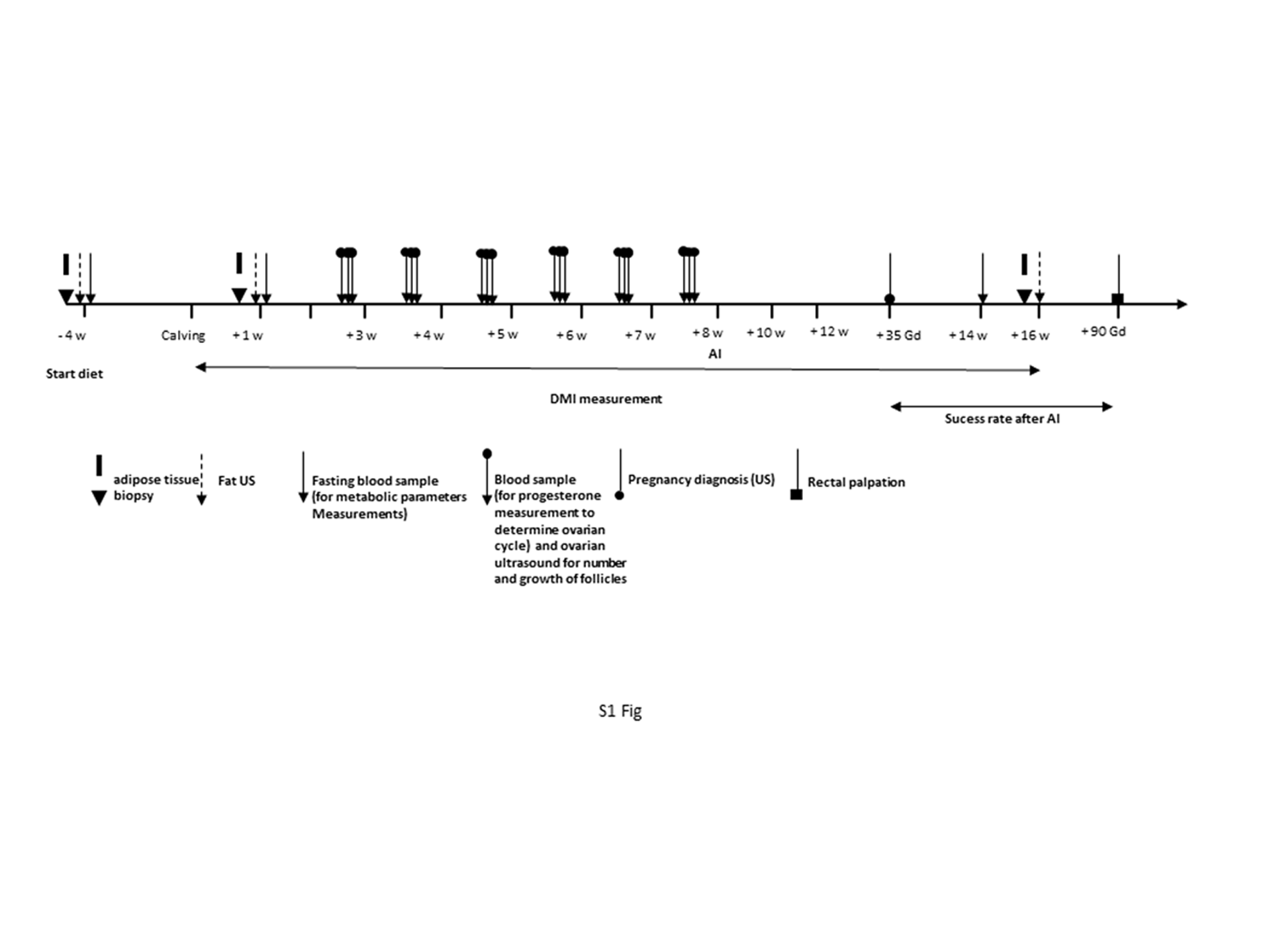

Supplement: S1 Fig — W = week peripartum, Gd = gestational day, A: first AI, US: ultrasound. (TIF) [file pone.0222954.s001.tif]

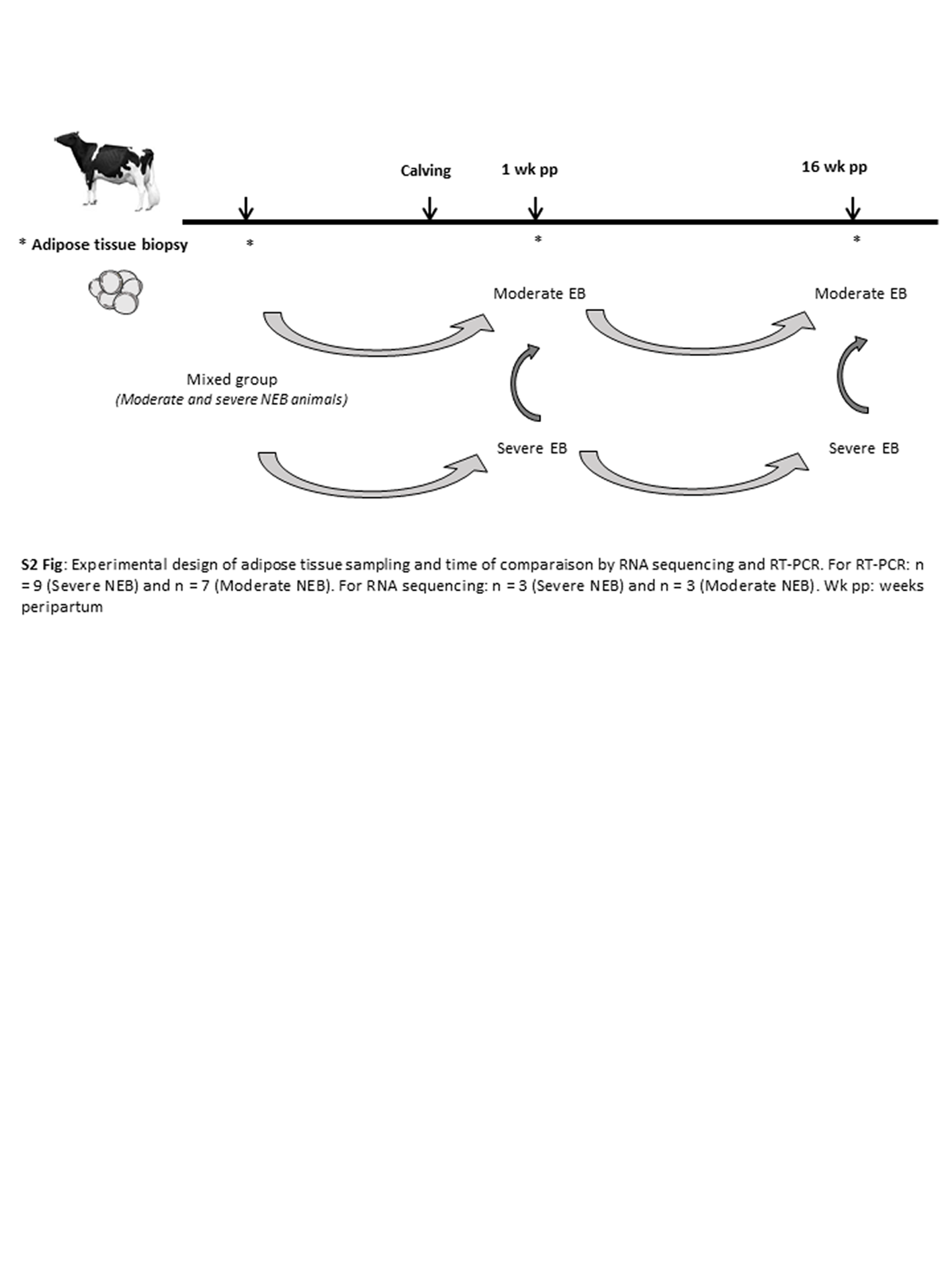

Supplement: S2 Fig — For RT-PCR: n = 7 (SNEB) and n = 7 (MNEB). For RNA sequencing: n = 3 (SNEB) and n = 3 (MNEB). wkpp: weeks peripartum (TIF) [file pone.0222954.s002.tif]

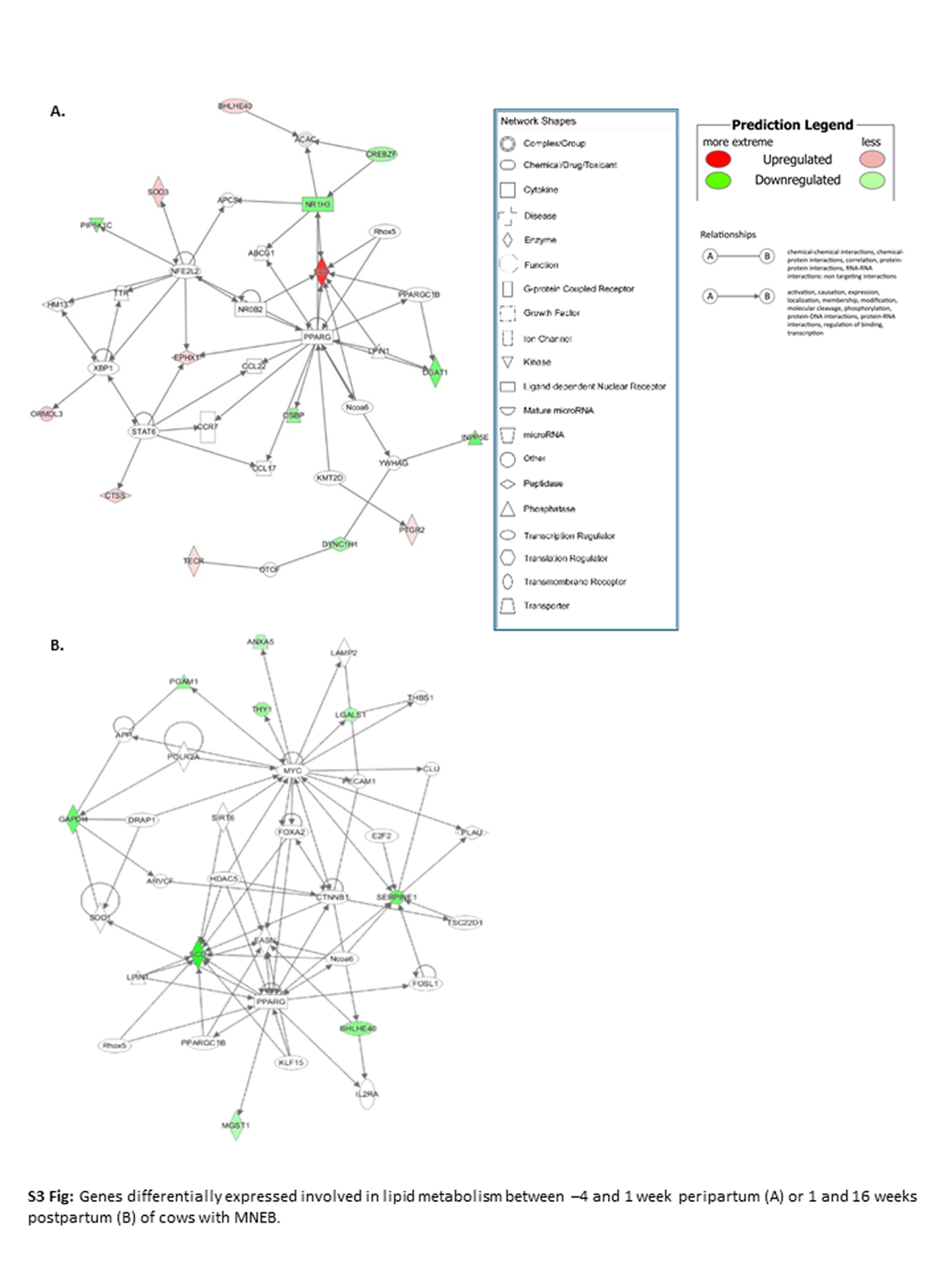

Supplement: S3 Fig — Genes differentially expressed involved in lipid metabolism between -4 and 1 weeks peripartum (A) or 1 and 16 weeks postpartum (B) of cows with moderate negative energy balance (MNEB). Functional gene interaction networks were identified by Ingenuity Pathway Analysis (IPA). (A) Genes are colored based on fold-change values determined by RNA-Seq analysis, where the red-color symbols signify higher expression at -4 wkpp and green-color gene symbols indicate higher expression at 1 wkpp. (B) Genes are colored based on fold-change values determined by RNA-Seq analysis, where the red-color symbols signify higher expression at 1 wkpp and green-color gene symbols indicate higher expression at 16 wkpp. Each gene was assigned a shape and function by IPA as shown in the “Network Shapes” legend inset. (TIF) [file pone.0222954.s003.tif]

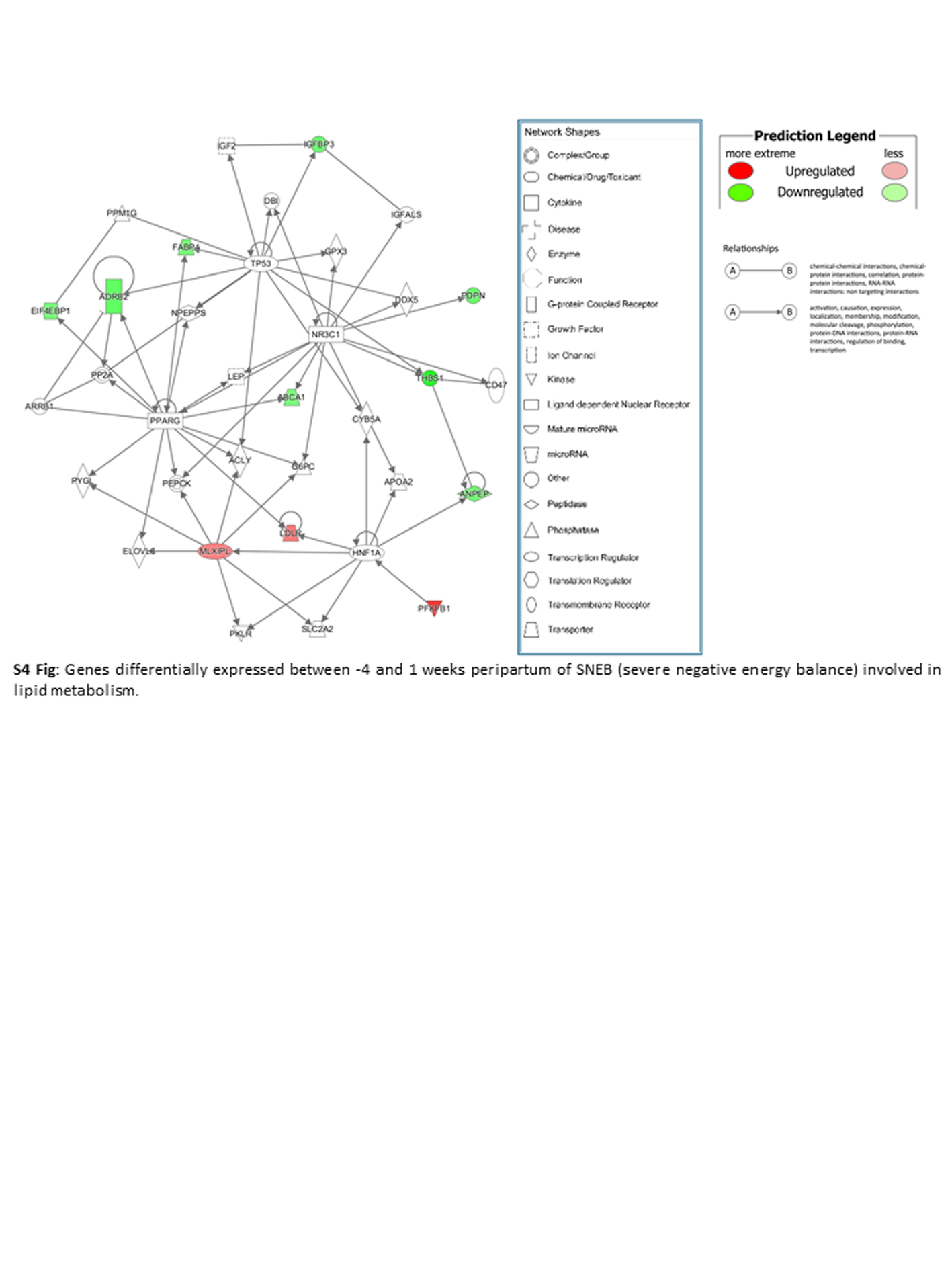

Supplement: S4 Fig — Functional gene interaction networks were identified by Ingenuity Pathway Analysis (IPA). Genes are colored based on fold-change values determined by RNA-Seq analysis, where the red-color symbols signify higher expression at -4 wkpp and green-color gene symbols indicate higher expression at 1 wkpp. Each gene was assigned a shape and function by IPA as shown in the “Network Shapes” legend inset. (TIF) [file pone.0222954.s004.tif]

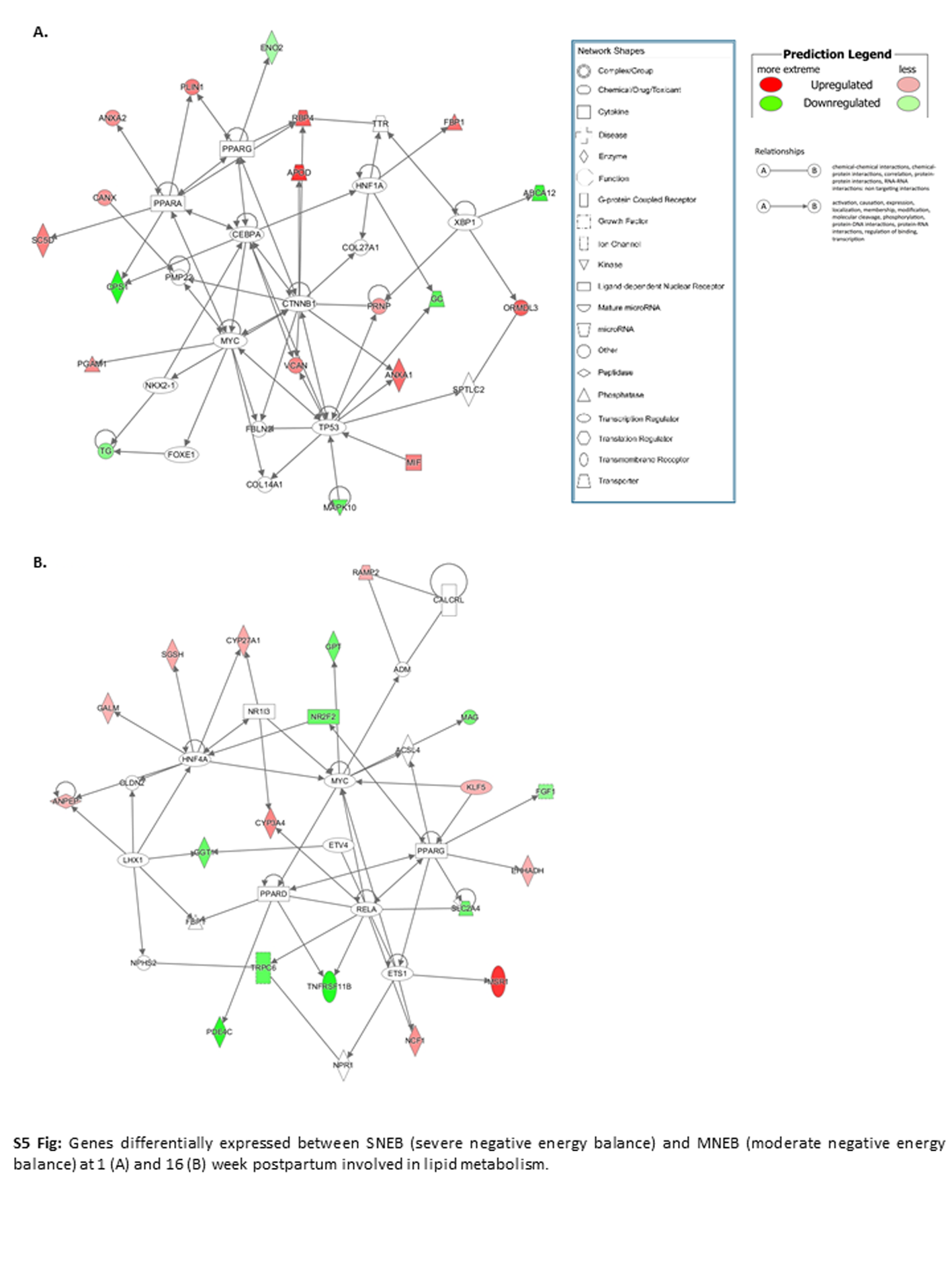

Supplement: S5 Fig — Genes differentially expressed involved in lipid metabolism between severe (SNEB) and moderate (MNEB) negative energy balance at 1 (A) and 16 (B) week postpartum. Functional gene interaction networks were identified by Ingenuity Pathway Analysis (IPA). Genes are colored based on fold-change values determined by RNA-Seq analysis, where the red-color symbols signify higher expression in SNEB cows and green-color gene symbols indicate higher expression in MNEB cows. Each gene was assigned a shape and function by IPA as shown in the “Network Shapes” legend insert. (TIF) [file pone.0222954.s005.tif]
