# Supplementary material for: Impact of the severity of negative energy balance on gene expression in the subcutaneous adipose tissue of periparturient primiparous Holstein dairy cows: Identification of potential novel metabolic signals for the reproductive system
Source: PLoS One. 2019 Sep 26;14(9):e0222954. doi: 10.1371/journal.pone.0222954 (PMC6763198; doi:10.1371/journal.pone.0222954)
Supplement: S1 Table — A. Composition of the high energy (HE) and low-energy (LE) diets (% of DM) and B. Chemical composition and nutritional value of feeds (g/kg of DM unless otherwise noted). (DOC) [file pone.0222954.s006.doc]

**A.**

| **Feed (% DM)** | **HE diet** | **LE diet** |
| --- | --- | --- |
| **Corn silage** | 61.2 | 50.4 |
| **Lucerne Hay** | 10.1 | 10.2 |
| **Grass silage** | 0 | 24 |
| **Energy Concentrate** | 17.2 | 8.5 |
| **Protein Concentrate** | 10.7 | 6.0 |
| **Ca Carbonate** | 0.5 | 0.4 |
| **Minerals CMV5/23** | 0.3 | 0.5 |

**B.**

|  | **Corn silage** | **Grass silage** | **Lucerne hay** | **Energy Concentrate** | **Protein Concentrate** | **Ca Carbonate** | **Minerals** |
| --- | --- | --- | --- | --- | --- | --- | --- |
| **DM, %** | 31.6 | 45.2 | 86.8 | 88 | 90 |  |  |
| **CP, g/kg DM** | 71.6 | 137.3 | 131.3 | 19 | 53 |  |  |
| **Cellulose, g/kg DM** | 201.9 | 299.5 | 380.8 | 5 | 7 |  |  |
| **Starch, g/kg DM** | 292.3 |  |  | 327 | 55 |  |  |
| **Energy, Mcal/kg DM** | 1.56 | 1.38 | 1.03 | 1.83 | 1.80 |  |  |
| **PDIN^1^, g/kg DM** | 44.0 | 84.5 | 85.7 | 136 | 366 |  |  |
| **PDIE^2^, g/kg DM** | 66.7 | 70.7 | 79.8 | 136 | 244 |  |  |
| **P, g/kg DM** | 1.9 |  |  | 3.7 | 6.1 |  | 70 |
| **Ca, g/kg DM** | 2.0 |  |  | 2.5 | 2.3 | 350 | 220 |

^1^PDIN = protein digested in the small intestine supplied by rumen-undegraded dietary protein and by microbial protein from rumen-degraded dietary nitrogen (“Protéines Digestibles dans l’Intestin permises par l’azote” in French) (INRA, 2007).

^2^PDIE = protein digested in the small intestine supplied by rumen-undegraded dietary protein and by microbial protein from rumen-fermented organic matter (“Protéines Digestibles dans l’Intestin permises par l’Energie” in French) (INRA, 2007)
