# Supplementary material for: Impact of the severity of negative energy balance on gene expression in the subcutaneous adipose tissue of periparturient primiparous Holstein dairy cows: Identification of potential novel metabolic signals for the reproductive system
Source: PLoS One. 2019 Sep 26;14(9):e0222954. doi: 10.1371/journal.pone.0222954 (PMC6763198; doi:10.1371/journal.pone.0222954)
Supplement: S2 Table — (DOCX) [file pone.0222954.s007.docx]

**S2 Table**: Summary of the average statistics of the sequence quality and alignment information for the high and low NEB cows

| Samples | Time | Energy state | Total reads (PF) | Post-Trim reads | Unique maps | Multiple maps | %Align (PT) |
| --- | --- | --- | --- | --- | --- | --- | --- |
|  |  |  | (x10^6^) | (x10^6^) | (x10^6^) | (x10^6^) |  |
| No.1 | -4 | Nd | 74.41 | 74.40 | 65.08 | 6.74 | 87.5 |
|  |  |  |  |  |  |  |  |
| No.2 | -4 | Nd | 67.21 | 67.21 | 54.81 | 9.62 | 81.6 |
|  |  |  |  |  |  |  |  |
| No.3 | -4 | Nd | 70.96 | 70.96 | 59.66 | 8.19 | 84.1 |
|  |  |  |  |  |  |  |  |
| No.4 | 1 | SNEB | 69.00 | 69.00 | 56.20 | 9.83 | 81.5 |
|  |  |  |  |  |  |  |  |
| No.5 | 1 | SNEB | 65.97 | 65.97 | 54.59 | 8.63 | 82.7 |
|  |  |  |  |  |  |  |  |
| No.6 | 1 | SNEB | 75.57 | 75.57 | 60.74 | 10.91 | 80.4 |
|  |  |  |  |  |  |  |  |
| No.7 | 16 | SNEB | 79.49 | 79.49 | 69.93 | 6.42 | 88 |
|  |  |  |  |  |  |  |  |
| No.8 | 16 | SNEB | 70.0 | 70.05 | 58.20 | 8.37 | 83.1 |
|  |  |  |  |  |  |  |  |
| No.9 | 16 | SNEB | 67.28 | 67.27 | 56.13 | 8.42 | 83.4 |
|  |  |  |  |  |  |  |  |
| No.10 | 1 | MNEB | 69.75 | 69.74 | 44.72 | 20.41 | 64.1 |
|  |  |  |  |  |  |  |  |
| No.11 | 1 | MNEB | 75.97 | 75.96 | 39.74 | 14.12 | 52.3 |
|  |  |  |  |  |  |  |  |
| No.12 | 1 | MNEB | 78.90 | 78.89 | 51.27 | 23.72 | 65 |
|  |  |  |  |  |  |  |  |
| No.13 | 16 | MNEB | 74.15 | 74.15 | 57.11 | 13.77 | 77 |
|  |  |  |  |  |  |  |  |
| No.14 | 16 | MNEB | 63.93 | 63.93 | 35.30 | 25.56 | 55.2 |
|  |  |  |  |  |  |  |  |
| No.15 | 16 | MNEB | 65.25 | 65.24 | 43.45 | 18.33 | 66.6 |
|  |  |  |  |  |  |  |  |
