# Supplementary material for: Impact of the severity of negative energy balance on gene expression in the subcutaneous adipose tissue of periparturient primiparous Holstein dairy cows: Identification of potential novel metabolic signals for the reproductive system
Source: PLoS One. 2019 Sep 26;14(9):e0222954. doi: 10.1371/journal.pone.0222954 (PMC6763198; doi:10.1371/journal.pone.0222954)
Supplement: S3 Table — (DOCX) [file pone.0222954.s008.docx]

**S3 Table**: List of primers (Bos Taurus) using for RT-qPCR.

| Gene | size | Forward | Reverse |
| --- | --- | --- | --- |
| *PPIA* | 216 | 5'-GCATACAGGTCCTGGCATCT-3' | 5'-TGTCCACAGTCAGCAATGGT-3' |
| *UXT* | 111 | 5'-CATTGAGCGACTCCAGGAAG-3' | 5'-GGCCACATAGATCCGTGAAG-3' |
| *SDHA* | 168 | 5'-TATATGGCGCTGGCTGTCTC-3' | 5'-CCTCTTCCCTCGCGGATTTC-3' |
| *ADAMTS4* | 111 | 5'-CCGCTTCATCACTGACTTC-3' | 5'-TCAGCGTCGTAGTCCTTG-3' |
| *TF* | 409 | 5'-AAGATGGCACCAGGAAACCTGT-3' | 5'-ATCACTCAGACCAGCGAAACCA-3' |
| *SERPINF1* | 235 | 5'-GTTTTACGGTACGGCTTGGA-3' | 5'-GACTTCGTGAGTTCGCCTTC-3' |
| *CCL21* | 193 | 5'-AGTTGCGCTATGCCAGCTAT-3' | 5'-TTGGAACCCTTTCCCTTCTT-3' |
| *CCR7* | 175 | 5'-GTACGAGTCCGTGTGCTTCA-3' | 5'-GGCTAGGTTGAGCAGGTACG-3' |
| *C1QTNF1* | 185 | 5'-GGACCTAAAGGGCAGAAAGG-3' | 5'-GCAGTAGAATTTCCCCGTGA-3' |
| *POSTN* | 220 | 5'-GTTAGGCTTGGCATCTGCTC-3' | 5'-TGCGGTACACGAAGACTCTG-3' |
| *HIF3A* | 248 | 5'-AGAGACCGAAGTGGTGCTGT-3' | 5'-GCTACTGAAGTCCCGTCCAG-3' |
| *RASGEF1A* | 247 | 5'-CCTTTGACTTCCAGGACGAG-3' | 5'-CACACCCAGGATGTCCTTCT-3' |
| *VASN* | 246 | 5'-AGCTGGACCTGAGCAACCTA-3' | 5'-GGGCAGCCAAAGTCTGAGTA-3' |
| *SERPINE1* | 241 | 5'-GACGGCCGTTACTACGACAT-3' | 5'-TCGGTCATTCCCAAGTTCTC-3' |
| *ANO4* | 224 | 5'-TACCGTGACCCTCCTCATTC-3' | 5'-ACGCTCCAGTTCTGCTTCAT-3' |
| *CPS1* | 200 | 5'-CCAGGCCTAGTAGCATCTCG-3' | 5'-TGCTTGCTTTAAGCCCACTT-3' |
| *SNAP91* | 169 | 5'-GCTGCACTTTCCTCTGTTCC-3' | 5'-GCAAAGGCATCTCCAAAGAG-3' |
| *STAB2* | 240 | 5'-GCTGAAGAATGACCTGCACA-3' | 5'-CTGGGGACACTTTCCACACT-3' |
| *ACADPS* | 153 | 5'-CAGCAGAACGAAGAACACCA-3' | 5'-GGAAACTGTCCCACGTGTCT-3' |
| *MAPK10* | 186 | 5'-CCAGGTTTTTGAAGCTGCTC-3' | 5'-GCAGAGACCCAAGAGCAATC-3' |
| *FGF14* | 248 | 5'-CGGTAACCTGGTGGACATCT-3' | 5'-TACAACCCCGTCTTCACTCC-3' |
| *TRPM8* | 152 | 5'-ACCACACCCACCTACTGCTC-3' | 5'-CTTGGGCAAAACACACAATG-3' |
| *DUSP27* | 190 | 5'-GTGAAACTCTCGCCCAGAAG-3' | 5'-GGTCTCAGAGCTGGGTCTTG-3' |
