# Supplementary material for: Impact of the severity of negative energy balance on gene expression in the subcutaneous adipose tissue of periparturient primiparous Holstein dairy cows: Identification of potential novel metabolic signals for the reproductive system
Source: PLoS One. 2019 Sep 26;14(9):e0222954. doi: 10.1371/journal.pone.0222954 (PMC6763198; doi:10.1371/journal.pone.0222954)
Supplement: S4 Table — (DOCX) [file pone.0222954.s009.docx]

| **S4 Table: Genes differentially expressed at 1 WKPP as compared to -4 WKPP in MNEB animals** | | | | | | |
| --- | --- | --- | --- | --- | --- | --- |
| name | log2FoldChange_exons | pvalue_exons | |  |  |  |
| *ROBO4* | -2,6121772 | 2,78E-23 |  |  |  |  |
| *SPEG* | -2,250023 | 8,07E-23 |  |  |  |  |
| *FAM118A* | -2,2779163 | 2,44E-22 |  |  |  |  |
| *HIF3A* | -4,0095117 | 8,42E-22 |  |  |  |  |
| *PLCG1* | -2,1689376 | 6,36E-21 |  |  |  |  |
| *NOTCH4* | -2,4271447 | 1,04E-20 |  |  |  |  |
| *MADD* | -2,2949524 | 1,91E-19 |  |  |  |  |
| *ADAMTS10* | -2,0576661 | 2,48E-19 |  |  |  |  |
| *FHOD1* | -2,1232242 | 3,95E-19 |  |  |  |  |
| *DGKD* | -2,0124513 | 7,01E-18 |  |  |  |  |
| *ULK1* | -1,9560335 | 6,85E-17 |  |  |  |  |
| *KANK3* | -2,38898 | 9,11E-17 |  |  |  |  |
| *DYSF* | -1,9837999 | 9,93E-17 |  |  |  |  |
| *MYO9B* | -1,7402293 | 2,49E-16 |  |  |  |  |
| *UNC13B* | -1,8644257 | 3,35E-16 |  |  |  |  |
| *BOD1L* | -1,5399765 | 4,02E-16 |  |  |  |  |
| *ALS2CL* | -2,6019968 | 4,73E-16 |  |  |  |  |
| *CASKIN2* | -1,8665412 | 5,09E-16 |  |  |  |  |
| *DEPTOR* | -2,4497924 | 5,85E-16 |  |  |  |  |
| *HAUS5* | -1,8507458 | 1,65E-15 |  |  |  |  |
| *PHRF1* | -1,8164506 | 3,31E-15 |  |  |  |  |
| *CCDC9* | -2,4465698 | 3,70E-15 |  |  |  |  |
| *ARGLU1* | -2,5271898 | 4,44E-15 |  |  |  |  |
| *ABI3* | -2,8713796 | 5,23E-15 |  |  |  |  |
| *CPSF1* | -1,9014655 | 5,72E-15 |  |  |  |  |
| *CCDC183* | -3,3188983 | 5,73E-15 |  |  |  |  |
| *LENG8* | -2,2620908 | 7,04E-15 |  |  |  |  |
| *SLC4A11* | -2,7667168 | 9,77E-15 |  |  |  |  |
| *GRIPAP1* | -2,6348768 | 1,01E-14 |  |  |  |  |
| *CRAMP1L* | -2,1334964 | 1,04E-14 |  |  |  |  |
| *TPM1* | -2,3567107 | 1,07E-14 |  |  |  |  |
| *ARHGEF15* | -2,3827652 | 1,30E-14 |  |  |  |  |
| *ZC3H7B* | -1,7956812 | 1,42E-14 |  |  |  |  |
| *HSF4* | -2,2209413 | 1,78E-14 |  |  |  |  |
| *TAOK2* | -1,7597665 | 2,16E-14 |  |  |  |  |
| *ZNF366* | -2,0363928 | 2,36E-14 |  |  |  |  |
| *MYH9* | -1,6762219 | 2,73E-14 |  |  |  |  |
| *LRRC8A* | -1,5690061 | 3,24E-14 |  |  |  |  |
| *ATG2A* | -2,0412777 | 3,51E-14 |  |  |  |  |
| *RBM19* | -1,7681879 | 4,27E-14 |  |  |  |  |
| *CLASRP* | -2,3898819 | 5,38E-14 |  |  |  |  |
| *POLRMT* | -2,1931753 | 6,77E-14 |  |  |  |  |
| *ATG16L1* | -1,5468832 | 7,40E-14 |  |  |  |  |
| *KANSL3* | -1,7445371 | 7,53E-14 |  |  |  |  |
| *ADGRL1* | -1,7792903 | 8,42E-14 |  |  |  |  |
| *ZSWIM8* | -1,418622 | 1,05E-13 |  |  |  |  |
| *SRRT* | -1,7494694 | 1,15E-13 |  |  |  |  |
| *ANKZF1* | -1,7263593 | 1,22E-13 |  |  |  |  |
| *XPC* | -2,0679715 | 1,42E-13 |  |  |  |  |
| *MAPK12* | -2,9737141 | 1,49E-13 |  |  |  |  |
| *TOP3B* | -2,0832742 | 1,71E-13 |  |  |  |  |
| *NFATC2IP* | -2,4821713 | 1,81E-13 |  |  |  |  |
| *IKBKB* | -1,7035695 | 1,81E-13 |  |  |  |  |
| *OSBP* | -1,7768711 | 2,91E-13 |  |  |  |  |
| *PACSIN3* | -2,4570745 | 4,69E-13 |  |  |  |  |
| *GRAMD1A* | -1,68947 | 5,20E-13 |  |  |  |  |
| *SURF6* | -2,3391454 | 5,40E-13 |  |  |  |  |
| *OSBPL7* | -2,2987635 | 6,42E-13 |  |  |  |  |
| *CHST3* | -1,9404851 | 6,52E-13 |  |  |  |  |
| *TCHP* | -1,9424041 | 6,73E-13 |  |  |  |  |
| *NOP14* | -1,5621295 | 7,17E-13 |  |  |  |  |
| *TRRAP* | -1,5524943 | 8,11E-13 |  |  |  |  |
| *EPHA2* | -2,0191891 | 9,92E-13 |  |  |  |  |
| *HID1* | -2,6455743 | 1,09E-12 |  |  |  |  |
| *FAM193B* | -2,0824182 | 1,22E-12 |  |  |  |  |
| *PRPF3* | -1,3354426 | 1,42E-12 |  |  |  |  |
| *GIGYF1* | -2,155314 | 1,66E-12 |  |  |  |  |
| *SMTN* | -2,2367412 | 1,69E-12 |  |  |  |  |
| *CCDC37* | -3,2340964 | 1,69E-12 |  |  |  |  |
| *DHX37* | -1,8414087 | 1,83E-12 |  |  |  |  |
| *NEURL4* | -1,7535671 | 1,89E-12 |  |  |  |  |
| *PTPN23* | -1,88035 | 2,23E-12 |  |  |  |  |
| *TOP3A* | -1,622 | 2,46E-12 |  |  |  |  |
| *GLE1* | -1,7499913 | 2,82E-12 |  |  |  |  |
| *BRPF1* | -1,576077 | 3,41E-12 |  |  |  |  |
| *ACTN4* | -1,4008417 | 4,69E-12 |  |  |  |  |
| *VSIG2* | -2,5444693 | 4,91E-12 |  |  |  |  |
| *TBC1D8* | -1,5596873 | 5,27E-12 |  |  |  |  |
| *MPRIP* | -1,8864082 | 6,07E-12 |  |  |  |  |
| *RNF123* | -1,8833561 | 6,23E-12 |  |  |  |  |
| *CHST1* | -2,2273254 | 6,49E-12 |  |  |  |  |
| *ACTN1* | -1,4505807 | 6,69E-12 |  |  |  |  |
| *C29H11orf84* | -1,8698297 | 7,23E-12 |  |  |  |  |
| *NR1H3* | -1,4511827 | 8,33E-12 |  |  |  |  |
| *COL18A1* | -1,7428779 | 9,24E-12 |  |  |  |  |
| *GRAMD1B* | -2,606433 | 9,22E-12 |  |  |  |  |
| *ATP2A1* | -2,8948628 | 1,09E-11 |  |  |  |  |
| *DAB2IP* | -1,6499125 | 1,10E-11 |  |  |  |  |
| *MYH10* | -1,4586693 | 1,21E-11 |  |  |  |  |
| *CDK5RAP2* | -2,0780882 | 1,22E-11 |  |  |  |  |
| *PREX2* | -1,3952883 | 1,41E-11 |  |  |  |  |
| *BRD3* | -1,6359943 | 1,47E-11 |  |  |  |  |
| *SETD1A* | -2,007746 | 1,63E-11 |  |  |  |  |
| *CACNA1A* | -1,7226115 | 1,69E-11 |  |  |  |  |
| *PIP5K1C* | -1,9650453 | 1,71E-11 |  |  |  |  |
| *PER2* | -1,6659967 | 2,01E-11 |  |  |  |  |
| *DGAT1* | -1,546666 | 2,18E-11 |  |  |  |  |
| *PRRC2A* | -1,3816198 | 2,19E-11 |  |  |  |  |
| *RNF40* | -1,6750681 | 2,21E-11 |  |  |  |  |
| *ARHGEF17* | -1,2996041 | 2,30E-11 |  |  |  |  |
| *MORC2* | -1,855954 | 2,61E-11 |  |  |  |  |
| *EML3* | -1,715685 | 2,62E-11 |  |  |  |  |
| *SBNO2* | -1,6789718 | 3,33E-11 |  |  |  |  |
| *NES* | -2,396256 | 3,40E-11 |  |  |  |  |
| *SYMPK* | -1,7172016 | 3,41E-11 |  |  |  |  |
| *TRABD* | -1,9794492 | 3,77E-11 |  |  |  |  |
| *TMEM63B* | -1,8387963 | 4,28E-11 |  |  |  |  |
| *RASIP1* | -1,9039662 | 4,32E-11 |  |  |  |  |
| *LZTR1* | -1,6959469 | 4,48E-11 |  |  |  |  |
| *SART1* | -1,6630557 | 4,48E-11 |  |  |  |  |
| *ADAP2* | -1,8255181 | 4,62E-11 |  |  |  |  |
| *EP400* | -1,4022749 | 5,02E-11 |  |  |  |  |
| *ACAD9* | -1,9206828 | 5,14E-11 |  |  |  |  |
| *DHX16* | -1,5590705 | 5,16E-11 |  |  |  |  |
| *ITPR1* | -1,2457826 | 5,25E-11 |  |  |  |  |
| *RRP1* | -1,811793 | 6,49E-11 |  |  |  |  |
| *AP5Z1* | -1,8940136 | 6,90E-11 |  |  |  |  |
| *KLHL17* | -1,9639783 | 7,00E-11 |  |  |  |  |
| *MED12* | -1,2803609 | 7,25E-11 |  |  |  |  |
| *NCAPH2* | -1,6859732 | 7,35E-11 |  |  |  |  |
| *RRP12* | -1,5116573 | 8,39E-11 |  |  |  |  |
| *TONSL* | -2,1929558 | 8,43E-11 |  |  |  |  |
| *PPP1R13L* | -2,106807 | 8,47E-11 |  |  |  |  |
| *GON4L* | -1,350198 | 9,50E-11 |  |  |  |  |
| *ANKRD52* | -1,4754794 | 9,55E-11 |  |  |  |  |
| *ZKSCAN5* | -1,3693872 | 1,05E-10 |  |  |  |  |
| *EHMT1* | -1,6397212 | 1,06E-10 |  |  |  |  |
| *CHMP4A* | -2,1022633 | 1,07E-10 |  |  |  |  |
| *APBB3* | -1,406746 | 1,17E-10 |  |  |  |  |
| *GABBR1* | -1,9397056 | 1,19E-10 |  |  |  |  |
| *STX3* | -2,6515165 | 1,20E-10 |  |  |  |  |
| *MAEA* | -1,6154146 | 1,28E-10 |  |  |  |  |
| *PSEN2* | -1,9641714 | 1,31E-10 |  |  |  |  |
| *ERC1* | -1,8179442 | 1,55E-10 |  |  |  |  |
| *MBD1* | -1,7363351 | 1,65E-10 |  |  |  |  |
| *GNAT1* | -1,9130939 | 1,75E-10 |  |  |  |  |
| *CAMSAP1* | -1,5417584 | 1,83E-10 |  |  |  |  |
| *ZNF592* | -1,3342766 | 1,85E-10 |  |  |  |  |
| *PKN1* | -1,5359555 | 2,33E-10 |  |  |  |  |
| *RIPK3* | -1,7642927 | 2,39E-10 |  |  |  |  |
| *INTS1* | -1,3993797 | 2,56E-10 |  |  |  |  |
| *TBX2* | -1,4992193 | 2,83E-10 |  |  |  |  |
| *OPLAH* | -1,7049068 | 2,92E-10 |  |  |  |  |
| *FILIP1* | -1,5027836 | 3,04E-10 |  |  |  |  |
| *GUCY1B3* | -1,2867236 | 3,12E-10 |  |  |  |  |
| *ANO1* | -1,524344 | 3,23E-10 |  |  |  |  |
| *IKBKG* | -1,7665892 | 3,43E-10 |  |  |  |  |
| *CARD11* | -1,9645923 | 3,46E-10 |  |  |  |  |
| *THSD7A* | -1,5015575 | 3,51E-10 |  |  |  |  |
| *MTA1* | -1,9338179 | 3,55E-10 |  |  |  |  |
| *CCNL2* | -1,3747502 | 3,56E-10 |  |  |  |  |
| *PISD* | -1,4229773 | 3,70E-10 |  |  |  |  |
| *NELFA* | -1,9289456 | 3,71E-10 |  |  |  |  |
| *CXXC1* | -2,3699955 | 3,81E-10 |  |  |  |  |
| *SGSM3* | -1,709129 | 4,10E-10 |  |  |  |  |
| *SGSM1* | -2,1840509 | 4,13E-10 |  |  |  |  |
| *ARID3B* | -2,0781569 | 4,18E-10 |  |  |  |  |
| *FLCN* | -1,4119698 | 4,22E-10 |  |  |  |  |
| *ZBTB40* | -1,6769666 | 4,29E-10 |  |  |  |  |
| *COG1* | -1,4038619 | 4,45E-10 |  |  |  |  |
| *EDN1* | -1,7418488 | 4,66E-10 |  |  |  |  |
| *MAPK8IP3* | -1,9962673 | 4,73E-10 |  |  |  |  |
| *PPP1R37* | -1,6969098 | 5,29E-10 |  |  |  |  |
| *SMARCA4* | -1,7255829 | 5,30E-10 |  |  |  |  |
| *RTEL1* | -1,9795053 | 5,78E-10 |  |  |  |  |
| *ELAC2* | -1,4591739 | 5,77E-10 |  |  |  |  |
| *KRI1* | -1,9292275 | 6,15E-10 |  |  |  |  |
| *CLK3* | -1,4120685 | 6,14E-10 |  |  |  |  |
| *GCC1* | -1,8960246 | 7,05E-10 |  |  |  |  |
| *ATF6B* | -1,6236989 | 7,35E-10 |  |  |  |  |
| *ADCK5* | -1,7491321 | 7,45E-10 |  |  |  |  |
| *PRPF4B* | -1,1650847 | 8,18E-10 |  |  |  |  |
| *ASL* | -1,7434389 | 8,35E-10 |  |  |  |  |
| *SNRNP70* | -1,8089346 | 8,65E-10 |  |  |  |  |
| *TJAP1* | -2,1454946 | 8,84E-10 |  |  |  |  |
| *LRWD1* | -1,6630998 | 8,95E-10 |  |  |  |  |
| *PNPLA6* | -1,5559398 | 9,17E-10 |  |  |  |  |
| *NOVA2* | -2,1760633 | 9,51E-10 |  |  |  |  |
| *SLC25A28* | -1,4550935 | 1,04E-09 |  |  |  |  |
| *GMEB2* | -1,5126386 | 1,07E-09 |  |  |  |  |
| *RASGRP2* | -1,8411737 | 1,13E-09 |  |  |  |  |
| *PIH1D1* | -2,036957 | 1,15E-09 |  |  |  |  |
| *FZR1* | -1,5900601 | 1,15E-09 |  |  |  |  |
| *MAD1L1* | -1,9687283 | 1,16E-09 |  |  |  |  |
| *ZNF629* | -1,5472306 | 1,17E-09 |  |  |  |  |
| *DOCK6* | -1,8710674 | 1,18E-09 |  |  |  |  |
| *GNPTG* | -1,6212782 | 1,19E-09 |  |  |  |  |
| *TNFRSF4* | -2,3213501 | 1,29E-09 |  |  |  |  |
| *TOM1L2* | -1,7936542 | 1,34E-09 |  |  |  |  |
| *FBXO10* | -1,6645569 | 1,35E-09 |  |  |  |  |
| *ULK3* | -1,5826955 | 1,50E-09 |  |  |  |  |
| *PER1* | -1,7258467 | 1,51E-09 |  |  |  |  |
| *SART3* | -1,2182791 | 1,57E-09 |  |  |  |  |
| *NR2C2* | -1,295637 | 1,61E-09 |  |  |  |  |
| *RANGAP1* | -1,6114111 | 1,61E-09 |  |  |  |  |
| *PEX6* | -1,4753907 | 1,75E-09 |  |  |  |  |
| *ADRBK1* | -1,4504234 | 1,82E-09 |  |  |  |  |
| *LIG1* | -2,4367932 | 1,92E-09 |  |  |  |  |
| *MTG1* | -1,8149766 | 2,00E-09 |  |  |  |  |
| *ANKS3* | -1,6248967 | 2,13E-09 |  |  |  |  |
| *SH2D3C* | -1,7394553 | 2,20E-09 |  |  |  |  |
| *UBAP2L* | -1,3273462 | 2,27E-09 |  |  |  |  |
| *PTPRE* | -1,5996849 | 2,30E-09 |  |  |  |  |
| *MROH1* | -1,4491223 | 2,37E-09 |  |  |  |  |
| *MICALL1* | -1,9608018 | 2,37E-09 |  |  |  |  |
| *CDK11B* | -1,561629 | 2,60E-09 |  |  |  |  |
| *IGF2* | -1,6550557 | 2,67E-09 |  |  |  |  |
| *sept-04* | -1,5443043 | 2,69E-09 |  |  |  |  |
| *PEAR1* | -1,4191125 | 2,75E-09 |  |  |  |  |
| *EIF2D* | -1,4508529 | 3,24E-09 |  |  |  |  |
| *SMPD4* | -1,6029827 | 3,45E-09 |  |  |  |  |
| *MYH3* | -2,4028812 | 3,48E-09 |  |  |  |  |
| *U2AF2* | -1,3882116 | 3,62E-09 |  |  |  |  |
| *PTH1R* | -1,8810948 | 3,74E-09 |  |  |  |  |
| *SLC12A4* | -1,1188022 | 3,79E-09 |  |  |  |  |
| *SPTAN1* | -1,0986768 | 3,80E-09 |  |  |  |  |
| *GTPBP3* | -1,7604482 | 3,86E-09 |  |  |  |  |
| *MICALL2* | -1,9308467 | 3,91E-09 |  |  |  |  |
| *RHOT2* | -1,6604593 | 4,21E-09 |  |  |  |  |
| *AXIN1* | -1,6976415 | 4,38E-09 |  |  |  |  |
| *ZNF335* | -1,5283377 | 4,52E-09 |  |  |  |  |
| *PAK4* | -1,9395578 | 4,66E-09 |  |  |  |  |
| *ABLIM3* | -1,6591806 | 4,73E-09 |  |  |  |  |
| *SPECC1L* | -1,4023563 | 4,81E-09 |  |  |  |  |
| *MAP6D1* | -1,8230222 | 5,24E-09 |  |  |  |  |
| *PPP1R18* | -1,4485757 | 5,24E-09 |  |  |  |  |
| *GTPBP2* | -1,3669123 | 5,76E-09 |  |  |  |  |
| *ODF2* | -1,7311154 | 5,93E-09 |  |  |  |  |
| *SSNA1* | -1,8823836 | 5,98E-09 |  |  |  |  |
| *PCID2* | -1,4745682 | 6,21E-09 |  |  |  |  |
| *MARK4* | -1,50697 | 6,33E-09 |  |  |  |  |
| *DLG5* | -1,368081 | 6,33E-09 |  |  |  |  |
| *ADCY4* | -1,750562 | 6,43E-09 |  |  |  |  |
| *LRCH4* | -1,4435845 | 7,57E-09 |  |  |  |  |
| *SLC26A6* | -1,6267569 | 7,92E-09 |  |  |  |  |
| *PLXNB2* | -1,6348834 | 7,94E-09 |  |  |  |  |
| *USP42* | -1,3883363 | 8,33E-09 |  |  |  |  |
| *MKNK2* | -1,4583339 | 8,95E-09 |  |  |  |  |
| *TMEM206* | -1,7217499 | 9,12E-09 |  |  |  |  |
| *ARHGAP39* | -2,1545924 | 1,09E-08 |  |  |  |  |
| *KIFC2* | -1,8918091 | 1,13E-08 |  |  |  |  |
| *PAXIP1* | -1,636495 | 1,16E-08 |  |  |  |  |
| *DYRK1B* | -1,2349393 | 1,23E-08 |  |  |  |  |
| *RGS3* | -1,7048253 | 1,23E-08 |  |  |  |  |
| *ABTB1* | -1,7829035 | 1,25E-08 |  |  |  |  |
| *NOS3* | -2,0355636 | 1,35E-08 |  |  |  |  |
| *DYNC1H1* | -1,0040458 | 1,66E-08 |  |  |  |  |
| *EVL* | -1,7743522 | 1,78E-08 |  |  |  |  |
| *MGRN1* | -1,2486482 | 1,83E-08 |  |  |  |  |
| *ZNF697* | -2,0218786 | 1,87E-08 |  |  |  |  |
| *HIP1* | -1,6150597 | 1,87E-08 |  |  |  |  |
| *SNCAIP* | -1,9499536 | 1,89E-08 |  |  |  |  |
| *ARHGEF28* | -1,6784618 | 1,98E-08 |  |  |  |  |
| *PPFIA1* | -1,3169463 | 2,14E-08 |  |  |  |  |
| *CSRP2BP* | -1,1636971 | 2,16E-08 |  |  |  |  |
| *ATP13A1* | -1,2510419 | 2,20E-08 |  |  |  |  |
| *ACVR2B* | -2,2583674 | 2,21E-08 |  |  |  |  |
| *OSBPL3* | -1,629182 | 2,26E-08 |  |  |  |  |
| *SRSF7* | -1,2355305 | 2,31E-08 |  |  |  |  |
| *HDGFRP2* | -1,4978458 | 2,33E-08 |  |  |  |  |
| *STARD3* | -1,6110531 | 2,36E-08 |  |  |  |  |
| *ATXN2L* | -1,1723462 | 2,38E-08 |  |  |  |  |
| *SF3A1* | -1,2259868 | 2,58E-08 |  |  |  |  |
| *FLNB* | -1,4134117 | 2,61E-08 |  |  |  |  |
| *STK19* | -1,2895143 | 2,84E-08 |  |  |  |  |
| *PLEKHJ1* | -1,7023967 | 2,98E-08 |  |  |  |  |
| *ACAP1* | -1,6523385 | 3,30E-08 |  |  |  |  |
| *PPRC1* | -1,3604798 | 3,31E-08 |  |  |  |  |
| *PLK3* | -2,038156 | 3,44E-08 |  |  |  |  |
| *C25H7orf50* | -1,8617136 | 3,58E-08 |  |  |  |  |
| *TNIP2* | -1,9162131 | 3,86E-08 |  |  |  |  |
| *TMEM44* | -1,7667576 | 3,85E-08 |  |  |  |  |
| *SUGP1* | -1,5867831 | 3,95E-08 |  |  |  |  |
| *NCAPD3* | -1,2580405 | 3,95E-08 |  |  |  |  |
| *PIDD1* | -2,0413739 | 3,99E-08 |  |  |  |  |
| *PLA2G4B* | -1,789859 | 4,03E-08 |  |  |  |  |
| *CCAR2* | -1,1773061 | 4,31E-08 |  |  |  |  |
| *SIPA1L2* | -1,3408747 | 4,39E-08 |  |  |  |  |
| *CHERP* | -1,3443718 | 4,44E-08 |  |  |  |  |
| *ING5* | -1,5940258 | 4,57E-08 |  |  |  |  |
| *LMCD1* | -1,3937024 | 4,84E-08 |  |  |  |  |
| *TPCN2* | -1,5450465 | 5,13E-08 |  |  |  |  |
| *AZI1* | -2,0146993 | 5,16E-08 |  |  |  |  |
| *ZER1* | -1,4825414 | 5,39E-08 |  |  |  |  |
| *STK25* | -1,7722042 | 5,72E-08 |  |  |  |  |
| *NUMA1* | -1,3607893 | 5,80E-08 |  |  |  |  |
| *PLEKHG3* | -1,1240647 | 5,88E-08 |  |  |  |  |
| *PKN3* | -1,6858347 | 5,89E-08 |  |  |  |  |
| *RASGEF1A* | -2,1717038 | 5,98E-08 |  |  |  |  |
| *EHBP1L1* | -1,3446935 | 6,45E-08 |  |  |  |  |
| *DLL4* | -1,5922955 | 6,62E-08 |  |  |  |  |
| *DGKZ* | -1,7313153 | 6,63E-08 |  |  |  |  |
| *NOC2L* | -1,2552346 | 7,14E-08 |  |  |  |  |
| *UBR4* | -0,9779313 | 7,19E-08 |  |  |  |  |
| *MAP3K14* | -1,7064152 | 7,54E-08 |  |  |  |  |
| *DNM2* | -1,1933821 | 7,85E-08 |  |  |  |  |
| *DPP7* | -1,6940044 | 9,31E-08 |  |  |  |  |
| *TAF6L* | -1,4641547 | 9,65E-08 |  |  |  |  |
| *CORO7* | -1,6398514 | 9,70E-08 |  |  |  |  |
| *NARFL* | -1,6853814 | 1,03E-07 |  |  |  |  |
| *DDX39B* | -1,1561699 | 1,08E-07 |  |  |  |  |
| *ELF4* | -1,4092236 | 1,08E-07 |  |  |  |  |
| *INPP5E* | -1,7840369 | 1,09E-07 |  |  |  |  |
| *ERCC6* | -1,1754589 | 1,10E-07 |  |  |  |  |
| *POLR3D* | -1,5401418 | 1,11E-07 |  |  |  |  |
| *NOP2* | -1,2530708 | 1,20E-07 |  |  |  |  |
| *LPIN3* | -1,4476771 | 1,34E-07 |  |  |  |  |
| *AMPD2* | -1,2561517 | 1,34E-07 |  |  |  |  |
| *SEMA7A* | -1,4175601 | 1,35E-07 |  |  |  |  |
| *COL13A1* | -2,7567215 | 1,38E-07 |  |  |  |  |
| *PHF1* | -1,3800397 | 1,38E-07 |  |  |  |  |
| *TRAF7* | -1,5410726 | 1,42E-07 |  |  |  |  |
| *NADSYN1* | -1,3617652 | 1,43E-07 |  |  |  |  |
| *PRPF6* | -1,1557104 | 1,46E-07 |  |  |  |  |
| *CCDC84* | -1,6077357 | 1,54E-07 |  |  |  |  |
| *DHX38* | -1,3260601 | 1,61E-07 |  |  |  |  |
| *LAMB3* | -1,7687155 | 1,66E-07 |  |  |  |  |
| *MINK1* | -1,988356 | 1,71E-07 |  |  |  |  |
| *LETM1* | -1,3739128 | 1,71E-07 |  |  |  |  |
| *DHX34* | -1,2689615 | 1,78E-07 |  |  |  |  |
| *THBS2* | -1,7459617 | 1,91E-07 |  |  |  |  |
| *NLE1* | -1,7691618 | 2,00E-07 |  |  |  |  |
| *CAD* | -1,1937586 | 2,11E-07 |  |  |  |  |
| *MAP3K11* | -1,4029568 | 2,12E-07 |  |  |  |  |
| *DHX30* | -1,1703163 | 2,13E-07 |  |  |  |  |
| *MOB2* | -1,6173933 | 2,36E-07 |  |  |  |  |
| *E4F1* | -1,5194731 | 2,49E-07 |  |  |  |  |
| *RPS6KA4* | -1,3626931 | 2,66E-07 |  |  |  |  |
| *TAF6* | -1,6567381 | 2,81E-07 |  |  |  |  |
| *FAM73B* | -1,3235287 | 2,84E-07 |  |  |  |  |
| *RABEP2* | -1,5585458 | 2,89E-07 |  |  |  |  |
| *ADCK3* | -1,5188054 | 2,98E-07 |  |  |  |  |
| *RBM10* | -1,2548774 | 2,99E-07 |  |  |  |  |
| *CLK2* | -1,2164761 | 3,04E-07 |  |  |  |  |
| *NDOR1* | -1,6420985 | 3,09E-07 |  |  |  |  |
| *PCBP4* | -1,5857569 | 3,12E-07 |  |  |  |  |
| *FBF1* | -1,7731106 | 3,14E-07 |  |  |  |  |
| *ITPR3* | -2,0150012 | 3,19E-07 |  |  |  |  |
| *WWP2* | -1,0328124 | 3,27E-07 |  |  |  |  |
| *SUPT5H* | -1,3137954 | 3,40E-07 |  |  |  |  |
| *BRMS1* | -1,7375316 | 3,52E-07 |  |  |  |  |
| *KLC2* | -1,9576993 | 3,54E-07 |  |  |  |  |
| *DGCR8* | -1,3767628 | 3,81E-07 |  |  |  |  |
| *QTRT1* | -1,4440348 | 4,06E-07 |  |  |  |  |
| *TAF1C* | -1,3172395 | 4,07E-07 |  |  |  |  |
| *BCL6B* | -1,4321426 | 4,08E-07 |  |  |  |  |
| *ITGA2B* | -1,6286569 | 4,24E-07 |  |  |  |  |
| *TCAP* | -1,4101589 | 4,68E-07 |  |  |  |  |
| *KAT5* | -1,3662003 | 5,19E-07 |  |  |  |  |
| *TBL3* | -1,4214945 | 5,20E-07 |  |  |  |  |
| *HECTD3* | -1,2436023 | 5,26E-07 |  |  |  |  |
| *HGS* | -1,215303 | 5,30E-07 |  |  |  |  |
| *BCL9L* | -1,2749474 | 5,33E-07 |  |  |  |  |
| *GAK* | -1,353043 | 6,01E-07 |  |  |  |  |
| *ITGA7* | -1,259947 | 6,04E-07 |  |  |  |  |
| *HEATR4* | -1,6169002 | 6,05E-07 |  |  |  |  |
| *VPS52* | -1,0528788 | 6,33E-07 |  |  |  |  |
| *TRIM7* | -1,4434786 | 6,64E-07 |  |  |  |  |
| *GGA1* | -1,8532132 | 6,74E-07 |  |  |  |  |
| *TMC6* | -1,362745 | 6,79E-07 |  |  |  |  |
| *LOC616942* | -1,5886555 | 7,45E-07 |  |  |  |  |
| *COL11A2* | -2,8828391 | 7,65E-07 |  |  |  |  |
| *BAZ1B* | -0,9475137 | 7,81E-07 |  |  |  |  |
| *ZNF205* | -1,3765496 | 7,89E-07 |  |  |  |  |
| *NCAPH* | -1,7954144 | 8,18E-07 |  |  |  |  |
| *CDRT4* | -2,0765297 | 8,37E-07 |  |  |  |  |
| *FMNL3* | -1,2079485 | 8,41E-07 |  |  |  |  |
| *AMT* | -1,1526246 | 8,52E-07 |  |  |  |  |
| *CARD9* | -1,8663443 | 8,52E-07 |  |  |  |  |
| *DNAJB2* | -1,2010325 | 8,51E-07 |  |  |  |  |
| *IFFO1* | -1,335647 | 8,63E-07 |  |  |  |  |
| *CC2D1A* | -1,4279743 | 9,84E-07 |  |  |  |  |
| *EFNA1* | -1,513103 | 9,83E-07 |  |  |  |  |
| *ITGA10* | -1,466401 | 1,06E-06 |  |  |  |  |
| *UNC5B* | -1,2404686 | 1,10E-06 |  |  |  |  |
| *RABL6* | -1,4315902 | 1,10E-06 |  |  |  |  |
| *USP11* | -1,1411079 | 1,15E-06 |  |  |  |  |
| *MIER2* | -1,2781782 | 1,21E-06 |  |  |  |  |
| *ARFGAP1* | -1,5950162 | 1,22E-06 |  |  |  |  |
| *FANCA* | -1,4185849 | 1,23E-06 |  |  |  |  |
| *VARS2* | -1,4413849 | 1,26E-06 |  |  |  |  |
| *ANKRD13D* | -1,6928659 | 1,27E-06 |  |  |  |  |
| *CNTNAP1* | -1,2579641 | 1,36E-06 |  |  |  |  |
| *GAS8* | -2,2806082 | 1,39E-06 |  |  |  |  |
| *SPG7* | -1,308165 | 1,40E-06 |  |  |  |  |
| *CDH20* | -1,8461282 | 1,41E-06 |  |  |  |  |
| *RAD9A* | -1,4605097 | 1,41E-06 |  |  |  |  |
| *TRIM45* | -1,8265288 | 1,58E-06 |  |  |  |  |
| *SH3BP2* | -1,5922512 | 1,61E-06 |  |  |  |  |
| *MYBL2* | -1,8655937 | 1,67E-06 |  |  |  |  |
| *GTF3C5* | -1,5828392 | 1,68E-06 |  |  |  |  |
| *TNFRSF10D* | -1,3548412 | 1,76E-06 |  |  |  |  |
| *ZNF777* | -1,2020287 | 1,77E-06 |  |  |  |  |
| *APBA3* | -1,2540575 | 1,80E-06 |  |  |  |  |
| *NCKAP5L* | -1,5669555 | 1,80E-06 |  |  |  |  |
| *PRR14* | -1,4656095 | 1,81E-06 |  |  |  |  |
| *TARS2* | -1,1533378 | 1,81E-06 |  |  |  |  |
| *ZC3H4* | -1,2800099 | 1,84E-06 |  |  |  |  |
| *TFIP11* | -1,1657357 | 1,87E-06 |  |  |  |  |
| *RSPH3* | -1,7963256 | 1,90E-06 |  |  |  |  |
| *TRMT1* | -1,3356818 | 2,00E-06 |  |  |  |  |
| *CAMTA2* | -1,0945165 | 2,01E-06 |  |  |  |  |
| *GNB3* | -1,8241759 | 2,09E-06 |  |  |  |  |
| *SSH3* | -1,5285479 | 2,15E-06 |  |  |  |  |
| *DUS3L* | -1,2859346 | 2,16E-06 |  |  |  |  |
| *EXOC3* | -1,0588583 | 2,18E-06 |  |  |  |  |
| *TICAM1* | -1,7626715 | 2,23E-06 |  |  |  |  |
| *SLC25A37* | -0,9817481 | 2,24E-06 |  |  |  |  |
| *MAP3K12* | -1,5199251 | 2,27E-06 |  |  |  |  |
| *NFKBIL1* | -1,352836 | 2,31E-06 |  |  |  |  |
| *WDR25* | -1,6867284 | 2,36E-06 |  |  |  |  |
| *CDK3* | -1,4958624 | 2,37E-06 |  |  |  |  |
| *HDAC7* | -1,3610118 | 2,52E-06 |  |  |  |  |
| *NOLC1* | -1,1099708 | 2,57E-06 |  |  |  |  |
| *AARS2* | -1,2454186 | 2,97E-06 |  |  |  |  |
| *PLCB3* | -1,3702343 | 3,09E-06 |  |  |  |  |
| *EGFL7* | -1,716141 | 3,19E-06 |  |  |  |  |
| *ASB6* | -1,4268221 | 3,21E-06 |  |  |  |  |
| *EXOC3L1* | -1,8105992 | 3,29E-06 |  |  |  |  |
| *MAP3K6* | -1,4058196 | 3,32E-06 |  |  |  |  |
| *LANCL3* | -1,7687921 | 3,69E-06 |  |  |  |  |
| *NRADD* | -1,1464076 | 3,75E-06 |  |  |  |  |
| *TBC1D13* | -1,3693939 | 3,75E-06 |  |  |  |  |
| *CCDC102A* | -1,731384 | 3,79E-06 |  |  |  |  |
| *GORASP1* | -1,2342641 | 3,85E-06 |  |  |  |  |
| *GPKOW* | -1,2107798 | 3,97E-06 |  |  |  |  |
| *TRIP10* | -1,3257749 | 4,00E-06 |  |  |  |  |
| *TNNT2* | -1,9643117 | 4,07E-06 |  |  |  |  |
| *TMCC3* | -1,3754798 | 4,10E-06 |  |  |  |  |
| *ZNF34* | -1,0417864 | 4,22E-06 |  |  |  |  |
| *MYO1C* | -0,8490397 | 4,47E-06 |  |  |  |  |
| *DLG4* | -1,5193103 | 4,57E-06 |  |  |  |  |
| *SYNGAP1* | -1,8731632 | 4,75E-06 |  |  |  |  |
| *ENG* | -1,6414495 | 4,77E-06 |  |  |  |  |
| *TSPAN4* | -1,1905483 | 4,78E-06 |  |  |  |  |
| *URB1* | -1,1606381 | 4,81E-06 |  |  |  |  |
| *RPS6KB2* | -1,2976481 | 5,02E-06 |  |  |  |  |
| *TADA3* | -1,264679 | 5,13E-06 |  |  |  |  |
| *SPSB3* | -1,3291142 | 5,17E-06 |  |  |  |  |
| *INPPL1* | -1,0847933 | 5,18E-06 |  |  |  |  |
| *PRPF31* | -1,3151701 | 5,79E-06 |  |  |  |  |
| *ELF3* | -2,3986706 | 5,85E-06 |  |  |  |  |
| *PITPNM2* | -1,1985777 | 5,86E-06 |  |  |  |  |
| *PHC1* | -1,2340544 | 5,87E-06 |  |  |  |  |
| *UBN1* | -1,0387895 | 6,04E-06 |  |  |  |  |
| *ABCF1* | -1,0507515 | 6,63E-06 |  |  |  |  |
| *LOC513767* | -1,4011757 | 6,69E-06 |  |  |  |  |
| *DVL1* | -1,328568 | 7,24E-06 |  |  |  |  |
| *TMEM140* | -1,0404454 | 7,40E-06 |  |  |  |  |
| *CCDC137* | -1,3208174 | 7,41E-06 |  |  |  |  |
| *RAB20* | -2,4624554 | 7,44E-06 |  |  |  |  |
| *RECQL5* | -1,2545693 | 7,59E-06 |  |  |  |  |
| *PDE4A* | -1,219098 | 7,73E-06 |  |  |  |  |
| *DHX33* | -1,2092964 | 7,79E-06 |  |  |  |  |
| *RFXANK* | -1,6289218 | 8,13E-06 |  |  |  |  |
| *PABPN1* | -1,2610843 | 8,16E-06 |  |  |  |  |
| *ARHGEF10L* | -1,3043523 | 8,20E-06 |  |  |  |  |
| *RPAP1* | -1,0709509 | 8,24E-06 |  |  |  |  |
| *GMEB1* | -1,085676 | 8,35E-06 |  |  |  |  |
| *DAGLB* | -1,1209942 | 8,34E-06 |  |  |  |  |
| *PPP1R16A* | -1,6106123 | 8,67E-06 |  |  |  |  |
| *DDX41* | -1,2111527 | 8,76E-06 |  |  |  |  |
| *LLGL2* | -1,5296252 | 8,77E-06 |  |  |  |  |
| *ZNF692* | -1,4117604 | 9,12E-06 |  |  |  |  |
| *DGKQ* | -1,5233832 | 9,23E-06 |  |  |  |  |
| *CTU2* | -1,2623995 | 9,23E-06 |  |  |  |  |
| *CCNF* | -2,0372305 | 9,32E-06 |  |  |  |  |
| *SIPA1* | -1,212694 | 9,86E-06 |  |  |  |  |
| *TCIRG1* | -1,2224071 | 1,02E-05 |  |  |  |  |
| *RUVBL2* | -1,3312227 | 1,03E-05 |  |  |  |  |
| *EVA1C* | -1,4047295 | 1,03E-05 |  |  |  |  |
| *USHBP1* | -1,3651864 | 1,04E-05 |  |  |  |  |
| *SLC4A2* | -1,0672411 | 1,07E-05 |  |  |  |  |
| *TCF20* | -0,8714919 | 1,12E-05 |  |  |  |  |
| *MAF* | -1,1120929 | 1,12E-05 |  |  |  |  |
| *MAFG* | -1,4930152 | 1,18E-05 |  |  |  |  |
| *BAZ2A* | -0,8940419 | 1,27E-05 |  |  |  |  |
| *HCRTR1* | -1,7686245 | 1,28E-05 |  |  |  |  |
| *SHKBP1* | -1,2860655 | 1,29E-05 |  |  |  |  |
| *TRIM47* | -1,3336831 | 1,31E-05 |  |  |  |  |
| *ITGB4* | -1,531344 | 1,33E-05 |  |  |  |  |
| *MAN2C1* | -1,0857406 | 1,43E-05 |  |  |  |  |
| *RBFOX3* | -2,0172494 | 1,50E-05 |  |  |  |  |
| *MAN2A2* | -0,9867686 | 1,52E-05 |  |  |  |  |
| *FAM50A* | -1,3828125 | 1,54E-05 |  |  |  |  |
| *TUBG2* | -1,3514759 | 1,66E-05 |  |  |  |  |
| *SMARCC2* | -1,1103044 | 1,67E-05 |  |  |  |  |
| *FARSA* | -1,0805422 | 1,71E-05 |  |  |  |  |
| *EHMT2* | -1,2332871 | 1,74E-05 |  |  |  |  |
| *FCHSD1* | -1,3173391 | 1,82E-05 |  |  |  |  |
| *FBXW5* | -1,2600777 | 1,82E-05 |  |  |  |  |
| *ANGPT2* | -1,0784319 | 1,84E-05 |  |  |  |  |
| *MAP1S* | -1,2219536 | 1,85E-05 |  |  |  |  |
| *ERCC2* | -1,2349121 | 2,06E-05 |  |  |  |  |
| *CDH23* | -1,5957104 | 2,10E-05 |  |  |  |  |
| *DEDD2* | -1,3557981 | 2,15E-05 |  |  |  |  |
| *GGA3* | -1,0188581 | 2,25E-05 |  |  |  |  |
| *ARHGEF16* | -2,7010546 | 2,46E-05 |  |  |  |  |
| *ATG4B* | -1,3078417 | 2,53E-05 |  |  |  |  |
| *CCDC88B* | -1,6938046 | 2,62E-05 |  |  |  |  |
| *FTSJ3* | -1,199505 | 2,73E-05 |  |  |  |  |
| *BCAR1* | -1,4401094 | 2,76E-05 |  |  |  |  |
| *DNASE1* | -1,1370213 | 3,20E-05 |  |  |  |  |
| *UNC45A* | -0,8403856 | 3,34E-05 |  |  |  |  |
| *PIK3CD* | -1,2044839 | 3,36E-05 |  |  |  |  |
| *RHOB* | -1,2339851 | 3,37E-05 |  |  |  |  |
| *ARHGEF2* | -0,9263804 | 3,54E-05 |  |  |  |  |
| *ERG* | -1,018141 | 3,56E-05 |  |  |  |  |
| *ZBTB48* | -1,3105368 | 3,70E-05 |  |  |  |  |
| *DCTN1* | -1,0276782 | 3,82E-05 |  |  |  |  |
| *ACAP3* | -1,2956798 | 3,94E-05 |  |  |  |  |
| *PPP2R5B* | -1,0869877 | 4,16E-05 |  |  |  |  |
| *BATF2* | -2,0104589 | 4,27E-05 |  |  |  |  |
| *ZNF608* | -1,2030854 | 4,28E-05 |  |  |  |  |
| *SH3GLB2* | -1,3680049 | 4,40E-05 |  |  |  |  |
| *SLC26A10* | -1,4064781 | 4,50E-05 |  |  |  |  |
| *SEMA4C* | -0,9977565 | 4,63E-05 |  |  |  |  |
| *SRSF2* | -0,7766166 | 4,64E-05 |  |  |  |  |
| *TNFAIP2* | -1,3621799 | 4,90E-05 |  |  |  |  |
| *SLC6A1* | -1,5197 | 5,26E-05 |  |  |  |  |
| *RRP8* | -1,1641742 | 5,52E-05 |  |  |  |  |
| *TYK2* | -0,80836 | 6,36E-05 |  |  |  |  |
| *RN7SL1* | -2,6573323 | 6,55E-05 |  |  |  |  |
| *MAP4K2* | -1,5100186 | 6,55E-05 |  |  |  |  |
| *MAMDC4* | -1,7631449 | 6,61E-05 |  |  |  |  |
| *NRBP2* | -1,4637601 | 6,67E-05 |  |  |  |  |
| *FPGS* | -1,3219343 | 6,75E-05 |  |  |  |  |
| *GTSE1* | -1,7067081 | 7,33E-05 |  |  |  |  |
| *GDPD3* | -1,667788 | 7,49E-05 |  |  |  |  |
| *ARRDC2* | -1,0701442 | 7,66E-05 |  |  |  |  |
| *SH2B1* | -1,1019865 | 7,80E-05 |  |  |  |  |
| *FANCG* | -0,9888692 | 7,93E-05 |  |  |  |  |
| *TSNARE1* | -1,4644633 | 7,96E-05 |  |  |  |  |
| *ITGA5* | -0,9734441 | 8,03E-05 |  |  |  |  |
| *PNKD* | -1,2171717 | 8,79E-05 |  |  |  |  |
| *EMID1* | -1,7221401 | 8,83E-05 |  |  |  |  |
| *SLC43A3* | -1,5342841 | 8,98E-05 |  |  |  |  |
| *DGCR2* | -1,1549963 | 9,38E-05 |  |  |  |  |
| *RDH13* | -1,2171163 | 9,38E-05 |  |  |  |  |
| *SYBU* | -1,2580094 | 9,50E-05 |  |  |  |  |
| *LYL1* | -1,4956383 | 9,64E-05 |  |  |  |  |
| *PBX2* | -1,0918353 | 9,87E-05 |  |  |  |  |
| *UBQLN4* | -1,0708848 | 9,95E-05 |  |  |  |  |
| *SIGIRR* | -1,3802601 | 0,00010013 |  |  |  |  |
| *POLD1* | -1,4442049 | 0,00010112 |  |  |  |  |
| *C8G* | -1,4238726 | 0,00010253 |  |  |  |  |
| *FAAH* | -1,5057548 | 0,0001084 |  |  |  |  |
| *ASMT* | -1,8456627 | 0,00011242 |  |  |  |  |
| *MUM1* | -0,9473176 | 0,00012108 |  |  |  |  |
| *SIRT5* | -1,2394769 | 0,00012487 |  |  |  |  |
| *VPS72* | -1,1589165 | 0,00013424 |  |  |  |  |
| *HNRNPDL* | -0,7664419 | 0,00013962 |  |  |  |  |
| *CCDC22* | -1,0582246 | 0,00014684 |  |  |  |  |
| *N4BP3* | -1,5407533 | 0,00015272 |  |  |  |  |
| *CORO1B* | -0,9729199 | 0,0001743 |  |  |  |  |
| *KCNJ2* | -1,6264397 | 0,00017525 |  |  |  |  |
| *CXHXorf36* | -0,9026525 | 0,00017724 |  |  |  |  |
| *CDK5RAP3* | -0,9647948 | 0,00018631 |  |  |  |  |
| *PNN* | -0,903582 | 0,00019039 |  |  |  |  |
| *MIR143* | -2,0293729 | 0,00019186 |  |  |  |  |
| *NOL6* | -1,3490696 | 0,00020142 |  |  |  |  |
| *NOXA1* | -1,6834902 | 0,00020238 |  |  |  |  |
| *PPP6R1* | -1,3200444 | 0,00020317 |  |  |  |  |
| *TRAPPC12* | -0,9570684 | 0,00020589 |  |  |  |  |
| *CREBZF* | -1,0343965 | 0,00023244 |  |  |  |  |
| *PAN2* | -0,8633014 | 0,00023317 |  |  |  |  |
| *ABCF2* | -0,9241855 | 0,0002396 |  |  |  |  |
| *PVRL2* | -0,9860637 | 0,00024334 |  |  |  |  |
| *FA2H* | -2,6776752 | 0,00025055 |  |  |  |  |
| *SERPINB9* | -1,3802564 | 0,00025392 |  |  |  |  |
| *ME3* | -0,9992873 | 0,00026456 |  |  |  |  |
| *TIMELESS* | -0,9150878 | 0,00027551 |  |  |  |  |
| *LONP1* | -0,8300966 | 0,00030369 |  |  |  |  |
| *ABCF3* | -0,7801076 | 0,00030865 |  |  |  |  |
| *FAM214B* | -0,8291909 | 0,00031882 |  |  |  |  |
| *PDCD11* | -0,8170222 | 0,00033892 |  |  |  |  |
| *DPF1* | -2,3981374 | 0,00035382 |  |  |  |  |
| *FAM19A5* | -2,019776 | 0,00035775 |  |  |  |  |
| *LYAR* | -1,0047379 | 0,00035926 |  |  |  |  |
| *PICK1* | -1,0483109 | 0,00037045 |  |  |  |  |
| *CCDC88C* | -1,0282213 | 0,00039919 |  |  |  |  |
| *ENKD1* | -1,3353673 | 0,00040451 |  |  |  |  |
| *NAT9* | -1,1000132 | 0,00041875 |  |  |  |  |
| *EEFSEC* | -1,1239959 | 0,00043496 |  |  |  |  |
| *BCAR3* | -1,0341293 | 0,00044517 |  |  |  |  |
| *SYNE2* | -0,7767205 | 0,0004514 |  |  |  |  |
| *NOB1* | -0,9972212 | 0,00047414 |  |  |  |  |
| *NPR2* | -0,7976645 | 0,00049874 |  |  |  |  |
| *KCNIP3* | -1,8326737 | 0,00050132 |  |  |  |  |
| *VAT1L* | -1,8584571 | 0,0005877 |  |  |  |  |
| *CYHR1* | -0,9383338 | 0,00060566 |  |  |  |  |
| *TIE1* | -1,0442983 | 0,00064646 |  |  |  |  |
| *PCDH1* | -0,8175242 | 0,00081196 |  |  |  |  |
| *PRKAR1B* | -1,1038043 | 0,00088778 |  |  |  |  |
| *TBC1D2* | -1,1495947 | 0,00097393 |  |  |  |  |
| *SLC9A3R2* | -1,0026213 | 0,00102357 |  |  |  |  |
| *TGIF2* | -1,1217928 | 0,00140977 |  |  |  |  |
| *DMD* | -1,3190667 | 0,00152923 |  |  |  |  |
| *MDN1* | -0,7313189 | 0,00182145 |  |  |  |  |
| *WRAP53* | -0,9443137 | 0,00187188 |  |  |  |  |
| *ADAMTS4* | -2,1950539 | 0,00230288 |  |  |  |  |
| *PDK4* | -2,1030805 | 3,98E-07 |  |  |  |  |
| *PLXNA4* | -1,2123362 | 0,0003402 |  |  |  |  |
| *ADM* | -2,2592309 | 3,33E-12 |  |  |  |  |
| *NRIP3* | -1,2059416 | 0,00089494 |  |  |  |  |
| *UCP2* | -1,9208559 | 1,42E-12 |  |  |  |  |
| *KLF11* | -1,7359031 | 4,51E-08 |  |  |  |  |
| *BNIP3* | -1,0961608 | 1,08E-05 |  |  |  |  |
| *LRRC71* | -2,3962284 | 1,15E-15 |  |  |  |  |
| *PDE1B* | -2,4485181 | 4,05E-16 |  |  |  |  |
| *C28H10orf10* | -1,878327 | 9,52E-09 |  |  |  |  |
| *PFKFB3* | -1,4668234 | 3,26E-08 |  |  |  |  |
| *VWF* | -1,4073317 | 2,50E-06 |  |  |  |  |
| *FOSL2* | -2,0497268 | 1,57E-21 |  |  |  |  |
| *PDE2A* | -2,6688519 | 2,80E-21 |  |  |  |  |
| *MLXIP* | -2,1545799 | 7,68E-23 |  |  |  |  |
| *SNAI1* | -2,0529245 | 1,62E-07 |  |  |  |  |
| *PPP5C* | -2,7110287 | 1,02E-18 |  |  |  |  |
| *ICAM3* | -1,5217715 | 1,89E-11 |  |  |  |  |
| *VEGFA* | -1,8216509 | 1,01E-07 |  |  |  |  |
| *BTNL9* | -2,2447549 | 9,90E-30 |  |  |  |  |
| *TMCC1* | -1,8449311 | 1,07E-16 |  |  |  |  |
| *NFKBIA* | -1,5300337 | 2,78E-07 |  |  |  |  |
| *SCD* | 7,90975952 | 1,45E-264 |  |  |  |  |
| *POSTN* | 5,94603303 | 1,49E-73 |  |  |  |  |
| *PRG4* | 5,57505018 | 6,44E-71 |  |  |  |  |
| *TF* | 5,45068641 | 2,30E-70 |  |  |  |  |
| *IGSF10* | 3,65071197 | 1,13E-54 |  |  |  |  |
| *FSTL1* | 2,96997999 | 9,93E-46 |  |  |  |  |
| *FMOD* | 3,47878206 | 1,22E-45 |  |  |  |  |
| *SERPINF1* | 4,37323366 | 3,60E-45 |  |  |  |  |
| *THY1* | 3,38010933 | 6,37E-44 |  |  |  |  |
| *CPA3* | 4,719894 | 3,20E-40 |  |  |  |  |
| *EFEMP1* | 3,08563166 | 3,04E-37 |  |  |  |  |
| *FBN1* | 2,60325068 | 4,60E-35 |  |  |  |  |
| *MFAP5* | 3,29776642 | 1,99E-34 |  |  |  |  |
| *ECM1* | 2,90811881 | 8,49E-33 |  |  |  |  |
| *CST3* | 3,10470547 | 2,03E-32 |  |  |  |  |
| *SPARC* | 2,86877659 | 8,20E-30 |  |  |  |  |
| *PCOLCE2* | 2,59259693 | 5,90E-28 |  |  |  |  |
| *SFRP2* | 3,36354579 | 3,77E-27 |  |  |  |  |
| *PDGFRA* | 2,36691778 | 4,08E-27 |  |  |  |  |
| *GLT8D2* | 3,47775741 | 4,17E-27 |  |  |  |  |
| *CIDEC* | 2,32638929 | 8,12E-27 |  |  |  |  |
| *LOX* | 2,45881994 | 3,10E-25 |  |  |  |  |
| *CCDC80* | 2,66502053 | 5,49E-25 |  |  |  |  |
| *KIT* | 3,03039954 | 1,86E-24 |  |  |  |  |
| *SERPING1* | 2,25838662 | 2,44E-24 |  |  |  |  |
| *COL1A2* | 3,08483822 | 1,96E-23 |  |  |  |  |
| *TMEM254* | 3,06362443 | 2,36E-23 |  |  |  |  |
| *SEMA3C* | 2,79261152 | 2,89E-23 |  |  |  |  |
| *TPSB2* | 5,17281174 | 3,43E-23 |  |  |  |  |
| *FN1* | 3,79623053 | 4,64E-23 |  |  |  |  |
| *OLFML1* | 2,16762571 | 3,43E-22 |  |  |  |  |
| *CCL21* | 4,65218492 | 7,88E-22 |  |  |  |  |
| *IL13RA1* | 2,23288807 | 1,15E-21 |  |  |  |  |
| *CYBRD1* | 2,26096274 | 2,09E-21 |  |  |  |  |
| *A2M* | 2,73673025 | 5,00E-21 |  |  |  |  |
| *NTRK2* | 2,26170583 | 1,06E-20 |  |  |  |  |
| *MGP* | 2,34623334 | 1,63E-20 |  |  |  |  |
| *RETSAT* | 2,26105628 | 2,44E-20 |  |  |  |  |
| *FGL2* | 2,76266357 | 2,80E-20 |  |  |  |  |
| *RNASE4* | 2,1944867 | 3,40E-20 |  |  |  |  |
| *ADAMTS2* | 2,45418853 | 3,37E-20 |  |  |  |  |
| *ENPP1* | 2,25884887 | 4,25E-20 |  |  |  |  |
| *LYVE1* | 2,83664079 | 6,65E-20 |  |  |  |  |
| *CHI3L1* | 2,93484611 | 6,70E-20 |  |  |  |  |
| *CLU* | 2,89396124 | 6,83E-20 |  |  |  |  |
| *ENTPD2* | 3,56844083 | 1,19E-19 |  |  |  |  |
| *CD164* | 2,72424573 | 2,19E-19 |  |  |  |  |
| *C1S* | 2,84983642 | 2,48E-19 |  |  |  |  |
| *ITM2B* | 2,38713014 | 4,48E-19 |  |  |  |  |
| *SERPINH1* | 2,11712696 | 1,19E-18 |  |  |  |  |
| *SDC2* | 2,56820977 | 1,54E-18 |  |  |  |  |
| *C1R* | 2,01770108 | 3,06E-18 |  |  |  |  |
| *CRYZ* | 1,98082207 | 1,05E-17 |  |  |  |  |
| *GJA1* | 2,09180099 | 1,19E-17 |  |  |  |  |
| *C3* | 2,37597612 | 4,42E-17 |  |  |  |  |
| *FKBP7* | 2,69283003 | 5,94E-17 |  |  |  |  |
| *LRRN4CL* | 2,82304088 | 1,04E-16 |  |  |  |  |
| *ISLR* | 2,68217954 | 1,32E-16 |  |  |  |  |
| *IDH1* | 1,94143302 | 1,98E-16 |  |  |  |  |
| *XPNPEP2* | 2,31327496 | 5,86E-16 |  |  |  |  |
| *FOLH1B* | 2,44623969 | 6,38E-16 |  |  |  |  |
| *C2* | 2,41251835 | 7,02E-16 |  |  |  |  |
| *TWSG1* | 1,9130765 | 1,42E-15 |  |  |  |  |
| *SLC25A34* | 2,24236019 | 3,23E-15 |  |  |  |  |
| *NT5E* | 2,42899638 | 3,30E-15 |  |  |  |  |
| *PLA2G7* | 2,20639868 | 3,66E-15 |  |  |  |  |
| *NFU1* | 1,93025288 | 3,96E-15 |  |  |  |  |
| *ANXA8L1* | 2,26519525 | 4,06E-15 |  |  |  |  |
| *CTSV* | 2,20287508 | 7,93E-15 |  |  |  |  |
| *ACKR3* | 2,25504406 | 8,38E-15 |  |  |  |  |
| *GSTA4* | 2,03331864 | 1,03E-14 |  |  |  |  |
| *SUOX* | 2,45438668 | 1,74E-14 |  |  |  |  |
| *EPHX1* | 1,75697299 | 1,87E-14 |  |  |  |  |
| *ACAT1* | 1,52191003 | 1,96E-14 |  |  |  |  |
| *FUCA2* | 2,57623708 | 2,28E-14 |  |  |  |  |
| *KLK7* | 3,77895278 | 3,60E-14 |  |  |  |  |
| *TMED3* | 2,4423685 | 4,53E-14 |  |  |  |  |
| *ANXA2* | 1,84954608 | 6,27E-14 |  |  |  |  |
| *FAP* | 1,83480119 | 2,20E-13 |  |  |  |  |
| *CPXM1* | 1,96273619 | 5,80E-13 |  |  |  |  |
| *C1QTNF1* | 1,82157266 | 6,98E-13 |  |  |  |  |
| *IDS* | 2,15668932 | 7,40E-13 |  |  |  |  |
| *CD52* | 2,62330719 | 7,98E-13 |  |  |  |  |
| *DSE* | 1,76738543 | 8,82E-13 |  |  |  |  |
| *ANGPTL1* | 2,86261822 | 8,87E-13 |  |  |  |  |
| *MAL2* | 2,3544255 | 8,93E-13 |  |  |  |  |
| *UQCRB* | 2,17322605 | 1,85E-12 |  |  |  |  |
| *DAD1* | 1,85792029 | 1,87E-12 |  |  |  |  |
| *PCOLCE* | 1,85411961 | 2,07E-12 |  |  |  |  |
| *HIST2H2BE* | 1,48069511 | 2,23E-12 |  |  |  |  |
| *ATP5A1* | 1,49662488 | 3,27E-12 |  |  |  |  |
| *CCL24* | 1,83992268 | 4,32E-12 |  |  |  |  |
| *ITIH5* | 1,63385317 | 4,49E-12 |  |  |  |  |
| *MGST3* | 2,1296191 | 4,65E-12 |  |  |  |  |
| *FBLN1* | 1,87176983 | 5,27E-12 |  |  |  |  |
| *DAB2* | 1,75773465 | 5,37E-12 |  |  |  |  |
| *LAMP2* | 1,73301147 | 8,14E-12 |  |  |  |  |
| *NDUFA4* | 1,59704337 | 1,04E-11 |  |  |  |  |
| *CR2* | 2,48831278 | 1,08E-11 |  |  |  |  |
| *B3GNT9* | 2,2526533 | 1,27E-11 |  |  |  |  |
| *ORMDL3* | 2,03060205 | 1,44E-11 |  |  |  |  |
| *REEP5* | 1,62789448 | 1,47E-11 |  |  |  |  |
| *LAPTM4A* | 1,92117969 | 2,22E-11 |  |  |  |  |
| *BOLA-DRA* | 2,24737627 | 2,60E-11 |  |  |  |  |
| *PGAM1* | 1,53554182 | 4,52E-11 |  |  |  |  |
| *RBP4* | 2,12202768 | 4,77E-11 |  |  |  |  |
| *FGG* | 2,59286398 | 5,69E-11 |  |  |  |  |
| *SLC1A4* | 1,78257488 | 7,18E-11 |  |  |  |  |
| *TNFAIP6* | 2,28073417 | 7,90E-11 |  |  |  |  |
| *CTHRC1* | 2,16660736 | 8,45E-11 |  |  |  |  |
| *ABCD2* | 2,32102672 | 8,51E-11 |  |  |  |  |
| *PCYOX1* | 1,76196724 | 9,91E-11 |  |  |  |  |
| *TIMP1* | 1,99667761 | 1,08E-10 |  |  |  |  |
| *CYP2U1* | 1,74161943 | 1,17E-10 |  |  |  |  |
| *HADH* | 1,69668886 | 1,22E-10 |  |  |  |  |
| *HIBADH* | 1,62278468 | 1,75E-10 |  |  |  |  |
| *LGALS3BP* | 1,93964209 | 1,92E-10 |  |  |  |  |
| *FKBP10* | 1,66389076 | 2,30E-10 |  |  |  |  |
| *OLFML3* | 2,12209366 | 3,19E-10 |  |  |  |  |
| *QSOX1* | 1,67448532 | 4,00E-10 |  |  |  |  |
| *CREG1* | 1,69426437 | 4,37E-10 |  |  |  |  |
| *COL12A1* | 1,37354063 | 4,38E-10 |  |  |  |  |
| *ANGPTL2* | 2,07216854 | 4,72E-10 |  |  |  |  |
| *CKB* | 2,22468608 | 4,78E-10 |  |  |  |  |
| *C9H6orf120* | 1,43931062 | 5,70E-10 |  |  |  |  |
| *MASP1* | 1,94459374 | 5,97E-10 |  |  |  |  |
| *HSD17B10* | 1,67160236 | 6,35E-10 |  |  |  |  |
| *DDHD2* | 1,45156556 | 7,60E-10 |  |  |  |  |
| *PEBP1* | 1,56651364 | 7,84E-10 |  |  |  |  |
| *MEDAG* | 1,26850304 | 8,99E-10 |  |  |  |  |
| *LOC510860* | 2,21672738 | 1,07E-09 |  |  |  |  |
| *TMCO1* | 1,68841909 | 1,11E-09 |  |  |  |  |
| *RAC2* | 1,91811843 | 1,22E-09 |  |  |  |  |
| *DBT* | 1,8901787 | 1,28E-09 |  |  |  |  |
| *SGCE* | 2,03027369 | 1,57E-09 |  |  |  |  |
| *MFSD1* | 1,64596387 | 1,87E-09 |  |  |  |  |
| *FRMD6* | 1,28145305 | 1,94E-09 |  |  |  |  |
| *DKK2* | 2,39804809 | 2,04E-09 |  |  |  |  |
| *COX6A1* | 1,48051549 | 2,51E-09 |  |  |  |  |
| *SPSB1* | 1,45499489 | 2,64E-09 |  |  |  |  |
| *EMC3* | 1,42723765 | 2,72E-09 |  |  |  |  |
| *CLMP* | 1,86713 | 3,01E-09 |  |  |  |  |
| *TSPAN3* | 1,70334122 | 3,54E-09 |  |  |  |  |
| *GHITM* | 1,38126368 | 3,61E-09 |  |  |  |  |
| *IGFBP4* | 1,47861894 | 4,21E-09 |  |  |  |  |
| *DSEL* | 1,47582307 | 4,44E-09 |  |  |  |  |
| *TGFBI* | 1,78448435 | 4,61E-09 |  |  |  |  |
| *ADAMTSL4* | 1,30966305 | 4,65E-09 |  |  |  |  |
| *sept-15* | 1,59040687 | 5,30E-09 |  |  |  |  |
| *C3AR1* | 2,14111901 | 5,83E-09 |  |  |  |  |
| *PRKAR2B* | 1,58505104 | 7,26E-09 |  |  |  |  |
| *SLC9A9* | 1,54472647 | 7,69E-09 |  |  |  |  |
| *RBPJ* | 1,28962037 | 7,68E-09 |  |  |  |  |
| *CD63* | 1,50960225 | 8,20E-09 |  |  |  |  |
| *FXYD1* | 1,38364038 | 8,29E-09 |  |  |  |  |
| *F13A1* | 1,77140977 | 8,43E-09 |  |  |  |  |
| *MIF* | 2,13992255 | 9,98E-09 |  |  |  |  |
| *ATP5B* | 1,47451792 | 1,01E-08 |  |  |  |  |
| *ATP5J2* | 1,71834361 | 1,01E-08 |  |  |  |  |
| *ALDH7A1* | 1,18545648 | 1,09E-08 |  |  |  |  |
| *DEGS1* | 1,42798913 | 1,11E-08 |  |  |  |  |
| *TECR* | 1,84393058 | 1,13E-08 |  |  |  |  |
| *DHRS7* | 1,54720879 | 1,48E-08 |  |  |  |  |
| *HADHB* | 1,25754691 | 1,58E-08 |  |  |  |  |
| *GPX7* | 1,74775123 | 1,75E-08 |  |  |  |  |
| *TH* | 2,15176481 | 1,97E-08 |  |  |  |  |
| *LOC504773* | 1,95352589 | 2,03E-08 |  |  |  |  |
| *CD99* | 1,46183995 | 2,17E-08 |  |  |  |  |
| *LANCL1* | 1,42837038 | 2,57E-08 |  |  |  |  |
| *ARL6IP1* | 1,53394732 | 2,76E-08 |  |  |  |  |
| *LOC507581* | 1,77683035 | 2,98E-08 |  |  |  |  |
| *TMEM50A* | 1,50535523 | 2,99E-08 |  |  |  |  |
| *SULT1A1* | 1,43509837 | 3,13E-08 |  |  |  |  |
| *S100A10* | 1,77149285 | 3,14E-08 |  |  |  |  |
| *CTSK* | 1,44704013 | 3,94E-08 |  |  |  |  |
| *ACKR2* | 2,6267123 | 4,90E-08 |  |  |  |  |
| *BHLHE40* | 1,61080315 | 4,99E-08 |  |  |  |  |
| *RPN2* | 1,75017213 | 4,99E-08 |  |  |  |  |
| *TLR5* | 2,14267342 | 5,82E-08 |  |  |  |  |
| *GSN* | 1,90899709 | 5,92E-08 |  |  |  |  |
| *GFPT1* | 1,17117776 | 6,03E-08 |  |  |  |  |
| *HP* | 2,59178128 | 6,12E-08 |  |  |  |  |
| *OSTC* | 1,4475514 | 6,15E-08 |  |  |  |  |
| *COL1A1* | 2,04431762 | 6,47E-08 |  |  |  |  |
| *PLP2* | 1,64350025 | 6,60E-08 |  |  |  |  |
| *NR3C1* | 1,20820686 | 6,91E-08 |  |  |  |  |
| *HSD17B4* | 1,0384439 | 7,15E-08 |  |  |  |  |
| *TLR4* | 1,40887457 | 7,71E-08 |  |  |  |  |
| *ANXA1* | 1,37457824 | 8,47E-08 |  |  |  |  |
| *DAG1* | 1,72628888 | 1,02E-07 |  |  |  |  |
| *MIR6516* | 2,4442154 | 1,07E-07 |  |  |  |  |
| *DCBLD2* | 1,24751747 | 1,12E-07 |  |  |  |  |
| *CPNE3* | 1,40310103 | 1,29E-07 |  |  |  |  |
| *OSR2* | 1,94762985 | 1,42E-07 |  |  |  |  |
| *PCDH18* | 1,58320188 | 1,45E-07 |  |  |  |  |
| *SUMF1* | 1,63861507 | 1,46E-07 |  |  |  |  |
| *SIRPA* | 1,74248403 | 1,47E-07 |  |  |  |  |
| *NUCB1* | 1,166175 | 1,60E-07 |  |  |  |  |
| *TPI1* | 1,40923146 | 1,66E-07 |  |  |  |  |
| *SCARA5* | 1,11659937 | 1,69E-07 |  |  |  |  |
| *SLC31A1* | 1,38958586 | 1,74E-07 |  |  |  |  |
| *CS* | 1,64186375 | 1,78E-07 |  |  |  |  |
| *ALOX5* | 1,39683989 | 1,80E-07 |  |  |  |  |
| *TXNDC12* | 1,63871857 | 1,86E-07 |  |  |  |  |
| *PSMA1* | 1,23683043 | 1,98E-07 |  |  |  |  |
| *S100B* | 1,51124051 | 2,00E-07 |  |  |  |  |
| *MARVELD1* | 1,63447218 | 2,07E-07 |  |  |  |  |
| *GM2A* | 2,16353265 | 2,13E-07 |  |  |  |  |
| *SC5D* | 1,42206326 | 2,28E-07 |  |  |  |  |
| *TMED10* | 1,27361268 | 2,39E-07 |  |  |  |  |
| *PTGR2* | 1,45547102 | 2,43E-07 |  |  |  |  |
| *CXCL14* | 1,75716775 | 2,58E-07 |  |  |  |  |
| *TMED7* | 1,57527988 | 2,94E-07 |  |  |  |  |
| *NPC2* | 1,12476804 | 2,93E-07 |  |  |  |  |
| *MFAP2* | 1,44580964 | 3,25E-07 |  |  |  |  |
| *MFAP4* | 2,04403625 | 3,56E-07 |  |  |  |  |
| *SUCLG1* | 1,15971384 | 3,78E-07 |  |  |  |  |
| *LOC615809* | 1,58256038 | 4,06E-07 |  |  |  |  |
| *TSPAN6* | 1,6633646 | 4,26E-07 |  |  |  |  |
| *NQO1* | 1,68453755 | 4,44E-07 |  |  |  |  |
| *FAM180B* | 2,11045989 | 4,46E-07 |  |  |  |  |
| *NDUFS7* | 1,53990967 | 4,81E-07 |  |  |  |  |
| *SEC22B* | 1,28052966 | 4,82E-07 |  |  |  |  |
| *STT3A* | 1,43993074 | 4,85E-07 |  |  |  |  |
| *CYB5R3* | 1,36161353 | 5,26E-07 |  |  |  |  |
| *ATP5O* | 1,11074172 | 5,42E-07 |  |  |  |  |
| *S1PR2* | 1,7554998 | 5,65E-07 |  |  |  |  |
| *ASPH* | 1,3310946 | 6,10E-07 |  |  |  |  |
| *PEX19* | 1,20011544 | 6,92E-07 |  |  |  |  |
| *GPD2* | 1,12392491 | 7,37E-07 |  |  |  |  |
| *ENO1* | 1,22954348 | 7,69E-07 |  |  |  |  |
| *LOC509055* | 1,99639402 | 8,02E-07 |  |  |  |  |
| *BRB* | 1,81567487 | 8,48E-07 |  |  |  |  |
| *LGALS1* | 1,31869273 | 8,48E-07 |  |  |  |  |
| *WLS* | 1,23252948 | 8,70E-07 |  |  |  |  |
| *SMIM19* | 1,02034515 | 9,26E-07 |  |  |  |  |
| *TM9SF2* | 1,63434247 | 9,30E-07 |  |  |  |  |
| *PDGFD* | 1,24014886 | 9,66E-07 |  |  |  |  |
| *WDR74* | 1,78920633 | 1,00E-06 |  |  |  |  |
| *ACKR4* | 2,14272348 | 1,03E-06 |  |  |  |  |
| *BTK* | 1,76802306 | 1,03E-06 |  |  |  |  |
| *IGJ* | 1,68932459 | 1,04E-06 |  |  |  |  |
| *CFB* | 2,02396753 | 1,06E-06 |  |  |  |  |
| *PRDX6* | 1,18691686 | 1,09E-06 |  |  |  |  |
| *FDX1* | 1,56873432 | 1,11E-06 |  |  |  |  |
| *PRDX4* | 1,48950396 | 1,11E-06 |  |  |  |  |
| *RAB5A* | 1,23711949 | 1,13E-06 |  |  |  |  |
| *ANXA7* | 1,13928415 | 1,14E-06 |  |  |  |  |
| *NDUFB8* | 1,27389107 | 1,16E-06 |  |  |  |  |
| *COPB2* | 1,12654373 | 1,17E-06 |  |  |  |  |
| *YIPF5* | 1,12331039 | 1,21E-06 |  |  |  |  |
| *CANX* | 1,2071453 | 1,23E-06 |  |  |  |  |
| *GBE1* | 1,20906861 | 1,27E-06 |  |  |  |  |
| *TCTA* | 1,64152283 | 1,28E-06 |  |  |  |  |
| *RNASE6* | 1,48658449 | 1,31E-06 |  |  |  |  |
| *HSPA9* | 1,32845398 | 1,37E-06 |  |  |  |  |
| *AHR* | 1,38860569 | 1,46E-06 |  |  |  |  |
| *METRNL* | 1,60753742 | 1,49E-06 |  |  |  |  |
| *EFNA5* | 1,33138448 | 1,56E-06 |  |  |  |  |
| *NDUFB3* | 1,58734393 | 1,60E-06 |  |  |  |  |
| *TUSC5* | 1,61107272 | 1,61E-06 |  |  |  |  |
| *PRELP* | 1,43742598 | 1,67E-06 |  |  |  |  |
| *PIGS* | 1,14315879 | 1,81E-06 |  |  |  |  |
| *ITGBL1* | 1,94661183 | 1,88E-06 |  |  |  |  |
| *TXN2* | 1,26134359 | 1,92E-06 |  |  |  |  |
| *EMP3* | 1,84973095 | 2,04E-06 |  |  |  |  |
| *CTSS* | 1,80246705 | 2,09E-06 |  |  |  |  |
| *UQCR10* | 1,48738713 | 2,44E-06 |  |  |  |  |
| *ATIC* | 1,12022545 | 2,50E-06 |  |  |  |  |
| *F2RL2* | 1,62654203 | 2,69E-06 |  |  |  |  |
| *SGPP1* | 1,59718789 | 2,71E-06 |  |  |  |  |
| *GLUD1* | 0,93252226 | 3,29E-06 |  |  |  |  |
| *GDA* | 1,34417467 | 3,29E-06 |  |  |  |  |
| *GAPDH* | 1,22532891 | 3,32E-06 |  |  |  |  |
| *MGC148714* | 1,31283975 | 3,51E-06 |  |  |  |  |
| *C4A* | 1,2567316 | 3,72E-06 |  |  |  |  |
| *TKT* | 0,90834086 | 3,89E-06 |  |  |  |  |
| *C5AR2* | 1,74587464 | 4,04E-06 |  |  |  |  |
| *MRPL49* | 1,32864319 | 4,08E-06 |  |  |  |  |
| *HPGD* | 2,15956286 | 4,13E-06 |  |  |  |  |
| *CD74* | 1,67593956 | 4,44E-06 |  |  |  |  |
| *ALG10* | 1,66017733 | 4,60E-06 |  |  |  |  |
| *GPR1* | 1,54643543 | 4,75E-06 |  |  |  |  |
| *GLCE* | 1,51679306 | 4,77E-06 |  |  |  |  |
| *TMEM14C* | 1,15634108 | 5,00E-06 |  |  |  |  |
| *NDUFS1* | 1,50464606 | 5,20E-06 |  |  |  |  |
| *CSGALNACT2* | 1,04485186 | 5,38E-06 |  |  |  |  |
| *MCFD2* | 1,49539819 | 5,52E-06 |  |  |  |  |
| *KITLG* | 1,28149146 | 5,81E-06 |  |  |  |  |
| *ANTXR2* | 1,05357623 | 5,87E-06 |  |  |  |  |
| *GSTP1* | 1,45036931 | 6,87E-06 |  |  |  |  |
| *SOD3* | 1,67238322 | 7,02E-06 |  |  |  |  |
| *TMEM100* | 1,8049361 | 7,22E-06 |  |  |  |  |
| *FBLN5* | 1,38673966 | 7,62E-06 |  |  |  |  |
| *LAMC1* | 1,09218239 | 7,67E-06 |  |  |  |  |
| *P4HB* | 1,38893705 | 8,35E-06 |  |  |  |  |
| *ATXN7L3B* | 1,28413426 | 8,91E-06 |  |  |  |  |
| *AOC1* | 1,92979856 | 9,53E-06 |  |  |  |  |
| *TMEM150C* | 1,25109356 | 9,97E-06 |  |  |  |  |
| *COX5B* | 1,36660563 | 1,06E-05 |  |  |  |  |
| *HSPB8* | 1,53249057 | 1,13E-05 |  |  |  |  |
| *ATP5C1* | 0,92071828 | 1,37E-05 |  |  |  |  |
| *ARHGDIB* | 1,32755445 | 1,41E-05 |  |  |  |  |
| *NID1* | 1,53471432 | 1,48E-05 |  |  |  |  |
| *CD44* | 1,226315 | 1,59E-05 |  |  |  |  |
| *OLFML2B* | 1,03299186 | 1,63E-05 |  |  |  |  |
| *SRGN* | 1,5661204 | 1,64E-05 |  |  |  |  |
| *GSTM3* | 2,20663244 | 1,99E-05 |  |  |  |  |
| *NOV* | 1,95332717 | 2,07E-05 |  |  |  |  |
| *ALDH1L2* | 1,14109419 | 2,10E-05 |  |  |  |  |
| *SEC23A* | 0,93575519 | 2,27E-05 |  |  |  |  |
| *TST* | 1,39585712 | 2,45E-05 |  |  |  |  |
| *LTBP1* | 1,40007637 | 3,13E-05 |  |  |  |  |
| *HS3ST1* | 1,71092089 | 3,47E-05 |  |  |  |  |
| *LPAR1* | 1,30164825 | 4,13E-05 |  |  |  |  |
| *SLC1A3* | 0,96284691 | 4,17E-05 |  |  |  |  |
| *SEPP1* | 1,48081623 | 4,34E-05 |  |  |  |  |
| *sept-10* | 1,02680546 | 4,45E-05 |  |  |  |  |
| *ADGRD1* | 1,21051955 | 4,46E-05 |  |  |  |  |
| *SLC1A5* | 1,26863732 | 4,86E-05 |  |  |  |  |
| *KRCC1* | 0,98592988 | 5,14E-05 |  |  |  |  |
| *ANXA6* | 0,96355604 | 5,46E-05 |  |  |  |  |
| *SSR3* | 1,09084017 | 5,95E-05 |  |  |  |  |
| *ERP29* | 0,98061948 | 5,99E-05 |  |  |  |  |
| *THBD* | 1,59332654 | 6,08E-05 |  |  |  |  |
| *ERLEC1* | 1,27261134 | 6,08E-05 |  |  |  |  |
| *C4H7orf73* | 1,0201598 | 6,64E-05 |  |  |  |  |
| *NPTN* | 1,12215249 | 7,39E-05 |  |  |  |  |
| *PDK1* | 1,08041113 | 7,89E-05 |  |  |  |  |
| *CD200* | 1,41235444 | 9,73E-05 |  |  |  |  |
| *SFXN3* | 1,09964276 | 0,00013218 |  |  |  |  |
| *COX14* | 1,2062749 | 0,00014088 |  |  |  |  |
| *PRICKLE1* | 0,84759643 | 0,00014703 |  |  |  |  |
| *SPTLC1* | 1,31920178 | 0,00015025 |  |  |  |  |
| *IGF1* | 1,34529249 | 0,00016539 |  |  |  |  |
| *COX7A2L* | 0,87902167 | 0,00016878 |  |  |  |  |
| *AQPEP* | 1,12240894 | 0,0001809 |  |  |  |  |
| *IL1RL1* | 1,41733072 | 0,00018216 |  |  |  |  |
| *FRMD4B* | 0,97231179 | 0,00019023 |  |  |  |  |
| *LHFP* | 1,1214123 | 0,00019172 |  |  |  |  |
| *ELL2* | 0,79271395 | 0,00020699 |  |  |  |  |
| *EMILIN2* | 1,08167423 | 0,00020797 |  |  |  |  |
| *MORF4L2* | 0,74015732 | 0,00021823 |  |  |  |  |
| *MAN1A1* | 0,9675164 | 0,00022294 |  |  |  |  |
| *SNX7* | 0,97340765 | 0,00023063 |  |  |  |  |
| *ARFGAP3* | 0,75374393 | 0,00023343 |  |  |  |  |
| *ADD3* | 1,00943094 | 0,00025804 |  |  |  |  |
| *DDR2* | 0,88758044 | 0,00035249 |  |  |  |  |
| *ABCD3* | 0,94963799 | 0,00044977 |  |  |  |  |
| *DOCK11* | 0,76955367 | 0,00048618 |  |  |  |  |
| *PLOD2* | 1,11504364 | 0,00049415 |  |  |  |  |
| *FAM129A* | 0,8800469 | 0,00053715 |  |  |  |  |
| *PRKD3* | 0,74223123 | 0,0006271 |  |  |  |  |
| *UGP2* | 0,74548619 | 0,00068017 |  |  |  |  |
| *SLC2A1* | 1,71057206 | 0,00069985 |  |  |  |  |
| *CDK14* | 0,77055537 | 0,00072566 |  |  |  |  |
| *SFXN1* | 0,86058236 | 0,0007349 |  |  |  |  |
| *BMPR1A* | 1,17083213 | 0,00081658 |  |  |  |  |
| *GFPT2* | 0,832996 | 0,00083946 |  |  |  |  |
| *CAPZA1* | 0,83825621 | 0,00090832 |  |  |  |  |
| *ATP2B1* | 0,84204501 | 0,00096855 |  |  |  |  |
| *MMADHC* | 0,74264314 | 0,00104223 |  |  |  |  |
| *GPI* | 0,74673577 | 0,00104864 |  |  |  |  |
| *ZYG11B* | 0,86372995 | 0,00112066 |  |  |  |  |
| *SLC4A4* | 0,85700092 | 0,00114939 |  |  |  |  |
| *NDFIP2* | 0,77579947 | 0,00115322 |  |  |  |  |
| *FYN* | 0,84154856 | 0,00121568 |  |  |  |  |
| *TPST1* | 0,88766531 | 0,00129325 |  |  |  |  |
| *TMEM135* | 0,95346652 | 0,00136722 |  |  |  |  |
| *RALGPS2* | 0,94690322 | 0,00150351 |  |  |  |  |
| *PTGIS* | 0,74439361 | 0,00163292 |  |  |  |  |
| *SLC25A16* | 0,96079253 | 0,00194116 |  |  |  |  |
| *CLCC1* | 0,82204248 | 0,00220683 |  |  |  |  |
| *UHRF1BP1L* | 0,7693452 | 0,00222163 |  |  |  |  |
| *PLXDC2* | 0,60216515 | 0,00225972 |  |  |  |  |
| *CIDEA* | 2,74977385 | 1,02E-13 |  |  |  |  |
| *ACACA* | 2,54074689 | 1,13E-19 |  |  |  |  |
| *GPAM* | 4,0063215 | 1,87E-33 |  |  |  |  |
| *PCK1* | 4,32414305 | 6,51E-30 |  |  |  |  |
| *FASN* | 3,86163631 | 3,00E-35 |  |  |  |  |
| *GSTT2* | 4,2932865 | 1,03E-29 |  |  |  |  |
| *PRNP* | 2,64081547 | 1,38E-27 |  |  |  |  |
| *TDH* | 3,2730577 | 3,43E-13 |  |  |  |  |
| *GPD1* | 2,20578513 | 3,76E-28 |  |  |  |  |
| *MID1IP1* | 2,96235861 | 2,67E-24 |  |  |  |  |
| *ASPN* | 3,94301438 | 3,64E-73 |  |  |  |  |
| *MGST1* | 4,50337539 | 8,67E-71 |  |  |  |  |
| *PCYT2* | 1,2459995 | 2,10E-06 |  |  |  |  |
| *ACSL1* | 2,86265519 | 8,68E-22 |  |  |  |  |
| *KRTAP11-1* | 3,88578217 | 7,29E-09 |  |  |  |  |
| *THRSP* | 5,00519928 | 2,13E-32 |  |  |  |  |
| *ELOVL6* | 4,78300397 | 2,97E-57 |  |  |  |  |
| *GPT2* | 4,08978615 | 1,30E-49 |  |  |  |  |
| *INSIG1* | 2,86510164 | 1,56E-11 |  |  |  |  |
| *QPRT* | 1,94180894 | 1,88E-08 |  |  |  |  |
| *ADIG* | 2,76344666 | 8,02E-20 |  |  |  |  |
| *AGPAT2* | 3,58219657 | 2,94E-45 |  |  |  |  |
| *MDFI* | 1,92116681 | 2,65E-09 |  |  |  |  |
| *ACER3* | 3,04443489 | 4,82E-36 |  |  |  |  |
| *DBI* | 2,6599386 | 2,15E-19 |  |  |  |  |
| *FKBP14* | 2,28000077 | 3,83E-13 |  |  |  |  |
| *CYB5A* | 1,49362233 | 1,04E-12 |  |  |  |  |
| *HMGCS1* | 2,28954151 | 8,42E-09 |  |  |  |  |
| *SFXN2* | 1,77521679 | 9,86E-09 |  |  |  |  |
| *MMP15* | 2,17873073 | 5,57E-13 |  |  |  |  |
| *ACLY* | 2,14574824 | 7,69E-14 |  |  |  |  |
| *HSD17B12* | 3,0712998 | 5,67E-24 |  |  |  |  |
| *LDHB* | 2,21041087 | 6,29E-22 |  |  |  |  |
| *SLC25A4* | 2,21062773 | 2,08E-15 |  |  |  |  |
| *GLYCTK* | 1,40818669 | 4,43E-06 |  |  |  |  |
| *AR* | 1,89852098 | 6,34E-08 |  |  |  |  |
| *ELOVL5* | 2,65768038 | 3,79E-19 |  |  |  |  |
| *MSRB1* | 2,69726389 | 2,55E-27 |  |  |  |  |
| *B3GALNT1* | 2,69444769 | 3,26E-17 |  |  |  |  |
| *AQP7* | 2,11493961 | 2,50E-10 |  |  |  |  |
| *BSG* | 2,4509261 | 1,75E-26 |  |  |  |  |
| *EBP* | 2,64736566 | 8,82E-15 |  |  |  |  |
| *DECR1* | 2,5618454 | 1,33E-16 |  |  |  |  |
| *SBDS* | 1,95941573 | 4,05E-15 |  |  |  |  |
| *LRRC17* | 3,5793053 | 1,01E-35 |  |  |  |  |
| *VCAN* | 2,21885092 | 2,07E-10 |  |  |  |  |
| *CCDC3* | 3,1937928 | 6,62E-31 |  |  |  |  |
| *FDFT1* | 1,20820416 | 3,46E-07 |  |  |  |  |
| *MDH1* | 1,80670853 | 1,83E-11 |  |  |  |  |
| *VCAM1* | 2,43486047 | 2,61E-10 |  |  |  |  |
| *LEP* | 3,34250945 | 8,78E-21 |  |  |  |  |
| *BCAT2* | 1,82484065 | 3,94E-13 |  |  |  |  |
| *SFRP4* | 3,72974189 | 9,27E-30 |  |  |  |  |
| *KLHL31* | 3,20129765 | 8,03E-16 |  |  |  |  |
| *ACAT2* | 2,58555867 | 2,18E-12 |  |  |  |  |
| *PI16* | 3,49584759 | 1,73E-14 |  |  |  |  |
| *BGN* | 2,0807178 | 9,62E-10 |  |  |  |  |
| *S100G* | 3,41906086 | 3,08E-22 |  |  |  |  |
| *LOXL1* | 2,25001185 | 3,73E-10 |  |  |  |  |
| *MPC1* | 2,24381408 | 7,27E-20 |  |  |  |  |
| *HMCN1* | 1,40888073 | 2,53E-07 |  |  |  |  |
| *LUM* | 2,60284173 | 2,66E-13 |  |  |  |  |
| *BCHE* | 3,06603123 | 2,07E-23 |  |  |  |  |
| *FAM84A* | 2,06539474 | 4,97E-06 |  |  |  |  |
| *PTPLB* | 2,07957723 | 1,71E-07 |  |  |  |  |
| *C18H19orf12* | 1,25304679 | 1,19E-07 |  |  |  |  |
| *QPCT* | 3,34569234 | 1,26E-18 |  |  |  |  |
| *EPDR1* | 2,84714756 | 3,20E-21 |  |  |  |  |
| *P4HA2* | 1,18205166 | 6,11E-06 |  |  |  |  |
| *WISP2* | 2,41674903 | 3,08E-09 |  |  |  |  |
| *NCALD* | 1,59153933 | 4,76E-08 |  |  |  |  |
| *TFRC* | 2,06328151 | 3,21E-10 |  |  |  |  |
| *NID2* | 1,26830545 | 2,17E-09 |  |  |  |  |
| *CCDC88A* | 1,08588032 | 1,60E-05 |  |  |  |  |
| *ATP5G3* | 1,5232048 | 1,30E-12 |  |  |  |  |
| *RCN1* | 1,29176828 | 2,12E-08 |  |  |  |  |
| *NIPSNAP1* | 1,90933571 | 4,09E-07 |  |  |  |  |
| *PYCR1* | 2,06728535 | 1,85E-06 |  |  |  |  |
| *MLEC* | 1,33500093 | 2,80E-07 |  |  |  |  |
| *FITM2* | 2,13274035 | 8,20E-12 |  |  |  |  |
| *SLC16A1* | 2,87240988 | 2,09E-32 |  |  |  |  |
| *FGF7* | 1,73037071 | 1,25E-07 |  |  |  |  |
| *ACADSB* | 2,90811317 | 2,10E-27 |  |  |  |  |
| *CALU* | 1,62391128 | 1,76E-07 |  |  |  |  |
| *TALDO1* | 1,67947912 | 4,64E-13 |  |  |  |  |
