# Supplementary material for: Impact of the severity of negative energy balance on gene expression in the subcutaneous adipose tissue of periparturient primiparous Holstein dairy cows: Identification of potential novel metabolic signals for the reproductive system
Source: PLoS One. 2019 Sep 26;14(9):e0222954. doi: 10.1371/journal.pone.0222954 (PMC6763198; doi:10.1371/journal.pone.0222954)
Supplement: S5 Table — (DOCX) [file pone.0222954.s010.docx]

| **S5 Table: Genes differentially expressed in MNEB animals at 16 WKPP as compared to 1WKPP** | | | | | | |
| --- | --- | --- | --- | --- | --- | --- |
| name | log2FoldChange_exons | pvalue_exons | |  |  |  |
| *SCD* | -2,4721789 | 5,91E-12 |  |  |  |  |
| *FMOD* | -1,3403484 | 3,81E-08 |  |  |  |  |
| *ANXA8L1* | -1,8616001 | 5,03E-08 |  |  |  |  |
| *RERG* | -1,5363715 | 1,19E-07 |  |  |  |  |
| *VASN* | -1,6323792 | 1,50E-07 |  |  |  |  |
| *TXN* | -1,310072 | 2,10E-07 |  |  |  |  |
| *THY1* | -1,3267345 | 2,14E-07 |  |  |  |  |
| *GAPDH* | -1,6843031 | 3,92E-07 |  |  |  |  |
| *ANXA7* | -1,0848692 | 4,28E-07 |  |  |  |  |
| *HIST2H2BE* | -1,1749777 | 6,36E-07 |  |  |  |  |
| *PCOLCE2* | -1,2102953 | 9,96E-07 |  |  |  |  |
| *PGAM1* | -1,3081375 | 1,44E-06 |  |  |  |  |
| *HSP90AA1* | -1,0289778 | 1,85E-06 |  |  |  |  |
| *SERPINE1* | -1,7024191 | 3,79E-06 |  |  |  |  |
| *PLN* | -1,652991 | 3,76E-06 |  |  |  |  |
| *BHLHE40* | -1,4071921 | 3,99E-06 |  |  |  |  |
| *SMIM19* | -1,0243787 | 4,44E-06 |  |  |  |  |
| *DSTN* | -1,2003161 | 5,98E-06 |  |  |  |  |
| *TMX4* | -1,1722074 | 8,39E-06 |  |  |  |  |
| *MGST1* | -1,109546 | 1,38E-05 |  |  |  |  |
| *HIST1H1C* | -1,1532057 | 1,88E-05 |  |  |  |  |
| *DSE* | -1,0417605 | 2,02E-05 |  |  |  |  |
| *SSR4* | -1,0592946 | 2,56E-05 |  |  |  |  |
| *IGFBP6* | -1,4082185 | 2,73E-05 |  |  |  |  |
| *LGALS1* | -1,0076322 | 3,18E-05 |  |  |  |  |
| *PDK1* | -1,0456489 | 3,78E-05 |  |  |  |  |
| *C9H6orf120* | -0,9626186 | 4,40E-05 |  |  |  |  |
| *LBH* | -1,0215806 | 4,94E-05 |  |  |  |  |
| *ANXA5* | -0,7762092 | 5,10E-05 |  |  |  |  |
| *EID1* | -0,9183749 | 5,41E-05 |  |  |  |  |
| *H3F3B* | -1,0219586 | 5,80E-05 |  |  |  |  |
| *ABCA9* | 1,08509726 | 1,36E-08 |  |  |  |  |
| *ICAM3* | 1,10539281 | 1,73E-06 |  |  |  |  |
| *CTNS* | 1,15336007 | 4,84E-06 |  |  |  |  |
| *ARHGEF28* | 1,13514965 | 1,03E-05 |  |  |  |  |
| *ZSWIM8* | 0,83661018 | 3,19E-05 |  |  |  |  |
| *NOTCH4* | 0,89240481 | 5,07E-05 |  |  |  |  |
