# Supplementary material for: Impact of the severity of negative energy balance on gene expression in the subcutaneous adipose tissue of periparturient primiparous Holstein dairy cows: Identification of potential novel metabolic signals for the reproductive system
Source: PLoS One. 2019 Sep 26;14(9):e0222954. doi: 10.1371/journal.pone.0222954 (PMC6763198; doi:10.1371/journal.pone.0222954)
Supplement: S6 Table — (DOCX) [file pone.0222954.s011.docx]

| **S6 Table: Genes differentially expressed in SNEB animals at 1 WKPP as compared to -4WKPP** | | | | | | |
| --- | --- | --- | --- | --- | --- | --- |
| name | log2FoldChange_exons | pvalue_exons | |  |  |  |
| *PHYH* | -1,6940046 | 1,45E-20 |  |  |  |  |
| *CYP4V2* | -1,5555539 | 4,00E-16 |  |  |  |  |
| *CD163* | -1,3351777 | 4,15E-14 |  |  |  |  |
| *TXNIP* | -1,4063065 | 2,16E-12 |  |  |  |  |
| *THBS1* | -1,919452 | 4,62E-12 |  |  |  |  |
| *GALNT15* | -1,9436959 | 6,11E-12 |  |  |  |  |
| *ABCA1* | -1,3255883 | 5,88E-10 |  |  |  |  |
| *ASGR1* | -2,1367416 | 5,82E-09 |  |  |  |  |
| *ADAMTS5* | -1,7373379 | 7,95E-09 |  |  |  |  |
| *PDPN* | -1,4045372 | 1,32E-08 |  |  |  |  |
| *FABP4* | -1,0601609 | 3,33E-08 |  |  |  |  |
| *CSF1* | -1,0014033 | 4,35E-08 |  |  |  |  |
| *RGS1* | -2,0585451 | 6,42E-08 |  |  |  |  |
| *APOA1* | -1,31083 | 4,51E-07 |  |  |  |  |
| *TRIB1* | -1,1652404 | 5,96E-07 |  |  |  |  |
| *KCNJ15* | -1,8763706 | 7,89E-07 |  |  |  |  |
| *GPX3* | -1,481277 | 1,12E-06 |  |  |  |  |
| *VNN1* | -1,108838 | 1,23E-06 |  |  |  |  |
| *ANPEP* | -1,1828781 | 2,15E-06 |  |  |  |  |
| *ADRB2* | -1,2818261 | 3,27E-06 |  |  |  |  |
| *HCAR1* | -1,3365052 | 5,95E-06 |  |  |  |  |
| *CD5L* | -1,1638375 | 7,00E-06 |  |  |  |  |
| *CDKN1A* | -1,5634198 | 1,04E-05 |  |  |  |  |
| *GPR1* | -1,0652968 | 1,12E-05 |  |  |  |  |
| *INHBB* | -1,5381582 | 1,17E-05 |  |  |  |  |
| *S1PR3* | -1,5177902 | 1,20E-05 |  |  |  |  |
| *LAMB1* | -1,0671164 | 1,26E-05 |  |  |  |  |
| *IGFBP3* | -1,2154233 | 1,44E-05 |  |  |  |  |
| *FAM105A* | -1,0711775 | 2,75E-05 |  |  |  |  |
| *IL6R* | -1,0295116 | 2,87E-05 |  |  |  |  |
| *RARRES1* | -1,0777333 | 3,31E-05 |  |  |  |  |
| *C27H8orf4* | -1,494678 | 3,60E-05 |  |  |  |  |
| *APOLD1* | -1,2524841 | 3,94E-05 |  |  |  |  |
| *HEY1* | -1,3000702 | 4,49E-05 |  |  |  |  |
| *SELENBP1* | -0,8559473 | 6,26E-05 |  |  |  |  |
| *SOD2* | -0,8489717 | 6,60E-05 |  |  |  |  |
| *CDC42EP4* | -0,9523625 | 0,00013825 |  |  |  |  |
| *EIF4EBP1* | -1,0926673 | 0,00016879 |  |  |  |  |
| *PDK4* | -2,5811099 | 5,57E-17 |  |  |  |  |
| *PLXNA4* | -1,2698823 | 6,90E-06 |  |  |  |  |
| *ADM* | -2,1672675 | 6,30E-12 |  |  |  |  |
| *NRIP3* | -2,1440549 | 1,68E-11 |  |  |  |  |
| *UCP2* | -1,2494998 | 2,12E-08 |  |  |  |  |
| *KLF11* | -1,7287472 | 1,37E-11 |  |  |  |  |
| *BNIP3* | -1,703271 | 8,78E-09 |  |  |  |  |
| *LRRC71* | -1,4797161 | 1,01E-11 |  |  |  |  |
| *PDE1B* | -1,1981272 | 1,41E-06 |  |  |  |  |
| *C28H10orf10* | -1,4606211 | 0,0003265 |  |  |  |  |
| *PFKFB3* | -1,317138 | 5,07E-06 |  |  |  |  |
| *VWF* | -0,7189633 | 1,39E-05 |  |  |  |  |
| *FOSL2* | -1,2943368 | 7,10E-06 |  |  |  |  |
| *PDE2A* | -0,9664935 | 6,09E-05 |  |  |  |  |
| *MLXIP* | -0,8950111 | 9,99E-05 |  |  |  |  |
| *SNAI1* | -1,7423988 | 3,01E-06 |  |  |  |  |
| *PPP5C* | -1,3196063 | 0,00014879 |  |  |  |  |
| *ICAM3* | -0,7660787 | 9,52E-05 |  |  |  |  |
| *VEGFA* | -1,3654102 | 0,00011977 |  |  |  |  |
| *BTNL9* | -1,1819561 | 9,75E-06 |  |  |  |  |
| *TMCC1* | -0,8775688 | 3,16E-05 |  |  |  |  |
| *NFKBIA* | -0,9118982 | 6,70E-05 |  |  |  |  |
| *COL27A1* | 2,21070379 | 1,48E-21 |  |  |  |  |
| *ALDH1L1* | 3,14494941 | 8,02E-19 |  |  |  |  |
| *STMN2* | 2,75231425 | 5,18E-17 |  |  |  |  |
| *LOC518623* | 2,63636888 | 1,27E-12 |  |  |  |  |
| *DSG1* | 2,73365381 | 1,48E-11 |  |  |  |  |
| *ETNPPL* | 1,75459261 | 2,92E-10 |  |  |  |  |
| *ATF5* | 1,38852621 | 3,05E-10 |  |  |  |  |
| *DNAH2* | 2,4821706 | 4,45E-10 |  |  |  |  |
| *SREBF1* | 1,23660984 | 2,38E-09 |  |  |  |  |
| *HEPHL1* | 2,39276164 | 3,87E-09 |  |  |  |  |
| *LSS* | 1,48425101 | 5,28E-09 |  |  |  |  |
| *EXTL1* | 1,56364695 | 6,34E-08 |  |  |  |  |
| *MLXIPL* | 1,23261733 | 1,10E-07 |  |  |  |  |
| *MST1* | 1,03086994 | 2,01E-07 |  |  |  |  |
| *LRRN1* | 1,85825328 | 2,69E-07 |  |  |  |  |
| *RCOR2* | 1,71066106 | 4,50E-07 |  |  |  |  |
| *IDI1* | 1,5106319 | 5,54E-07 |  |  |  |  |
| *SERTAD4* | 1,27683705 | 7,93E-07 |  |  |  |  |
| *DSC1* | 1,97714113 | 8,51E-07 |  |  |  |  |
| *SLIT2* | 1,30265706 | 1,13E-06 |  |  |  |  |
| *PFKFB1* | 1,73368724 | 1,16E-06 |  |  |  |  |
| *HOOK2* | 1,60258882 | 1,23E-06 |  |  |  |  |
| *TGM5* | 1,95249212 | 1,55E-06 |  |  |  |  |
| *COLQ* | 1,65305544 | 1,56E-06 |  |  |  |  |
| *LDLR* | 1,30117927 | 1,99E-06 |  |  |  |  |
| *DHCR24* | 1,11071543 | 3,13E-06 |  |  |  |  |
| *BMPER* | 1,61271702 | 3,36E-06 |  |  |  |  |
| *CACNA1G* | 1,70033903 | 4,16E-06 |  |  |  |  |
| *ARVCF* | 1,18179182 | 5,46E-06 |  |  |  |  |
| *ADAMTS6* | 1,24350378 | 9,15E-06 |  |  |  |  |
| *LIN7A* | 1,24361663 | 1,02E-05 |  |  |  |  |
| *DIRAS2* | 1,70467757 | 1,12E-05 |  |  |  |  |
| *DPT* | 1,11153551 | 1,36E-05 |  |  |  |  |
| *BCL6* | 1,05571475 | 1,87E-05 |  |  |  |  |
| *VSIG8* | 1,66431142 | 4,09E-05 |  |  |  |  |
| *KCNK12* | 1,5618185 | 5,65E-05 |  |  |  |  |
| *TSPAN15* | 0,99897059 | 6,70E-05 |  |  |  |  |
| *NLGN1* | 1,33405291 | 0,00013264 |  |  |  |  |
| *DAGLA* | 0,85851335 | 0,00017604 |  |  |  |  |
| *UNC13C* | 1,31549299 | 0,00023236 |  |  |  |  |
| *TMEM182* | 1,4395605 | 0,00027206 |  |  |  |  |
| *CIDEA* | 2,85694893 | 1,51E-25 |  |  |  |  |
| *ACACA* | 2,76866801 | 7,42E-31 |  |  |  |  |
| *GPAM* | 3,52305299 | 2,36E-29 |  |  |  |  |
| *PCK1* | 3,98134858 | 1,39E-31 |  |  |  |  |
| *FASN* | 3,84478769 | 2,59E-33 |  |  |  |  |
| *GSTT2* | 3,01106687 | 4,60E-34 |  |  |  |  |
| *PRNP* | 1,43069525 | 6,16E-17 |  |  |  |  |
| *TDH* | 3,02459284 | 5,83E-26 |  |  |  |  |
| *GPD1* | 2,11137562 | 1,01E-21 |  |  |  |  |
| *MID1IP1* | 2,43966295 | 5,49E-27 |  |  |  |  |
| *ASPN* | 1,6379561 | 2,27E-12 |  |  |  |  |
| *MGST1* | 2,4836601 | 6,34E-18 |  |  |  |  |
| *PCYT2* | 1,7611747 | 1,05E-22 |  |  |  |  |
| *ACSL1* | 2,11665859 | 3,37E-19 |  |  |  |  |
| *KRTAP11-1* | 3,70219139 | 1,79E-20 |  |  |  |  |
| *THRSP* | 2,0929591 | 2,55E-07 |  |  |  |  |
| *ELOVL6* | 2,67727452 | 2,40E-21 |  |  |  |  |
| *GPT2* | 2,41128191 | 5,23E-13 |  |  |  |  |
| *INSIG1* | 2,44606406 | 3,94E-15 |  |  |  |  |
| *QPRT* | 1,62657096 | 9,46E-11 |  |  |  |  |
| *ADIG* | 1,40178779 | 9,49E-11 |  |  |  |  |
| *AGPAT2* | 2,08014019 | 2,38E-15 |  |  |  |  |
| *MDFI* | 1,44750222 | 1,11E-12 |  |  |  |  |
| *ACER3* | 1,87583079 | 8,69E-15 |  |  |  |  |
| *DBI* | 1,76351869 | 1,38E-11 |  |  |  |  |
| *FKBP14* | 1,55423965 | 1,43E-12 |  |  |  |  |
| *CYB5A* | 1,18092674 | 1,65E-12 |  |  |  |  |
| *HMGCS1* | 1,81049704 | 1,02E-10 |  |  |  |  |
| *SFXN2* | 1,7268711 | 9,41E-11 |  |  |  |  |
| *MMP15* | 1,92404449 | 2,23E-13 |  |  |  |  |
| *ACLY* | 1,63229911 | 1,84E-10 |  |  |  |  |
| *HSD17B12* | 1,98510879 | 3,17E-15 |  |  |  |  |
| *LDHB* | 1,21884329 | 1,75E-07 |  |  |  |  |
| *SLC25A4* | 1,2308337 | 1,53E-12 |  |  |  |  |
| *GLYCTK* | 1,28397519 | 3,18E-08 |  |  |  |  |
| *AR* | 1,44037236 | 9,02E-07 |  |  |  |  |
| *ELOVL5* | 1,87080366 | 8,26E-14 |  |  |  |  |
| *MSRB1* | 1,50358651 | 1,24E-11 |  |  |  |  |
| *B3GALNT1* | 0,98250343 | 3,76E-06 |  |  |  |  |
| *AQP7* | 1,32842006 | 1,12E-07 |  |  |  |  |
| *BSG* | 1,36962899 | 5,01E-10 |  |  |  |  |
| *EBP* | 1,60715256 | 4,80E-10 |  |  |  |  |
| *DECR1* | 1,63158374 | 5,45E-10 |  |  |  |  |
| *SBDS* | 0,9868349 | 2,41E-05 |  |  |  |  |
| *LRRC17* | 1,26162622 | 0,00012273 |  |  |  |  |
| *VCAN* | 0,9673242 | 1,19E-05 |  |  |  |  |
| *CCDC3* | 1,24155734 | 5,45E-07 |  |  |  |  |
| *FDFT1* | 0,94183364 | 1,29E-05 |  |  |  |  |
| *MDH1* | 0,98095119 | 7,05E-07 |  |  |  |  |
| *VCAM1* | 1,06329363 | 5,78E-06 |  |  |  |  |
| *LEP* | 1,26094766 | 7,83E-08 |  |  |  |  |
| *BCAT2* | 1,45409135 | 1,73E-14 |  |  |  |  |
| *SFRP4* | 1,48053686 | 4,64E-07 |  |  |  |  |
| *KLHL31* | 1,551239 | 5,08E-08 |  |  |  |  |
| *ACAT2* | 1,53862338 | 4,83E-07 |  |  |  |  |
| *PI16* | 1,62769549 | 2,60E-06 |  |  |  |  |
| *BGN* | 1,38575575 | 1,68E-07 |  |  |  |  |
| *S100G* | 1,48569269 | 1,16E-06 |  |  |  |  |
| *LOXL1* | 1,53232694 | 1,07E-05 |  |  |  |  |
| *MPC1* | 1,25671715 | 1,98E-09 |  |  |  |  |
| *HMCN1* | 0,83072489 | 9,85E-06 |  |  |  |  |
| *LUM* | 1,08593192 | 1,16E-05 |  |  |  |  |
| *BCHE* | 1,25497434 | 6,78E-06 |  |  |  |  |
| *FAM84A* | 1,47308106 | 1,20E-08 |  |  |  |  |
| *PTPLB* | 1,06526679 | 9,22E-05 |  |  |  |  |
| *C18H19orf12* | 0,9401142 | 1,80E-07 |  |  |  |  |
| *QPCT* | 1,54587981 | 7,86E-06 |  |  |  |  |
| *EPDR1* | 1,25472974 | 8,19E-06 |  |  |  |  |
| *P4HA2* | 0,9513966 | 1,47E-05 |  |  |  |  |
| *WISP2* | 1,74912683 | 2,53E-07 |  |  |  |  |
| *NCALD* | 1,08361842 | 6,17E-07 |  |  |  |  |
| *TFRC* | 1,20046219 | 1,97E-05 |  |  |  |  |
| *NID2* | 0,9899983 | 5,30E-07 |  |  |  |  |
| *CCDC88A* | 0,82137468 | 7,76E-05 |  |  |  |  |
| *ATP5G3* | 0,84574548 | 5,04E-08 |  |  |  |  |
| *RCN1* | 0,8718857 | 3,86E-05 |  |  |  |  |
| *NIPSNAP1* | 1,29277887 | 2,73E-06 |  |  |  |  |
| *PYCR1* | 1,68707599 | 1,32E-06 |  |  |  |  |
| *MLEC* | 1,24471675 | 1,23E-08 |  |  |  |  |
| *FITM2* | 0,876617 | 2,00E-06 |  |  |  |  |
| *SLC16A1* | 1,10370436 | 2,34E-08 |  |  |  |  |
| *FGF7* | 0,92448554 | 8,01E-06 |  |  |  |  |
| *ACADSB* | 1,18856535 | 5,89E-06 |  |  |  |  |
| *CALU* | 0,85906199 | 2,13E-06 |  |  |  |  |
| *TALDO1* | 1,01543294 | 1,36E-06 |  |  |  |  |
