# Supplementary material for: Impact of the severity of negative energy balance on gene expression in the subcutaneous adipose tissue of periparturient primiparous Holstein dairy cows: Identification of potential novel metabolic signals for the reproductive system
Source: PLoS One. 2019 Sep 26;14(9):e0222954. doi: 10.1371/journal.pone.0222954 (PMC6763198; doi:10.1371/journal.pone.0222954)
Supplement: S7 Table — (DOCX) [file pone.0222954.s012.docx]

| **S7 Table: Genes differentially expressed at 16 WKPP as compared to 1 WKPP in SNEB animals** | | | | | | |
| --- | --- | --- | --- | --- | --- | --- |
| name | log2FoldChange_exons | pvalue_exons |  |  |  |  |
| *PERP* | -1,46107968 | 2,39E-08 |  |  |  |  |
| *TDH* | -1,48218699 | 6,55E-08 |  |  |  |  |
| *SPRY4* | 1,500729052 | 1,88E-12 |  |  |  |  |
| *PHYH* | 1,20626917 | 4,31E-11 |  |  |  |  |
| *RGS7* | 1,246164737 | 2,49E-09 |  |  |  |  |
| *FABP4* | 0,904882276 | 3,09E-08 |  |  |  |  |
| *ADIPOR2* | 0,920543781 | 3,79E-08 |  |  |  |  |
| *FAM198B* | 1,066631752 | 2,42E-07 |  |  |  |  |
| *HCRTR1* | 1,069275811 | 3,93E-06 |  |  |  |  |
