# Supplementary material for: Impact of the severity of negative energy balance on gene expression in the subcutaneous adipose tissue of periparturient primiparous Holstein dairy cows: Identification of potential novel metabolic signals for the reproductive system
Source: PLoS One. 2019 Sep 26;14(9):e0222954. doi: 10.1371/journal.pone.0222954 (PMC6763198; doi:10.1371/journal.pone.0222954)
Supplement: S8 Table — (DOCX) [file pone.0222954.s013.docx]

| **S8 Table: Genes differentially expressed in SNEB and MNEB animals at 1 WKPP** | | | | | | |
| --- | --- | --- | --- | --- | --- | --- |
| name | log2FoldChange_exons | pvalue_exons | |  |  |  |
| *CHRNA1* | -1,8417896 | 5,34E-11 |  |  |  |  |
| *C10H14orf53* | -3,6651312 | 9,09E-09 |  |  |  |  |
| *TRAF7* | -1,4347955 | 6,82E-08 |  |  |  |  |
| *LOC616942* | -1,5020868 | 9,74E-07 |  |  |  |  |
| *MGC148328* | -3,1326384 | 1,33E-06 |  |  |  |  |
| *C8G* | -1,3560301 | 1,64E-06 |  |  |  |  |
| *RYR1* | -1,4156383 | 3,88E-06 |  |  |  |  |
| *SLC25A37* | -0,9645327 | 4,49E-06 |  |  |  |  |
| *SF3A1* | -0,9774391 | 7,70E-06 |  |  |  |  |
| *ANO4* | -2,8939976 | 7,78E-06 |  |  |  |  |
| *CCDC89* | -2,291268 | 7,92E-06 |  |  |  |  |
| *SMYD1* | -2,8684569 | 9,41E-06 |  |  |  |  |
| *RHOT2* | -1,2581528 | 1,27E-05 |  |  |  |  |
| *GMEB2* | -1,0490258 | 1,54E-05 |  |  |  |  |
| *HEATR4* | -1,5211288 | 1,57E-05 |  |  |  |  |
| *DNAH2* | -2,6849322 | 1,69E-05 |  |  |  |  |
| *GDAP1L1* | -2,5632944 | 1,80E-05 |  |  |  |  |
| *DPP7* | -1,3035672 | 2,19E-05 |  |  |  |  |
| *RECQL5* | -1,1243187 | 2,25E-05 |  |  |  |  |
| *RBM20* | -2,2965601 | 2,35E-05 |  |  |  |  |
| *MAF* | -1,0434585 | 2,50E-05 |  |  |  |  |
| *DSG1* | -2,6955875 | 3,29E-05 |  |  |  |  |
| *MAP3K11* | -0,9998506 | 3,36E-05 |  |  |  |  |
| *HR* | -1,8140543 | 4,96E-05 |  |  |  |  |
| *ARNT2* | -1,543947 | 5,02E-05 |  |  |  |  |
| *NOXA1* | -1,4990637 | 5,72E-05 |  |  |  |  |
| *RFXANK* | -1,4459009 | 5,91E-05 |  |  |  |  |
| *SNAP91* | -2,5987367 | 6,20E-05 |  |  |  |  |
| *STAB2* | -2,5927634 | 6,44E-05 |  |  |  |  |
| *SEMA3B* | -1,3504114 | 7,32E-05 |  |  |  |  |
| *DOK4* | -1,3716062 | 8,20E-05 |  |  |  |  |
| *MYBPC1* | -2,5300092 | 9,55E-05 |  |  |  |  |
| *GTPBP2* | -0,9080022 | 9,99E-05 |  |  |  |  |
| *ASB15* | -2,523573 | 0,00010148 |  |  |  |  |
| *HOXD4* | -1,1280419 | 0,00010227 |  |  |  |  |
| *FANCG* | -0,8035834 | 0,00010577 |  |  |  |  |
| *PRODH* | -1,1721511 | 0,00010649 |  |  |  |  |
| *THBS2* | -0,9798439 | 0,00010819 |  |  |  |  |
| *ABCA12* | -2,5046103 | 0,00011356 |  |  |  |  |
| *TG* | -2,1572834 | 0,00011917 |  |  |  |  |
| *C7H1orf35* | -1,1334206 | 0,00011918 |  |  |  |  |
| *TFIP11* | -0,8891203 | 0,00012585 |  |  |  |  |
| *CPS1* | -2,4870581 | 0,00012736 |  |  |  |  |
| *RCOR2* | -1,453506 | 0,00013859 |  |  |  |  |
| *DNASE1* | -0,9859076 | 0,00015358 |  |  |  |  |
| *ZNF697* | -1,2790948 | 0,00017266 |  |  |  |  |
| *PRPF31* | -0,8820858 | 0,00017404 |  |  |  |  |
| *ADAMTS14* | -1,9588674 | 0,00017904 |  |  |  |  |
| *PNN* | -0,9498939 | 0,00018054 |  |  |  |  |
| *BRPF1* | -1,0268862 | 0,00020994 |  |  |  |  |
| *LOC507550* | -2,2354521 | 0,00022135 |  |  |  |  |
| *VSTM2A* | -2,3887109 | 0,0002239 |  |  |  |  |
| *SEMA7A* | -1,0512628 | 0,00023308 |  |  |  |  |
| *IGF2BP3* | -2,3287452 | 0,00024938 |  |  |  |  |
| *CHGB* | -2,3609694 | 0,00026157 |  |  |  |  |
| *USH2A* | -2,3458705 | 0,00027072 |  |  |  |  |
| *TRPM8* | -2,35752 | 0,00027979 |  |  |  |  |
| *CFTR* | -2,3186298 | 0,00029517 |  |  |  |  |
| *DUSP27* | -2,3354279 | 0,00032144 |  |  |  |  |
| *ZNF34* | -0,8182869 | 0,00032437 |  |  |  |  |
| *PRPF4B* | -0,8258809 | 0,00033195 |  |  |  |  |
| *NPR2* | -0,7969638 | 0,00034159 |  |  |  |  |
| *LOC100616526* | -2,3122831 | 0,0003478 |  |  |  |  |
| *CLDN15* | -1,2406967 | 0,00036081 |  |  |  |  |
| *KCNA4* | -2,2864719 | 0,00037323 |  |  |  |  |
| *ENO2* | -1,9757223 | 0,00040568 |  |  |  |  |
| *LSAMP* | -1,7559002 | 0,00041661 |  |  |  |  |
| *SYBU* | -1,1306016 | 0,0004168 |  |  |  |  |
| *RASGEF1A* | -1,4414498 | 0,00045396 |  |  |  |  |
| *NOP2* | -0,7350695 | 0,00047645 |  |  |  |  |
| *VIPR1* | -1,8850167 | 0,00048767 |  |  |  |  |
| *TMEM52B* | -2,2418005 | 0,00050729 |  |  |  |  |
| *MPP3* | -2,2044723 | 0,00051312 |  |  |  |  |
| *IP6K3* | -2,253263 | 0,00051454 |  |  |  |  |
| *BPIFC* | -2,2315515 | 0,00056803 |  |  |  |  |
| *FGF14* | -2,2224196 | 0,00061388 |  |  |  |  |
| *CDK5RAP3* | -0,8713282 | 0,00061909 |  |  |  |  |
| *MAP1LC3A* | -0,7126551 | 0,00066223 |  |  |  |  |
| *NOS2* | -1,3701488 | 0,00070077 |  |  |  |  |
| *BATF2* | -1,5599084 | 0,00070609 |  |  |  |  |
| *CLDN16* | -2,1752371 | 0,00072256 |  |  |  |  |
| *GC* | -2,1871207 | 0,0007344 |  |  |  |  |
| *LRRIQ1* | -2,1816606 | 0,00074594 |  |  |  |  |
| *HKDC1* | -2,1642298 | 0,00076021 |  |  |  |  |
| *TSR3* | -0,8878146 | 0,00076591 |  |  |  |  |
| *FBXO40* | -2,1314662 | 0,00076937 |  |  |  |  |
| *STXBP5L* | -2,1618396 | 0,00084841 |  |  |  |  |
| *STAG3* | -1,7553669 | 0,00088733 |  |  |  |  |
| *CADPS* | -2,1544324 | 0,00089035 |  |  |  |  |
| *KRT5* | -2,1563778 | 0,00089096 |  |  |  |  |
| *MAPK10* | -2,1467801 | 0,00092767 |  |  |  |  |
| *EPHA7* | -1,9542415 | 0,00093016 |  |  |  |  |
| *MYH2* | -2,1192883 | 0,00103324 |  |  |  |  |
| *DMP1* | -2,1130232 | 0,00104065 |  |  |  |  |
| *MGC134232* | -2,1029156 | 0,0010432 |  |  |  |  |
| *PLS1* | -2,1097218 | 0,00108601 |  |  |  |  |
| *TNNI3K* | -2,1090429 | 0,00114026 |  |  |  |  |
| *KIF5C* | -1,8443173 | 0,00114215 |  |  |  |  |
| *SLC4A10* | -2,109302 | 0,00115617 |  |  |  |  |
| *ITIH1* | -2,0784017 | 0,00118403 |  |  |  |  |
| *BMPR1B* | -1,7327703 | 0,00120299 |  |  |  |  |
| *MRAP2* | -2,0581887 | 0,0012612 |  |  |  |  |
| *DSC1* | -2,0688002 | 0,00129023 |  |  |  |  |
| *PBX2* | -0,8566906 | 0,00131916 |  |  |  |  |
| *JAKMIP2* | -1,9967915 | 0,00135725 |  |  |  |  |
| *DNAH9* | -1,8892861 | 0,00143475 |  |  |  |  |
| *GBP4* | -1,3552582 | 0,00143869 |  |  |  |  |
| *CYLC1* | -2,0349743 | 0,00146275 |  |  |  |  |
| *ASPN* | 2,10706092 | 5,50E-13 |  |  |  |  |
| *GHR* | 1,96297164 | 1,26E-11 |  |  |  |  |
| *FHL1* | 1,23630428 | 1,32E-11 |  |  |  |  |
| *MEDAG* | 1,44102029 | 3,85E-11 |  |  |  |  |
| *CRYZ* | 1,41734717 | 1,11E-09 |  |  |  |  |
| *ORMDL3* | 1,73673039 | 1,32E-09 |  |  |  |  |
| *CYP1B1* | 1,97151109 | 1,98E-09 |  |  |  |  |
| *NFU1* | 1,48049876 | 9,30E-09 |  |  |  |  |
| *LBP* | 1,98654502 | 3,39E-08 |  |  |  |  |
| *HSD17B4* | 1,0639217 | 4,57E-08 |  |  |  |  |
| *CCDC3* | 1,77662632 | 6,68E-08 |  |  |  |  |
| *PLIN1* | 1,36776165 | 6,93E-08 |  |  |  |  |
| *PGAM1* | 1,23606978 | 1,08E-07 |  |  |  |  |
| *UQCRB* | 1,66707774 | 1,10E-07 |  |  |  |  |
| *C9H6orf120* | 1,22328435 | 1,11E-07 |  |  |  |  |
| *SSR1* | 1,33404961 | 1,50E-07 |  |  |  |  |
| *MBOAT1* | 1,41761794 | 1,78E-07 |  |  |  |  |
| *GPX3* | 1,70691413 | 2,05E-07 |  |  |  |  |
| *KDSR* | 1,33255414 | 2,35E-07 |  |  |  |  |
| *MIR6516* | 2,42820811 | 2,55E-07 |  |  |  |  |
| *MCFD2* | 1,51227733 | 2,72E-07 |  |  |  |  |
| *TUSC5* | 1,5954951 | 5,35E-07 |  |  |  |  |
| *CANX* | 1,18775829 | 1,44E-06 |  |  |  |  |
| *LANCL1* | 1,10730163 | 2,10E-06 |  |  |  |  |
| *ACAT1* | 1,0890917 | 2,16E-06 |  |  |  |  |
| *REEP5* | 1,10690244 | 2,48E-06 |  |  |  |  |
| *PLP2* | 1,49304725 | 2,99E-06 |  |  |  |  |
| *PRNP* | 1,16522852 | 3,23E-06 |  |  |  |  |
| *B3GALNT1* | 1,61049718 | 3,79E-06 |  |  |  |  |
| *SOD2* | 1,36662049 | 3,97E-06 |  |  |  |  |
| *PDHA1* | 1,00923389 | 4,26E-06 |  |  |  |  |
| *DDIT4L* | 1,43779272 | 5,15E-06 |  |  |  |  |
| *SLC26A2* | 1,68604908 | 6,77E-06 |  |  |  |  |
| *APOD* | 2,073157 | 8,64E-06 |  |  |  |  |
| *FSTL3* | 1,2876728 | 9,24E-06 |  |  |  |  |
| *RBP4* | 1,71852724 | 9,92E-06 |  |  |  |  |
| *BNIP3L* | 0,9362524 | 1,02E-05 |  |  |  |  |
| *SLC1A5* | 1,47926659 | 1,15E-05 |  |  |  |  |
| *DCBLD2* | 1,01751826 | 1,35E-05 |  |  |  |  |
| *MFGE8* | 1,37694316 | 1,68E-05 |  |  |  |  |
| *ARL2BP* | 1,10085414 | 2,08E-05 |  |  |  |  |
| *SC5D* | 1,22508512 | 2,32E-05 |  |  |  |  |
| *ARL6IP1* | 1,11986701 | 2,36E-05 |  |  |  |  |
| *MTPN* | 0,86554397 | 2,57E-05 |  |  |  |  |
| *GHITM* | 1,07866456 | 2,79E-05 |  |  |  |  |
| *SLC48A1* | 1,33722368 | 2,85E-05 |  |  |  |  |
| *PSMB5* | 1,05171388 | 3,79E-05 |  |  |  |  |
| *HSD17B10* | 1,00274789 | 4,22E-05 |  |  |  |  |
| *CHTF8* | 1,09211347 | 4,32E-05 |  |  |  |  |
| *FBP1* | 1,74544984 | 4,66E-05 |  |  |  |  |
| *ISCA1* | 0,97428373 | 5,41E-05 |  |  |  |  |
| *ANXA1* | 1,15584198 | 5,61E-05 |  |  |  |  |
| *DSE* | 1,13693127 | 6,42E-05 |  |  |  |  |
| *ATP5B* | 1,02617764 | 7,49E-05 |  |  |  |  |
| *EIF1AX* | 0,88612472 | 8,16E-05 |  |  |  |  |
| *PCDH18* | 0,93799898 | 8,36E-05 |  |  |  |  |
| *PTGER3* | 1,46981494 | 8,49E-05 |  |  |  |  |
| *ATP5A1* | 0,88545076 | 8,50E-05 |  |  |  |  |
| *SEL1L* | 0,842085 | 8,86E-05 |  |  |  |  |
| *CCNDBP1* | 0,86391184 | 9,33E-05 |  |  |  |  |
| *FAM105A* | 1,24984209 | 0,00011185 |  |  |  |  |
| *GFPT1* | 0,84253075 | 0,00011863 |  |  |  |  |
| *RPS17* | 0,91926003 | 0,00012018 |  |  |  |  |
| *ALDH9A1* | 0,76042801 | 0,000124 |  |  |  |  |
| *TMED4* | 1,08168776 | 0,00013115 |  |  |  |  |
| *DNAJC18* | 0,82389947 | 0,00013425 |  |  |  |  |
| *ADIRF* | 1,19296288 | 0,00013963 |  |  |  |  |
| *PSMA1* | 0,93493661 | 0,00014087 |  |  |  |  |
| *EIF4A3* | 0,99216873 | 0,00014127 |  |  |  |  |
| *MIF* | 1,30584275 | 0,00014705 |  |  |  |  |
| *PAIP2* | 0,95256677 | 0,0001484 |  |  |  |  |
| *LSM8* | 1,03526786 | 0,00015353 |  |  |  |  |
| *VIM* | 0,86269063 | 0,00015683 |  |  |  |  |
| *PDP1* | 0,96836011 | 0,0001719 |  |  |  |  |
| *TMEM167B* | 0,93979404 | 0,00018841 |  |  |  |  |
| *NDUFA4* | 0,97023655 | 0,00018878 |  |  |  |  |
| *FITM2* | 1,21417434 | 0,00019698 |  |  |  |  |
| *HERPUD1* | 1,11829248 | 0,00020351 |  |  |  |  |
| *TIPARP* | 1,20524956 | 0,00021693 |  |  |  |  |
| *HSP90AA1* | 0,68373526 | 0,00022554 |  |  |  |  |
| *ALG10* | 1,27823808 | 0,00025137 |  |  |  |  |
| *TSPYL1* | 1,01096176 | 0,00025218 |  |  |  |  |
| *SYT4* | 0,90451964 | 0,00027496 |  |  |  |  |
| *OXCT1* | 0,80038336 | 0,00027875 |  |  |  |  |
| *YIPF5* | 0,82760287 | 0,00028685 |  |  |  |  |
| *PGM5* | 0,86777851 | 0,00029179 |  |  |  |  |
| *ATP5G2* | 1,02243916 | 0,00033497 |  |  |  |  |
| *ADIG* | 1,25065942 | 0,00038279 |  |  |  |  |
| *PDK1* | 0,89102452 | 0,00041396 |  |  |  |  |
| *PRDX6* | 0,87250768 | 0,00044792 |  |  |  |  |
| *DUSP1* | 0,88320556 | 0,00045532 |  |  |  |  |
| *SRP14* | 0,82261756 | 0,00046133 |  |  |  |  |
| *FAM210B* | 0,99870561 | 0,00046313 |  |  |  |  |
| *VCAN* | 1,19546698 | 0,00046582 |  |  |  |  |
| *LDHB* | 0,88921161 | 0,00050467 |  |  |  |  |
| *SBDS* | 0,89681554 | 0,00056057 |  |  |  |  |
| *DDB1* | 0,77621898 | 0,00056535 |  |  |  |  |
| *AHR* | 1,06183872 | 0,00059582 |  |  |  |  |
| *OAT* | 0,9908399 | 0,00061163 |  |  |  |  |
| *SMIM19* | 0,75156303 | 0,00061582 |  |  |  |  |
| *ELL2* | 0,97504815 | 0,00073645 |  |  |  |  |
| *NUDT4* | 1,2229201 | 0,00073918 |  |  |  |  |
| *ASB8* | 0,91915136 | 0,00077435 |  |  |  |  |
| *ANXA2* | 1,03830272 | 0,00079489 |  |  |  |  |
| *sept-10* | 0,81089721 | 0,00085413 |  |  |  |  |
| *TMEM14C* | 0,84636771 | 0,0008658 |  |  |  |  |
| *MPC1* | 0,90941215 | 0,00099705 |  |  |  |  |
| *SF3B6* | 0,88174787 | 0,00110178 |  |  |  |  |
| *FOS* | 0,99673382 | 0,00113027 |  |  |  |  |
| *HP* | 2,0823449 | 0,00115225 |  |  |  |  |
| *MFF* | 0,75046863 | 0,00123593 |  |  |  |  |
| *C4H7orf73* | 0,8733297 | 0,00125019 |  |  |  |  |
| *TES* | 0,72016326 | 0,00138519 |  |  |  |  |
| *SPEG* | -2,3106195 | 6,80E-24 |  |  |  |  |
| *ADGRL1* | -1,84126535 | 3,33E-16 |  |  |  |  |
| *PLCG1* | -1,59439006 | 3,83E-11 |  |  |  |  |
| *PPP1R13L* | -1,50529335 | 7,08E-12 |  |  |  |  |
| *COL18A1* | -1,74333583 | 6,67E-12 |  |  |  |  |
| *ROBO4* | -1,863469 | 1,80E-12 |  |  |  |  |
| *ALS2CL* | -2,01905387 | 9,80E-12 |  |  |  |  |
| *EML3* | -1,39436891 | 1,90E-09 |  |  |  |  |
| *OSBPL7* | -1,94764946 | 2,69E-11 |  |  |  |  |
| *ITGA7* | -1,78578962 | 2,01E-11 |  |  |  |  |
| *ADAMTS10* | -1,63017099 | 3,61E-11 |  |  |  |  |
| *SMTN* | -1,99421294 | 1,15E-10 |  |  |  |  |
| *DDX39B* | -1,02675752 | 8,26E-06 |  |  |  |  |
| *RN7SL1* | -2,83838166 | 8,86E-12 |  |  |  |  |
| *CAMTA2* | -1,22146666 | 1,49E-09 |  |  |  |  |
| *MED12* | -1,0513456 | 5,66E-08 |  |  |  |  |
| *CLASRP* | -2,1958515 | 1,30E-11 |  |  |  |  |
| *MPRIP* | -1,81636026 | 1,65E-10 |  |  |  |  |
| *NEURL4* | -1,36043817 | 7,96E-08 |  |  |  |  |
| *SETD1A* | -1,71374863 | 1,55E-09 |  |  |  |  |
| *GRIPAP1* | -2,19853678 | 1,01E-09 |  |  |  |  |
| *TRABD* | -1,69672026 | 1,02E-08 |  |  |  |  |
| *RNF123* | -1,70492313 | 1,97E-09 |  |  |  |  |
| *HID1* | -2,2136846 | 5,55E-10 |  |  |  |  |
| *PKN1* | -1,13950796 | 2,85E-08 |  |  |  |  |
| *ZSWIM8* | -1,18760501 | 2,94E-07 |  |  |  |  |
| *BCL9L* | -1,42385035 | 2,40E-10 |  |  |  |  |
| *HAUS5* | -1,40526795 | 2,86E-08 |  |  |  |  |
| *PIDD1* | -1,90497162 | 4,24E-10 |  |  |  |  |
| *CCDC183* | -2,59786106 | 3,29E-11 |  |  |  |  |
| *ULK1* | -1,42654729 | 6,74E-10 |  |  |  |  |
| *GIGYF1* | -1,65346387 | 2,77E-08 |  |  |  |  |
| *STK25* | -1,66343938 | 1,15E-07 |  |  |  |  |
| *RABL6* | -1,39358405 | 9,80E-08 |  |  |  |  |
| *ANKRD52* | -1,26254674 | 1,26E-07 |  |  |  |  |
| *SRRT* | -1,37583464 | 7,46E-08 |  |  |  |  |
| *NDOR1* | -1,62698471 | 4,48E-08 |  |  |  |  |
| *TCAP* | -1,49610924 | 3,99E-08 |  |  |  |  |
| *KLHL17* | -1,82179335 | 6,34E-10 |  |  |  |  |
| *OPLAH* | -1,55157308 | 1,67E-07 |  |  |  |  |
| *FHOD1* | -1,59557076 | 3,12E-08 |  |  |  |  |
| *MORC2* | -1,60563851 | 9,79E-09 |  |  |  |  |
| *DOCK6* | -1,72945979 | 8,42E-09 |  |  |  |  |
| *ATF6B* | -1,48992587 | 2,97E-08 |  |  |  |  |
| *EHBP1L1* | -1,10733386 | 2,44E-07 |  |  |  |  |
| *CXXC1* | -2,14248194 | 1,02E-08 |  |  |  |  |
| *ZBTB40* | -1,71003574 | 8,13E-11 |  |  |  |  |
| *LRWD1* | -1,4734134 | 5,57E-08 |  |  |  |  |
| *TAF1C* | -1,16621888 | 3,14E-07 |  |  |  |  |
| *PPP6R1* | -1,16831886 | 0,000255895 |  |  |  |  |
| *RNF40* | -1,4395555 | 1,01E-07 |  |  |  |  |
| *AMT* | -1,19961067 | 3,14E-07 |  |  |  |  |
| *U2AF2* | -1,3117772 | 4,62E-08 |  |  |  |  |
| *ADCY4* | -1,57630654 | 1,06E-07 |  |  |  |  |
| *CCDC88B* | -2,04242605 | 3,56E-10 |  |  |  |  |
| *PHRF1* | -1,50072144 | 2,22E-08 |  |  |  |  |
| *AKAP6* | -0,99352099 | 0,001195497 |  |  |  |  |
| *CABP1* | -2,3057292 | 5,68E-05 |  |  |  |  |
| *BRD3* | -1,26289799 | 1,57E-06 |  |  |  |  |
| *HSF4* | -1,50912971 | 3,49E-07 |  |  |  |  |
| *HDAC7* | -1,38014386 | 2,98E-08 |  |  |  |  |
| *MADD* | -1,55303867 | 1,89E-08 |  |  |  |  |
| *PCBP4* | -1,74556724 | 1,06E-09 |  |  |  |  |
| *MINK1* | -1,90145251 | 2,43E-07 |  |  |  |  |
| *PKN3* | -1,49344096 | 2,29E-08 |  |  |  |  |
| *MICALL1* | -1,88284406 | 4,23E-09 |  |  |  |  |
| *KIFC2* | -1,8485017 | 3,79E-09 |  |  |  |  |
| *PIP5K1C* | -1,46424833 | 4,11E-08 |  |  |  |  |
| *PLEKHJ1* | -1,59563773 | 2,01E-07 |  |  |  |  |
| *TONSL* | -1,67092116 | 3,42E-08 |  |  |  |  |
| *PPP1R18* | -1,21514323 | 1,01E-07 |  |  |  |  |
| *RABEP2* | -1,50251944 | 5,82E-08 |  |  |  |  |
| *SURF6* | -1,79689627 | 1,13E-07 |  |  |  |  |
| *ANO1* | -1,31273232 | 6,92E-08 |  |  |  |  |
| *CASKIN2* | -1,33507171 | 1,44E-07 |  |  |  |  |
| *STARD3* | -1,37777257 | 3,56E-07 |  |  |  |  |
| *ATXN2L* | -0,89710204 | 9,48E-06 |  |  |  |  |
| *NCAPH2* | -1,32082941 | 1,16E-06 |  |  |  |  |
| *HDGFRP2* | -1,30231424 | 1,30E-06 |  |  |  |  |
| *GCC1* | -1,72417931 | 4,77E-07 |  |  |  |  |
| *ZNF335* | -1,40808207 | 9,76E-08 |  |  |  |  |
| *DGCR8* | -1,27464765 | 1,21E-06 |  |  |  |  |
| *MYBL2* | -2,10377344 | 1,55E-09 |  |  |  |  |
| *CAD* | -0,8623394 | 1,45E-05 |  |  |  |  |
| *TMEM63B* | -1,72637117 | 1,66E-11 |  |  |  |  |
| *RAPGEF3* | -1,64102076 | 3,44E-09 |  |  |  |  |
| *SYMPK* | -1,44234491 | 3,30E-08 |  |  |  |  |
| *DYNC1H1* | -0,81977171 | 5,62E-05 |  |  |  |  |
| *NTRK3* | -1,97584759 | 8,93E-08 |  |  |  |  |
| *LZTR1* | -1,29703226 | 1,42E-06 |  |  |  |  |
| *GTPBP3* | -1,29761029 | 8,03E-06 |  |  |  |  |
| *POLRMT* | -1,51917432 | 6,12E-08 |  |  |  |  |
| *GABBR1* | -1,73941739 | 1,69E-08 |  |  |  |  |
| *COG1* | -1,15025176 | 9,10E-07 |  |  |  |  |
| *PDLIM7* | -1,43419941 | 4,33E-11 |  |  |  |  |
| *NELFA* | -1,69787461 | 2,63E-08 |  |  |  |  |
| *TAOK2* | -1,42268996 | 3,13E-09 |  |  |  |  |
| *C29H11orf84* | -1,37770819 | 1,06E-06 |  |  |  |  |
| *PDE2A* | -1,61218756 | 2,05E-10 |  |  |  |  |
| *GNAT1* | -1,524282 | 1,08E-07 |  |  |  |  |
| *SLC6A17* | -1,61921539 | 0,000399855 |  |  |  |  |
| *ZNF629* | -1,04820934 | 3,77E-06 |  |  |  |  |
| *PACSIN3* | -1,89333206 | 4,37E-08 |  |  |  |  |
| *COL27A1* | -2,742038 | 3,55E-10 |  |  |  |  |
| *KRI1* | -1,54654054 | 3,96E-07 |  |  |  |  |
| *GNB3* | -2,13905677 | 1,10E-08 |  |  |  |  |
| *ACAP1* | -1,44271726 | 2,84E-07 |  |  |  |  |
| *LIG1* | -2,16614527 | 3,75E-08 |  |  |  |  |
| *HECTD3* | -1,13173205 | 4,87E-06 |  |  |  |  |
| *CCDC9* | -1,75212489 | 7,63E-08 |  |  |  |  |
| *SREBF2* | -1,39010551 | 7,87E-10 |  |  |  |  |
| *ARHGEF15* | -1,56018414 | 1,63E-06 |  |  |  |  |
| *ATP2A1* | -2,52838892 | 1,78E-08 |  |  |  |  |
| *ARGLU1* | -2,20257903 | 6,17E-10 |  |  |  |  |
| *RTEL1* | -1,66452408 | 3,46E-07 |  |  |  |  |
| *MICALL2* | -1,73457094 | 4,83E-08 |  |  |  |  |
| *VARS2* | -1,10931642 | 2,12E-06 |  |  |  |  |
| *AHDC1* | -1,25011728 | 1,89E-07 |  |  |  |  |
| *MAEA* | -1,33321702 | 4,90E-07 |  |  |  |  |
| *SART1* | -1,22466911 | 7,10E-06 |  |  |  |  |
| *PER2* | -1,61586569 | 1,25E-08 |  |  |  |  |
| *ADRBK1* | -1,08387577 | 7,33E-06 |  |  |  |  |
| *PER1* | -1,1774766 | 4,34E-06 |  |  |  |  |
| *MAP3K6* | -1,1836924 | 3,58E-06 |  |  |  |  |
| *LZTS1* | -1,61784967 | 1,38E-06 |  |  |  |  |
| *TAF6L* | -1,33596165 | 1,02E-07 |  |  |  |  |
| *CARD11* | -1,68571016 | 5,65E-08 |  |  |  |  |
| *SHKBP1* | -1,37202154 | 8,98E-07 |  |  |  |  |
| *MYO1C* | -0,86189317 | 6,05E-06 |  |  |  |  |
| *ACAD9* | -1,5832775 | 8,80E-09 |  |  |  |  |
| *SPSB3* | -1,13530822 | 2,50E-05 |  |  |  |  |
| *SYNGAP1* | -1,84357533 | 2,26E-07 |  |  |  |  |
| *PNPLA6* | -1,42105754 | 9,16E-09 |  |  |  |  |
| *MTA1* | -1,74597139 | 2,30E-08 |  |  |  |  |
| *SLC9A5* | -2,23296303 | 1,03E-07 |  |  |  |  |
| *CYHR1* | -1,1390614 | 3,73E-05 |  |  |  |  |
| *CDK11B* | -1,33872649 | 1,65E-06 |  |  |  |  |
| *NUMA1* | -1,16487405 | 5,58E-06 |  |  |  |  |
| *TAF6* | -1,57140153 | 1,74E-06 |  |  |  |  |
| *TRIP10* | -1,28808998 | 5,93E-06 |  |  |  |  |
| *CCAR2* | -0,99579604 | 1,11E-05 |  |  |  |  |
| *MAPK8IP3* | -1,65326032 | 2,04E-07 |  |  |  |  |
| *NARFL* | -1,61785742 | 1,60E-07 |  |  |  |  |
| *RASGRP2* | -1,56605291 | 1,46E-07 |  |  |  |  |
| *INSC* | -2,09345862 | 0,000477025 |  |  |  |  |
| *TNNT2* | -2,13890438 | 2,28E-07 |  |  |  |  |
| *IGF2* | -0,96979588 | 3,82E-05 |  |  |  |  |
| *DUS3L* | -1,16360317 | 1,93E-05 |  |  |  |  |
| *PLEKHH3* | -1,59631344 | 1,17E-06 |  |  |  |  |
| *MLXIPL* | -1,76283937 | 1,65E-07 |  |  |  |  |
| *RBM10* | -1,11009467 | 3,46E-06 |  |  |  |  |
| *NOTCH4* | -1,56891066 | 1,84E-06 |  |  |  |  |
| *PHF1* | -0,91838043 | 3,78E-05 |  |  |  |  |
| *PLCB3* | -1,16752191 | 1,06E-05 |  |  |  |  |
| *NOP14* | -1,23186068 | 9,46E-07 |  |  |  |  |
| *HGS* | -0,97980708 | 9,44E-06 |  |  |  |  |
| *TOP3A* | -1,22718085 | 1,68E-07 |  |  |  |  |
| *SSH3* | -1,28963241 | 5,40E-06 |  |  |  |  |
| *ANKRD13D* | -1,51934184 | 3,84E-06 |  |  |  |  |
| *EHMT1* | -1,35037997 | 6,41E-08 |  |  |  |  |
| *TNIP2* | -1,32107817 | 8,65E-05 |  |  |  |  |
| *ARRDC1* | -1,07176359 | 1,45E-05 |  |  |  |  |
| *MROH1* | -1,37348897 | 1,18E-07 |  |  |  |  |
| *ARHGEF10L* | -1,56467611 | 7,18E-08 |  |  |  |  |
| *PLA2G4B* | -1,66519403 | 1,72E-07 |  |  |  |  |
| *PPP1R37* | -1,5335857 | 2,54E-08 |  |  |  |  |
| *ITGA2B* | -1,43827212 | 7,76E-07 |  |  |  |  |
| *TNFRSF4* | -1,76000592 | 2,73E-06 |  |  |  |  |
| *DAGLA* | -1,56745238 | 1,60E-06 |  |  |  |  |
| *ADCY6* | -0,81634752 | 0,000154427 |  |  |  |  |
| *PABPN1* | -1,37112111 | 5,10E-07 |  |  |  |  |
| *TJAP1* | -1,8786129 | 7,37E-08 |  |  |  |  |
| *SMARCA4* | -1,52842604 | 5,12E-08 |  |  |  |  |
| *EHMT2* | -1,24571136 | 3,82E-06 |  |  |  |  |
| *LRCH4* | -1,13371493 | 8,50E-07 |  |  |  |  |
| *USP11* | -1,14054792 | 4,94E-06 |  |  |  |  |
| *AXIN1* | -1,55151544 | 2,36E-08 |  |  |  |  |
| *AZI1* | -1,74644429 | 1,06E-06 |  |  |  |  |
| *NUDT16L1* | -1,44528284 | 1,23E-05 |  |  |  |  |
| *DHX37* | -1,42225274 | 3,28E-08 |  |  |  |  |
| *ANKZF1* | -1,14735397 | 2,21E-05 |  |  |  |  |
| *ABTB1* | -1,14759859 | 7,14E-05 |  |  |  |  |
| *AMPD2* | -1,09299915 | 2,62E-05 |  |  |  |  |
| *TNFRSF10D* | -1,02050049 | 0,000136533 |  |  |  |  |
| *SPTAN1* | -0,83526672 | 0,000166558 |  |  |  |  |
| *TCIRG1* | -0,96231931 | 6,70E-05 |  |  |  |  |
| *MAPK12* | -2,11166096 | 2,20E-07 |  |  |  |  |
| *CNTNAP1* | -1,51596553 | 2,06E-06 |  |  |  |  |
| *CDK3* | -1,23427631 | 1,74E-05 |  |  |  |  |
| *NRBP2* | -1,59791203 | 9,80E-06 |  |  |  |  |
| *PPP1R16A* | -1,36487479 | 3,00E-05 |  |  |  |  |
| *ARHGEF17* | -1,13602511 | 5,29E-07 |  |  |  |  |
| *CCDC102A* | -1,8197073 | 3,12E-07 |  |  |  |  |
| *SSNA1* | -1,38058095 | 2,76E-05 |  |  |  |  |
| *LLGL2* | -1,50568629 | 3,51E-06 |  |  |  |  |
| *XPC* | -1,48969737 | 4,09E-07 |  |  |  |  |
| *SLC4A11* | -1,71707129 | 2,06E-06 |  |  |  |  |
| *ZC3H4* | -0,91213074 | 0,000208084 |  |  |  |  |
| *RIPK3* | -1,23619357 | 3,63E-05 |  |  |  |  |
| *ELAC2* | -1,19681133 | 4,79E-06 |  |  |  |  |
| *MAP3K12* | -1,45596938 | 3,13E-06 |  |  |  |  |
| *DVL1* | -1,13052903 | 2,64E-05 |  |  |  |  |
| *CHMP4A* | -1,48387767 | 6,07E-06 |  |  |  |  |
| *IKBKG* | -1,5253685 | 3,85E-07 |  |  |  |  |
| *CORO7* | -1,4336562 | 2,00E-07 |  |  |  |  |
| *EGFL7* | -1,15878157 | 1,76E-05 |  |  |  |  |
| *TGM2* | -1,21764533 | 0,000215513 |  |  |  |  |
| *VPS52* | -0,95903505 | 6,43E-05 |  |  |  |  |
| *ITPR3* | -1,8745755 | 1,69E-06 |  |  |  |  |
| *INPPL1* | -0,78585058 | 2,80E-05 |  |  |  |  |
| *DGKD* | -1,50630479 | 4,04E-07 |  |  |  |  |
| *PCNXL3* | -0,83094428 | 0,000113306 |  |  |  |  |
| *MKNK2* | -1,08887272 | 2,86E-05 |  |  |  |  |
| *CYTH2* | -1,12073442 | 7,61E-06 |  |  |  |  |
| *PANK4* | -1,19238076 | 1,06E-06 |  |  |  |  |
| *DHX38* | -0,94966321 | 0,000103843 |  |  |  |  |
| *MYH3* | -1,8177549 | 4,77E-05 |  |  |  |  |
| *DDX41* | -1,08772796 | 0,000100281 |  |  |  |  |
| *TADA3* | -1,22319031 | 7,74E-06 |  |  |  |  |
| *ELF3* | -2,11422275 | 2,37E-06 |  |  |  |  |
| *COL11A2* | -2,31166884 | 8,18E-06 |  |  |  |  |
| *FBF1* | -1,59275968 | 3,87E-06 |  |  |  |  |
| *PLEKHG3* | -0,87143223 | 2,40E-05 |  |  |  |  |
| *CHERP* | -1,01816772 | 4,76E-05 |  |  |  |  |
| *EP400* | -1,04687689 | 1,01E-05 |  |  |  |  |
| *STK19* | -0,97037442 | 7,21E-05 |  |  |  |  |
| *ATG16L1* | -1,13906476 | 1,48E-05 |  |  |  |  |
| *ELF4* | -1,09869028 | 3,58E-06 |  |  |  |  |
| *DGKQ* | -1,13093649 | 0,000172434 |  |  |  |  |
| *OSBP* | -1,3638885 | 3,67E-08 |  |  |  |  |
| *RHBDF1* | -1,39920814 | 1,68E-09 |  |  |  |  |
| *KLC2* | -1,55285402 | 2,24E-05 |  |  |  |  |
| *FAM50A* | -1,2732681 | 5,82E-05 |  |  |  |  |
| *PRR14* | -1,17201383 | 5,42E-05 |  |  |  |  |
| *KANK3* | -1,4764465 | 1,71E-06 |  |  |  |  |
| *RAD9A* | -1,19757175 | 9,37E-05 |  |  |  |  |
| *USHBP1* | -1,14756402 | 5,44E-05 |  |  |  |  |
| *UBR4* | -0,88526189 | 6,26E-06 |  |  |  |  |
| *SH3GLB2* | -1,30507419 | 3,18E-05 |  |  |  |  |
| *SFN* | -2,4122273 | 1,21E-05 |  |  |  |  |
| *PDE4A* | -1,01739908 | 0,000104224 |  |  |  |  |
| *FZR1* | -1,28426017 | 4,94E-07 |  |  |  |  |
| *HRC* | -2,09482601 | 9,99E-06 |  |  |  |  |
| *DGCR2* | -1,0797055 | 2,15E-05 |  |  |  |  |
| *IRAK1* | -1,01240497 | 6,79E-05 |  |  |  |  |
| *VASH1* | -1,13844385 | 1,11E-05 |  |  |  |  |
| *PTP4A3* | -1,47915824 | 2,19E-05 |  |  |  |  |
| *NAPRT* | -1,09979738 | 0,0001162 |  |  |  |  |
| *SNRNP70* | -1,52767507 | 8,14E-08 |  |  |  |  |
| *DEPTOR* | -2,08073415 | 5,40E-15 |  |  |  |  |
| *ATP13A2* | -1,15577583 | 1,22E-06 |  |  |  |  |
| *EVI5L* | -1,13706788 | 5,70E-06 |  |  |  |  |
| *BOD1L* | -0,99401222 | 7,35E-05 |  |  |  |  |
| *SPG7* | -1,251626 | 6,15E-06 |  |  |  |  |
| *CHST3* | -1,09107961 | 0,000270258 |  |  |  |  |
| *GGA3* | -0,89011276 | 0,000197921 |  |  |  |  |
| *USP20* | -1,33517729 | 2,95E-06 |  |  |  |  |
| *TCHP* | -1,27658034 | 1,23E-05 |  |  |  |  |
| *MAMDC4* | -1,57248615 | 1,72E-05 |  |  |  |  |
| *RPS6KB2* | -0,96267526 | 9,92E-05 |  |  |  |  |
| *RNF207* | -1,91283944 | 0,000281469 |  |  |  |  |
| *MAP3K14* | -1,49566275 | 2,03E-07 |  |  |  |  |
| *POLR3D* | -1,3873058 | 3,43E-06 |  |  |  |  |
| *ODF2* | -1,49393152 | 2,19E-07 |  |  |  |  |
| *PIH1D1* | -1,71859473 | 9,08E-07 |  |  |  |  |
| *ZNF692* | -1,27733397 | 3,50E-05 |  |  |  |  |
| *SEMA4C* | -0,94634659 | 9,37E-05 |  |  |  |  |
| *HOOK2* | -2,3433923 | 1,09E-05 |  |  |  |  |
| *LETM1* | -1,30633131 | 4,58E-05 |  |  |  |  |
| *RRP1* | -1,24142296 | 2,01E-05 |  |  |  |  |
| *AFAP1L2* | -1,39908258 | 1,66E-05 |  |  |  |  |
| *SLC4A2* | -0,8882754 | 0,000288275 |  |  |  |  |
| *EVL* | -1,6534559 | 2,24E-07 |  |  |  |  |
| *HIP1* | -1,39036145 | 3,64E-07 |  |  |  |  |
| *SRSF2* | -0,75255298 | 0,000813493 |  |  |  |  |
| *PTOV1* | -1,31554932 | 8,21E-06 |  |  |  |  |
| *NFATC4* | -0,9416822 | 8,11E-05 |  |  |  |  |
| *DCTN1* | -0,88414555 | 0,000275093 |  |  |  |  |
| *PLD2* | -1,14546072 | 0,000120695 |  |  |  |  |
| *AP5Z1* | -1,2646444 | 3,33E-05 |  |  |  |  |
| *SPAG7* | -1,21284832 | 1,52E-05 |  |  |  |  |
| *SH2B1* | -0,85480737 | 0,000884892 |  |  |  |  |
| *ANKS3* | -1,13753097 | 3,54E-05 |  |  |  |  |
| *REXO1* | -0,93463379 | 4,67E-05 |  |  |  |  |
| *TBX2* | -1,17656973 | 7,37E-06 |  |  |  |  |
| *SCLY* | -1,12611367 | 0,000190008 |  |  |  |  |
| *SH2D3C* | -1,46047403 | 2,02E-07 |  |  |  |  |
| *UNC45A* | -0,75302498 | 0,000331554 |  |  |  |  |
| *GRAMD4* | -1,41692856 | 0,000170774 |  |  |  |  |
| *APBB3* | -0,89080283 | 0,000311957 |  |  |  |  |
| *PEX6* | -1,1789228 | 1,01E-05 |  |  |  |  |
| *RPS6KL1* | -1,60673152 | 9,59E-05 |  |  |  |  |
| *KAT5* | -0,9737845 | 0,000401585 |  |  |  |  |
| *TNNT3* | -2,84558555 | 6,81E-11 |  |  |  |  |
| *COL15A1* | -1,23822677 | 1,72E-07 |  |  |  |  |
| *IQSEC1* | -1,12185588 | 0,000823519 |  |  |  |  |
| *DHX30* | -0,9568651 | 4,81E-05 |  |  |  |  |
| *ESPNL* | -1,74519161 | 4,63E-09 |  |  |  |  |
| *MYL4* | -1,74158051 | 0,000268414 |  |  |  |  |
| *GTF3C1* | -1,13532225 | 2,39E-05 |  |  |  |  |
| *SIRT5* | -1,15843474 | 0,000472083 |  |  |  |  |
| *SIGIRR* | -1,37999874 | 5,37E-05 |  |  |  |  |
| *ITGB4* | -1,30844474 | 0,000270999 |  |  |  |  |
| *LDB3* | -1,80863738 | 0,000900866 |  |  |  |  |
| *ARHGEF2* | -0,94610624 | 4,98E-05 |  |  |  |  |
| *ASB6* | -1,1931822 | 0,000136489 |  |  |  |  |
| *ITIH4* | -1,12942812 | 5,33E-05 |  |  |  |  |
| *ATP13A1* | -0,92497788 | 5,89E-05 |  |  |  |  |
| *TFDP1* | -1,17560786 | 4,17E-05 |  |  |  |  |
| *SH3BP2* | -1,37203618 | 5,43E-06 |  |  |  |  |
| *ZNF608* | -1,08816042 | 0,000130832 |  |  |  |  |
| *NCLN* | -0,9571327 | 0,000258445 |  |  |  |  |
| *TNK2* | -0,77831603 | 0,000437107 |  |  |  |  |
| *CLIP2* | -1,26135865 | 4,22E-05 |  |  |  |  |
| *TIE1* | -0,99390432 | 0,000414731 |  |  |  |  |
| *NFATC2IP* | -1,51639513 | 1,08E-05 |  |  |  |  |
| *BCL6B* | -1,06943611 | 0,000930589 |  |  |  |  |
| *LAMB3* | -1,28093865 | 4,21E-05 |  |  |  |  |
| *SLC26A10* | -1,12660805 | 0,000331726 |  |  |  |  |
| *SLC6A1* | -1,10109209 | 0,000940951 |  |  |  |  |
| *LOX* | 2,375186691 | 4,38E-20 |  |  |  |  |
| *A2M* | 3,027405525 | 4,76E-24 |  |  |  |  |
| *SEMA3C* | 2,723697174 | 4,81E-20 |  |  |  |  |
| *PCOLCE2* | 2,899469744 | 1,09E-19 |  |  |  |  |
| *OLFML1* | 2,207502334 | 4,10E-18 |  |  |  |  |
| *PLXDC2* | 0,825590758 | 8,07E-05 |  |  |  |  |
| *C3* | 2,894975981 | 9,87E-18 |  |  |  |  |
| *GNG2* | 1,253500417 | 4,05E-05 |  |  |  |  |
| *MFAP5* | 2,253560907 | 4,35E-10 |  |  |  |  |
| *C1R* | 2,261563238 | 4,11E-16 |  |  |  |  |
| *RNASE4* | 1,802594173 | 3,37E-13 |  |  |  |  |
| *PDGFRA* | 1,901504301 | 7,96E-16 |  |  |  |  |
| *TWSG1* | 1,524568242 | 4,51E-10 |  |  |  |  |
| *GFPT2* | 1,51439053 | 3,85E-08 |  |  |  |  |
| *SELENBP1* | 1,822406585 | 4,41E-13 |  |  |  |  |
| *CST3* | 2,501413857 | 1,88E-14 |  |  |  |  |
| *ADGRD1* | 1,361385982 | 1,46E-05 |  |  |  |  |
| *IL1RL1* | 2,289690296 | 1,32E-10 |  |  |  |  |
| *IL13RA1* | 1,697189128 | 5,76E-13 |  |  |  |  |
| *RARRES1* | 2,566445557 | 9,79E-09 |  |  |  |  |
| *CTSV* | 2,20227034 | 1,31E-14 |  |  |  |  |
| *GDA* | 1,380547357 | 2,41E-05 |  |  |  |  |
| *CD164* | 2,252446751 | 4,46E-13 |  |  |  |  |
| *SERPING1* | 2,16955411 | 6,51E-13 |  |  |  |  |
| *CIDEC* | 1,770580323 | 2,91E-11 |  |  |  |  |
| *EPHX1* | 2,127788448 | 3,98E-15 |  |  |  |  |
| *PHYH* | 2,078324492 | 4,53E-11 |  |  |  |  |
| *EFEMP1* | 2,101793993 | 3,13E-08 |  |  |  |  |
| *TMEM254* | 2,390355947 | 1,03E-12 |  |  |  |  |
| *CTSK* | 1,634366234 | 6,98E-11 |  |  |  |  |
| *TNFAIP6* | 2,080375399 | 1,43E-09 |  |  |  |  |
| *DAD1* | 1,551249033 | 7,60E-09 |  |  |  |  |
| *ENPP1* | 1,450619464 | 1,18E-07 |  |  |  |  |
| *LOC508666* | 1,753373696 | 3,02E-10 |  |  |  |  |
| *CAT* | 1,63868814 | 1,31E-10 |  |  |  |  |
| *GLCE* | 2,284432522 | 1,45E-09 |  |  |  |  |
| *SCARA5* | 1,231616437 | 1,59E-09 |  |  |  |  |
| *FBLN5* | 1,639942691 | 7,25E-07 |  |  |  |  |
| *IGFBP3* | 2,134902583 | 1,18E-08 |  |  |  |  |
| *LAMP2* | 1,4198083 | 9,63E-09 |  |  |  |  |
| *LAPTM4A* | 1,667748495 | 8,71E-09 |  |  |  |  |
| *FSTL1* | 1,839270785 | 1,51E-08 |  |  |  |  |
| *TGFBI* | 1,557461778 | 1,66E-05 |  |  |  |  |
| *AOX1* | 1,11883013 | 4,76E-05 |  |  |  |  |
| *CFB* | 2,486303682 | 2,49E-08 |  |  |  |  |
| *CPA3* | 3,64455293 | 1,51E-14 |  |  |  |  |
| *TIMP2* | 1,206425177 | 4,48E-06 |  |  |  |  |
| *FGL2* | 1,869692729 | 3,13E-08 |  |  |  |  |
| *FGG* | 2,433785409 | 2,64E-11 |  |  |  |  |
| *CDO1* | 1,729337072 | 1,10E-07 |  |  |  |  |
| *NTRK2* | 1,401437852 | 2,22E-06 |  |  |  |  |
| *FUCA2* | 2,179152139 | 1,44E-09 |  |  |  |  |
| *CLU* | 2,171270007 | 3,29E-08 |  |  |  |  |
| *ACSM1* | 1,21335522 | 6,98E-06 |  |  |  |  |
| *CD63* | 1,403732912 | 1,30E-07 |  |  |  |  |
| *TSPAN3* | 1,368021642 | 2,58E-06 |  |  |  |  |
| *CPNE3* | 1,272989162 | 1,94E-06 |  |  |  |  |
| *ECM1* | 2,159105143 | 1,97E-08 |  |  |  |  |
| *ITM2B* | 1,672672537 | 8,58E-08 |  |  |  |  |
| *MMP2* | 1,683976585 | 3,72E-07 |  |  |  |  |
| *SFRP4* | 1,94156979 | 4,31E-07 |  |  |  |  |
| *AKR1C4* | 1,204464869 | 0,000571707 |  |  |  |  |
| *ACKR3* | 1,58358661 | 2,20E-06 |  |  |  |  |
| *CREG1* | 1,442322742 | 2,88E-07 |  |  |  |  |
| *THY1* | 2,040577225 | 1,95E-07 |  |  |  |  |
| *TXNDC12* | 1,427649663 | 3,47E-06 |  |  |  |  |
| *GJA1* | 1,594042228 | 8,77E-07 |  |  |  |  |
| *SERPINF1* | 2,451032838 | 1,87E-07 |  |  |  |  |
| *TSPAN6* | 1,528124611 | 2,50E-06 |  |  |  |  |
| *CFD* | 1,777321001 | 8,87E-07 |  |  |  |  |
| *NID1* | 1,80967032 | 1,34E-06 |  |  |  |  |
| *C2* | 1,870666508 | 4,13E-07 |  |  |  |  |
| *TLR4* | 1,21137581 | 6,83E-06 |  |  |  |  |
| *C5AR2* | 1,866335758 | 1,71E-06 |  |  |  |  |
| *MIR421* | 2,211397552 | 4,99E-07 |  |  |  |  |
| *CCL24* | 1,612985313 | 1,82E-06 |  |  |  |  |
| *DAB2* | 1,230851746 | 7,32E-05 |  |  |  |  |
| *SUOX* | 1,621179652 | 1,29E-06 |  |  |  |  |
| *MFSD1* | 1,33841103 | 2,26E-06 |  |  |  |  |
| *SOD3* | 1,234148723 | 0,000653912 |  |  |  |  |
| *RNASE6* | 1,879833626 | 1,51E-06 |  |  |  |  |
| *RBPJ* | 1,140505139 | 2,12E-06 |  |  |  |  |
| *MMRN1* | 3,244632672 | 2,77E-18 |  |  |  |  |
| *CTSF* | 1,282113105 | 4,06E-06 |  |  |  |  |
| *FBLN1* | 1,260985513 | 5,88E-05 |  |  |  |  |
| *F2RL2* | 1,589107729 | 1,75E-06 |  |  |  |  |
| *AOC1* | 2,222936091 | 5,21E-06 |  |  |  |  |
| *MGP* | 1,4016781 | 4,55E-06 |  |  |  |  |
| *BRB* | 1,943674815 | 3,64E-06 |  |  |  |  |
| *VNN1* | 1,905421613 | 6,34E-10 |  |  |  |  |
| *KIT* | 2,063569246 | 1,37E-09 |  |  |  |  |
| *SLC31A1* | 1,227247467 | 2,94E-06 |  |  |  |  |
| *DENND4A* | 1,085060934 | 1,85E-05 |  |  |  |  |
| *RPN2* | 1,318569921 | 3,35E-05 |  |  |  |  |
| *LRRN4CL* | 1,894085647 | 3,94E-06 |  |  |  |  |
| *DHRS7* | 1,515371223 | 1,18E-06 |  |  |  |  |
| *GINM1* | 1,010679697 | 2,54E-05 |  |  |  |  |
| *GNS* | 1,34138113 | 3,43E-06 |  |  |  |  |
| *HADHB* | 1,139599294 | 2,27E-06 |  |  |  |  |
| *B3GNT9* | 1,622395272 | 6,44E-06 |  |  |  |  |
| *GSN* | 1,642061499 | 4,75E-06 |  |  |  |  |
| *STT3A* | 1,253254929 | 5,58E-06 |  |  |  |  |
| *PSENEN* | 1,419499184 | 4,07E-06 |  |  |  |  |
| *C5AR1* | 2,102909721 | 1,12E-06 |  |  |  |  |
| *NT5E* | 1,793079407 | 2,04E-07 |  |  |  |  |
| *TIMP1* | 1,67515834 | 1,26E-05 |  |  |  |  |
| *MAN2B1* | 1,239416964 | 2,28E-05 |  |  |  |  |
| *FMOD* | 1,879616789 | 3,62E-06 |  |  |  |  |
| *FOLH1B* | 2,282970139 | 1,78E-13 |  |  |  |  |
| *KLB* | 2,077612332 | 2,60E-05 |  |  |  |  |
| *CD86* | 1,928525804 | 1,08E-07 |  |  |  |  |
| *CDHR4* | 1,521399666 | 5,70E-05 |  |  |  |  |
| *SRPX2* | 1,132045365 | 8,58E-05 |  |  |  |  |
| *SLC39A1* | 1,583156057 | 2,50E-05 |  |  |  |  |
| *CD52* | 2,147441613 | 9,19E-06 |  |  |  |  |
| *MPEG1* | 1,870747631 | 3,93E-05 |  |  |  |  |
| *BOLA-DRA* | 1,765926382 | 3,74E-05 |  |  |  |  |
| *C3AR1* | 1,715855395 | 1,24E-05 |  |  |  |  |
| *ENPP5* | 2,013749211 | 1,83E-06 |  |  |  |  |
| *HIF1A* | 1,117783666 | 0,001145313 |  |  |  |  |
| *PROS1* | 1,25853888 | 0,0001469 |  |  |  |  |
| *CD59* | 1,600006237 | 1,97E-09 |  |  |  |  |
| *GPD2* | 0,964102486 | 4,58E-05 |  |  |  |  |
| *LUM* | 1,408668884 | 9,73E-05 |  |  |  |  |
| *CRISPLD2* | 1,216003242 | 7,70E-05 |  |  |  |  |
| *CD302* | 1,270632731 | 0,000585794 |  |  |  |  |
| *CD99* | 1,228829219 | 1,09E-05 |  |  |  |  |
| *OS9* | 0,873924774 | 0,000587611 |  |  |  |  |
| *PAMR1* | 1,670892636 | 0,000707865 |  |  |  |  |
| *SQSTM1* | 0,96034847 | 4,90E-05 |  |  |  |  |
| *QSOX1* | 1,273323208 | 3,22E-06 |  |  |  |  |
| *C7* | 1,884272832 | 5,92E-05 |  |  |  |  |
| *FKBP7* | 1,687510369 | 4,57E-06 |  |  |  |  |
| *ACO1* | 1,104452046 | 3,65E-05 |  |  |  |  |
| *CD163* | 1,471507968 | 0,000462888 |  |  |  |  |
| *F13A1* | 1,507610264 | 0,000121836 |  |  |  |  |
| *THBD* | 1,498293044 | 0,000159454 |  |  |  |  |
| *NUCB1* | 1,028630992 | 0,000175415 |  |  |  |  |
| *GPR137B* | 0,860565619 | 0,001254821 |  |  |  |  |
| *ALDH1L2* | 1,049632857 | 0,000349542 |  |  |  |  |
| *MESDC2* | 0,967562144 | 0,000146676 |  |  |  |  |
| *SUMF1* | 1,534738548 | 4,19E-06 |  |  |  |  |
| *ATP13A3* | 1,145671158 | 0,001167958 |  |  |  |  |
| *TMED9* | 1,105068559 | 0,000238508 |  |  |  |  |
