# Supplementary material for: Impact of the severity of negative energy balance on gene expression in the subcutaneous adipose tissue of periparturient primiparous Holstein dairy cows: Identification of potential novel metabolic signals for the reproductive system
Source: PLoS One. 2019 Sep 26;14(9):e0222954. doi: 10.1371/journal.pone.0222954 (PMC6763198; doi:10.1371/journal.pone.0222954)
Supplement: S9 Table — (DOCX) [file pone.0222954.s014.docx]

| **S9 Table: Genes differentially expressed between MNEB and SNEB animals at 16 WKPP** | | | | | | |
| --- | --- | --- | --- | --- | --- | --- |
| name | log2FoldChange_exons | pvalue_exons | |  |  |  |
| *EPHA2* | -1,9372144 | 1,07E-25 |  |  |  |  |
| *NOS3* | -1,6998797 | 8,64E-18 |  |  |  |  |
| *MIR143* | -3,0096286 | 5,74E-16 |  |  |  |  |
| *RASSF3* | -1,4823342 | 1,01E-15 |  |  |  |  |
| *HCRTR1* | -2,0367338 | 1,21E-15 |  |  |  |  |
| *ATP1A2* | -2,2423744 | 3,39E-15 |  |  |  |  |
| *LGI4* | -1,7727952 | 6,75E-14 |  |  |  |  |
| *FBXW5* | -1,5990893 | 7,86E-14 |  |  |  |  |
| *RCAN2* | -2,1388188 | 8,71E-14 |  |  |  |  |
| *MAPK11* | -2,4966871 | 2,19E-13 |  |  |  |  |
| *LMNA* | -1,6510492 | 3,88E-13 |  |  |  |  |
| *URB1* | -1,2638391 | 4,40E-12 |  |  |  |  |
| *EDNRA* | -1,4913506 | 4,51E-12 |  |  |  |  |
| *FHL2* | -1,3958073 | 5,18E-12 |  |  |  |  |
| *HABP4* | -1,440933 | 1,46E-11 |  |  |  |  |
| *PHLDB3* | -1,6834811 | 1,74E-11 |  |  |  |  |
| *NR2F2* | -1,3743557 | 4,52E-11 |  |  |  |  |
| *KCTD10* | -1,2840172 | 6,53E-11 |  |  |  |  |
| *ARVCF* | -2,2690526 | 7,41E-11 |  |  |  |  |
| *CRIM1* | -1,408704 | 7,93E-11 |  |  |  |  |
| *FADS3* | -1,60057 | 1,62E-10 |  |  |  |  |
| *HSPA12B* | -0,9895366 | 3,43E-10 |  |  |  |  |
| *SMARCD3* | -1,4307251 | 6,58E-10 |  |  |  |  |
| *SH3RF1* | -1,5278114 | 1,19E-09 |  |  |  |  |
| *MFSD10* | -1,2616966 | 1,30E-09 |  |  |  |  |
| *CDH19* | -2,0429399 | 1,73E-09 |  |  |  |  |
| *NR4A1* | -1,970096 | 1,94E-09 |  |  |  |  |
| *FBXL22* | -1,8794207 | 3,02E-09 |  |  |  |  |
| *KIF1C* | -1,148927 | 3,68E-09 |  |  |  |  |
| *RPS25* | -1,5717766 | 5,69E-09 |  |  |  |  |
| *EBI3* | -1,7655574 | 5,70E-09 |  |  |  |  |
| *DYNLL2* | -1,3938286 | 6,99E-09 |  |  |  |  |
| *GRIK3* | -1,6691972 | 7,40E-09 |  |  |  |  |
| *ARHGEF25* | -0,9991702 | 7,58E-09 |  |  |  |  |
| *CDC42BPG* | -1,4717026 | 9,23E-09 |  |  |  |  |
| *STAC* | -1,67664 | 9,51E-09 |  |  |  |  |
| *RRP12* | -1,1446349 | 1,11E-08 |  |  |  |  |
| *PACSIN2* | -1,0079509 | 1,15E-08 |  |  |  |  |
| *LBH* | -1,3323263 | 1,29E-08 |  |  |  |  |
| *MYOM1* | -1,4171759 | 1,41E-08 |  |  |  |  |
| *RDH13* | -1,4905078 | 1,87E-08 |  |  |  |  |
| *RANGAP1* | -1,208285 | 2,15E-08 |  |  |  |  |
| *PKIG* | -1,3825366 | 2,90E-08 |  |  |  |  |
| *DHX16* | -1,1375221 | 3,00E-08 |  |  |  |  |
| *TBL3* | -1,2709904 | 3,12E-08 |  |  |  |  |
| *C16H1orf95* | -2,0280611 | 3,15E-08 |  |  |  |  |
| *TNC* | -2,2295513 | 3,40E-08 |  |  |  |  |
| *KCTD15* | -1,6548902 | 3,43E-08 |  |  |  |  |
| *RRP8* | -1,2679309 | 3,45E-08 |  |  |  |  |
| *CCND1* | -2,1832836 | 3,48E-08 |  |  |  |  |
| *TRAF3* | -1,513143 | 3,94E-08 |  |  |  |  |
| *KATNB1* | -1,4350111 | 5,07E-08 |  |  |  |  |
| *TPPP* | -1,0914298 | 5,11E-08 |  |  |  |  |
| *KIAA1462* | -0,9087262 | 8,31E-08 |  |  |  |  |
| *FOXP4* | -1,4297938 | 8,56E-08 |  |  |  |  |
| *GMFB* | -1,5238383 | 8,56E-08 |  |  |  |  |
| *NRGN* | -2,0999169 | 8,58E-08 |  |  |  |  |
| *RERG* | -1,228835 | 1,40E-07 |  |  |  |  |
| *ATP8B1* | -0,9345585 | 1,52E-07 |  |  |  |  |
| *C3H1orf226* | -1,6255387 | 1,71E-07 |  |  |  |  |
| *BREH1* | -1,3429719 | 1,89E-07 |  |  |  |  |
| *ANGPT1* | -1,388125 | 1,90E-07 |  |  |  |  |
| *TRIM3* | -1,0157308 | 2,18E-07 |  |  |  |  |
| *N4BP3* | -1,6776525 | 2,47E-07 |  |  |  |  |
| *PPP1R3C* | -1,0371783 | 2,57E-07 |  |  |  |  |
| *ADRA1D* | -1,6750006 | 2,78E-07 |  |  |  |  |
| *TNFAIP2* | -1,3100647 | 2,78E-07 |  |  |  |  |
| *PLEKHA4* | -1,2372778 | 2,83E-07 |  |  |  |  |
| *TRIM56* | -1,0067162 | 3,04E-07 |  |  |  |  |
| *SLC2A4* | -1,3895746 | 4,44E-07 |  |  |  |  |
| *SYNPO* | -1,1271677 | 4,73E-07 |  |  |  |  |
| *GLIS2* | -1,3103975 | 5,05E-07 |  |  |  |  |
| *GAS2L2* | -2,06477 | 5,14E-07 |  |  |  |  |
| *RPS23* | -1,865035 | 5,31E-07 |  |  |  |  |
| *MYLK* | -1,4963979 | 6,40E-07 |  |  |  |  |
| *BCL2L2* | -0,8902439 | 6,58E-07 |  |  |  |  |
| *PC* | -1,5349815 | 8,51E-07 |  |  |  |  |
| *IDI1* | -1,4965404 | 8,92E-07 |  |  |  |  |
| *NFKB2* | -1,3845845 | 9,27E-07 |  |  |  |  |
| *L1CAM* | -1,8462209 | 9,77E-07 |  |  |  |  |
| *TRPC6* | -1,3742941 | 1,18E-06 |  |  |  |  |
| *ERBB3* | -1,8207793 | 1,24E-06 |  |  |  |  |
| *TGFB1I1* | -1,1658582 | 1,24E-06 |  |  |  |  |
| *CBS* | -1,3586171 | 1,45E-06 |  |  |  |  |
| *NES* | -1,3855856 | 1,57E-06 |  |  |  |  |
| *GPRC5C* | -1,3669885 | 1,82E-06 |  |  |  |  |
| *PDE4C* | -2,2589848 | 1,88E-06 |  |  |  |  |
| *TMEM243* | -1,0321915 | 2,00E-06 |  |  |  |  |
| *BRD9* | -0,8741563 | 2,37E-06 |  |  |  |  |
| *DCAF15* | -0,8897073 | 2,69E-06 |  |  |  |  |
| *CHSY3* | -1,2611265 | 2,79E-06 |  |  |  |  |
| *RPS13* | -1,6928614 | 2,82E-06 |  |  |  |  |
| *MARK1* | -0,9639292 | 3,43E-06 |  |  |  |  |
| *MAMSTR* | -1,1157065 | 3,59E-06 |  |  |  |  |
| *DLL4* | -1,2221081 | 3,73E-06 |  |  |  |  |
| *TNFAIP8L1* | -1,1470062 | 3,85E-06 |  |  |  |  |
| *TPM2* | -1,6394829 | 3,98E-06 |  |  |  |  |
| *CGN* | -1,5319978 | 4,47E-06 |  |  |  |  |
| *KCNC4* | -1,1734625 | 4,50E-06 |  |  |  |  |
| *P3H2* | -1,1122965 | 4,87E-06 |  |  |  |  |
| *HDAC10* | -1,314021 | 5,29E-06 |  |  |  |  |
| *GIPC3* | -2,3528382 | 5,43E-06 |  |  |  |  |
| *SMG5* | -0,829666 | 5,87E-06 |  |  |  |  |
| *SLC4A3* | -1,4405111 | 6,28E-06 |  |  |  |  |
| *ITGA3* | -1,2420278 | 6,69E-06 |  |  |  |  |
| *TMEM145* | -1,7473172 | 7,13E-06 |  |  |  |  |
| *SMARCB1* | -1,3705502 | 7,29E-06 |  |  |  |  |
| *GGT1* | -2,0733145 | 8,74E-06 |  |  |  |  |
| *CDC42EP1* | -0,9274908 | 9,80E-06 |  |  |  |  |
| *FAM198B* | -1,4760747 | 9,88E-06 |  |  |  |  |
| *MTMR7* | -1,307053 | 1,12E-05 |  |  |  |  |
| *PKIA* | -1,5432553 | 1,26E-05 |  |  |  |  |
| *EPB41L4A* | -0,7709137 | 1,41E-05 |  |  |  |  |
| *UBAC1* | -0,9392137 | 1,43E-05 |  |  |  |  |
| *ABCF1* | -0,8330867 | 1,45E-05 |  |  |  |  |
| *TNFRSF11B* | -2,5755632 | 1,46E-05 |  |  |  |  |
| *PLK3* | -1,3094187 | 1,57E-05 |  |  |  |  |
| *C7H19orf24* | -1,1611288 | 1,61E-05 |  |  |  |  |
| *HSPA4* | -1,1269788 | 1,72E-05 |  |  |  |  |
| *PLCD1* | -0,9418895 | 1,77E-05 |  |  |  |  |
| *LONP1* | -0,8304193 | 1,80E-05 |  |  |  |  |
| *ARHGAP10* | -1,1544563 | 2,02E-05 |  |  |  |  |
| *FOXS1* | -1,0082599 | 2,20E-05 |  |  |  |  |
| *SLC12A4* | -0,8014125 | 2,27E-05 |  |  |  |  |
| *LMCD1* | -0,9482164 | 2,28E-05 |  |  |  |  |
| *NAT9* | -1,1094439 | 2,29E-05 |  |  |  |  |
| *CDK18* | -1,5142141 | 2,58E-05 |  |  |  |  |
| *WTIP* | -1,0673963 | 2,69E-05 |  |  |  |  |
| *DNAJB5* | -1,4380528 | 2,72E-05 |  |  |  |  |
| *SDC1* | -1,4438907 | 2,75E-05 |  |  |  |  |
| *ADRA2B* | -1,2421321 | 3,08E-05 |  |  |  |  |
| *RPS28* | -0,8530858 | 3,19E-05 |  |  |  |  |
| *STK32A* | -2,0598664 | 3,67E-05 |  |  |  |  |
| *FARSA* | -0,9030187 | 3,69E-05 |  |  |  |  |
| *SOX10* | -1,7448971 | 3,69E-05 |  |  |  |  |
| *TRAPPC9* | -1,1725813 | 3,75E-05 |  |  |  |  |
| *SPRY4* | -1,2203225 | 3,82E-05 |  |  |  |  |
| *PTH1R* | -1,2085196 | 3,82E-05 |  |  |  |  |
| *GRIK5* | -1,4720795 | 4,45E-05 |  |  |  |  |
| *GNL1* | -1,0086787 | 4,56E-05 |  |  |  |  |
| *CARS2* | -0,9937756 | 4,58E-05 |  |  |  |  |
| *PPAN* | -1,12221 | 4,69E-05 |  |  |  |  |
| *MAP1B* | -0,8454168 | 4,89E-05 |  |  |  |  |
| *PPRC1* | -0,8677941 | 4,95E-05 |  |  |  |  |
| *CHMP4C* | -1,597684 | 5,18E-05 |  |  |  |  |
| *RGS11* | -2,3290878 | 5,25E-05 |  |  |  |  |
| *LRAT* | -1,1627931 | 5,33E-05 |  |  |  |  |
| *NACC2* | -1,09803 | 5,47E-05 |  |  |  |  |
| *ITGA10* | -1,1244987 | 5,83E-05 |  |  |  |  |
| *ASMTL* | -0,865044 | 7,05E-05 |  |  |  |  |
| *PGM2L1* | -0,9771562 | 7,29E-05 |  |  |  |  |
| *MIR145* | -1,5374971 | 7,58E-05 |  |  |  |  |
| *HDAC5* | -0,848765 | 7,70E-05 |  |  |  |  |
| *AMH* | -2,0080459 | 7,80E-05 |  |  |  |  |
| *ALDH16A1* | -0,7719103 | 7,86E-05 |  |  |  |  |
| *MICAL1* | -1,004842 | 7,87E-05 |  |  |  |  |
| *FXYD6* | -0,8419074 | 7,98E-05 |  |  |  |  |
| *ZC3H12A* | -1,0080037 | 8,44E-05 |  |  |  |  |
| *TRAK2* | -1,1040166 | 8,58E-05 |  |  |  |  |
| *MYO1D* | -1,0859591 | 9,20E-05 |  |  |  |  |
| *SPATA6L* | -1,2342617 | 9,41E-05 |  |  |  |  |
| *DNAJB2* | -0,778131 | 0,00010626 |  |  |  |  |
| *EHD3* | -1,0539649 | 0,00010887 |  |  |  |  |
| *RNF112* | -1,5868658 | 0,00011125 |  |  |  |  |
| *KCNH2* | -1,8457114 | 0,00011787 |  |  |  |  |
| *SLC25A22* | -1,6401311 | 0,00012245 |  |  |  |  |
| *CHKB* | -1,1585318 | 0,00012276 |  |  |  |  |
| *CDK9* | -0,6474819 | 0,00012289 |  |  |  |  |
| *SOCS3* | -1,2289994 | 0,00012342 |  |  |  |  |
| *BRAT1* | -1,2823777 | 0,00012376 |  |  |  |  |
| *ATAD3A* | -1,0730653 | 0,00012447 |  |  |  |  |
| *VCL* | -0,9574211 | 0,00012882 |  |  |  |  |
| *PNKD* | -0,8916947 | 0,00013011 |  |  |  |  |
| *SCHIP1* | -1,1150732 | 0,0001417 |  |  |  |  |
| *IRF7* | -1,0432055 | 0,00014333 |  |  |  |  |
| *CSGALNACT1* | -0,8478326 | 0,00015335 |  |  |  |  |
| *MAFG* | -0,9318638 | 0,0001571 |  |  |  |  |
| *BMP5* | -1,3318135 | 0,0001693 |  |  |  |  |
| *GPT* | -1,5076586 | 0,00017589 |  |  |  |  |
| *AKAP2* | -0,7400636 | 0,00018275 |  |  |  |  |
| *PRICKLE3* | -1,1092929 | 0,00018432 |  |  |  |  |
| *CCDC130* | -0,7472435 | 0,00018849 |  |  |  |  |
| *RRAD* | -1,4644035 | 0,00019087 |  |  |  |  |
| *HIRIP3* | -0,9475316 | 0,0001936 |  |  |  |  |
| *SYNC* | -1,0467577 | 0,00019776 |  |  |  |  |
| *CAMK1* | -1,0144079 | 0,00020579 |  |  |  |  |
| *COX4I2* | -1,5635509 | 0,00020698 |  |  |  |  |
| *AP3D1* | -0,7239287 | 0,00020705 |  |  |  |  |
| *EGR3* | -1,536504 | 0,000217 |  |  |  |  |
| *DHRS11* | -0,9366369 | 0,00022054 |  |  |  |  |
| *SORBS2* | -1,3662178 | 0,00022438 |  |  |  |  |
| *MAFF* | -1,5009484 | 0,00023603 |  |  |  |  |
| *NR1H2* | -0,5976659 | 0,0002392 |  |  |  |  |
| *NAPA* | -0,6480875 | 0,00024854 |  |  |  |  |
| *MRGPRF* | -1,1992532 | 0,00027081 |  |  |  |  |
| *RBPMS* | -1,1783027 | 0,00028142 |  |  |  |  |
| *FAM19A5* | -1,6214376 | 0,00030364 |  |  |  |  |
| *NELFE* | -0,7396048 | 0,00032273 |  |  |  |  |
| *PCDH12* | -1,091313 | 0,00035521 |  |  |  |  |
| *TMEM86B* | -1,090392 | 0,00035788 |  |  |  |  |
| *KANK2* | -0,6868092 | 0,00036591 |  |  |  |  |
| *NXN* | -0,7424955 | 0,00037315 |  |  |  |  |
| *ZNF74* | -1,0504591 | 0,00037647 |  |  |  |  |
| *HTATSF1* | -0,744117 | 0,0003801 |  |  |  |  |
| *GJC1* | -1,091108 | 0,00038411 |  |  |  |  |
| *ARFGAP2* | -0,6941843 | 0,00038712 |  |  |  |  |
| *RPL5* | -1,1310526 | 0,00039234 |  |  |  |  |
| *NDUFA4L2* | -1,6263911 | 0,0004118 |  |  |  |  |
| *FLNB* | -0,9289674 | 0,00041554 |  |  |  |  |
| *RAB3C* | -1,3400093 | 0,00042552 |  |  |  |  |
| *FHL5* | -1,3668476 | 0,00043518 |  |  |  |  |
| *FGF1* | -0,9922814 | 0,00044575 |  |  |  |  |
| *TBXA2R* | -1,0380428 | 0,00045197 |  |  |  |  |
| *CYSLTR2* | -1,1242065 | 0,00045758 |  |  |  |  |
| *HBB* | -1,7364173 | 0,00047146 |  |  |  |  |
| *KCNAB1* | -1,8267819 | 0,00047694 |  |  |  |  |
| *SRM* | -0,8572668 | 0,00047729 |  |  |  |  |
| *ADRA1A* | -1,3646032 | 0,0004788 |  |  |  |  |
| *LOC524810* | -1,9062652 | 0,00048716 |  |  |  |  |
| *RAB40C* | -0,7138463 | 0,00049712 |  |  |  |  |
| *ISYNA1* | -1,033275 | 0,00049744 |  |  |  |  |
| *ADAM23* | -1,3517606 | 0,00053488 |  |  |  |  |
| *PHC1* | -0,7980558 | 0,00057074 |  |  |  |  |
| *RFX2* | -1,2154303 | 0,00060144 |  |  |  |  |
| *TSEN54* | -0,8926502 | 0,00060493 |  |  |  |  |
| *PTGES3* | -0,7176582 | 0,00063286 |  |  |  |  |
| *CSRP1* | -0,9172086 | 0,00063914 |  |  |  |  |
| *BAP1* | -0,5793029 | 0,00066147 |  |  |  |  |
| *MYO1B* | -0,68576 | 0,00066787 |  |  |  |  |
| *APLP1* | -1,011737 | 0,00068777 |  |  |  |  |
| *POLD2* | -0,7184057 | 0,00072557 |  |  |  |  |
| *AARSD1* | -0,6911009 | 0,00073023 |  |  |  |  |
| *ARFRP1* | -0,7148165 | 0,00078766 |  |  |  |  |
| *ARRDC4* | -0,9471025 | 0,00079 |  |  |  |  |
| *ATP8B2* | -0,5689956 | 0,00081134 |  |  |  |  |
| *PLN* | -1,4167648 | 0,00082231 |  |  |  |  |
| *SLC22A17* | -0,7319781 | 0,00088641 |  |  |  |  |
| *TSR1* | -0,6570826 | 0,00091255 |  |  |  |  |
| *MED19* | -0,8633604 | 0,00093377 |  |  |  |  |
| *OTUB1* | -0,7188996 | 0,00094627 |  |  |  |  |
| *RPL34* | -0,9877708 | 0,00097551 |  |  |  |  |
| *MRPS26* | -0,835871 | 0,00098183 |  |  |  |  |
| *ENG* | -0,7991074 | 0,00098984 |  |  |  |  |
| *ENSA* | -0,6061861 | 0,00100431 |  |  |  |  |
| *PURA* | -0,8639182 | 0,00105511 |  |  |  |  |
| *SORBS1* | -0,7139335 | 0,00105662 |  |  |  |  |
| *TMSB4X* | -0,698754 | 0,00106848 |  |  |  |  |
| *C15H11orf96* | -0,8382136 | 0,00111815 |  |  |  |  |
| *S100A14* | -0,9091212 | 0,00112539 |  |  |  |  |
| *NT5C* | -0,8219855 | 0,0012059 |  |  |  |  |
| *HNRNPUL2* | -0,7772552 | 0,00121255 |  |  |  |  |
| *VASP* | -0,650451 | 0,00121937 |  |  |  |  |
| *MTMR4* | -0,7843313 | 0,00123975 |  |  |  |  |
| *GNA12* | -0,6508471 | 0,00130164 |  |  |  |  |
| *EZR* | -0,7231322 | 0,00130248 |  |  |  |  |
| *CHL1* | -1,0256631 | 0,00130965 |  |  |  |  |
| *HMG20B* | -0,6227555 | 0,00135355 |  |  |  |  |
| *FTSJ1* | -0,6553656 | 0,00136261 |  |  |  |  |
| *ASB2* | -1,0677209 | 0,00138867 |  |  |  |  |
| *RNH1* | -1,0380468 | 0,00142464 |  |  |  |  |
| *PES1* | -0,7281317 | 0,00146568 |  |  |  |  |
| *KLHL23* | -1,1944138 | 0,00146738 |  |  |  |  |
| *BOK* | -0,7354282 | 0,00148547 |  |  |  |  |
| *VWF* | -0,6189648 | 0,00151939 |  |  |  |  |
| *TMEM200B* | -0,8492136 | 0,00163073 |  |  |  |  |
| *SYNPO2* | -0,9525205 | 0,00168468 |  |  |  |  |
| *FXYD2* | -1,373352 | 0,00168514 |  |  |  |  |
| *SYNM* | -0,8044385 | 0,00170561 |  |  |  |  |
| *RGS16* | -1,0220779 | 0,0017515 |  |  |  |  |
| *LRRC72* | -1,9500699 | 0,00176586 |  |  |  |  |
| *GUCD1* | -0,7021309 | 0,00182325 |  |  |  |  |
| *MAG* | -1,7271364 | 0,0018267 |  |  |  |  |
| *PDLIM3* | -1,0887459 | 0,00188575 |  |  |  |  |
| *RXRA* | -0,6482972 | 0,00191956 |  |  |  |  |
| *TAGLN* | -1,2198881 | 0,00193943 |  |  |  |  |
| *LACC1* | -0,7258803 | 0,00199486 |  |  |  |  |
| *CSF1R* | 1,92197312 | 1,29E-20 |  |  |  |  |
| *CTSB* | 1,59124264 | 2,03E-17 |  |  |  |  |
| *MSR1* | 2,77277135 | 8,62E-16 |  |  |  |  |
| *CD74* | 2,06255451 | 3,21E-15 |  |  |  |  |
| *NCKAP1L* | 1,877563 | 3,59E-15 |  |  |  |  |
| *PEPD* | 1,71870732 | 8,04E-15 |  |  |  |  |
| *EVI2B* | 2,63696783 | 3,18E-14 |  |  |  |  |
| *FRMD4B* | 1,74953153 | 6,83E-14 |  |  |  |  |
| *HIST1H2BN* | 1,92299046 | 8,97E-14 |  |  |  |  |
| *RAC2* | 1,9946725 | 1,39E-13 |  |  |  |  |
| *SPP1* | 2,55103907 | 2,24E-13 |  |  |  |  |
| *LAPTM5* | 1,92556481 | 2,33E-13 |  |  |  |  |
| *P2RY13* | 3,16196197 | 2,74E-13 |  |  |  |  |
| *CYBB* | 2,15361492 | 4,50E-13 |  |  |  |  |
| *PPT1* | 1,81448933 | 1,05E-12 |  |  |  |  |
| *CTHRC1* | 2,02525126 | 2,90E-12 |  |  |  |  |
| *NLRP3* | 2,19502897 | 3,06E-12 |  |  |  |  |
| *CD209* | 2,22099623 | 9,44E-12 |  |  |  |  |
| *OLFML3* | 2,00407258 | 1,45E-11 |  |  |  |  |
| *PTPRC* | 1,5353714 | 1,52E-11 |  |  |  |  |
| *HTRA1* | 1,4893736 | 1,74E-11 |  |  |  |  |
| *CLEC3B* | 1,46279148 | 2,24E-11 |  |  |  |  |
| *CLIC2* | 1,35243763 | 3,22E-11 |  |  |  |  |
| *LOC407171* | 2,30438907 | 4,66E-11 |  |  |  |  |
| *CD68* | 2,5729508 | 4,74E-11 |  |  |  |  |
| *ITGAM* | 2,15590575 | 5,53E-11 |  |  |  |  |
| *BOLA-DMB* | 1,84232022 | 9,05E-11 |  |  |  |  |
| *ACKR1* | 1,67029495 | 1,32E-10 |  |  |  |  |
| *BOLA-DQA5* | 1,99224366 | 6,01E-10 |  |  |  |  |
| *MFAP2* | 1,7042408 | 6,15E-10 |  |  |  |  |
| *TFEC* | 2,41473624 | 6,80E-10 |  |  |  |  |
| *BOLA-DQA2* | 1,89407273 | 7,76E-10 |  |  |  |  |
| *ENPP2* | 1,48828588 | 8,48E-10 |  |  |  |  |
| *TMEM100* | 1,71074751 | 8,47E-10 |  |  |  |  |
| *SPN* | 2,22166091 | 1,11E-09 |  |  |  |  |
| *SGSH* | 1,36348716 | 1,47E-09 |  |  |  |  |
| *C1H3orf58* | 1,3063378 | 1,50E-09 |  |  |  |  |
| *BPI* | 2,96614595 | 1,58E-09 |  |  |  |  |
| *SEMA3D* | 1,36513122 | 2,67E-09 |  |  |  |  |
| *CTSD* | 1,0697897 | 2,98E-09 |  |  |  |  |
| *TMEM176A* | 1,87505361 | 3,03E-09 |  |  |  |  |
| *CD53* | 1,86822599 | 1,06E-08 |  |  |  |  |
| *RAMP2* | 1,32971968 | 1,34E-08 |  |  |  |  |
| *TLR2* | 2,19373163 | 1,39E-08 |  |  |  |  |
| *PTGDS* | 1,0360781 | 1,44E-08 |  |  |  |  |
| *BOLA-DRB3* | 2,24976556 | 2,37E-08 |  |  |  |  |
| *PLTP* | 1,97844443 | 2,39E-08 |  |  |  |  |
| *TLR6* | 1,43657732 | 3,61E-08 |  |  |  |  |
| *P2RY12* | 2,31425652 | 3,93E-08 |  |  |  |  |
| *SLC38A4* | 2,63324691 | 4,38E-08 |  |  |  |  |
| *FCN1* | 2,10216245 | 4,54E-08 |  |  |  |  |
| *SYK* | 1,44414091 | 4,86E-08 |  |  |  |  |
| *IFI30* | 1,37268935 | 5,64E-08 |  |  |  |  |
| *ELF1* | 0,96482382 | 5,65E-08 |  |  |  |  |
| *TM2D2* | 1,38817217 | 5,91E-08 |  |  |  |  |
| *GLMP* | 1,23613195 | 6,08E-08 |  |  |  |  |
| *AGTRAP* | 1,49669437 | 6,43E-08 |  |  |  |  |
| *GIMAP7* | 1,6011062 | 7,20E-08 |  |  |  |  |
| *APH1A* | 1,0196748 | 7,86E-08 |  |  |  |  |
| *SEMA3A* | 1,85440604 | 9,42E-08 |  |  |  |  |
| *LCP1* | 1,63382981 | 9,81E-08 |  |  |  |  |
| *CCR1* | 2,25014318 | 1,26E-07 |  |  |  |  |
| *CCR5* | 2,72607437 | 1,57E-07 |  |  |  |  |
| *ZNF385B* | 2,47487684 | 1,63E-07 |  |  |  |  |
| *CFP* | 1,49563792 | 1,83E-07 |  |  |  |  |
| *LRRC8D* | 1,40426111 | 1,84E-07 |  |  |  |  |
| *CSTF2T* | 1,10007994 | 1,94E-07 |  |  |  |  |
| *RNASET2* | 1,3763843 | 2,10E-07 |  |  |  |  |
| *LIPA* | 2,01926458 | 2,15E-07 |  |  |  |  |
| *CARD6* | 1,12643571 | 2,34E-07 |  |  |  |  |
| *CTSA* | 1,13499811 | 2,37E-07 |  |  |  |  |
| *FAM49B* | 1,25337826 | 2,52E-07 |  |  |  |  |
| *C1QTNF1* | 1,15683044 | 2,56E-07 |  |  |  |  |
| *MFAP4* | 1,33957794 | 2,82E-07 |  |  |  |  |
| *OLFML2B* | 1,36474944 | 2,86E-07 |  |  |  |  |
| *CD1D* | 1,81533409 | 3,04E-07 |  |  |  |  |
| *AGA* | 1,07067133 | 3,19E-07 |  |  |  |  |
| *PIK3CG* | 0,9895121 | 3,23E-07 |  |  |  |  |
| *CLEC12A* | 2,16769022 | 3,59E-07 |  |  |  |  |
| *CYP27A1* | 1,22642979 | 4,35E-07 |  |  |  |  |
| *FKBP9* | 0,88777143 | 4,78E-07 |  |  |  |  |
| *ABHD4* | 1,10268884 | 4,94E-07 |  |  |  |  |
| *RGS1* | 1,78117897 | 5,79E-07 |  |  |  |  |
| *TDH* | 2,652577 | 6,59E-07 |  |  |  |  |
| *GM2A* | 1,55227769 | 6,68E-07 |  |  |  |  |
| *CD37* | 1,79940582 | 6,98E-07 |  |  |  |  |
| *PI15* | 1,91386994 | 7,57E-07 |  |  |  |  |
| *FCER1A* | 2,42599909 | 8,01E-07 |  |  |  |  |
| *GMFG* | 1,42660042 | 8,45E-07 |  |  |  |  |
| *PDZD8* | 0,80195128 | 8,72E-07 |  |  |  |  |
| *LOC513911* | 2,55887954 | 9,42E-07 |  |  |  |  |
| *ERAP2* | 1,2896486 | 1,09E-06 |  |  |  |  |
| *F5* | 2,32343842 | 1,16E-06 |  |  |  |  |
| *B2M* | 1,1628183 | 1,19E-06 |  |  |  |  |
| *DIRC2* | 1,35271316 | 1,19E-06 |  |  |  |  |
| *MANSC1* | 1,61400848 | 1,20E-06 |  |  |  |  |
| *LGMN* | 1,51901117 | 1,22E-06 |  |  |  |  |
| *IL17RB* | 2,15010245 | 1,27E-06 |  |  |  |  |
| *BOLA-DMA* | 1,43364708 | 1,27E-06 |  |  |  |  |
| *ID2* | 0,80580691 | 1,32E-06 |  |  |  |  |
| *GALM* | 1,29515731 | 1,34E-06 |  |  |  |  |
| *ANGPTL1* | 1,64877235 | 1,35E-06 |  |  |  |  |
| *LYZ* | 2,01781026 | 1,47E-06 |  |  |  |  |
| *PRAF2* | 1,12618149 | 1,70E-06 |  |  |  |  |
| *ICAM1* | 0,95040507 | 1,73E-06 |  |  |  |  |
| *MMP16* | 1,46097141 | 1,84E-06 |  |  |  |  |
| *NCF2* | 2,04169936 | 2,01E-06 |  |  |  |  |
| *NCSTN* | 1,12793567 | 2,12E-06 |  |  |  |  |
| *CMTM7* | 1,03185618 | 2,13E-06 |  |  |  |  |
| *EVI2A* | 1,81283137 | 2,19E-06 |  |  |  |  |
| *TMEM156* | 2,05319914 | 2,21E-06 |  |  |  |  |
| *ACSL1* | 1,00091791 | 2,24E-06 |  |  |  |  |
| *DNAJC3* | 0,87389302 | 2,27E-06 |  |  |  |  |
| *HCLS1* | 1,00788983 | 2,38E-06 |  |  |  |  |
| *TMED2* | 0,91180994 | 3,05E-06 |  |  |  |  |
| *MFAP3* | 1,07399417 | 3,10E-06 |  |  |  |  |
| *TYROBP* | 2,102552 | 3,29E-06 |  |  |  |  |
| *CYP3A4* | 1,74936469 | 3,35E-06 |  |  |  |  |
| *ARRB2* | 1,17758385 | 3,63E-06 |  |  |  |  |
| *ARHGAP20* | 1,13285836 | 3,88E-06 |  |  |  |  |
| *TIMD4* | 2,49442805 | 3,87E-06 |  |  |  |  |
| *IL2RG* | 1,67336577 | 4,01E-06 |  |  |  |  |
| *ARSA* | 1,22581672 | 4,27E-06 |  |  |  |  |
| *LGALS3* | 1,13424345 | 5,34E-06 |  |  |  |  |
| *SLCO2B1* | 0,91255679 | 5,80E-06 |  |  |  |  |
| *CPT2* | 1,01985794 | 6,05E-06 |  |  |  |  |
| *CHI3L1* | 1,76611857 | 6,20E-06 |  |  |  |  |
| *KLK10* | 1,94258312 | 6,32E-06 |  |  |  |  |
| *REL* | 1,24243676 | 6,89E-06 |  |  |  |  |
| *ABCA1* | 1,709302 | 7,30E-06 |  |  |  |  |
| *C1GALT1* | 1,01506256 | 7,55E-06 |  |  |  |  |
| *IFITM3* | 0,92475295 | 7,71E-06 |  |  |  |  |
| *GLIPR1* | 1,5573316 | 7,89E-06 |  |  |  |  |
| *ESR1* | 1,20565861 | 7,96E-06 |  |  |  |  |
| *SIRPB1* | 1,80090866 | 8,93E-06 |  |  |  |  |
| *PITHD1* | 0,82976077 | 9,04E-06 |  |  |  |  |
| *BLA-DQB* | 1,39049903 | 9,18E-06 |  |  |  |  |
| *ARHGAP30* | 1,26052621 | 1,11E-05 |  |  |  |  |
| *NCF1* | 1,83615779 | 1,17E-05 |  |  |  |  |
| *IGF1* | 1,86604747 | 1,23E-05 |  |  |  |  |
| *CMTM6* | 0,89189657 | 1,23E-05 |  |  |  |  |
| *SLC37A2* | 1,60399414 | 1,29E-05 |  |  |  |  |
| *CTNS* | 1,35125095 | 1,53E-05 |  |  |  |  |
| *GUSB* | 1,1407242 | 1,69E-05 |  |  |  |  |
| *PLIN2* | 1,06481262 | 1,70E-05 |  |  |  |  |
| *GPR87* | 2,75112617 | 1,77E-05 |  |  |  |  |
| *TOR3A* | 0,83900226 | 1,82E-05 |  |  |  |  |
| *IL6R* | 0,82447819 | 1,96E-05 |  |  |  |  |
| *CXADR* | 1,31629499 | 2,01E-05 |  |  |  |  |
| *PTGER4* | 1,0455077 | 2,06E-05 |  |  |  |  |
| *TMEM173* | 1,2818625 | 2,09E-05 |  |  |  |  |
| *AQP7* | 1,35289592 | 2,21E-05 |  |  |  |  |
| *MEIS1* | 0,95945835 | 2,32E-05 |  |  |  |  |
| *CYP2U1* | 1,18489006 | 2,37E-05 |  |  |  |  |
| *SCARB2* | 1,04029678 | 2,42E-05 |  |  |  |  |
| *QPCTL* | 1,10015407 | 2,45E-05 |  |  |  |  |
| *PTPLAD2* | 1,38706244 | 2,75E-05 |  |  |  |  |
| *FCGR3A* | 1,69549548 | 2,91E-05 |  |  |  |  |
| *B4GALT1* | 0,88512265 | 3,27E-05 |  |  |  |  |
| *LY9* | 1,87064097 | 3,28E-05 |  |  |  |  |
| *ABHD3* | 1,4685985 | 3,40E-05 |  |  |  |  |
| *ATP6V1D* | 0,81280878 | 3,54E-05 |  |  |  |  |
| *SLC23A2* | 0,98551111 | 3,72E-05 |  |  |  |  |
| *THRSP* | 1,4489097 | 4,29E-05 |  |  |  |  |
| *MR1* | 1,4019455 | 4,63E-05 |  |  |  |  |
| *GLB1L* | 0,88857893 | 5,37E-05 |  |  |  |  |
| *ARRDC3* | 1,03016 | 5,39E-05 |  |  |  |  |
| *FYB* | 1,60662351 | 7,00E-05 |  |  |  |  |
| *SDF2* | 0,97362847 | 7,05E-05 |  |  |  |  |
| *TGFBR2* | 0,82506191 | 7,22E-05 |  |  |  |  |
| *PLD1* | 1,00695248 | 7,75E-05 |  |  |  |  |
| *ANPEP* | 1,25842513 | 7,96E-05 |  |  |  |  |
| *GSTT2* | 1,6350974 | 8,03E-05 |  |  |  |  |
| *SPI1* | 1,28403791 | 8,05E-05 |  |  |  |  |
| *NFATC3* | 0,68773248 | 8,16E-05 |  |  |  |  |
| *DSC2* | 1,96032268 | 8,69E-05 |  |  |  |  |
| *EHHADH* | 1,26712303 | 8,93E-05 |  |  |  |  |
| *TLR8* | 1,60074592 | 9,02E-05 |  |  |  |  |
| *SAMSN1* | 1,28534841 | 0,0001125 |  |  |  |  |
| *ATP6V0C* | 0,77890818 | 0,00011404 |  |  |  |  |
| *HAS2* | 1,15664177 | 0,00011535 |  |  |  |  |
| *CASP8* | 0,83690818 | 0,00011701 |  |  |  |  |
| *HNMT* | 1,36335644 | 0,00011923 |  |  |  |  |
| *CD48* | 1,62674941 | 0,00012853 |  |  |  |  |
| *CENPF* | 1,85286788 | 0,00013658 |  |  |  |  |
| *TFPI2* | 1,23164995 | 0,00013805 |  |  |  |  |
| *NFE2L2* | 0,87150531 | 0,0001384 |  |  |  |  |
| *LOC515418* | 1,74303212 | 0,00014229 |  |  |  |  |
| *GDPD1* | 1,41911131 | 0,00014546 |  |  |  |  |
| *PDGFRL* | 1,06757275 | 0,00014881 |  |  |  |  |
| *TP53INP1* | 1,3714376 | 0,00015979 |  |  |  |  |
| *MCM6* | 0,75672681 | 0,00016237 |  |  |  |  |
| *PRKCQ* | 1,08716185 | 0,00017169 |  |  |  |  |
| *SESN1* | 0,87098495 | 0,00017726 |  |  |  |  |
| *KLF3* | 0,79481588 | 0,00019646 |  |  |  |  |
| *FNDC3A* | 0,70047166 | 0,00021136 |  |  |  |  |
| *NCOA1* | 0,63474193 | 0,00024737 |  |  |  |  |
| *LPXN* | 1,36479952 | 0,00025089 |  |  |  |  |
| *ADAM9* | 0,81324946 | 0,00026752 |  |  |  |  |
| *SCIN* | 1,31628039 | 0,00027276 |  |  |  |  |
| *SIRPA* | 1,08902549 | 0,000303 |  |  |  |  |
| *KLF5* | 1,1299439 | 0,00042074 |  |  |  |  |
| *GLRX* | 0,98556589 | 0,0004562 |  |  |  |  |
| *ZNRF2* | 0,8437508 | 0,00048288 |  |  |  |  |
| *RBM47* | 1,22529875 | 0,00051323 |  |  |  |  |
| *CD200R1L* | 1,55998185 | 0,00054308 |  |  |  |  |
| *LRP12* | 0,96790422 | 0,00055412 |  |  |  |  |
| *PYROXD2* | 1,59796536 | 0,00059872 |  |  |  |  |
| *LCP2* | 0,94479659 | 0,00061248 |  |  |  |  |
| *NPL* | 1,63243184 | 0,00071893 |  |  |  |  |
| *CNTFR* | 1,17741828 | 0,00072217 |  |  |  |  |
| *ADAMTS19* | 1,42515033 | 0,00072602 |  |  |  |  |
| *MCOLN3* | 1,39445285 | 0,00072822 |  |  |  |  |
| *RASSF5* | 0,98037816 | 0,00073665 |  |  |  |  |
| *WWP1* | 0,7853271 | 0,00085644 |  |  |  |  |
| *ATP6AP2* | 0,84900877 | 0,00093491 |  |  |  |  |
| *RASGRP3* | 0,71315474 | 0,00094885 |  |  |  |  |
| *BMP7* | 1,07090488 | 0,00103277 |  |  |  |  |
| *HMGN3* | 0,7875938 | 0,00109952 |  |  |  |  |
| *VAV1* | 1,17907055 | 0,00112156 |  |  |  |  |
| *ATRN* | 0,57624883 | 0,00116342 |  |  |  |  |
| *CEP170* | 0,54742346 | 0,00118937 |  |  |  |  |
| *BDH2* | 0,73230984 | 0,00128873 |  |  |  |  |
| *CCBL2* | 0,75696501 | 0,0013071 |  |  |  |  |
| *CPT1A* | 0,78168275 | 0,00133591 |  |  |  |  |
| *OSBPL11* | 0,60253348 | 0,00149038 |  |  |  |  |
| *STK26* | 0,99689237 | 0,00151153 |  |  |  |  |
| *MBD5* | 0,69378549 | 0,00161055 |  |  |  |  |
| *PAK7* | 1,19864306 | 0,00168924 |  |  |  |  |
| *ICK* | 0,6474509 | 0,00181811 |  |  |  |  |
| *COL4A3BP* | 0,67389942 | 0,00192848 |  |  |  |  |
| *TNFAIP8* | 0,69274887 | 0,00192943 |  |  |  |  |
| *PLAGL1* | 0,68937453 | 0,00197055 |  |  |  |  |
| *NCEH1* | 0,73575072 | 0,00200686 |  |  |  |  |
| *SPEG* | -2,95504063 | 1,52E-41 |  |  |  |  |
| *ADGRL1* | -2,48628315 | 4,37E-30 |  |  |  |  |
| *PLCG1* | -1,44101607 | 2,21E-14 |  |  |  |  |
| *PPP1R13L* | -1,80117472 | 1,36E-13 |  |  |  |  |
| *COL18A1* | -1,6966492 | 4,28E-12 |  |  |  |  |
| *ROBO4* | -1,6935939 | 1,27E-11 |  |  |  |  |
| *ALS2CL* | -1,90750615 | 6,18E-14 |  |  |  |  |
| *EML3* | -1,10838949 | 1,28E-08 |  |  |  |  |
| *OSBPL7* | -1,6904112 | 4,08E-13 |  |  |  |  |
| *ITGA7* | -2,67935127 | 1,04E-37 |  |  |  |  |
| *ADAMTS10* | -1,48799913 | 4,07E-15 |  |  |  |  |
| *SMTN* | -2,7588366 | 3,25E-21 |  |  |  |  |
| *DDX39B* | -0,58555389 | 0,000592911 |  |  |  |  |
| *RN7SL1* | -2,71642781 | 1,21E-05 |  |  |  |  |
| *CAMTA2* | -1,25293405 | 5,01E-14 |  |  |  |  |
| *MED12* | -0,82456608 | 5,43E-06 |  |  |  |  |
| *CLASRP* | -2,0904125 | 1,39E-15 |  |  |  |  |
| *MPRIP* | -1,72601349 | 6,26E-17 |  |  |  |  |
| *NEURL4* | -1,33440264 | 3,27E-11 |  |  |  |  |
| *SETD1A* | -1,73686746 | 7,26E-13 |  |  |  |  |
| *GRIPAP1* | -2,21891267 | 1,06E-23 |  |  |  |  |
| *TRABD* | -1,99255798 | 3,88E-16 |  |  |  |  |
| *RNF123* | -1,81475324 | 1,51E-23 |  |  |  |  |
| *HID1* | -2,29644133 | 1,88E-17 |  |  |  |  |
| *PKN1* | -0,91137669 | 5,68E-09 |  |  |  |  |
| *ZSWIM8* | -0,53430223 | 0,000725036 |  |  |  |  |
| *BCL9L* | -1,14859458 | 1,01E-05 |  |  |  |  |
| *HAUS5* | -1,28611149 | 4,57E-10 |  |  |  |  |
| *PIDD1* | -2,17493207 | 1,49E-17 |  |  |  |  |
| *CCDC183* | -2,33759552 | 1,71E-08 |  |  |  |  |
| *ULK1* | -1,20741584 | 5,72E-12 |  |  |  |  |
| *GIGYF1* | -1,29764485 | 6,60E-10 |  |  |  |  |
| *STK25* | -1,38040719 | 1,51E-09 |  |  |  |  |
| *RABL6* | -1,45913089 | 1,36E-07 |  |  |  |  |
| *ANKRD52* | -1,10793428 | 1,90E-06 |  |  |  |  |
| *SRRT* | -1,04864152 | 2,29E-07 |  |  |  |  |
| *NDOR1* | -1,8026161 | 1,25E-13 |  |  |  |  |
| *TCAP* | -1,75420991 | 2,61E-15 |  |  |  |  |
| *KLHL17* | -1,66079893 | 8,36E-10 |  |  |  |  |
| *OPLAH* | -1,98113798 | 4,24E-17 |  |  |  |  |
| *FHOD1* | -2,02212645 | 3,45E-26 |  |  |  |  |
| *MORC2* | -1,39238009 | 6,58E-11 |  |  |  |  |
| *DOCK6* | -1,86298547 | 1,36E-18 |  |  |  |  |
| *ATF6B* | -1,24447047 | 3,88E-10 |  |  |  |  |
| *EHBP1L1* | -1,30756174 | 3,42E-07 |  |  |  |  |
| *CXXC1* | -1,85516474 | 7,54E-14 |  |  |  |  |
| *ZBTB40* | -1,71585478 | 8,90E-16 |  |  |  |  |
| *LRWD1* | -1,10951123 | 2,00E-05 |  |  |  |  |
| *TAF1C* | -1,11797867 | 2,47E-06 |  |  |  |  |
| *PPP6R1* | -1,26586393 | 1,76E-07 |  |  |  |  |
| *RNF40* | -1,19911267 | 8,05E-11 |  |  |  |  |
| *AMT* | -0,91151009 | 1,85E-06 |  |  |  |  |
| *U2AF2* | -1,06091269 | 3,67E-07 |  |  |  |  |
| *ADCY4* | -1,58372843 | 8,23E-18 |  |  |  |  |
| *CCDC88B* | -1,74330866 | 2,74E-06 |  |  |  |  |
| *PHRF1* | -1,56551173 | 3,29E-11 |  |  |  |  |
| *AKAP6* | -1,79867713 | 1,78E-08 |  |  |  |  |
| *CABP1* | -2,63608731 | 4,86E-06 |  |  |  |  |
| *BRD3* | -1,47479512 | 1,12E-06 |  |  |  |  |
| *HSF4* | -1,26476282 | 1,01E-05 |  |  |  |  |
| *HDAC7* | -1,34196128 | 3,77E-09 |  |  |  |  |
| *MADD* | -1,86740196 | 1,19E-11 |  |  |  |  |
| *PCBP4* | -1,26761071 | 1,27E-11 |  |  |  |  |
| *MINK1* | -1,47408673 | 3,79E-13 |  |  |  |  |
| *PKN3* | -1,61271177 | 8,27E-16 |  |  |  |  |
| *MICALL1* | -2,01210135 | 8,93E-12 |  |  |  |  |
| *KIFC2* | -1,5014158 | 3,05E-08 |  |  |  |  |
| *PIP5K1C* | -1,34027329 | 1,69E-10 |  |  |  |  |
| *PLEKHJ1* | -1,19585629 | 2,61E-07 |  |  |  |  |
| *TONSL* | -1,43002999 | 3,95E-08 |  |  |  |  |
| *PPP1R18* | -1,29171437 | 1,06E-10 |  |  |  |  |
| *RABEP2* | -1,56604474 | 1,69E-10 |  |  |  |  |
| *SURF6* | -2,18989228 | 2,69E-13 |  |  |  |  |
| *ANO1* | -1,94954777 | 1,93E-13 |  |  |  |  |
| *CASKIN2* | -1,27804837 | 5,10E-15 |  |  |  |  |
| *STARD3* | -1,18064835 | 2,02E-08 |  |  |  |  |
| *ATXN2L* | -0,741817 | 2,94E-05 |  |  |  |  |
| *NCAPH2* | -1,3272042 | 2,38E-10 |  |  |  |  |
| *HDGFRP2* | -1,10243837 | 5,48E-06 |  |  |  |  |
| *GCC1* | -1,65015926 | 1,85E-10 |  |  |  |  |
| *ZNF335* | -1,5181075 | 3,01E-13 |  |  |  |  |
| *DGCR8* | -1,46668712 | 1,82E-09 |  |  |  |  |
| *MYBL2* | -1,48765451 | 5,71E-07 |  |  |  |  |
| *CAD* | -0,69702959 | 0,00129994 |  |  |  |  |
| *TMEM63B* | -1,51047589 | 4,46E-13 |  |  |  |  |
| *RAPGEF3* | -1,85293033 | 2,29E-11 |  |  |  |  |
| *SYMPK* | -1,50164352 | 4,30E-08 |  |  |  |  |
| *DYNC1H1* | -0,93448872 | 8,53E-06 |  |  |  |  |
| *NTRK3* | -2,13517188 | 1,67E-19 |  |  |  |  |
| *LZTR1* | -1,52391933 | 1,72E-11 |  |  |  |  |
| *GTPBP3* | -1,13403797 | 2,91E-07 |  |  |  |  |
| *POLRMT* | -1,4179599 | 2,55E-06 |  |  |  |  |
| *GABBR1* | -1,48711153 | 1,93E-11 |  |  |  |  |
| *COG1* | -0,91498238 | 3,91E-06 |  |  |  |  |
| *PDLIM7* | -1,70005206 | 6,34E-17 |  |  |  |  |
| *NELFA* | -1,64795265 | 1,23E-09 |  |  |  |  |
| *TAOK2* | -1,19752158 | 7,01E-10 |  |  |  |  |
| *C29H11orf84* | -1,18226459 | 1,02E-06 |  |  |  |  |
| *PDE2A* | -1,3354856 | 2,31E-08 |  |  |  |  |
| *GNAT1* | -1,99981746 | 1,34E-17 |  |  |  |  |
| *SLC6A17* | -1,8974378 | 2,16E-05 |  |  |  |  |
| *ZNF629* | -1,04409824 | 2,16E-06 |  |  |  |  |
| *PACSIN3* | -2,50409115 | 2,79E-19 |  |  |  |  |
| *COL27A1* | -2,3793466 | 8,34E-10 |  |  |  |  |
| *KRI1* | -1,47659318 | 1,38E-08 |  |  |  |  |
| *GNB3* | -1,44495279 | 1,00E-05 |  |  |  |  |
| *ACAP1* | -1,21858449 | 4,29E-07 |  |  |  |  |
| *LIG1* | -1,47875336 | 8,20E-07 |  |  |  |  |
| *HECTD3* | -1,11282925 | 5,08E-08 |  |  |  |  |
| *CCDC9* | -1,85580556 | 8,95E-17 |  |  |  |  |
| *SREBF2* | -1,92944895 | 7,12E-08 |  |  |  |  |
| *ARHGEF15* | -1,72812229 | 1,30E-12 |  |  |  |  |
| *ATP2A1* | -2,42548215 | 8,17E-07 |  |  |  |  |
| *ARGLU1* | -1,646298 | 4,60E-13 |  |  |  |  |
| *RTEL1* | -1,43281073 | 1,54E-10 |  |  |  |  |
| *MICALL2* | -2,22468187 | 9,63E-11 |  |  |  |  |
| *VARS2* | -0,95479258 | 3,62E-05 |  |  |  |  |
| *AHDC1* | -1,57698649 | 2,18E-10 |  |  |  |  |
| *MAEA* | -1,00362731 | 1,62E-08 |  |  |  |  |
| *SART1* | -1,35233966 | 1,52E-11 |  |  |  |  |
| *PER2* | -1,81322871 | 9,72E-12 |  |  |  |  |
| *ADRBK1* | -0,64572186 | 0,00167202 |  |  |  |  |
| *PER1* | -1,14595208 | 2,23E-05 |  |  |  |  |
| *MAP3K6* | -1,52436551 | 7,88E-09 |  |  |  |  |
| *LZTS1* | -2,20400248 | 1,76E-13 |  |  |  |  |
| *TAF6L* | -1,0557457 | 5,08E-06 |  |  |  |  |
| *CARD11* | -1,55322987 | 5,76E-15 |  |  |  |  |
| *SHKBP1* | -1,21066314 | 3,74E-07 |  |  |  |  |
| *MYO1C* | -1,22572579 | 4,84E-14 |  |  |  |  |
| *ACAD9* | -1,3252125 | 4,09E-10 |  |  |  |  |
| *SPSB3* | -1,08889927 | 5,40E-05 |  |  |  |  |
| *SYNGAP1* | -1,69087338 | 4,09E-09 |  |  |  |  |
| *PNPLA6* | -1,28532721 | 4,14E-10 |  |  |  |  |
| *MTA1* | -1,44188947 | 2,91E-07 |  |  |  |  |
| *SLC9A5* | -2,13415815 | 1,18E-08 |  |  |  |  |
| *CYHR1* | -0,94415315 | 5,51E-05 |  |  |  |  |
| *CDK11B* | -1,18575386 | 4,82E-06 |  |  |  |  |
| *NUMA1* | -0,62500263 | 0,000231188 |  |  |  |  |
| *TAF6* | -1,35196427 | 1,18E-07 |  |  |  |  |
| *TRIP10* | -0,84283561 | 0,000107993 |  |  |  |  |
| *CCAR2* | -0,85046161 | 3,02E-06 |  |  |  |  |
| *MAPK8IP3* | -1,43873314 | 2,05E-09 |  |  |  |  |
| *NARFL* | -1,41120966 | 1,23E-10 |  |  |  |  |
| *RASGRP2* | -1,95363768 | 2,04E-13 |  |  |  |  |
| *INSC* | -2,40604262 | 2,26E-05 |  |  |  |  |
| *TNNT2* | -2,05208488 | 6,19E-16 |  |  |  |  |
| *IGF2* | -1,11939107 | 2,24E-05 |  |  |  |  |
| *DUS3L* | -1,20055442 | 7,83E-09 |  |  |  |  |
| *PLEKHH3* | -2,21475409 | 1,04E-23 |  |  |  |  |
| *MLXIPL* | -1,85278914 | 5,39E-14 |  |  |  |  |
| *RBM10* | -0,80978149 | 1,09E-05 |  |  |  |  |
| *NOTCH4* | -1,78299087 | 4,60E-25 |  |  |  |  |
| *PHF1* | -1,09813095 | 7,03E-10 |  |  |  |  |
| *PLCB3* | -1,27309286 | 1,26E-05 |  |  |  |  |
| *NOP14* | -1,39678226 | 2,75E-11 |  |  |  |  |
| *HGS* | -0,83436092 | 1,46E-05 |  |  |  |  |
| *TOP3A* | -1,23280439 | 2,13E-10 |  |  |  |  |
| *SSH3* | -1,08075857 | 1,19E-08 |  |  |  |  |
| *ANKRD13D* | -1,59088199 | 1,53E-11 |  |  |  |  |
| *EHMT1* | -1,39715655 | 8,77E-11 |  |  |  |  |
| *TNIP2* | -1,93526178 | 1,03E-12 |  |  |  |  |
| *ARRDC1* | -0,83112013 | 0,000193107 |  |  |  |  |
| *MROH1* | -1,01222197 | 1,09E-07 |  |  |  |  |
| *ARHGEF10L* | -1,34902296 | 3,76E-07 |  |  |  |  |
| *PLA2G4B* | -1,43593346 | 6,95E-11 |  |  |  |  |
| *PPP1R37* | -1,69528857 | 5,14E-11 |  |  |  |  |
| *ITGA2B* | -0,9437498 | 3,42E-05 |  |  |  |  |
| *TNFRSF4* | -1,51389869 | 1,02E-09 |  |  |  |  |
| *DAGLA* | -1,8228756 | 3,11E-08 |  |  |  |  |
| *ADCY6* | -1,67434466 | 8,40E-23 |  |  |  |  |
| *PABPN1* | -0,96080908 | 1,44E-06 |  |  |  |  |
| *TJAP1* | -1,75510461 | 1,23E-08 |  |  |  |  |
| *SMARCA4* | -1,35823434 | 1,90E-11 |  |  |  |  |
| *EHMT2* | -0,98280978 | 1,65E-06 |  |  |  |  |
| *LRCH4* | -1,00265824 | 1,29E-06 |  |  |  |  |
| *USP11* | -1,17984064 | 4,68E-09 |  |  |  |  |
| *AXIN1* | -1,34641777 | 8,50E-10 |  |  |  |  |
| *AZI1* | -1,5804581 | 2,65E-13 |  |  |  |  |
| *NUDT16L1* | -1,28718305 | 5,40E-05 |  |  |  |  |
| *DHX37* | -1,43132092 | 2,16E-09 |  |  |  |  |
| *ANKZF1* | -0,84850991 | 4,84E-05 |  |  |  |  |
| *ABTB1* | -1,16488549 | 2,31E-07 |  |  |  |  |
| *AMPD2* | -1,10820723 | 5,75E-05 |  |  |  |  |
| *TNFRSF10D* | -1,05121599 | 7,95E-07 |  |  |  |  |
| *SPTAN1* | -1,05526498 | 6,91E-08 |  |  |  |  |
| *TCIRG1* | -0,92749317 | 0,00010562 |  |  |  |  |
| *MAPK12* | -2,3732287 | 1,35E-12 |  |  |  |  |
| *CNTNAP1* | -1,02077537 | 0,000226747 |  |  |  |  |
| *CDK3* | -0,946027 | 4,44E-05 |  |  |  |  |
| *NRBP2* | -1,64307987 | 2,46E-09 |  |  |  |  |
| *PPP1R16A* | -1,71082331 | 1,00E-07 |  |  |  |  |
| *ARHGEF17* | -1,97329487 | 9,00E-39 |  |  |  |  |
| *CCDC102A* | -1,41961522 | 1,63E-05 |  |  |  |  |
| *SSNA1* | -1,40374672 | 1,55E-07 |  |  |  |  |
| *LLGL2* | -2,05114115 | 1,27E-12 |  |  |  |  |
| *XPC* | -1,19883005 | 2,76E-08 |  |  |  |  |
| *SLC4A11* | -1,59788853 | 2,70E-08 |  |  |  |  |
| *ZC3H4* | -0,62579724 | 0,000568766 |  |  |  |  |
| *RIPK3* | -0,87535905 | 0,000216346 |  |  |  |  |
| *ELAC2* | -1,18071347 | 1,12E-08 |  |  |  |  |
| *MAP3K12* | -1,292188 | 3,50E-07 |  |  |  |  |
| *DVL1* | -1,43543105 | 4,50E-12 |  |  |  |  |
| *CHMP4A* | -0,88154407 | 0,000130064 |  |  |  |  |
| *IKBKG* | -1,51772521 | 3,03E-15 |  |  |  |  |
| *CORO7* | -1,85450466 | 1,29E-14 |  |  |  |  |
| *EGFL7* | -1,26943952 | 6,02E-09 |  |  |  |  |
| *TGM2* | -1,52400317 | 0,00045501 |  |  |  |  |
| *VPS52* | -0,67388007 | 0,000146156 |  |  |  |  |
| *ITPR3* | -2,09054087 | 1,64E-12 |  |  |  |  |
| *INPPL1* | -0,7231495 | 3,53E-05 |  |  |  |  |
| *DGKD* | -1,52006737 | 1,61E-14 |  |  |  |  |
| *PCNXL3* | -0,70162815 | 0,00201412 |  |  |  |  |
| *MKNK2* | -0,97996769 | 8,41E-05 |  |  |  |  |
| *CYTH2* | -1,02973285 | 4,39E-08 |  |  |  |  |
| *PANK4* | -1,19657114 | 2,09E-08 |  |  |  |  |
| *DHX38* | -0,77752231 | 0,001701319 |  |  |  |  |
| *MYH3* | -1,45515588 | 4,42E-05 |  |  |  |  |
| *DDX41* | -0,85755273 | 0,001383635 |  |  |  |  |
| *TADA3* | -1,06075705 | 2,98E-07 |  |  |  |  |
| *ELF3* | -2,04337765 | 5,22E-07 |  |  |  |  |
| *COL11A2* | -1,70010458 | 4,09E-05 |  |  |  |  |
| *FBF1* | -1,48220634 | 5,33E-10 |  |  |  |  |
| *PLEKHG3* | -1,05271055 | 8,67E-07 |  |  |  |  |
| *CHERP* | -1,07883542 | 6,15E-09 |  |  |  |  |
| *EP400* | -1,09778646 | 6,44E-07 |  |  |  |  |
| *STK19* | -0,67819457 | 0,000154185 |  |  |  |  |
| *ATG16L1* | -0,93430611 | 5,14E-06 |  |  |  |  |
| *ELF4* | -0,95594982 | 2,61E-05 |  |  |  |  |
| *DGKQ* | -1,72566597 | 7,14E-19 |  |  |  |  |
| *OSBP* | -1,65270338 | 1,04E-12 |  |  |  |  |
| *RHBDF1* | -1,5365689 | 6,17E-12 |  |  |  |  |
| *KLC2* | -2,13216933 | 2,90E-12 |  |  |  |  |
| *FAM50A* | -0,7118789 | 0,001179493 |  |  |  |  |
| *PRR14* | -0,76441946 | 0,001219185 |  |  |  |  |
| *KANK3* | -1,36361671 | 4,70E-06 |  |  |  |  |
| *RAD9A* | -1,3196921 | 9,39E-09 |  |  |  |  |
| *USHBP1* | -1,12732876 | 9,79E-06 |  |  |  |  |
| *UBR4* | -0,84322276 | 2,20E-06 |  |  |  |  |
| *SH3GLB2* | -1,23481145 | 3,45E-10 |  |  |  |  |
| *SFN* | -2,01065808 | 2,08E-05 |  |  |  |  |
| *PDE4A* | -1,26258254 | 5,40E-08 |  |  |  |  |
| *FZR1* | -1,39068167 | 6,67E-09 |  |  |  |  |
| *HRC* | -2,13132607 | 1,08E-06 |  |  |  |  |
| *DGCR2* | -1,06522211 | 2,11E-07 |  |  |  |  |
| *IRAK1* | -1,21999499 | 1,74E-05 |  |  |  |  |
| *VASH1* | -1,45598773 | 1,94E-09 |  |  |  |  |
| *PTP4A3* | -2,290366 | 2,04E-14 |  |  |  |  |
| *NAPRT* | -1,02268089 | 5,91E-07 |  |  |  |  |
| *SNRNP70* | -1,62138023 | 3,52E-08 |  |  |  |  |
| *DEPTOR* | -1,930577 | 1,82E-15 |  |  |  |  |
| *ATP13A2* | -1,6693857 | 8,16E-07 |  |  |  |  |
| *EVI5L* | -1,07528284 | 9,02E-08 |  |  |  |  |
| *BOD1L* | -0,8427769 | 2,95E-06 |  |  |  |  |
| *SPG7* | -0,78306401 | 0,000109705 |  |  |  |  |
| *CHST3* | -1,47840763 | 1,44E-11 |  |  |  |  |
| *GGA3* | -0,73448702 | 0,001070675 |  |  |  |  |
| *USP20* | -1,52919669 | 2,86E-14 |  |  |  |  |
| *TCHP* | -0,9965934 | 1,10E-05 |  |  |  |  |
| *MAMDC4* | -1,77905811 | 1,61E-06 |  |  |  |  |
| *RPS6KB2* | -0,90565607 | 6,96E-06 |  |  |  |  |
| *RNF207* | -2,02952423 | 0,000254969 |  |  |  |  |
| *MAP3K14* | -1,31697786 | 8,69E-08 |  |  |  |  |
| *POLR3D* | -1,43914011 | 2,77E-11 |  |  |  |  |
| *ODF2* | -1,21443827 | 3,15E-09 |  |  |  |  |
| *PIH1D1* | -1,61463424 | 4,78E-12 |  |  |  |  |
| *ZNF692* | -1,14616202 | 3,65E-06 |  |  |  |  |
| *SEMA4C* | -0,87202136 | 0,000199941 |  |  |  |  |
| *HOOK2* | -2,12956676 | 4,78E-08 |  |  |  |  |
| *LETM1* | -1,58460851 | 4,43E-10 |  |  |  |  |
| *RRP1* | -1,18575644 | 2,41E-07 |  |  |  |  |
| *AFAP1L2* | -1,75210523 | 1,87E-15 |  |  |  |  |
| *SLC4A2* | -0,97895819 | 0,000817362 |  |  |  |  |
| *EVL* | -1,4304361 | 9,36E-09 |  |  |  |  |
| *HIP1* | -1,62987959 | 4,37E-20 |  |  |  |  |
| *SRSF2* | -0,57164146 | 0,001525889 |  |  |  |  |
| *PTOV1* | -1,37926633 | 7,66E-09 |  |  |  |  |
| *NFATC4* | -1,0934487 | 6,45E-05 |  |  |  |  |
| *DCTN1* | -0,63357589 | 0,000878997 |  |  |  |  |
| *PLD2* | -0,8310039 | 8,01E-05 |  |  |  |  |
| *AP5Z1* | -1,61461126 | 7,87E-12 |  |  |  |  |
| *SPAG7* | -1,16485585 | 1,52E-09 |  |  |  |  |
| *SH2B1* | -0,81548405 | 6,66E-06 |  |  |  |  |
| *ANKS3* | -1,26748307 | 1,16E-08 |  |  |  |  |
| *REXO1* | -0,73830415 | 0,000225709 |  |  |  |  |
| *TBX2* | -1,86322197 | 1,40E-11 |  |  |  |  |
| *SCLY* | -1,47880379 | 1,13E-07 |  |  |  |  |
| *SH2D3C* | -1,39804495 | 5,51E-07 |  |  |  |  |
| *UNC45A* | -1,04353462 | 1,22E-07 |  |  |  |  |
| *GRAMD4* | -1,88844177 | 8,93E-11 |  |  |  |  |
| *APBB3* | -0,85362405 | 1,70E-05 |  |  |  |  |
| *PEX6* | -1,14032963 | 3,76E-09 |  |  |  |  |
| *RPS6KL1* | -1,33150222 | 0,00016444 |  |  |  |  |
| *KAT5* | -0,80427967 | 4,49E-06 |  |  |  |  |
| *TNNT3* | -3,10064891 | 1,51E-24 |  |  |  |  |
| *COL15A1* | -0,89006924 | 0,000615158 |  |  |  |  |
| *IQSEC1* | -1,3519145 | 7,08E-08 |  |  |  |  |
| *DHX30* | -0,98109725 | 2,06E-06 |  |  |  |  |
| *ESPNL* | -2,13190873 | 1,24E-07 |  |  |  |  |
| *MYL4* | -1,65174118 | 9,00E-05 |  |  |  |  |
| *GTF3C1* | -0,98850667 | 5,32E-08 |  |  |  |  |
| *SIRT5* | -1,11874373 | 4,36E-06 |  |  |  |  |
| *SIGIRR* | -1,37925952 | 3,02E-07 |  |  |  |  |
| *ITGB4* | -1,2604046 | 1,01E-05 |  |  |  |  |
| *LDB3* | -2,124188 | 4,05E-05 |  |  |  |  |
| *ARHGEF2* | -0,94787154 | 4,51E-07 |  |  |  |  |
| *ASB6* | -1,28677427 | 3,79E-08 |  |  |  |  |
| *ITIH4* | -1,29141139 | 3,67E-05 |  |  |  |  |
| *ATP13A1* | -0,96454259 | 1,72E-07 |  |  |  |  |
| *TFDP1* | -1,19527871 | 9,28E-08 |  |  |  |  |
| *SH3BP2* | -1,17454437 | 2,89E-06 |  |  |  |  |
| *ZNF608* | -0,95859861 | 4,32E-06 |  |  |  |  |
| *NCLN* | -0,9757956 | 1,82E-05 |  |  |  |  |
| *TNK2* | -1,07431793 | 1,49E-07 |  |  |  |  |
| *CLIP2* | -1,20816151 | 0,000157289 |  |  |  |  |
| *TIE1* | -1,11610887 | 8,82E-06 |  |  |  |  |
| *NFATC2IP* | -1,74727307 | 4,24E-11 |  |  |  |  |
| *BCL6B* | -1,12888913 | 4,49E-07 |  |  |  |  |
| *LAMB3* | -1,2532579 | 8,05E-09 |  |  |  |  |
| *SLC26A10* | -1,06946082 | 0,000724659 |  |  |  |  |
| *SLC6A1* | -1,79776137 | 1,47E-05 |  |  |  |  |
| *LOX* | 1,958943491 | 3,33E-11 |  |  |  |  |
| *A2M* | 2,686130251 | 2,37E-21 |  |  |  |  |
| *SEMA3C* | 2,030303337 | 8,58E-19 |  |  |  |  |
| *PCOLCE2* | 1,781825349 | 9,54E-07 |  |  |  |  |
| *OLFML1* | 2,012107494 | 7,48E-15 |  |  |  |  |
| *PLXDC2* | 1,530500456 | 2,55E-08 |  |  |  |  |
| *C3* | 2,67017886 | 5,94E-17 |  |  |  |  |
| *GNG2* | 0,742905893 | 4,33E-05 |  |  |  |  |
| *MFAP5* | 2,757356807 | 3,82E-25 |  |  |  |  |
| *C1R* | 1,927109297 | 6,58E-13 |  |  |  |  |
| *RNASE4* | 1,891942519 | 1,93E-11 |  |  |  |  |
| *PDGFRA* | 1,860470088 | 1,81E-09 |  |  |  |  |
| *TWSG1* | 0,930651134 | 2,69E-05 |  |  |  |  |
| *GFPT2* | 1,267283307 | 0,000239703 |  |  |  |  |
| *SELENBP1* | 1,777021354 | 3,13E-06 |  |  |  |  |
| *CST3* | 2,173708648 | 4,86E-23 |  |  |  |  |
| *ADGRD1* | 1,282349269 | 1,67E-06 |  |  |  |  |
| *IL1RL1* | 1,310685567 | 6,48E-07 |  |  |  |  |
| *IL13RA1* | 1,252687582 | 2,13E-13 |  |  |  |  |
| *RARRES1* | 2,885294036 | 2,88E-12 |  |  |  |  |
| *CTSV* | 1,971932415 | 1,55E-18 |  |  |  |  |
| *GDA* | 1,414260795 | 5,01E-06 |  |  |  |  |
| *CD164* | 1,788147109 | 1,72E-22 |  |  |  |  |
| *SERPING1* | 2,014988442 | 6,66E-24 |  |  |  |  |
| *CIDEC* | 1,495472796 | 2,90E-06 |  |  |  |  |
| *EPHX1* | 1,670523735 | 5,30E-22 |  |  |  |  |
| *PHYH* | 1,242834196 | 4,04E-05 |  |  |  |  |
| *EFEMP1* | 2,81923826 | 1,50E-12 |  |  |  |  |
| *TMEM254* | 2,249846375 | 2,84E-13 |  |  |  |  |
| *CTSK* | 1,74958279 | 3,12E-09 |  |  |  |  |
| *TNFAIP6* | 1,199138054 | 5,39E-06 |  |  |  |  |
| *DAD1* | 1,202751883 | 1,02E-09 |  |  |  |  |
| *ENPP1* | 0,846980385 | 0,000355913 |  |  |  |  |
| *LOC508666* | 2,420490115 | 7,03E-10 |  |  |  |  |
| *CAT* | 1,25106021 | 1,69E-07 |  |  |  |  |
| *GLCE* | 1,861036854 | 1,09E-11 |  |  |  |  |
| *SCARA5* | 1,279981691 | 0,000696089 |  |  |  |  |
| *FBLN5* | 2,205181048 | 2,23E-23 |  |  |  |  |
| *IGFBP3* | 1,852873569 | 4,00E-13 |  |  |  |  |
| *LAMP2* | 1,397735586 | 4,61E-15 |  |  |  |  |
| *LAPTM4A* | 1,094847899 | 4,47E-08 |  |  |  |  |
| *FSTL1* | 2,086404704 | 7,74E-14 |  |  |  |  |
| *TGFBI* | 2,036188933 | 2,31E-10 |  |  |  |  |
| *AOX1* | 0,997623082 | 4,60E-05 |  |  |  |  |
| *CFB* | 2,147259263 | 1,92E-09 |  |  |  |  |
| *CPA3* | 3,848759829 | 3,16E-30 |  |  |  |  |
| *TIMP2* | 1,303756329 | 1,45E-08 |  |  |  |  |
| *FGL2* | 1,379522824 | 1,68E-08 |  |  |  |  |
| *FGG* | 1,869562368 | 2,20E-06 |  |  |  |  |
| *CDO1* | 1,732278474 | 1,99E-09 |  |  |  |  |
| *NTRK2* | 1,182669253 | 1,18E-06 |  |  |  |  |
| *FUCA2* | 1,94819685 | 6,15E-18 |  |  |  |  |
| *CLU* | 2,029731423 | 1,17E-14 |  |  |  |  |
| *ACSM1* | 1,570641386 | 4,43E-07 |  |  |  |  |
| *CD63* | 0,920764134 | 1,85E-07 |  |  |  |  |
| *TSPAN3* | 0,968323488 | 1,42E-05 |  |  |  |  |
| *CPNE3* | 0,745804769 | 0,000469173 |  |  |  |  |
| *ECM1* | 2,363868171 | 9,52E-12 |  |  |  |  |
| *ITM2B* | 1,597392987 | 1,35E-27 |  |  |  |  |
| *MMP2* | 2,026518637 | 2,64E-12 |  |  |  |  |
| *SFRP4* | 2,495042818 | 1,16E-11 |  |  |  |  |
| *AKR1C4* | 1,57738056 | 1,35E-06 |  |  |  |  |
| *ACKR3* | 1,822888415 | 3,08E-09 |  |  |  |  |
| *CREG1* | 1,211267423 | 1,06E-05 |  |  |  |  |
| *THY1* | 2,010275381 | 3,03E-14 |  |  |  |  |
| *TXNDC12* | 1,240030485 | 4,45E-08 |  |  |  |  |
| *GJA1* | 1,193450033 | 5,82E-05 |  |  |  |  |
| *SERPINF1* | 2,934216685 | 4,40E-13 |  |  |  |  |
| *TSPAN6* | 1,562426224 | 1,07E-08 |  |  |  |  |
| *CFD* | 2,34909419 | 3,08E-16 |  |  |  |  |
| *NID1* | 2,18996374 | 1,29E-34 |  |  |  |  |
| *C2* | 2,063609432 | 2,18E-16 |  |  |  |  |
| *TLR4* | 1,494979976 | 5,42E-11 |  |  |  |  |
| *C5AR2* | 1,500137269 | 4,91E-07 |  |  |  |  |
| *MIR421* | 1,895284373 | 5,34E-08 |  |  |  |  |
| *CCL24* | 1,99002169 | 6,19E-07 |  |  |  |  |
| *DAB2* | 1,800050587 | 8,85E-23 |  |  |  |  |
| *SUOX* | 1,364746146 | 4,57E-06 |  |  |  |  |
| *MFSD1* | 1,250372506 | 1,09E-10 |  |  |  |  |
| *SOD3* | 1,141285938 | 1,34E-05 |  |  |  |  |
| *RNASE6* | 2,213993888 | 1,80E-07 |  |  |  |  |
| *RBPJ* | 0,977143739 | 1,52E-05 |  |  |  |  |
| *MMRN1* | 3,457557131 | 8,34E-30 |  |  |  |  |
| *CTSF* | 1,024442946 | 2,56E-08 |  |  |  |  |
| *FBLN1* | 1,514081399 | 2,76E-09 |  |  |  |  |
| *F2RL2* | 1,484172654 | 1,82E-07 |  |  |  |  |
| *AOC1* | 4,078158577 | 1,83E-36 |  |  |  |  |
| *MGP* | 1,498549771 | 2,87E-05 |  |  |  |  |
| *BRB* | 3,598778521 | 8,62E-19 |  |  |  |  |
| *VNN1* | 1,537797194 | 5,62E-05 |  |  |  |  |
| *KIT* | 2,420708323 | 4,06E-24 |  |  |  |  |
| *SLC31A1* | 0,855760503 | 5,54E-05 |  |  |  |  |
| *DENND4A* | 0,79374598 | 1,26E-06 |  |  |  |  |
| *RPN2* | 1,04001755 | 7,79E-05 |  |  |  |  |
| *LRRN4CL* | 1,870065022 | 2,13E-14 |  |  |  |  |
| *DHRS7* | 0,84510265 | 0,001195793 |  |  |  |  |
| *GINM1* | 0,678963205 | 9,14E-05 |  |  |  |  |
| *GNS* | 1,335170975 | 4,52E-11 |  |  |  |  |
| *HADHB* | 0,808765318 | 0,000286039 |  |  |  |  |
| *B3GNT9* | 1,282156618 | 4,30E-08 |  |  |  |  |
| *GSN* | 1,509553616 | 1,33E-10 |  |  |  |  |
| *STT3A* | 1,07055076 | 8,59E-08 |  |  |  |  |
| *PSENEN* | 1,196274924 | 1,02E-06 |  |  |  |  |
| *C5AR1* | 2,231153502 | 9,91E-07 |  |  |  |  |
| *NT5E* | 1,384986062 | 6,95E-07 |  |  |  |  |
| *TIMP1* | 1,269588171 | 1,16E-06 |  |  |  |  |
| *MAN2B1* | 1,52699526 | 2,68E-17 |  |  |  |  |
| *FMOD* | 2,012510498 | 1,73E-18 |  |  |  |  |
| *FOLH1B* | 1,973742407 | 4,65E-19 |  |  |  |  |
| *KLB* | 1,901212259 | 8,11E-08 |  |  |  |  |
| *CD86* | 2,630581037 | 7,41E-13 |  |  |  |  |
| *CDHR4* | 1,7788961 | 1,27E-06 |  |  |  |  |
| *SRPX2* | 1,977213401 | 8,86E-09 |  |  |  |  |
| *SLC39A1* | 1,082663435 | 1,67E-07 |  |  |  |  |
| *CD52* | 2,334843326 | 3,90E-14 |  |  |  |  |
| *MPEG1* | 3,165377421 | 8,78E-19 |  |  |  |  |
| *BOLA-DRA* | 2,367826101 | 4,78E-18 |  |  |  |  |
| *C3AR1* | 3,21931334 | 6,11E-15 |  |  |  |  |
| *ENPP5* | 2,43983158 | 8,79E-17 |  |  |  |  |
| *HIF1A* | 0,927050945 | 3,83E-05 |  |  |  |  |
| *PROS1* | 1,420128465 | 1,32E-10 |  |  |  |  |
| *CD59* | 1,207634666 | 4,59E-05 |  |  |  |  |
| *GPD2* | 0,891296707 | 2,46E-05 |  |  |  |  |
| *LUM* | 2,006144168 | 1,01E-07 |  |  |  |  |
| *CRISPLD2* | 1,204626761 | 1,88E-10 |  |  |  |  |
| *CD302* | 0,872905809 | 0,001113218 |  |  |  |  |
| *CD99* | 1,075106019 | 1,50E-08 |  |  |  |  |
| *OS9* | 1,154616371 | 9,66E-11 |  |  |  |  |
| *PAMR1* | 1,931830861 | 7,49E-09 |  |  |  |  |
| *SQSTM1* | 0,727007687 | 1,76E-05 |  |  |  |  |
| *QSOX1* | 1,226734648 | 3,03E-08 |  |  |  |  |
| *C7* | 1,283769861 | 5,60E-06 |  |  |  |  |
| *FKBP7* | 1,602442117 | 7,01E-07 |  |  |  |  |
| *ACO1* | 0,72805606 | 0,00013975 |  |  |  |  |
| *CD163* | 3,194074128 | 1,87E-39 |  |  |  |  |
| *F13A1* | 1,98600706 | 7,50E-10 |  |  |  |  |
| *THBD* | 1,411526075 | 7,50E-15 |  |  |  |  |
| *NUCB1* | 1,099024626 | 5,36E-13 |  |  |  |  |
| *GPR137B* | 1,101850111 | 5,50E-06 |  |  |  |  |
| *ALDH1L2* | 0,966149755 | 4,69E-05 |  |  |  |  |
| *MESDC2* | 0,892947235 | 4,52E-05 |  |  |  |  |
| *SUMF1* | 1,489819516 | 1,32E-11 |  |  |  |  |
| *ATP13A3* | 0,946297494 | 3,58E-06 |  |  |  |  |
| *TMED9* | 0,896442737 | 2,81E-05 |  |  |  |  |
