# Supplementary material for: Impact of the severity of negative energy balance on gene expression in the subcutaneous adipose tissue of periparturient primiparous Holstein dairy cows: Identification of potential novel metabolic signals for the reproductive system
Source: PLoS One. 2019 Sep 26;14(9):e0222954. doi: 10.1371/journal.pone.0222954 (PMC6763198; doi:10.1371/journal.pone.0222954)
Supplement: S10 Table — (DOCX) [file pone.0222954.s015.docx]

**S10 Table:** List of differential expressed genes between -4 and 1week peripartum in adipose tissue of cows with MNEB (moderate negative energy balance) highlighted as biomarkers with IPA and their links with reproductive parameters.

| Symbol | Description | Fold change | Pvalue | Location | Types | Biomarker Applications | Reference linking  to reproduction | Specie |
| --- | --- | --- | --- | --- | --- | --- | --- | --- |
| *ACAT1* | acetyl-CoA acetyltransferase 1 | 1.52 | 1.96E-14 | Cytoplasm | enzyme | unspecified application | [62] | Bovine |
| *ADAMTS10* | ADAM metallopeptidase with thrombospondin type 1 motif 10 | -2.06 | 2.48E-19 | Extracellular Space | peptidase | diagnosis | nd |  |
| *ADAMTS2* | ADAM metallopeptidase with thrombospondin type 1 motif 2 | 2.45 | 3.37E-20 | Extracellular Space | peptidase | diagnosis,efficacy | [63] | Bovine |
| *ADGRD1* | adhesion G protein-coupled receptor D1 | 1.21 | 4.46E-05 | Plasma Membrane | G-protein coupled receptor | safety | nd |  |
| *ALOX5* | arachidonate 5-lipoxygenase | 1.40 | 1.80E-07 | Cytoplasm | enzyme | diagnosis,efficacy | nd |  |
| *ANGPT2* | angiopoietin 2 | -1.08 | 1.84E-05 | Extracellular Space | growth factor | diagnosis,efficacy,prognosis,unspecified application | [64] | Bovine |
| *ANTXR2* | ANTXR cell adhesion molecule 2 | 1.05 | 5.87E-06 | Plasma Membrane | transmembrane receptor | diagnosis | nd |  |
| *ANXA1* | annexin A1 | 1.37 | 8.47E-08 | Plasma Membrane | enzyme | diagnosis,prognosis,unspecified application | [65] | Bovine |
| *ANXA2* | annexin A2 | 1.85 | 6.27E-14 | Plasma Membrane | other | diagnosis,unspecified application | [65] | Bovine |
| *ARHGDIB* | Rho GDP dissociation inhibitor beta | 1.33 | 1.41E-05 | Cytoplasm | enzyme | diagnosis | nd |  |
| *ATP2A1* | ATPase sarcoplasmic/endoplasmic reticulum Ca2+ transporting 1 | -2.89 | 1.09E-11 | Cytoplasm | transporter | unspecified application | nd |  |
| *BCAR1* | BCAR1, Cas family scaffold protein | -1.44 | 2.76E-05 | Plasma Membrane | enzyme | prognosis | [66] | Bovine |
| *BMPR1A* | bone morphogenetic protein receptor type 1A | 1.17 | 8.17E-04 | Plasma Membrane | kinase | diagnosis,unspecified application | [67] | Bovine |
| *BTK* | Bruton tyrosine kinase | 1.77 | 1.03E-06 | Cytoplasm | kinase | efficacy | nd |  |
| *CANX* | calnexin | 1.21 | 1.23E-06 | Cytoplasm | other | efficacy | nd |  |
| *CARD11* | caspase recruitment domain family member 11 | -1.96 | 3.46E-10 | Cytoplasm | kinase | efficacy | [68] | Other |
| *CARD9* | caspase recruitment domain family member 9 | -1.87 | 8.52E-07 | Cytoplasm | other | diagnosis | nd |  |
| *CCL21* | C-C motif chemokine ligand 21 | 4.65 | 7.88E-22 | Extracellular Space | cytokine | disease progression | [60] | Bovine |
| *CD44* | CD44 molecule (Indian blood group) | 1.23 | 1.59E-05 | Plasma Membrane | other | diagnosis,disease progression,prognosis,unspecified application | [69] | Bovine |
| *CD52* | CD52 molecule | 2.62 | 7.98E-13 | Plasma Membrane | other | unspecified application | [70] | Other |
| *CFB* | complement factor B | 2.02 | 1.06E-06 | Extracellular Space | peptidase | unspecified application | nd |  |
| *CHI3L1* | chitinase 3 like 1 | 2.93 | 6.70E-20 | Extracellular Space | enzyme | diagnosis,efficacy,prognosis | [71] | Other |
| *CKB* | creatine kinase B | 2.22 | 4.78E-10 | Cytoplasm | kinase | safety | [72] | Other |
| *CLU* | clusterin | 2.89 | 6.83E-20 | Cytoplasm | other | efficacy,unspecified application | [73] | Bovine |
| *COL18A1* | collagen type XVIII alpha 1 chain | -1.74 | 9.24E-12 | Extracellular Space | other | diagnosis | [74] | Bovine |
| *COL1A1* | collagen type I alpha 1 chain | 2.04 | 6.47E-08 | Extracellular Space | other | diagnosis | [75] | Other |
| *CR2* | complement C3d receptor 2 | 2.49 | 1.08E-11 | Plasma Membrane | transmembrane receptor | unspecified application | nd |  |
| *CST3* | cystatin C | 3.10 | 2.03E-32 | Extracellular Space | other | diagnosis,efficacy,prognosis,safety,unspecified application | nd |  |
| *CTSK* | cathepsin K | 1.45 | 3.94E-08 | Cytoplasm | peptidase | efficacy | [76] | Bovine |
| *DDX39B* | DExD-box helicase 39B | -1.16 | 1.08E-07 | Nucleus | enzyme | safety | nd |  |
| *DLG4* | discs large MAGUK scaffold protein 4 | -1.52 | 4.57E-06 | Plasma Membrane | kinase | unspecified application | nd |  |
| *ECM1* | extracellular matrix protein 1 | 2.91 | 8.49E-33 | Extracellular Space | transporter | diagnosis | nd |  |
| *EDN1* | endothelin 1 | -1.74 | 4.66E-10 | Extracellular Space | cytokine | diagnosis,efficacy,prognosis,unspecified application | [77, 78] | Bovine |
| *EGFL7* | EGF like domain multiple 7 | -1.72 | 3.19E-06 | Extracellular Space | other | diagnosis,prognosis | [79] | Other |
| *EMP3* | epithelial membrane protein 3 | 1.85 | 2.04E-06 | Plasma Membrane | other | diagnosis | [80] | Other |
| *ENG* | endoglin | -1.64 | 4.77E-06 | Plasma Membrane | transmembrane receptor | disease progression,efficacy | [81, 82] | Other |
| *ENO1* | enolase 1 | 1.23 | 7.69E-07 | Cytoplasm | enzyme | diagnosis | [83] | Bovine |
| *EPHA2* | EPH receptor A2 | -2.02 | 9.92E-13 | Plasma Membrane | kinase | disease progression | [84, 85] | Other |
| *EPHX1* | epoxide hydrolase 1 | 1.76 | 1.87E-14 | Cytoplasm | peptidase | diagnosis | [86] | Other |
| *ERCC2* | ERCC excision repair 2, TFIIH core complex helicase subunit | -1.23 | 2.06E-05 | Nucleus | enzyme | diagnosis,efficacy,prognosis,safety | nd |  |
| *ERG* | ERG, ETS transcription factor | -1.02 | 3.56E-05 | Nucleus | transcription regulator | prognosis,unspecified application | nd |  |
| *FBLN1* | fibulin 1 | 1.87 | 5.27E-12 | Extracellular Space | other | unspecified application | [87, 88] | Other |
| *FLNB* | filamin B | -1.41 | 2.61E-08 | Cytoplasm | other | unspecified application | nd |  |
| *FN1* | fibronectin 1 | 3.80 | 4.64E-23 | Extracellular Space | enzyme | diagnosis,efficacy,prognosis,unspecified application | [89, 90] | Bovine |
| *FYN* | FYN proto-oncogene, Src family tyrosine kinase | 0.84 | 1.22E-03 | Plasma Membrane | kinase | efficacy | [91] | Other |
| *GAPDH* | glyceraldehyde-3-phosphate dehydrogenase | 1.23 | 3.32E-06 | Cytoplasm | enzyme | diagnosis,unspecified application | [92] | House keeping gene |
| *GHITM* | growth hormone inducible transmembrane protein | 1.38 | 3.61E-09 | Cytoplasm | other | unspecified application | nd |  |
| *GLUD1* | glutamate dehydrogenase 1 | 0.93 | 3.29E-06 | Cytoplasm | enzyme | unspecified application | [93] | Other |
| *GNB3* | G protein subunit beta 3 | -1.82 | 2.09E-06 | Plasma Membrane | enzyme | safety,unspecified application | [94] | Other |
| *GPI* | glucose-6-phosphate isomerase | 0.75 | 1.05E-03 | Extracellular Space | enzyme | unspecified application | [95] | Bovine |
| *GSN* | gelsolin | 1.91 | 5.92E-08 | Extracellular Space | other | disease progression,efficacy | [96] | Bovine |
| *GSTM3* | glutathione S-transferase mu 3 | 2.21 | 1.99E-05 | Cytoplasm | enzyme | diagnosis,prognosis | nd |  |
| *GSTP1* | glutathione S-transferase pi 1 | 1.45 | 6.87E-06 | Cytoplasm | enzyme | diagnosis,efficacy,prognosis,safety,unspecified application | [93] | Bovine |
| *HADH* | hydroxyacyl-CoA dehydrogenase | 1.70 | 1.22E-10 | Cytoplasm | enzyme | unspecified application | nd |  |
| *HADHB* | hydroxyacyl-CoA dehydrogenase trifunctional multienzyme complex subunit beta | 1.26 | 1.58E-08 | Cytoplasm | enzyme | unspecified application | nd |  |
| *HNRNPDL* | heterogeneous nuclear ribonucleoprotein D like | -0.77 | 1.40E-04 | Nucleus | other | unspecified application | nd |  |
| *HP* | haptoglobin | 2.59 | 6.12E-08 | Extracellular Space | peptidase | diagnosis,efficacy,unspecified application | [97-98] | Bovine |
| *HPGD* | 15-hydroxyprostaglandin dehydrogenase | 2.16 | 4.13E-06 | Cytoplasm | enzyme | prognosis | [99] | Bovine |
| *IDH1* | isocitrate dehydrogenase (NADP(+)) 1, cytosolic | 1.94 | 1.98E-16 | Cytoplasm | enzyme | unspecified application | [100] | Bovine |
| *IGF1* | insulin like growth factor 1 | 1.35 | 1.65E-04 | Extracellular Space | growth factor | diagnosis,efficacy,prognosis,safety | [101] | Bovine |
| *IGF2* | insulin like growth factor 2 | -1.66 | 2.67E-09 | Extracellular Space | growth factor | diagnosis,efficacy,prognosis | [102] | Bovine |
| *IKBKB* | inhibitor of nuclear factor kappa B kinase subunit beta | -1.70 | 1.81E-13 | Cytoplasm | kinase | unspecified application | [103] | Bovine |
| *ITGA2B* | integrin subunit alpha 2b | -1.63 | 4.24E-07 | Plasma Membrane | transmembrane receptor | unspecified application | [104] | Bovine |
| *ITGA5* | integrin subunit alpha 5 | -0.97 | 8.03E-05 | Plasma Membrane | transmembrane receptor | prognosis | [104] | Bovine |
| *ITGB4* | integrin subunit beta 4 | -1.53 | 1.33E-05 | Plasma Membrane | transmembrane receptor | diagnosis | [105] | Bovine |
| *ITIH5* | inter-alpha-trypsin inhibitor heavy chain family member 5 | 1.63 | 4.49E-12 | Plasma Membrane | other | prognosis | nd |  |
| *KIT* | KIT proto-oncogene receptor tyrosine kinase | 3.03 | 1.86E-24 | Plasma Membrane | transmembrane receptor | diagnosis,efficacy,prognosis,safety,unspecified application | [106] | Bovine |
| *KITLG* | KIT ligand | 1.28 | 5.81E-06 | Extracellular Space | growth factor | unspecified application | [107, 108] | Bovine |
| *KLK7* | kallikrein related peptidase 7 | 3.78 | 3.60E-14 | Extracellular Space | peptidase | diagnosis | [109] | Other |
| *LGALS1* | galectin 1 | 1.32 | 8.48E-07 | Extracellular Space | other | diagnosis,prognosis | [110, 111] | Bovine |
| *LGALS3BP* | galectin 3 binding protein | 1.94 | 1.92E-10 | Plasma Membrane | transmembrane receptor | prognosis | [112] | Bovine |
| *LMCD1* | LIM and cysteine rich domains 1 | -1.39 | 4.84E-08 | Cytoplasm | transcription regulator | unspecified application | nd |  |
| *LYVE1* | lymphatic vessel endothelial hyaluronan receptor 1 | 2.84 | 6.65E-20 | Plasma Membrane | transmembrane receptor | disease progression | [113, 114] | Bovine |
| *MAP3K12* | mitogen-activated protein kinase kinase kinase 12 | -1.52 | 2.27E-06 | Cytoplasm | kinase | unspecified application | nd |  |
| *MED12* | mediator complex subunit 12 | -1.28 | 7.25E-11 | Nucleus | transcription regulator | unspecified application | nd |  |
| *MICALL1* | MICAL like 1 | -1.96 | 2.37E-09 | Cytoplasm | other | unspecified application | nd |  |
| *MIF* | macrophage migration inhibitory factor | 2.14 | 9.98E-09 | Extracellular Space | cytokine | diagnosis,prognosis,response to therapy | [115] | Bovine |
| *MIR143* | microRNA 143 | -2.03 | 1.92E-04 | Cytoplasm | microRNA | unspecified application | [116] | Bovine |
| *MTA1* | metastasis associated 1 | -1.93 | 3.55E-10 | Nucleus | transcription regulator | diagnosis,response to therapy | [117] | Other |
| *MYH10* | myosin heavy chain 10 | -1.46 | 1.21E-11 | Cytoplasm | enzyme | unspecified application | nd |  |
| *MYH9* | myosin heavy chain 9 | -1.68 | 2.73E-14 | Cytoplasm | enzyme | unspecified application | nd |  |
| *NOS3* | nitric oxide synthase 3 | -2.04 | 1.35E-08 | Cytoplasm | enzyme | disease progression,prognosis | [118, 119] | Bovine |
| *NOTCH4* | notch 4 | -2.43 | 1.04E-20 | Plasma Membrane | transcription regulator | diagnosis | [119] | Bovine |
| *NQO1* | NAD(P)H quinone dehydrogenase 1 | 1.68 | 4.44E-07 | Cytoplasm | enzyme | diagnosis,unspecified application | [120] | Other |
| *NR3C1* | nuclear receptor subfamily 3 group C member 1 | 1.21 | 6.91E-08 | Nucleus | ligand-dependent nuclear receptor | efficacy |  |  |
| *NTRK2* | neurotrophic receptor tyrosine kinase 2 | 2.26 | 1.06E-20 | Plasma Membrane | kinase | diagnosis,disease progression,response to therapy | [121] | Other |
| *NUCB1* | nucleobindin 1 | 1.17 | 1.60E-07 | Cytoplasm | other | diagnosis | nd |  |
| *NUMA1* | nuclear mitotic apparatus protein 1 | -1.36 | 5.80E-08 | Nucleus | other | unspecified application | nd |  |
| *PDGFRA* | platelet derived growth factor receptor alpha | 2.37 | 4.08E-27 | Plasma Membrane | kinase | efficacy,prognosis,safety,unspecified application | nd |  |
| *PEBP1* | phosphatidylethanolamine binding protein 1 | 1.57 | 7.84E-10 | Cytoplasm | other | unspecified application | nd |  |
| *PLA2G7* | phospholipase A2 group VII | 2.21 | 3.66E-15 | Extracellular Space | enzyme | diagnosis,efficacy | nd |  |
| *POLD1* | DNA polymerase delta 1, catalytic subunit | -1.44 | 1.01E-04 | Nucleus | enzyme | diagnosis | nd |  |
| *POSTN* | periostin | 5.95 | 1.49E-73 | Extracellular Space | other | unspecified application | [122] | Bovine |
| *PRDX6* | peroxiredoxin 6 | 1.19 | 1.09E-06 | Cytoplasm | enzyme | unspecified application | [123] | Bovine |
| *PRRC2A* | proline rich coiled-coil 2A | -1.38 | 2.19E-11 | Cytoplasm | other | safety | nd |  |
| *PSEN2* | presenilin 2 | -1.96 | 1.31E-10 | Cytoplasm | peptidase | diagnosis | nd |  |
| *RBP4* | retinol binding protein 4 | 2.12 | 4.77E-11 | Extracellular Space | other | unspecified application | [124] | Bovine |
| *S100B* | S100 calcium binding protein B | 1.51 | 2.00E-07 | Cytoplasm | other | diagnosis,efficacy | [125] | Other |
| *SCD* | stearoyl-CoA desaturase | 7.91 | 1.45E-264 | Cytoplasm | enzyme | diagnosis | [126, 127] | Bovine |
| *SFRP2* | secreted frizzled related protein 2 | 3.36 | 3.77E-27 | Plasma Membrane | transmembrane receptor | diagnosis | [60] | Bovine |
| *SLC2A1* | solute carrier family 2 member 1 | 1.71 | 7.00E-04 | Plasma Membrane | transporter | diagnosis,efficacy | [128] | Bovine |
| *SMTN* | smoothelin | -2.24 | 1.69E-12 | Extracellular Space | other | unspecified application | nd |  |
| *SPARC* | secreted protein acidic and cysteine rich | 2.87 | 8.20E-30 | Extracellular Space | other | disease progression,efficacy | [129] | Bovine |
| *SULT1A1* | sulfotransferase family 1A member 1 | 1.44 | 3.13E-08 | Cytoplasm | enzyme | diagnosis | [130] | Other |
| *TF* | transferrin | 5.45 | 2.30E-70 | Extracellular Space | transporter | efficacy,prognosis,safety,unspecified application | [131] | Bovine |
| *TGIF2* | TGFB induced factor homeobox 2 | -1.12 | 1.41E-03 | Nucleus | transcription regulator | prognosis | [132] | Other |
| *THBD* | thrombomodulin | 1.59 | 6.08E-05 | Plasma Membrane | transmembrane receptor | efficacy | [133] | Other |
| *THY1* | Thy-1 cell surface antigen | 3.38 | 6.37E-44 | Plasma Membrane | other | diagnosis,disease progression | [134] | Other |
| *TIMP1* | TIMP metallopeptidase inhibitor 1 | 2.00 | 1.08E-10 | Extracellular Space | cytokine | diagnosis,disease progression,efficacy,prognosis,unspecified application | [135, 136] | Bovine |
| *TLR4* | toll like receptor 4 | 1.41 | 7.71E-08 | Plasma Membrane | transmembrane receptor | efficacy | [137] | Bovine |
| *TNFAIP6* | TNF alpha induced protein 6 | 2.28 | 7.90E-11 | Extracellular Space | other | unspecified application | [138] | Bovine |
| *TNFRSF4* | TNF receptor superfamily member 4 | -2.32 | 1.29E-09 | Plasma Membrane | transmembrane receptor | diagnosis | nd |  |
| *TNNT2* | troponin T2, cardiac type | -1.96 | 4.07E-06 | Cytoplasm | other | diagnosis,efficacy,prognosis,safety,unspecified application | nd |  |
| *TPI1* | triosephosphate isomerase 1 | 1.41 | 1.66E-07 | Cytoplasm | enzyme | diagnosis,unspecified application | [139] | Bovine |
| *TPM1* | tropomyosin 1 | -2.36 | 1.07E-14 | Cytoplasm | other | diagnosis | nd |  |
| *XPC* | XPC complex subunit, DNA damage recognition and repair factor | -2.07 | 1.42E-13 | Nucleus | other | diagnosis | nd |  |

References :

62. Bowdridge EC, Goravanahally MP, Inskeep EK, Flores JA. Activation of Adenosine Monophosphate-Activated Protein Kinase Is an Additional Mechanism That Participates in Mediating Inhibitory Actions of Prostaglandin F2Alpha in Mature, but Not Developing, Bovine Corpora Lutea. Biol Reprod 2015; 93(1):7.

63. Willis EL, Bridges PJ, Fortune JE. Progesterone receptor and prostaglandins mediate luteinizing hormone-induced changes in messenger RNAs for ADAMTS proteases in theca cells of bovine periovulatory follicles. Mol Reprod Dev 2017; 84(1):55-66.

64. Girard A, Dufort I, Douville G, Sirard MA. Global gene expression in granulosa cells of growing, plateau and atretic dominant follicles in cattle. Reprod Biol Endocrinol 2015; 13:17.

65. Puglisi R, Cambuli C, Capoferri R, Giannino L, Lukaj A, Duchi R, et al. Differential gene expression in cumulus oocyte complexes collected by ovum pick up from repeat breeder and normally fertile Holstein Friesian heifers. Anim Reprod Sci 2013; 141(1-2):26-33.

66. Forde N, Mihm M, Canty MJ, Zielak AE, Baker PJ, Park S, et al. Differential expression of signal transduction factors in ovarian follicle development: a functional role for betaglycan and FIBP in granulosa cells in cattle. Physiol Genomics 2008; 33(2):193-204.

67. Glister C, Satchell L, Knight PG. Changes in expression of bone morphogenetic proteins (BMPs), their receptors and inhibin co-receptor betaglycan during bovine antral follicle development: inhibin can antagonize the suppressive effect of BMPs on thecal androgen production. Reproduction 2010; 140(5):699-712.

68. Kiba A, Banno K, Yanokura M, Asada M, Nakayama Y, Aoki D, et al. Differential mRNA expression profiling in ovarian endometriotic tissue with versus without leuprolide acetate treatment. J Obstet Gynaecol Res 2015; 41(10):1598-1606.

69. Marei WF, Ghafari F, Fouladi-Nashta AA. Role of hyaluronic acid in maturation and further early embryo development of bovine oocytes. Theriogenology 2012; 78(3):670-677.

70. Koyama K, Hasegawa A, Komori S. Functional aspects of CD52 in reproduction. J Reprod Immunol 2009; 83(1-2):56-59.

71. Aziz M, Wissing ML, Naver KV, Faber J, Skouby SO. Polycystic ovary syndrome and low-grade inflammation with special reference to YKL-40. Gynecol Endocrinol 2014; 30(4):311-315.

72. Huddleston HG, Wong KK, Welch WR, Berkowitz RS, Mok SC. Clinical applications of microarray technology: creatine kinase B is an up-regulated gene in epithelial ovarian cancer and shows promise as a serum marker. Gynecol Oncol 2005; 96(1):77-83.

73. Salhab M, Tosca L, Cabau C, Papillier P, Perreau C, Dupont J, et al. Kinetics of gene expression and signaling in bovine cumulus cells throughout IVM in different mediums in relation to oocyte developmental competence, cumulus apoptosis and progesterone secretion. Theriogenology 2011; 75(1):90-104.

74. Melo EO, Cordeiro DM, Pellegrino R, Wei Z, Daye ZJ, Nishimura RC, et al. Identification of molecular markers for oocyte competence in bovine cumulus cells. Anim Genet 2017; 48(1):19-29.

75. Assou S, Haouzi D, Dechaud H, Gala A, Ferrieres A, Hamamah S. Comparative gene expression profiling in human cumulus cells according to ovarian gonadotropin treatments. Biomed Res Int 2013; 2013:354582.

76. Ashry M, Lee K, Mondal M, Datta TK, Folger JK, Rajput SK, et al. Expression of TGFbeta superfamily components and other markers of oocyte quality in oocytes selected by brilliant cresyl blue staining: relevance to early embryonic development. Mol Reprod Dev 2015; 82(3):251-264.

77. Klipper E, Levit A, Mastich Y, Berisha B, Schams D, Meidan R. Induction of endothelin-2 expression by luteinizing hormone and hypoxia: possible role in bovine corpus luteum formation. Endocrinology 2010; 151(4):1914-1922.

78. Shirasuna K, Shimizu T, Hayashi KG, Nagai K, Matsui M, Miyamoto A. Positive association, in local release, of luteal oxytocin with endothelin 1 and prostaglandin F2alpha during spontaneous luteolysis in the cow: a possible intermediatory role for luteolytic cascade within the corpus luteum. Biol Reprod 2007; 76(6):965-970.

79. Campagnolo L, Moscatelli I, Pellegrini M, Siracusa G, Stuhlmann H. Expression of EGFL7 in primordial germ cells and in adult ovaries and testes. Gene Expr Patterns 2008; 8(6):389-396.

80. Taylor V, Suter U. Epithelial membrane protein-2 and epithelial membrane protein-3: two novel members of the peripheral myelin protein 22 gene family. Gene 1996; 175(1-2):115-120.

81. Tal R, Seifer DB, Shohat-Tal A, Grazi RV, Malter HE. Transforming growth factor-beta1 and its receptor soluble endoglin are altered in polycystic ovary syndrome during controlled ovarian stimulation. Fertil Steril 2013; 100(2):538-543.

82. Henriksen R, Gobl A, Wilander E, Oberg K, Miyazono K, Funa K. Expression and prognostic significance of TGF-beta isotypes, latent TGF-beta 1 binding protein, TGF-beta type I and type II receptors, and endoglin in normal ovary and ovarian neoplasms. Lab Invest 1995; 73(2):213-220.

83. Ledgard AM, Smolenski GA, Henderson H, Lee RS. Influence of pathogenic bacteria species present in the postpartum bovine uterus on proteome profiles. Reprod Fertil Dev 2015; 27(2):395-406.

84. Xu Y, Zagoura D, Keck C, Pietrowski D. Expression of Eph receptor tyrosine kinases and their ligands in human Granulosa lutein cells and human umbilical vein endothelial cells. Exp Clin Endocrinol Diabetes 2006; 114(10):590-595.

85. Tandon M, Vemula SV, Mittal SK. Emerging strategies for EphA2 receptor targeting for cancer therapeutics. Expert Opin Ther Targets 2011; 15(1):31-51.

86. Hattori N, Fujiwara H, Maeda M, Fujii S, Ueda M. Epoxide hydrolase affects estrogen production in the human ovary. Endocrinology 2000; 141(9):3353-3365.

87. Singh U, Sun T, Larsson T, Elliott RW, Kostka G, Fundele RH. Expression and functional analysis of fibulin-1 (Fbln1) during normal and abnormal placental development of the mouse. Placenta 2006; 27(9-10):1014-1021.

88. Olijnyk D, Ibrahim AM, Ferrier RK, Tsuda T, Chu ML, Gusterson BA, et al. Fibulin-2 is involved in early extracellular matrix development of the outgrowing mouse mammary epithelium. Cell Mol Life Sci 2014; 71(19):3811-3828.

89. Hatzirodos N, Hummitzsch K, Irving-Rodgers HF, Rodgers RJ. Transcriptome comparisons identify new cell markers for theca interna and granulosa cells from small and large antral ovarian follicles. PLoS One 2015; 10(3):e0119800.

90. Dias FC, Khan MI, Sirard MA, Adams GP, Singh J. Differential gene expression of granulosa cells after ovarian superstimulation in beef cattle. Reproduction 2013; 146(2):181-191.

91. Grossman H, Chuderland D, Ninio-Many L, Hasky N, Kaplan-Kraicer R, Shalgi R. A novel regulatory pathway in granulosa cells, the LH/human chorionic gonadotropin-microRNA-125a-3p-Fyn pathway, is required for ovulation. FASEB J 2015; 29(8):3206-3216.

92. Schoen K, Plendl J, Gabler C, Kaessmeyer S. Identification of stably expressed reference genes for RT-qPCR data normalization in defined localizations of cyclic bovine ovaries. Anat Histol Embryol 2015; 44(3):200-211.

93. Spanaki C, Kotzamani D, Petraki Z, Drakos E, Plaitakis A. Expression of human GLUD1 and GLUD2 glutamate dehydrogenases in steroid producing tissues. Mol Cell Endocrinol 2015; 415:1-11.

94. Dravecka I, Lazurova I, Habalova V. The prevalence of Gly972Arg and C825T polymorphisms in Slovak women with polycystic ovary syndrome and their relation to the metabolic syndrome. Gynecol Endocrinol 2010; 26(5):356-360.

95. Lequarre AS, Grisart B, Moreau B, Schuurbiers N, Massip A, Dessy F. Glucose metabolism during bovine preimplantation development: analysis of gene expression in single oocytes and embryos. Mol Reprod Dev 1997; 48(2):216-226.

96. Forde N, McGettigan PA, Mehta JP, O'Hara L, Mamo S, Bazer FW, et al. Proteomic analysis of uterine fluid during the pre-implantation period of pregnancy in cattle. Reproduction 2014; 147(5):575-587.

97. Rabahi F, Brule S, Sirois J, Beckers JF, Silversides DW, Lussier JG. High expression of bovine alpha glutathione S-transferase (GSTA1, GSTA2) subunits is mainly associated with steroidogenically active cells and regulated by gonadotropins in bovine ovarian follicles. Endocrinology 1999; 140(8):3507-3517.

98. Lavery K, Way A, Killian G. Identification and immunohistochemical localization of a haptoglobin-like protein in the tissues and fluids of the bovine (Bos taurus) ovary and oviduct. Reproduction 2003; 125(6):837-846.

99. Lavery K, Gabler C, Day J, Killian G. Expression of haptoglobin mRNA in the liver and oviduct during the oestrous cycle of cows (Bos taurus). Anim Reprod Sci 2004; 84(1-2):13-26.

100. Bergamo P, Balestrieri M, Carratore V, Abrescia P. Purification of a 240 kDa protein from serum and follicular fluid of water buffalo and its identification as haptoglobin. J Exp Zool 1995; 271(6):452-461.

101. Mani AM, Fenwick MA, Cheng Z, Sharma MK, Singh D, Wathes DC. IGF1 induces up-regulation of steroidogenic and apoptotic regulatory genes via activation of phosphatidylinositol-dependent kinase/AKT in bovine granulosa cells. Reproduction 2010; 139(1):139-151.

102. Spicer LJ, Aad PY. Insulin-like growth factor (IGF) 2 stimulates steroidogenesis and mitosis of bovine granulosa cells through the IGF1 receptor: role of follicle-stimulating hormone and IGF2 receptor. Biol Reprod 2007; 77(1):18-27.

103. Hatzirodos N, Irving-Rodgers HF, Hummitzsch K, Rodgers RJ. Transcriptome profiling of the theca interna from bovine ovarian follicles during atresia. PLoS One 2014; 9(6):e99706.

104. Goossens K, Van Soom A, Van Zeveren A, Favoreel H, Peelman LJ. Quantification of fibronectin 1 (FN1) splice variants, including two novel ones, and analysis of integrins as candidate FN1 receptors in bovine preimplantation embryos. BMC Dev Biol 2009; 9:1.

105. Mitko K, Ulbrich SE, Wenigerkind H, Sinowatz F, Blum H, Wolf E, et al. Dynamic changes in messenger RNA profiles of bovine endometrium during the oestrous cycle. Reproduction 2008; 135(2):225-240.

106. Hatzirodos N, Irving-Rodgers HF, Hummitzsch K, Harland ML, Morris SE, Rodgers RJ. Transcriptome profiling of granulosa cells of bovine ovarian follicles during growth from small to large antral sizes. BMC Genomics 2014; 15:24.

107. Parrott JA, Skinner MK. Direct actions of kit-ligand on theca cell growth and differentiation during follicle development. Endocrinology 1997; 138(9):3819-3827.

108. Parrott JA, Skinner MK. Kit ligand actions on ovarian stromal cells: effects on theca cell recruitment and steroid production. Mol Reprod Dev 2000; 55(1):55-64.

109. Shan SJ, Scorilas A, Katsaros D, Rigault de la Longrais I, Massobrio M, Diamandis EP. Unfavorable prognostic value of human kallikrein 7 quantified by ELISA in ovarian cancer cytosols. Clin Chem 2006; 52(10):1879-1886.

110. Armstrong DL, McGowen MR, Weckle A, Pantham P, Caravas J, Agnew D, et al. The core transcriptome of mammalian placentas and the divergence of expression with placental shape. Placenta 2017; 57:71-78.

111. Baba NA, Panigrahi M, Verma AD, Sadam A, Sulabh S, Chhotaray S, et al. Endometrial transcript profile of progesterone-regulated genes during early pregnancy of Water Buffalo (Bubalus bubalis). Reprod Domest Anim 2019;54(1):100-107.

112. Okumu LA, Fair T, Szekeres-Bartho J, O'Doherty AM, Crowe MA, Roche JF, et al. Endometrial expression of progesterone-induced blocking factor and galectins-1, -3, -9, and -3 binding protein in the luteal phase and early pregnancy in cattle. Physiol Genomics 2011; 43(14):903-910.

113. Nitta A, Shirasuna K, Haneda S, Matsui M, Shimizu T, Matsuyama S, et al. Possible involvement of IFNT in lymphangiogenesis in the corpus luteum during the maternal recognition period in the cow. Reproduction 2011; 142(6):879-892.

114. Nitta A, Shirasuna K, Nibuno S, Bollwein H, Shimizu T, Miyamoto A. Downregulation of lymphatic vessel formation factors in PGF2alpha-induced luteolysis in the cow. J Reprod Dev 2013; 59(3):296-301.

115. Bove SE, Petroff MG, Nishibori M, Pate JL. Macrophage migration inhibitory factor in the bovine corpus luteum: characterization of steady-state messenger ribonucleic acid and immunohistochemical localization. Biol Reprod 2000; 62(4):879-885.

116. Hossain MM, Ghanem N, Hoelker M, Rings F, Phatsara C, Tholen E, et al. Identification and characterization of miRNAs expressed in the bovine ovary. BMC Genomics 2009; 10:443.

117. Huang D, Chen J, Yang C, Wang M. TPX2 silencing mediated by joint action of microvesicles and ultrasonic radiation inhibits the migration and invasion of SKOV3 cells. Mol Med Rep 2018; 17(6):7627-7635.

118. Kobayashi Y, Yamamoto Y, Kageyama S, Hirayama H, Kimura K, Okuda K. Regulation of bovine oviductal NO synthesis by follicular steroids and prostaglandins. Reproduction 2016; 151(6):577-587.

119. Kfir S, Basavaraja R, Wigoda N, Ben-Dor S, Orr I, Meidan R. Genomic profiling of bovine corpus luteum maturation. PLoS One 2018; 13(3):e0194456.

120. Atiomo W, Shafiee MN, Chapman C, Metzler VM, Abouzeid J, Latif A, et al. Expression of NAD(P)H quinone dehydrogenase 1 (NQO1) is increased in the endometrium of women with endometrial cancer and women with polycystic ovary syndrome. Clin Endocrinol (Oxf) 2017; 87(5):557-565.

121. Dorfman MD, Garcia-Rudaz C, Alderman Z, Kerr B, Lomniczi A, Dissen GA, et al. Loss of Ntrk2/Kiss1r signaling in oocytes causes premature ovarian failure. Endocrinology 2014; 155(8):3098-3111.

122. Lussier JG, Diouf MN, Levesque V, Sirois J, Ndiaye K. Gene expression profiling of upregulated mRNAs in granulosa cells of bovine ovulatory follicles following stimulation with hCG. Reprod Biol Endocrinol 2017; 15(1):88.

123. Leyens G, Verhaeghe B, Landtmeters M, Marchandise J, Knoops B, Donnay I. Peroxiredoxin 6 is upregulated in bovine oocytes and cumulus cells during in vitro maturation: role of intercellular communication. Biol Reprod 2004; 71(5):1646-1651.

124. Katska-Ksiazkiewicz L, Lechniak-Cieslak D, Korwin-Kossakowska A, Alm H, Rynska B, Warzych E, et al. Genetical and biotechnological methods of utilization of female reproductive potential in mammals. Reprod Biol 2006; 6 Suppl 1:21-36.

125. Kuge O, Yamakawa Y, Nishijima M. Enhancement of transport-dependent decarboxylation of phosphatidylserine by S100B protein in permeabilized Chinese hamster ovary cells. J Biol Chem 2001; 276(26):23700-23706.

126. Hayashi KG, Ushizawa K, Hosoe M, Takahashi T. Differential genome-wide gene expression profiling of bovine largest and second-largest follicles: identification of genes associated with growth of dominant follicles. Reprod Biol Endocrinol 2010; 8:11.

127. Ndiaye K, Fayad T, Silversides DW, Sirois J, Lussier JG. Identification of downregulated messenger RNAs in bovine granulosa cells of dominant follicles following stimulation with human chorionic gonadotropin. Biol Reprod 2005; 73(2):324-333.

128. Velazquez MA, Hadeler KG, Herrmann D, Kues WA, Ulbrich SE, Meyer HH, et al. In vivo oocyte developmental competence is reduced in lean but not in obese superovulated dairy cows after intraovarian administration of IGF1. Reproduction 2011; 142(1):41-52.

129. Joseph C, Hunter MG, Sinclair KD, Robinson RS. The expression, regulation and function of secreted protein, acidic, cysteine-rich in the follicle-luteal transition. Reproduction 2012; 144(3):361-372.

130. Dunn RT, 2nd, Klaassen CD. Tissue-specific expression of rat sulfotransferase messenger RNAs. Drug Metab Dispos 1998; 26(6):598-604.

131. Guimaraes AL, Pereira SA, Kussano NR, Dode MA. The effect of pre-maturation culture using phosphodiesterase type 3 inhibitor and insulin, transferrin and selenium on nuclear and cytoplasmic maturation of bovine oocytes. Zygote 2016; 24(2):219-229.

132. Hu Y, Yu H, Shaw G, Renfree MB, Pask AJ. Differential roles of TGIF family genes in mammalian reproduction. BMC Dev Biol 2011; 11:58.

133. Cheng Y, Kawamura K, Deguchi M, Takae S, Mulders SM, Hsueh AJ. Intraovarian thrombin and activated protein C signaling system regulates steroidogenesis during the periovulatory period. Mol Endocrinol 2012; 26(2):331-340.

134. Itami S, Tamotsu S, Sakai A, Yasuda K. The roles of THY1 and integrin beta3 in cell adhesion during theca cell layer formation and the effect of follicle-stimulating hormone on THY1 and integrin beta3 localization in mouse ovarian follicles. Biol Reprod 2011; 84(5):986-995.

135. Satoh T, Kobayashi K, Yamashita S, Kikuchi M, Sendai Y, Hoshi H. Tissue inhibitor of metalloproteinases (TIMP-1) produced by granulosa and oviduct cells enhances in vitro development of bovine embryo. Biol Reprod 1994; 50(4):835-844.

136. Goldberg MJ, Moses MA, Tsang PC. Identification of matrix metalloproteinases and metalloproteinase inhibitors in bovine corpora lutea and their variation during the estrous cycle. J Anim Sci 1996; 74(4):849-857.

137. Luttgenau J, Herzog K, Struve K, Latter S, Boos A, Bruckmaier RM, et al. LPS-mediated effects and spatio-temporal expression of TLR2 and TLR4 in the bovine corpus luteum. Reproduction 2016; 151(4):391-399.

138. Price JC, Bromfield JJ, Sheldon IM. Pathogen-associated molecular patterns initiate inflammation and perturb the endocrine function of bovine granulosa cells from ovarian dominant follicles via TLR2 and TLR4 pathways. Endocrinology 2013; 154(9):3377-3386.

139. Cagnone GL, Sirard MA. Transcriptomic signature to oxidative stress exposure at the time of embryonic genome activation in bovine blastocysts. Mol Reprod Dev 2013; 80(4):297-314.
