# Supplementary material for: Impact of the severity of negative energy balance on gene expression in the subcutaneous adipose tissue of periparturient primiparous Holstein dairy cows: Identification of potential novel metabolic signals for the reproductive system
Source: PLoS One. 2019 Sep 26;14(9):e0222954. doi: 10.1371/journal.pone.0222954 (PMC6763198; doi:10.1371/journal.pone.0222954)
Supplement: S11 Table — (DOCX) [file pone.0222954.s016.docx]

**S11 Table:** List of differential expressed genes between 1 and 16 weeks peripartum in adipose tissue of cows with MNEB (moderate negative energy balance) highlighted as biomarkers with IPA and their links with reproductive parameters.

| Symbol | Description | Fold change | P-value | Location | Types | Biomarker Applications | References linking  to reproduction | Specie |
| --- | --- | --- | --- | --- | --- | --- | --- | --- |
| *ANXA5* | annexin A5 | -0.78 | 5.10E-05 | Plasma Membrane | transporter | diagnosis,efficacy | [110] | Bovine |
| *GAPDH* | glyceraldehyde-3-phosphate dehydrogenase | -1.68 | 3.92E-07 | Cytoplasm | enzyme | diagnosis,unspecified application | [92] | Houskeeping gene |
| *H3F3B* | H3 histone family member 3A | -1.02 | 5.80E-05 | Nucleus | other | diagnosis | [92] | Houskeeping gene |
| *HSP90AA1* | heat shock protein 90 alpha family class A member 1 | -1.03 | 1.85E-06 | Cytoplasm | enzyme | safety,unspecified application | [140] | Bovine |
| *LGALS1* | galectin 1 | -1.01 | 3.18E-05 | Extracellular Space | other | diagnosis,prognosis | [110, 111] | Bovine |
| *NOTCH4* | notch 4 | 0.89 | 5.07E-05 | Plasma Membrane | transcription regulator | diagnosis | [119] | Bovine |
| *SCD* | stearoyl-CoA desaturase | -2.47 | 5.91E-12 | Cytoplasm | enzyme | diagnosis | [126, 127] | Bovine |
| *SERPINE1* | serpin family E member 1 | -1.70 | 3.79E-06 | Extracellular Space | other | diagnosis,disease progression,efficacy,prognosis,safety | [141] | Bovine |
| *THY1* | Thy-1 cell surface antigen | -1.33 | 2.14E-07 | Plasma Membrane | other | diagnosis,disease progression | [134] | Other |
| *TXN* | thioredoxin | -1.31 | 2.10E-07 | Cytoplasm | enzyme | efficacy,unspecified application | [142] | Bovine |

References :

92. Schoen K, Plendl J, Gabler C, Kaessmeyer S. Identification of stably expressed reference genes for RT-qPCR data normalization in defined localizations of cyclic bovine ovaries. Anat Histol Embryol 2015; 44(3):200-211.

110. Armstrong DL, McGowen MR, Weckle A, Pantham P, Caravas J, Agnew D, et al. The core transcriptome of mammalian placentas and the divergence of expression with placental shape. Placenta 2017; 57:71-78.

111. Baba NA, Panigrahi M, Verma AD, Sadam A, Sulabh S, Chhotaray S, et al. Endometrial transcript profile of progesterone-regulated genes during early pregnancy of Water Buffalo (Bubalus bubalis). Reprod Domest Anim 2019;5

119. Kfir S, Basavaraja R, Wigoda N, Ben-Dor S, Orr I, Meidan R. Genomic profiling of bovine corpus luteum maturation. PLoS One 2018; 13(3)

126. Hayashi KG, Ushizawa K, Hosoe M, Takahashi T. Differential genome-wide gene expression profiling of bovine largest and second-largest follicles: identification of genes associated with growth of dominant follicles. Reprod Biol Endocrinol 2010; 8:11.

127. Ndiaye K, Fayad T, Silversides DW, Sirois J, Lussier JG. Identification of downregulated messenger RNAs in bovine granulosa cells of dominant follicles following stimulation with human chorionic gonadotropin. Biol Reprod 2005; 73(2):324-333.

134. Itami S, Tamotsu S, Sakai A, Yasuda K. The roles of THY1 and integrin beta3 in cell adhesion during theca cell layer formation and the effect of follicle-stimulating hormone on THY1 and integrin beta3 localization in mouse ovarian follicles. Biol Reprod 2011; 84(5):986-995.

140. Sakatani M, Bonilla L, Dobbs KB, Block J, Ozawa M, Shanker S, et al. Changes in the transcriptome of morula-stage bovine embryos caused by heat shock: relationship to developmental acquisition of thermotolerance. Reprod Biol Endocrinol 2013; 11:3.

141. Hayashi KG, Ushizawa K, Hosoe M, Takahashi T. Differential gene expression of serine protease inhibitors in bovine ovarian follicle: possible involvement in follicular growth and atresia. Reprod Biol Endocrinol 2011; 9:72.

142. Deb GK, Jin JI, Kwon TH, Choi BH, Bang JI, Dey SR, et al. Improved blastocyst development of single cow OPU-derived presumptive zygotes by group culture with agarose-embedded helper embryos. Reprod Biol Endocrinol 2011; 9:121.
