# Supplementary material for: Impact of the severity of negative energy balance on gene expression in the subcutaneous adipose tissue of periparturient primiparous Holstein dairy cows: Identification of potential novel metabolic signals for the reproductive system
Source: PLoS One. 2019 Sep 26;14(9):e0222954. doi: 10.1371/journal.pone.0222954 (PMC6763198; doi:10.1371/journal.pone.0222954)
Supplement: S12 Table — (DOCX) [file pone.0222954.s017.docx]

**S12 Table:** List of differential expressed genes between -4 and 1 week peripartum in adipose tissue of cows with SNEB (severe negative energy balance) highlighted as biomarkers with IPA and their links with reproductive parameters.

| Symbol | Description | Fold change | p-value | Location | Types | Biomarker Applications | References linking  to reproduction | Specie |
| --- | --- | --- | --- | --- | --- | --- | --- | --- |
| *ADRB2* | adrenoceptor beta 2 | -1.28 | 3.27E-06 | Plasma Membrane | G-protein coupled receptor | diagnosis,efficacy,unspecified application | [143] | Bovine |
| *ANPEP* | alanyl aminopeptidase, membrane | -1.18 | 2.15E-06 | Plasma Membrane | peptidase | safety,unspecified application | [142] | Bovine |
| *APOA1* | apolipoprotein A1 | -1.31 | 4.51E-07 | Extracellular Space | transporter | diagnosis,efficacy,unspecified application | [145, 146] | Bovine |
| *BCL6* | B cell CLL/lymphoma 6 | 1.06 | 1.87E-05 | Nucleus | transcription regulator | diagnosis,efficacy,prognosis,unspecified application | nd |  |
| *CACNA1G* | calcium voltage-gated channel subunit alpha1 G | 1.70 | 4.16E-06 | Plasma Membrane | ion channel | diagnosis | nd |  |
| *CDKN1A* | cyclin dependent kinase inhibitor 1A | -1.56 | 1.04E-05 | Nucleus | kinase | diagnosis,efficacy,prognosis,response to therapy | [147] | Bovine |
| *CSF1* | colony stimulating factor 1 | -1.00 | 4.35E-08 | Extracellular Space | cytokine | diagnosis,disease progression,efficacy,prognosis,unspecified application | [148] | Bovine |
| *EIF4EBP1* | eukaryotic translation initiation factor 4E binding protein 1 | -1.09 | 1.69E-04 | Cytoplasm | translation regulator | efficacy | [149] | Other |
| *FABP4* | fatty acid binding protein 4 | -1.06 | 3.33E-08 | Cytoplasm | transporter | disease progression | [150] | Other |
| *GPX3* | glutathione peroxidase 3 | -1.48 | 1.12E-06 | Extracellular Space | enzyme | unspecified application | [89] | Bovine |
| *IGFBP3* | insulin like growth factor binding protein 3 | -1.22 | 1.44E-05 | Extracellular Space | other | diagnosis,disease progression,efficacy,prognosis,safety | [151-153] | Bovine |
| *IL6R* | interleukin 6 receptor | -1.03 | 2.87E-05 | Plasma Membrane | transmembrane receptor | efficacy | [154] | Bovine |
| *INHBB* | inhibin subunit beta B | -1.54 | 1.17E-05 | Extracellular Space | growth factor | efficacy | [155] | Bovine |
| *LDLR* | low density lipoprotein receptor | 1.30 | 1.99E-06 | Plasma Membrane | transporter | disease progression,unspecified application | [119] | Bovine |
| *PDPN* | podoplanin | -1.40 | 1.32E-08 | Plasma Membrane | other | diagnosis,disease progression,prognosis,unspecified application | nd |  |
| *PFKFB1* | 6-phosphofructo-2-kinase/fructose-2,6-biphosphatase 1 | 1.73 | 1.16E-06 | Cytoplasm | kinase | unspecified application | nd |  |
| *PHYH* | phytanoyl-CoA 2-hydroxylase | -1.69 | 1.45E-20 | Cytoplasm | enzyme | unspecified application | nd |  |
| *RARRES1* | retinoic acid receptor responder 1 | -1.08 | 3.31E-05 | Plasma Membrane | other | diagnosis | [89] | Bovine |
| *SLIT2* | slit guidance ligand 2 | 1.30 | 1.13E-06 | Extracellular Space | other | diagnosis | [156] | Bovine |
| *SOD2* | superoxide dismutase 2 | -0.85 | 6.60E-05 | Cytoplasm | enzyme | diagnosis,unspecified application | [157] | Bovine |
| *SREBF1* | sterol regulatory element binding transcription factor 1 | 1.24 | 2.38E-09 | Nucleus | transcription regulator | diagnosis | [158] | Other |
| *THBS1* | thrombospondin 1 | -1.92 | 4.62E-12 | Extracellular Space | other | diagnosis,efficacy | [159, 160] | Bovine |

References:

89. Hatzirodos N, Hummitzsch K, Irving-Rodgers HF, Rodgers RJ. Transcriptome comparisons identify new cell markers for theca interna and granulosa cells from small and large antral ovarian follicles. PLoS One 2015; 10(3):e0119800.

119. Kfir S, Basavaraja R, Wigoda N, Ben-Dor S, Orr I, Meidan R. Genomic profiling of bovine corpus luteum maturation. PLoS One 2018; 13(3):e0194456.

142. Deb GK, Jin JI, Kwon TH, Choi BH, Bang JI, Dey SR, et al. Improved blastocyst development of single cow OPU-derived presumptive zygotes by group culture with agarose-embedded helper embryos. Reprod Biol Endocrinol 2011; 9:121.

143. Luck MR, Munker M. Beta adrenoceptors mediate the catecholamine-induced stimulation of oxytocin secretion from cultured bovine granulosa cells. Reprod Fertil Dev 1991; 3(6):715-723.

145. Regassa A, Rings F, Hoelker M, Cinar U, Tholen E, Looft C, et al. Transcriptome dynamics and molecular cross-talk between bovine oocyte and its companion cumulus cells. BMC Genomics 2011; 12:57.

146. Huang S, Qiao J, Li R, Wang L, Li M. Can serum apolipoprotein C-I demonstrate metabolic abnormality early in women with polycystic ovary syndrome? Fertil Steril 2010; 94(1):205-210.

147. Shimizu T, Hirai Y, Miyamoto A. Expression of cyclins and cyclin-dependent kinase inhibitors in granulosa cells from bovine ovary. Reprod Domest Anim 2013; 48(5):e65-69.

148. Ibrahim S, Salilew-Wondim D, Rings F, Hoelker M, Neuhoff C, Tholen E, et al. Expression pattern of inflammatory response genes and their regulatory micrornas in bovine oviductal cells in response to lipopolysaccharide: implication for early embryonic development. PLoS One 2015; 10(3):e0119388.

149. Palaniappan M, Menon B, Menon KM. Stimulatory effect of insulin on theca-interstitial cell proliferation and cell cycle regulatory proteins through MTORC1 dependent pathway. Mol Cell Endocrinol 2013;366(1):81-89.

150. Abali R, Temel Yuksel I, Yuksel MA, Bulut B, Imamoglu M, Emirdar V, et al. Implications of circulating irisin and Fabp4 levels in patients with polycystic ovary syndrome. J Obstet Gynaecol 2016; 36(7):897-901.

151. Armstrong DG, Baxter G, Hogg CO, Woad KJ. Insulin-like growth factor (IGF) system in the oocyte and somatic cells of bovine preantral follicles. Reproduction 2002; 123(6):789-797.

152. Sirotkin AV, Makarevich AV, Corkins MR, Kotwica J, Kwon HB, Bulla J, et al. Secretory activity of bovine ovarian granulosa cells transfected with sense and antisense insulin-like growth factor (IGF) binding protein-3 and the response to IGF-I, GH, LH, oxytocin and oestradiol. J Mol Endocrinol 2001; 27(3):329-338.

153. Brown TA, Braden TD. Expression of insulin-like growth factor binding protein (IGFBP)-3, and the effects of IGFBP-2 and -3 in the bovine corpus luteum. Domest Anim Endocrinol 2001; 20(3):203-216.

154. Samir M, Glister C, Mattar D, Laird M, Knight PG. Follicular expression of pro-inflammatory cytokines tumour necrosis factor-alpha (TNFalpha), interleukin 6 (IL6) and their receptors in cattle: TNFalpha, IL6 and macrophages suppress thecal androgen production in vitro. Reproduction 2017; 154(1):35-49.

155. Wang S, Liu W, Pang X, Dai S, Liu G. The Mechanism of Melatonin and Its Receptor MT2 Involved in the Development of Bovine Granulosa Cells. Int J Mol Sci 2018; 19(7).

156. Negron-Perez VM, Zhang Y, Hansen PJ. Single-cell gene expression of the bovine blastocyst. Reproduction 2017; 154(5):627-644.

157. Combelles CM, Holick EA, Paolella LJ, Walker DC, Wu Q. Profiling of superoxide dismutase isoenzymes in compartments of the developing bovine antral follicles. Reproduction 2010; 139(5):871-881.

158. Christenson LK, Osborne TF, McAllister JM, Strauss JF, 3rd. Conditional response of the human steroidogenic acute regulatory protein gene promoter to sterol regulatory element binding protein-1a. Endocrinology 2001; 142(1):28-36.

159. Berisha B, Schams D, Rodler D, Sinowatz F, Pfaffl MW. Expression and localization of members of the thrombospondin family during final follicle maturation and corpus luteum formation and function in the bovine ovary. J Reprod Dev 2016; 62(5):501-510.

160. Farberov S, Meidan R. Thrombospondin-1 Affects Bovine Luteal Function via Transforming Growth Factor-Beta1-Dependent and Independent Actions. Biol Reprod 2016; 94(1):25.
