# Supplementary material for: Impact of the severity of negative energy balance on gene expression in the subcutaneous adipose tissue of periparturient primiparous Holstein dairy cows: Identification of potential novel metabolic signals for the reproductive system
Source: PLoS One. 2019 Sep 26;14(9):e0222954. doi: 10.1371/journal.pone.0222954 (PMC6763198; doi:10.1371/journal.pone.0222954)
Supplement: S13 Table — (DOCX) [file pone.0222954.s018.docx]

**S13 Table:** List of differential expressed genes between 1 and 16 weeks peripartum in adipose tissue of cows with SNEB (severe negative energy balance) highlighted as biomarkers with IPA and their links with reproductive parameters.

| ID | Entrez Gene Name | Fold change | P-value | Location | Types | Biomarker Applications | References linking  to reproduction | Specie |
| --- | --- | --- | --- | --- | --- | --- | --- | --- |
| *FABP4* | fatty acid binding protein 4 | 0.90 | 3.09E-08 | Cytoplasm | transporter | disease progression | [149] | Other |
| *PHYH* | phytanoyl-CoA 2-hydroxylase | 1.21 | 4.31E-11 | Cytoplasm | enzyme | unspecified application | nd |  |

Reference :

149. Palaniappan M, Menon B, Menon KM. Stimulatory effect of insulin on theca-interstitial cell proliferation and cell cycle regulatory proteins through MTORC1 dependent pathway. Mol Cell Endocrinol 2013;366(1):81-89.
