# Supplementary material for: Impact of the severity of negative energy balance on gene expression in the subcutaneous adipose tissue of periparturient primiparous Holstein dairy cows: Identification of potential novel metabolic signals for the reproductive system
Source: PLoS One. 2019 Sep 26;14(9):e0222954. doi: 10.1371/journal.pone.0222954 (PMC6763198; doi:10.1371/journal.pone.0222954)
Supplement: S14 Table — (DOCX) [file pone.0222954.s019.docx]

**S14 Table:** List of differential expressed genes in adipose tissue between cows with SNEB (severe negative energy balance) and cows with MNEB (moderate negative energy balance) at 1 week peripartum, highlighted as biomarkers with IPA and their links with reproductive parameters.

| Symbol | Description | Fold change | P-value | Location | Types | Biomarker Applications | References linking  to reproduction | Specie |
| --- | --- | --- | --- | --- | --- | --- | --- | --- |
| *ACAT1* | acetyl-CoA acetyltransferase 1 | 1.09 | 2.16E-06 | Cytoplasm | enzyme | unspecified application | [62] | Bovine |
| *ANXA1* | annexin A1 | 1.16 | 5.61E-05 | Plasma Membrane | enzyme | diagnosis,prognosis,unspecified application | [65] | Bovine |
| *ANXA2* | annexin A2 | 1.04 | 7.95E-04 | Plasma Membrane | other | diagnosis,unspecified application | [65] | Bovine |
| *APOD* | apolipoprotein D | 2.07 | 8.64E-06 | Extracellular Space | transporter | safety | [89, 119] | Bovine |
| *BMPR1B* | bone morphogenetic protein receptor type 1B | -1.73 | 1.20E-03 | Plasma Membrane | kinase | unspecified application | [67, 161] | Bovine |
| *CANX* | calnexin | 1.19 | 1.44E-06 | Cytoplasm | other | efficacy | nd |  |
| *CFTR* | cystic fibrosis transmembrane conductance regulator | -2.32 | 2.95E-04 | Plasma Membrane | ion channel | diagnosis,disease progression | nd |  |
| *CPS1* | carbamoyl-phosphate synthase 1 | -2.49 | 1.27E-04 | Cytoplasm | enzyme | unspecified application | [162] | Other |
| *CYP1B1* | cytochrome P450 family 1 subfamily B member 1 | 1.97 | 1.98E-09 | Cytoplasm | enzyme | diagnosis,efficacy,prognosis,unspecified application | [163] | Other |
| *DUSP1* | dual specificity phosphatase 1 | 0.88 | 4.55E-04 | Nucleus | phosphatase | diagnosis,efficacy | [162, 165] | Bovine |
| *EIF1AX* | eukaryotic translation initiation factor 1A X-linked | 0.89 | 8.16E-05 | Cytoplasm | translation regulator | diagnosis | nd |  |
| *ENO2* | enolase 2 | -1.98 | 4.06E-04 | Cytoplasm | enzyme | diagnosis,efficacy,prognosis,unspecified application | [166] | Other |
| *FITM2* | fat storage inducing transmembrane protein 2 | 1.21 | 1.97E-04 | Cytoplasm | other | unspecified application | nd |  |
| *FOS* | Fos proto-oncogene, AP-1 transcription factor subunit | 1.00 | 1.13E-03 | Nucleus | transcription regulator | diagnosis,efficacy | [90] | Bovine |
| *GC* | GC, vitamin D binding protein | -2.19 | 7.34E-04 | Extracellular Space | transporter | unspecified application | nd |  |
| *GHITM* | growth hormone inducible transmembrane protein | 1.08 | 2.79E-05 | Cytoplasm | other | unspecified application | nd |  |
| *GPX3* | glutathione peroxidase 3 | 1.71 | 2.05E-07 | Extracellular Space | enzyme | unspecified application | [89] | Bovine |
| *HP* | haptoglobin | 2.08 | 1.15E-03 | Extracellular Space | peptidase | diagnosis,efficacy,unspecified application | [98, 99] | Bovine |
| *HSP90AA1* | heat shock protein 90 alpha family class A member 1 | 0.68 | 2.26E-04 | Cytoplasm | enzyme | safety,unspecified application | [140, 167] | Bovine |
| *IGF2BP3* | insulin like growth factor 2 mRNA binding protein 3 | -2.33 | 2.49E-04 | Cytoplasm | translation regulator | diagnosis | [154-153] | Bovine |
| *KRT5* | keratin 5 | -2.16 | 8.91E-04 | Cytoplasm | other | diagnosis,efficacy,unspecified application | [168] | Bovine |
| *LBP* | lipopolysaccharide binding protein | 1.99 | 3.39E-08 | Plasma Membrane | transporter | diagnosis,efficacy | [169] | Bovine |
| *LDHB* | lactate dehydrogenase B | 0.89 | 5.05E-04 | Cytoplasm | enzyme | unspecified application | [170] | Other |
| *PRODH* | proline dehydrogenase 1 | -1.17 | 1.06E-04 | Cytoplasm | enzyme | diagnosis | nd |  |
| *MAP1LC3A* | microtubule associated protein 1 light chain 3 alpha | -0.71 | 6.62E-04 | Cytoplasm | other | efficacy | nd |  |
| *MIF* | macrophage migration inhibitory factor | 1.31 | 1.47E-04 | Extracellular Space | cytokine | diagnosis,prognosis,response to therapy | [115] | Bovine |
| *NOS2* | nitric oxide synthase 2 | -1.37 | 7.01E-04 | Cytoplasm | enzyme | diagnosis | [171] | Bovine |
| *PDHA1* | pyruvate dehydrogenase E1 alpha 1 subunit | 1.01 | 4.26E-06 | Cytoplasm | enzyme | unspecified application | [172] | Other |
| *PRDX6* | peroxiredoxin 6 | 0.87 | 4.48E-04 | Cytoplasm | enzyme | unspecified application | [123] | Bovine |
| *RBP4* | retinol binding protein 4 | 1.72 | 9.92E-06 | Extracellular Space | other | unspecified application | [124] | Bovine |
| *SOD2* | superoxide dismutase 2 | 1.37 | 3.97E-06 | Cytoplasm | enzyme | diagnosis,unspecified application | [157] | Bovine |
| *STAB2* | stabilin 2 | -2.59 | 6.44E-05 | Plasma Membrane | transmembrane receptor | diagnosis | nd |  |
| *TG* | thyroglobulin | -2.16 | 1.19E-04 | Extracellular Space | other | diagnosis,unspecified application | [173] | Bovine |
| *VIM* | vimentin | 0.86 | 1.57E-04 | Cytoplasm | other | diagnosis,efficacy,prognosis,unspecified application | [174] | Bovine |

References :

62. Bowdridge EC, Goravanahally MP, Inskeep EK, Flores JA. Activation of Adenosine Monophosphate-Activated Protein Kinase Is an Additional Mechanism That Participates in Mediating Inhibitory Actions of Prostaglandin F2Alpha in Mature, but Not Developing, Bovine Corpora Lutea. Biol Reprod 2015; 93(1):7.

65. Puglisi R, Cambuli C, Capoferri R, Giannino L, Lukaj A, Duchi R, et al. Differential gene expression in cumulus oocyte complexes collected by ovum pick up from repeat breeder and normally fertile Holstein Friesian heifers. Anim Reprod Sci 2013; 141(1-2):26-33.

67. Glister C, Satchell L, Knight PG. Changes in expression of bone morphogenetic proteins (BMPs), their receptors and inhibin co-receptor betaglycan during bovine antral follicle development: inhibin can antagonize the suppressive effect of BMPs on thecal androgen production. Reproduction 2010; 140(5):699-712.

89. Hatzirodos N, Hummitzsch K, Irving-Rodgers HF, Rodgers RJ. Transcriptome comparisons identify new cell markers for theca interna and granulosa cells from small and large antral ovarian follicles. PLoS One 2015; 10(3):e0119800.

90. Dias FC, Khan MI, Sirard MA, Adams GP, Singh J. Differential gene expression of granulosa cells after ovarian superstimulation in beef cattle. Reproduction 2013; 146(2):181-191.

98. Lavery K, Way A, Killian G. Identification and immunohistochemical localization of a haptoglobin-like protein in the tissues and fluids of the bovine (Bos taurus) ovary and oviduct. Reproduction 2003; 125(6):837-846.

99. Lavery K, Gabler C, Day J, Killian G. Expression of haptoglobin mRNA in the liver and oviduct during the oestrous cycle of cows (Bos taurus). Anim Reprod Sci 2004; 84(1-2):13-26.

115. Bove SE, Petroff MG, Nishibori M, Pate JL. Macrophage migration inhibitory factor in the bovine corpus luteum: characterization of steady-state messenger ribonucleic acid and immunohistochemical localization. Biol Reprod 2000; 62(4):879-885.

119. Kfir S, Basavaraja R, Wigoda N, Ben-Dor S, Orr I, Meidan R. Genomic profiling of bovine corpus luteum maturation. PLoS One 2018; 13(3):e0194456.

123. Leyens G, Verhaeghe B, Landtmeters M, Marchandise J, Knoops B, Donnay I. Peroxiredoxin 6 is upregulated in bovine oocytes and cumulus cells during in vitro maturation: role of intercellular communication. Biol Reprod 2004; 71(5):1646-1651.

124. Katska-Ksiazkiewicz L, Lechniak-Cieslak D, Korwin-Kossakowska A, Alm H, Rynska B, Warzych E, et al. Genetical and biotechnological methods of utilization of female reproductive potential in mammals. Reprod Biol 2006; 6 Suppl 1:21-36.

140. Sakatani M, Bonilla L, Dobbs KB, Block J, Ozawa M, Shanker S, et al. Changes in the transcriptome of morula-stage bovine embryos caused by heat shock: relationship to developmental acquisition of thermotolerance. Reprod Biol Endocrinol 2013; 11:3.

153. Brown TA, Braden TD. Expression of insulin-like growth factor binding protein (IGFBP)-3, and the effects of IGFBP-2 and -3 in the bovine corpus luteum. Domest Anim Endocrinol 2001; 20(3):203-216.

154. Samir M, Glister C, Mattar D, Laird M, Knight PG. Follicular expression of pro-inflammatory cytokines tumour necrosis factor-alpha (TNFalpha), interleukin 6 (IL6) and their receptors in cattle: TNFalpha, IL6 and macrophages suppress thecal androgen production in vitro. Reproduction 2017; 154(1):35-49.

157. Combelles CM, Holick EA, Paolella LJ, Walker DC, Wu Q. Profiling of superoxide dismutase isoenzymes in compartments of the developing bovine antral follicles. Reproduction 2010; 139(5):871-881.

161. Diaz PU, Hein GJ, Belotti EM, Rodriguez FM, Rey F, Amweg AN, et al. BMP2, 4 and 6 and BMPR1B are altered from early stages of bovine cystic ovarian disease development. Reproduction 2016; 152(4):333-350.

162. Pacella-Ince L, Zander-Fox DL, Lane M. Mitochondrial SIRT5 is present in follicular cells and is altered by reduced ovarian reserve and advanced maternal age. Reprod Fertil Dev 2014; 26(8):1072-1083.

163. Gregoraszczuk E, Ptak A. Involvement of caspase-9 but not caspase-8 in the anti-apoptotic effects of estradiol and 4-OH-Estradiol in MCF-7 human breast cancer cells. Endocr Regul 2011; 45(1):3-8.

165. Khan DR, Guillemette C, Sirard MA, Richard FJ. Characterization of FSH signalling networks in bovine cumulus cells: a perspective on oocyte competence acquisition. Mol Hum Reprod 2015; 21(9):688-701.

166. Yoshioka N, Takahashi N, Tarumi W, Itoh MT, Ishizuka B. Gonadotropins up-regulate the expression of enolase 2, but not enolase 1, in the rat ovary. Endocr J 2011; 58(11):941-948.

167. Peng Q, Yang H, Xue S, Shi L, Yu Q, Kuang Y. Secretome profile of mouse oocytes after activation using mass spectrum. J Assist Reprod Genet 2012; 29(8):765-771.

168. Crish J, Conti MA, Sakai T, Adelstein RS, Egelhoff TT. Keratin 5-Cre-driven excision of nonmuscle myosin IIA in early embryo trophectoderm leads to placenta defects and embryonic lethality. Dev Biol 2013; 382(1):136-148.

169. Sina M, Dirandeh E, Deldar H, Shohreh B. Inflammatory status and its relationships with different patterns of postpartum luteal activity and reproductive performance in early lactating Holstein cows. Theriogenology 2018; 108:262-268.
