# Supplementary material for: Impact of the severity of negative energy balance on gene expression in the subcutaneous adipose tissue of periparturient primiparous Holstein dairy cows: Identification of potential novel metabolic signals for the reproductive system
Source: PLoS One. 2019 Sep 26;14(9):e0222954. doi: 10.1371/journal.pone.0222954 (PMC6763198; doi:10.1371/journal.pone.0222954)
Supplement: S15 Table — (DOCX) [file pone.0222954.s020.docx]

**S15 Table:** List of differential expressed genes in adipose tissue between cows with SNEB (severe negative energy balance) and cows with MNEB (moderate negative energy balance) at 16 weeks peripartum, highlighted as biomarkers with IPA and their links with reproductive parameters.

| Symbol | Description | Fold change | P-value | Location | Types | Biomarker Applications | References linking  to reproduction | Specie |
| --- | --- | --- | --- | --- | --- | --- | --- | --- |
| *ACSL1* | acyl-CoA synthetase long chain family member 1 | 1.00 | 2.24E-06 | Cytoplasm | enzyme | unspecified application | [175] | Other |
| *ADAM9* | ADAM metallopeptidase domain 9 | 0.81 | 2.68E-04 | Plasma Membrane | peptidase | efficacy,prognosis | nd |  |
| *AMH* | anti-Mullerian hormone | -2.01 | 7.80E-05 | Extracellular Space | growth factor | efficacy | [174, 176, 177] | Bovine |
| *ANGPT1* | angiopoietin 1 | -1.39 | 1.90E-07 | Extracellular Space | growth factor | disease progression,efficacy,prognosis | [171] | Bovine |
| *ANPEP* | alanyl aminopeptidase, membrane | 1.26 | 7.96E-05 | Plasma Membrane | peptidase | safety,unspecified application | [144] | Bovine |
| *B2M* | beta-2-microglobulin | 1.16 | 1.19E-06 | Plasma Membrane | transmembrane receptor | disease progression,efficacy,safety,unspecified application | [178, 179] | Housekeeping gene |
| *B4GALT1* | beta-1,4-galactosyltransferase 1 | 0.89 | 3.27E-05 | Cytoplasm | enzyme | diagnosis | nd |  |
| *BMP7* | bone morphogenetic protein 7 | 1.07 | 1.03E-03 | Extracellular Space | growth factor | diagnosis | [67, 180] | Bovine |
| *CASP8* | caspase 8 | 0.84 | 1.17E-04 | Nucleus | peptidase | diagnosis,efficacy,prognosis | [181] | Bovine |
| *CCND1* | cyclin D1 | -2.18 | 3.48E-08 | Nucleus | transcription regulator | diagnosis,efficacy,prognosis,response to therapy,unspecified application | [147, 182] | Bovine |
| *CCR5* | C-C motif chemokine receptor 5 (gene/pseudogene) | 2.73 | 1.57E-07 | Plasma Membrane | G-protein coupled receptor | diagnosis,efficacy | nd |  |
| *CD68* | CD68 molecule | 2.57 | 4.74E-11 | Plasma Membrane | other | diagnosis,efficacy | [183] | Bovine |
| *CHI3L1* | chitinase 3 like 1 | 1.77 | 6.20E-06 | Extracellular Space | enzyme | diagnosis,efficacy,prognosis | [71, 184] | Other |
| *CPT1A* | carnitine palmitoyltransferase 1A | 0.78 | 1.34E-03 | Cytoplasm | enzyme | unspecified application | nd |  |
| *CPT2* | carnitine palmitoyltransferase 2 | 1.02 | 6.05E-06 | Cytoplasm | enzyme | unspecified application | nd |  |
| *CRIM1* | cysteine rich transmembrane BMP regulator 1 | -1.41 | 7.93E-11 | Extracellular Space | kinase | unspecified application | [185] | Other |
| *CSF1R* | colony stimulating factor 1 receptor | 1.92 | 1.29E-20 | Plasma Membrane | kinase | prognosis | [186] | Bovine |
| *CTSD* | cathepsin D | 1.07 | 2.98E-09 | Cytoplasm | peptidase | diagnosis,unspecified application | [187] | Bovine |
| *CYP3A4* | cytochrome P450 family 3 subfamily A member 4 | 1.75 | 3.35E-06 | Cytoplasm | enzyme | diagnosis,efficacy,safety | [188] | Bovine |
| *DSC2* | desmocollin 2 | 1.96 | 8.69E-05 | Plasma Membrane | other | unspecified application | [189] | Bovine |
| *ENG* | endoglin | -0.80 | 9.90E-04 | Plasma Membrane | transmembrane receptor | disease progression,efficacy | [81, 82] | Other |
| *EPHA2* | EPH receptor A2 | -1.94 | 1.07E-25 | Plasma Membrane | kinase | disease progression | [84, 85] | Other |
| *ERBB3* | erb-b2 receptor tyrosine kinase 3 | -1.82 | 1.24E-06 | Plasma Membrane | kinase | efficacy,prognosis,safety | [190] | Bovine |
| *ESR1* | estrogen receptor 1 | 1.21 | 7.96E-06 | Nucleus | ligand-dependent nuclear receptor | diagnosis,disease progression,efficacy,prognosis,response to therapy,unspecified application | [191] | Bovine |
| *EZR* | ezrin | -0.72 | 1.30E-03 | Plasma Membrane | other | prognosis | [192] | Bovine |
| *F5* | coagulation factor V | 2.32 | 1.16E-06 | Extracellular Space | other | diagnosis | [193] | Other |
| *FCN1* | ficolin 1 | 2.10 | 4.54E-08 | Extracellular Space | peptidase | efficacy | [194] | Other |
| *FGF1* | fibroblast growth factor 1 | -0.99 | 4.46E-04 | Extracellular Space | growth factor | prognosis | [195] | Bovine |
| *FLNB* | filamin B | -0.93 | 4.16E-04 | Cytoplasm | other | unspecified application | nd |  |
| *GGT1* | gamma-glutamyltransferase 1 | -2.07 | 8.74E-06 | Plasma Membrane | enzyme | efficacy,safety,unspecified application | [196, 197] | Other |
| *GNA12* | G protein subunit alpha 12 | -0.65 | 1.30E-03 | Plasma Membrane | enzyme | response to therapy | nd |  |
| *GPRC5C* | G protein-coupled receptor class C group 5 member C | -1.37 | 1.82E-06 | Plasma Membrane | G-protein coupled receptor | unspecified application | nd |  |
| *HSPA4* | heat shock protein family A (Hsp70) member 4 | -1.13 | 1.72E-05 | Cytoplasm | other | efficacy | [198] | Other |
| *ICAM1* | intercellular adhesion molecule 1 | 0.95 | 1.73E-06 | Plasma Membrane | transmembrane receptor | diagnosis,efficacy,prognosis,unspecified application | [199] | Other |
| *IGF1* | insulin like growth factor 1 | 1.87 | 1.23E-05 | Extracellular Space | growth factor | diagnosis,efficacy,prognosis,safety | [101] | Bovine |
| *IL2RG* | interleukin 2 receptor subunit gamma | 1.67 | 4.01E-06 | Plasma Membrane | transmembrane receptor | unspecified application | nd |  |
| *IL6R* | interleukin 6 receptor | 0.82 | 1.96E-05 | Plasma Membrane | transmembrane receptor | efficacy | [154] | Bovine |
| *ITGA3* | integrin subunit alpha 3 | -1.24 | 6.69E-06 | Plasma Membrane | other | prognosis | [104] | Bovine |
| *ITGAM* | integrin subunit alpha M | 2.16 | 5.53E-11 | Plasma Membrane | transmembrane receptor | efficacy,unspecified application | nd | Other |
| *KLK10* | kallikrein related peptidase 10 | 1.94 | 6.32E-06 | Extracellular Space | peptidase | diagnosis,prognosis,response to therapy | [200] | Other |
| *L1CAM* | L1 cell adhesion molecule | -1.85 | 9.77E-07 | Plasma Membrane | other | unspecified application | [201] | Other |
| *LCP1* | lymphocyte cytosolic protein 1 | 1.63 | 9.81E-08 | Cytoplasm | other | disease progression | [202] | Bovine |
| *LGALS3* | galectin 3 | 1.13 | 5.34E-06 | Extracellular Space | other | diagnosis,unspecified application | [203, 204] | Bovine |
| *LIPA* | lipase A, lysosomal acid type | 2.02 | 2.15E-07 | Cytoplasm | enzyme | unspecified application | nd |  |
| *LMCD1* | LIM and cysteine rich domains 1 | -0.95 | 2.28E-05 | Cytoplasm | transcription regulator | unspecified application | nd |  |
| *LYZ* | lysozyme | 2.02 | 1.47E-06 | Extracellular Space | enzyme | unspecified application | [205] | Bovine |
| *MIR143* | microRNA 143 | -3.01 | 5.74E-16 | Cytoplasm | microRNA | unspecified application | [206] | Other |
| *MIR145* | microRNA 145 | -1.54 | 7.58E-05 | Cytoplasm | microRNA | unspecified application | nd |  |
| *MYLK* | myosin light chain kinase | -1.50 | 6.40E-07 | Cytoplasm | kinase | prognosis | nd |  |
| *NCF1* | neutrophil cytosolic factor 1 | 1.84 | 1.17E-05 | Cytoplasm | enzyme | efficacy | nd |  |
| *NFE2L2* | nuclear factor, erythroid 2 like 2 | 0.87 | 1.38E-04 | Nucleus | transcription regulator | efficacy | [89] | Bovine |
| *NOS3* | nitric oxide synthase 3 | -1.70 | 8.64E-18 | Cytoplasm | enzyme | disease progression,prognosis | [118, 119] | Bovine |
| *NR4A1* | nuclear receptor subfamily 4 group A member 1 | -1.97 | 1.94E-09 | Nucleus | ligand-dependent nuclear receptor | unspecified application | [207] | Bovine |
| *NT5C* | 5', 3'-nucleotidase, cytosolic | -0.82 | 1.21E-03 | Cytoplasm | phosphatase | efficacy | nd |  |
| *PC* | pyruvate carboxylase | -1.53 | 8.51E-07 | Cytoplasm | enzyme | unspecified application | [208] | Bovine |
| *PLIN2* | perilipin 2 | 1.06 | 1.70E-05 | Plasma Membrane | other | disease progression | [209] | Bovine |
| *PRKCQ* | protein kinase C theta | 1.09 | 1.72E-04 | Cytoplasm | kinase | diagnosis | nd |  |
| *PTGDS* | prostaglandin D2 synthase | 1.04 | 1.44E-08 | Cytoplasm | enzyme | efficacy | [210] | Other |
| *PTGER4* | prostaglandin E receptor 4 | 1.05 | 2.06E-05 | Plasma Membrane | G-protein coupled receptor | unspecified application | [211] | Bovine |
| *PTPRC* | protein tyrosine phosphatase, receptor type C | 1.54 | 1.52E-11 | Plasma Membrane | phosphatase | diagnosis,efficacy,unspecified application | nd |  |
| *SCARB2* | scavenger receptor class B member 2 | 1.04 | 2.42E-05 | Plasma Membrane | transmembrane receptor | disease progression | [212] | Other |
| *SDC1* | syndecan 1 | -1.44 | 2.75E-05 | Plasma Membrane | enzyme | efficacy,unspecified application | [213] | Bovine |
| *SLC2A4* | solute carrier family 2 member 4 | -1.39 | 4.44E-07 | Plasma Membrane | transporter | unspecified application | [214] | Bovine |
| *SMARCB1* | SWI/SNF related, matrix associated, actin dependent regulator of chromatin, subfamily b, member 1 | -1.37 | 7.29E-06 | Nucleus | transcription regulator | diagnosis | [215] | Other |
| *SOCS3* | suppressor of cytokine signaling 3 | -1.23 | 1.23E-04 | Cytoplasm | phosphatase | diagnosis,efficacy | [216] | Bovine |
| *SOX10* | SRY-box 10 | -1.74 | 3.69E-05 | Nucleus | transcription regulator | unspecified application | nd |  |
| *SPN* | sialophorin | 2.22 | 1.11E-09 | Plasma Membrane | transmembrane receptor | unspecified application | nd |  |
| *SPP1* | secreted phosphoprotein 1 | 2.55 | 2.24E-13 | Extracellular Space | cytokine | diagnosis,efficacy,unspecified application | [217] | Bovine |
| *SYK* | spleen associated tyrosine kinase | 1.44 | 4.86E-08 | Cytoplasm | kinase | efficacy,response to therapy | nd |  |
| *TAGLN* | transgelin | -1.22 | 1.94E-03 | Cytoplasm | other | diagnosis | [218] | Bovine |
| *THRSP* | thyroid hormone responsive | 1.45 | 4.29E-05 | Nucleus | other | efficacy | nd |  |
| *TLR2* | toll like receptor 2 | 2.19 | 1.39E-08 | Plasma Membrane | transmembrane receptor | diagnosis,efficacy,unspecified application | [137] | Bovine |
| *TMSB4X* | thymosin beta 4 X-linked | -0.70 | 1.07E-03 | Cytoplasm | other | diagnosis | nd |  |
| *TNC* | tenascin C | -2.23 | 3.40E-08 | Extracellular Space | other | diagnosis,response to therapy | [122] | Bovine |
| *TNFRSF11B* | TNF receptor superfamily member 11b | -2.58 | 1.46E-05 | Plasma Membrane | transmembrane receptor | diagnosis,efficacy | nd |  |
| *TPM2* | tropomyosin 2 | -1.64 | 3.98E-06 | Other | other | diagnosis | [219] | Other |
| *UBAC1* | UBA domain containing 1 | -0.94 | 1.43E-05 | Nucleus | other | unspecified application | nd |  |
| *VASP* | vasodilator stimulated phosphoprotein | -0.65 | 1.22E-03 | Plasma Membrane | other | efficacy | nd |  |
| *VCL* | vinculin | -0.96 | 1.29E-04 | Plasma Membrane | enzyme | unspecified application | [220] | Other |
| *VWF* | von Willebrand factor | -0.62 | 1.52E-03 | Extracellular Space | other | diagnosis,efficacy,prognosis | [221] | Bovine |

References:

67. Glister C, Satchell L, Knight PG. Changes in expression of bone morphogenetic proteins (BMPs), their receptors and inhibin co-receptor betaglycan during bovine antral follicle development: inhibin can antagonize the suppressive effect of BMPs on thecal androgen production. Reproduction 2010; 140(5):699-712.

71. Aziz M, Wissing ML, Naver KV, Faber J, Skouby SO. Polycystic ovary syndrome and low-grade inflammation with special reference to YKL-40. Gynecol Endocrinol 2014; 30(4):311-315.

81. Tal R, Seifer DB, Shohat-Tal A, Grazi RV, Malter HE. Transforming growth factor-beta1 and its receptor soluble endoglin are altered in polycystic ovary syndrome during controlled ovarian stimulation. Fertil Steril 2013; 100(2):538-543.

82. Henriksen R, Gobl A, Wilander E, Oberg K, Miyazono K, Funa K. Expression and prognostic significance of TGF-beta isotypes, latent TGF-beta 1 binding protein, TGF-beta type I and type II receptors, and endoglin in normal ovary and ovarian neoplasms. Lab Invest 1995; 73(2):213-220.

84. Xu Y, Zagoura D, Keck C, Pietrowski D. Expression of Eph receptor tyrosine kinases and their ligands in human Granulosa lutein cells and human umbilical vein endothelial cells. Exp Clin Endocrinol Diabetes 2006; 114(10):590-595.

85. Tandon M, Vemula SV, Mittal SK. Emerging strategies for EphA2 receptor targeting for cancer therapeutics. Expert Opin Ther Targets 2011; 15(1):31-51.

101. Mani AM, Fenwick MA, Cheng Z, Sharma MK, Singh D, Wathes DC. IGF1 induces up-regulation of steroidogenic and apoptotic regulatory genes via activation of phosphatidylinositol-dependent kinase/AKT in bovine granulosa cells. Reproduction 2010; 139(1):139-151.

104. Goossens K, Van Soom A, Van Zeveren A, Favoreel H, Peelman LJ. Quantification of fibronectin 1 (FN1) splice variants, including two novel ones, and analysis of integrins as candidate FN1 receptors in bovine preimplantation embryos. BMC Dev Biol 2009; 9:1.

118. Kobayashi Y, Yamamoto Y, Kageyama S, Hirayama H, Kimura K, Okuda K. Regulation of bovine oviductal NO synthesis by follicular steroids and prostaglandins. Reproduction 2016; 151(6):577-587.

119. Kfir S, Basavaraja R, Wigoda N, Ben-Dor S, Orr I, Meidan R. Genomic profiling of bovine corpus luteum maturation. PLoS One 2018; 13(3):e0194456.

122. Lussier JG, Diouf MN, Levesque V, Sirois J, Ndiaye K. Gene expression profiling of upregulated mRNAs in granulosa cells of bovine ovulatory follicles following stimulation with hCG. Reprod Biol Endocrinol 2017; 15(1):88.

137. Luttgenau J, Herzog K, Struve K, Latter S, Boos A, Bruckmaier RM, et al. LPS-mediated effects and spatio-temporal expression of TLR2 and TLR4 in the bovine corpus luteum. Reproduction 2016; 151(4):391-399.

144. Forde N, Spencer TE, Bazer FW, Song G, Roche JF, Lonergan P. Effect of pregnancy and progesterone concentration on expression of genes encoding for transporters or secreted proteins in the bovine endometrium. Physiol Genomics 2010; 41(1):53-62.

147. Shimizu T, Hirai Y, Miyamoto A. Expression of cyclins and cyclin-dependent kinase inhibitors in granulosa cells from bovine ovary. Reprod Domest Anim 2013; 48(5):e65-69.

171. Sousa LM, Mendes GP, Campos DB, Baruselli PS, Papa PC. Equine Chorionic Gonadotropin Modulates the Expression of Genes Related to the Structure and Function of the Bovine Corpus Luteum. PLoS One 2016; 11(10):e0164089.

172. Johnson MT, Freeman EA, Gardner DK, Hunt PA. Oxidative metabolism of pyruvate is required for meiotic maturation of murine oocytes in vivo. Biol Reprod 2007; 77(1):2-8.

173. Mutinati M, Desantis S, Rizzo A, Zizza S, Ventriglia G, Pantaleo M, et al. Localization of thyrotropin receptor and thyroglobulin in the bovine corpus luteum. Anim Reprod Sci 2010; 118(1):1-6.

174. Teh A, Izzati UZ, Mori K, Fuke N, Hirai T, Kitahara G, et al. Histological and immunohistochemical evaluation of granulosa cells during different stages of folliculogenesis in bovine ovaries. Reprod Domest Anim 2018; 53(3):569-581.

175. Dunning KR, Anastasi MR, Zhang VJ, Russell DL, Robker RL. Regulation of fatty acid oxidation in mouse cumulus-oocyte complexes during maturation and modulation by PPAR agonists. PLoS One 2014; 9(2):e87327.

176. Maculan R, Pinto TLC, Moreira GM, Vasconcelos GL, Sanches JA, Rosa RG, et al. Anti-Mullerian Hormone (AMH), antral follicle count (AFC), external morphometrics and fertility in Tabapua cows. Anim Reprod Sci 2018; 189:84-92.

177. Yang MY, Cushman RA, Fortune JE. Anti-Mullerian hormone inhibits activation and growth of bovine ovarian follicles in vitro and is localized to growing follicles. Mol Hum Reprod 2017; 23(5):282-291.

178. Baddela VS, Baufeld A, Yenuganti VR, Vanselow J, Singh D. Suitable housekeeping genes for normalization of transcript abundance analysis by real-time RT-PCR in cultured bovine granulosa cells during hypoxia and differential cell plating density. Reprod Biol Endocrinol 2014; 12:118.

179. Rekawiecki R, Rutkowska J, Kotwica J. Identification of optimal housekeeping genes for examination of gene expression in bovine corpus luteum. Reprod Biol 2012; 12(4):362-367.

180. Glister C, Regan SL, Samir M, Knight P. Gremlin, Noggin, Chordin and follistatin differentially modulate BMP induced suppression of androgen secretion by bovine ovarian theca cells. J Mol Endocrinol 2018; 62 (1): 15-25

181. Sinderewicz E, Grycmacher K, Boruszewska D, Kowalczyk-Zieba I, Staszkiewicz J, Slezak T, et al. Expression of factors involved in apoptosis and cell survival is correlated with enzymes synthesizing lysophosphatidic acid and its receptors in granulosa cells originating from different types of bovine ovarian follicles. Reprod Biol Endocrinol 2017; 15(1):72.

182. Totty ML, Morrell BC, Spicer LJ. Fibroblast growth factor 9 (FGF9) regulation of cyclin D1 and cyclin-dependent kinase-4 in ovarian granulosa and theca cells of cattle. Mol Cell Endocrinol 2017; 440:25-33.

183. Irving-Rodgers HF, van Wezel IL, Mussard ML, Kinder JE, Rodgers RJ. Atresia revisited: two basic patterns of atresia of bovine antral follicles. Reproduction 2001; 122(5):761-775.

184. Celik C, Abali R, Guzel S, Bastu E, Kucukyalcin V, Yilmaz M. Elevated circulating levels of YKL-40 are a marker of abnormal glucose tolerance in women with polycystic ovary syndrome. Clin Endocrinol (Oxf) 2012; 77(6):893-897.

185. Pennisi DJ, Wilkinson L, Kolle G, Sohaskey ML, Gillinder K, Piper MJ, et al. Crim1KST264/KST264 mice display a disruption of the Crim1 gene resulting in perinatal lethality with defects in multiple organ systems. Dev Dyn 2007; 236(2):502-511.

186. Cui XS, Xu YN, Shen XH, Zhang LQ, Zhang JB, Kim NH. Trichostatin A modulates apoptotic-related gene expression and improves embryo viability in cloned bovine embryos. Cell Reprogram 2011; 13(2):179-189.

187. Aboelenain M, Kawahara M, Balboula AZ, Montasser Ael M, Zaabel SM, Okuda K, et al. Status of autophagy, lysosome activity and apoptosis during corpus luteum regression in cattle. J Reprod Dev 2015; 61(3):229-236.

188. Moore SG, Scully S, Browne JA, Fair T, Butler ST. Genetic merit for fertility traits in Holstein cows: V. Factors affecting circulating progesterone concentrations. J Dairy Sci 2014; 97(9):5543-5557.

189. Algarra B, Maillo V, Aviles M, Gutierrez-Adan A, Rizos D, Jimenez-Movilla M. Effects of recombinant OVGP1 protein on in vitro bovine embryo development. J Reprod Dev 2018; 64(5):433-443.

190. Hall VJ, Ruddock NT, French AJ. Expression profiling of genes crucial for placental and preimplantation development in bovine in vivo, in vitro, and nuclear transfer blastocysts. Mol Reprod Dev 2005; 72(1):16-24.

191. Rovani MT, Gasperin BG, Ilha GF, Ferreira R, Bohrer RC, Duggavathi R, et al. Expression and molecular consequences of inhibition of estrogen receptors in granulosa cells of bovine follicles. J Ovarian Res 2014; 7:96.

192. Nakamura K, Kusama K, Bai R, Sakurai T, Isuzugawa K, Godkin JD, et al. Induction of IFNT-Stimulated Genes by Conceptus-Derived Exosomes during the Attachment Period. PLoS One 2016; 11(6):e0158278.

193. Annamalai AE, Stewart GJ, Hansel B, Memoli M, Chiu HC, Manuel DW, et al. Expression of factor V on human umbilical vein endothelial cells is modulated by cell injury. Arteriosclerosis 1986; 6(2):196-202.

194. Rorvig S, Honore C, Larsson LI, Ohlsson S, Pedersen CC, Jacobsen LC, et al. Ficolin-1 is present in a highly mobilizable subset of human neutrophil granules and associates with the cell surface after stimulation with fMLP. J Leukoc Biol 2009; 86(6):1439-1449.

195. Han P, Guerrero-Netro H, Estienne A, Cao B, Price CA. Regulation and action of early growth response 1 in bovine granulosa cells. Reproduction 2017; 154(4):547-557.

196. Mahata P: Biomarkers for epithelial ovarian cancers. Genome Inform 2006; 17(2):184-193.

197. Xu X, Qin L, Tian Y, Wang M, Li G, Du Y, et al. Family-based analysis of GGT1 and HNF1A gene polymorphisms in patients with polycystic ovary syndrome. Reprod Biomed Online 2018; 36(1):115-119.

198. Pennarossa G, Maffei S, Rahman MM, Berruti G, Brevini TA, Gandolfi F. Characterization of the constitutive pig ovary heat shock chaperone machinery and its response to acute thermal stress or to seasonal variations. Biol Reprod 2012; 87(5):119.

199. Diamanti-Kandarakis E, Alexandraki K, Piperi C, Protogerou A, Katsikis I, Paterakis T, et al. Inflammatory and endothelial markers in women with polycystic ovary syndrome. Eur J Clin Invest 2006; 36(10):691-697.

200. Koh SC, Razvi K, Chan YH, Narasimhan K, Ilancheran A, Low JJ, et al. Ovarian Cancer Research Consortium of SEA: The association with age, human tissue kallikreins 6 and 10 and hemostatic markers for survival outcome from epithelial ovarian cancer. Arch Gynecol Obstet 2011; 284(1):183-190.

201. Moulla A, Miliaras D, Sioga A, Kaidoglou A, Economou L. The immunohistochemical expression of CD24 and CD171 adhesion molecules in borderline ovarian tumors. Pol J Pathol 2013; 64(3):180-184.

202. Penagaricano F, Souza AH, Carvalho PD, Driver AM, Gambra R, Kropp J, et al. Effect of maternal methionine supplementation on the transcriptome of bovine preimplantation embryos. PLoS One 2013; 8(8):e72302.

203. Hashiba K, Sano M, Nio-Kobayashi J, Hojo T, Skarzynski DJ, Okuda K. Galectin-3 contributes to luteolysis by binding to Beta 1 integrin in the bovine corpus luteum. Biol Reprod 2014; 91(1):2.

204. Kim M, Kim S, Kim H, Kim H, Joo HG, Shin T. Immunohistochemical localization of galectin-3 in the reproductive organs of the cow. Acta Histochem 2008; 110(6):473-480.

205. Hoelker M, Salilew-Wondim D, Drillich M, Christine GB, Ghanem N, Goetze L, et al. Transcriptional response of the bovine endometrium and embryo to endometrial polymorphonuclear neutrophil infiltration as an indicator of subclinical inflammation of the uterine environment. Reprod Fertil Dev 2012; 24(6):778-793.

206. Nteeba J, Ortinau LC, Perfield JW, 2nd, Keating AF. Diet-induced obesity alters immune cell infiltration and expression of inflammatory cytokine genes in mouse ovarian and peri-ovarian adipose depot tissues. Mol Reprod Dev 2013; 80(11):948-958.

207. Jiang Z, Guerrero-Netro HM, Juengel JL, Price CA. Divergence of intracellular signaling pathways and early response genes of two closely related fibroblast growth factors, FGF8 and FGF18, in bovine ovarian granulosa cells. Mol Cell Endocrinol 2013; 375(1-2):97-105.

208. Castro N, Kawashima C, van Dorland HA, Morel I, Miyamoto A, Bruckmaier RM. Metabolic and energy status during the dry period is crucial for the resumption of ovarian activity postpartum in dairy cows. J Dairy Sci 2012; 95(10):5804-5812.

209. Gamarra G, Ponsart C, Lacaze S, Nuttinck F, Cordova A, Mermillod P, et al. Oral propylene glycol modifies follicular fluid and gene expression profiles in cumulus-oocyte complexes and embryos in feed-restricted heifers. Reprod Fertil Dev 2018; 30(3):417-429.

210. Farhat A, Philibert P, Sultan C, Poulat F, Boizet-Bonhoure B. Hematopoietic-Prostaglandin D2 synthase through PGD2 production is involved in the adult ovarian physiology. J Ovarian Res 2011; 4:3.

211. Nuttinck F, Gall L, Ruffini S, Laffont L, Clement L, Reinaud P, et al. PTGS2-related PGE2 affects oocyte MAPK phosphorylation and meiosis progression in cattle: late effects on early embryonic development. Biol Reprod 2011; 84(6):1248-1257.

212. Webb NR, Connell PM, Graf GA, Smart EJ, de Villiers WJ, de Beer FC, et al. SR-BII, an isoform of the scavenger receptor BI containing an alternate cytoplasmic tail, mediates lipid transfer between high density lipoprotein and cells. J Biol Chem 1998; 273(24):15241-15248.

213. Bergqvist AS, Rodriguez-Martinez H. Sulphated glycosaminoglycans (S-GAGs) and syndecans in the bovine oviduct. Anim Reprod Sci 2006; 93(1-2):46-60.

214. Franca MR, Mesquita FS, Lopes E, Pugliesi G, Van Hoeck V, Chiaratti MR, et al. Modulation of periovulatory endocrine profiles in beef cows: consequences for endometrial glucose transporters and uterine fluid glucose levels. Domest Anim Endocrinol 2015; 50:83-90.

215. Clarke BA, Witkowski L, Ton Nu TN, Shaw PA, Gilks CB, Huntsman D, et al: Loss of SMARCA4 (BRG1) protein expression as determined by immunohistochemistry in small-cell carcinoma of the ovary, hypercalcaemic type distinguishes these tumours from their mimics. Histopathology 2016; 69(5):727-738.

216. Rovani MT, Ilha GF, Gasperin BG, Nobrega JE, Jr., Siddappa D, Glanzner WG, et al. Prostaglandin F2alpha-induced luteolysis involves activation of Signal transducer and activator of transcription 3 and inhibition of AKT signaling in cattle. Mol Reprod Dev 2017; 84(6):486-494.

217. Poole DH, Ndiaye K, Pate JL. Expression and regulation of secreted phosphoprotein 1 in the bovine corpus luteum and effects on T lymphocyte chemotaxis. Reproduction 2013; 146(6):527-537.

218. Talbot NC, Powell AM, Caperna TJ, Garrett WM. Proteomic analysis of the major cellular proteins of bovine trophectoderm cell lines derived from IVP, parthenogenetic and nuclear transfer embryos: Reduced expression of annexins I and II in nuclear transfer-derived cell lines. Anim Reprod Sci 2010; 120(1-4):187-202.

219. Li L, Mo H, Zhang J, Zhou Y, Peng X, Luo X. The Role of Heat Shock Protein 90B1 in Patients with Polycystic Ovary Syndrome. PLoS One 2016; 11(4):e0152837.

220. Zhang SM, Yu LL, Qu T, Hu Y, Yuan DZ, Zhang S, et al. The Changes of Cytoskeletal Proteins Induced by the Fast Effect of Estrogen in Mouse Blastocysts and Its Roles in Implantation. Reprod Sci 2017; 24(12):1639-1646.

221. Woad KJ, Hammond AJ, Hunter M, Mann GE, Hunter MG, Robinson RS. FGF2 is crucial for the development of bovine luteal endothelial networks in vitro. Reproduction 2009; 138(3):581-588.
